# Assessing the uncertainty of treatment outcomes in a previous systematic review of venous leg ulcer randomized controlled trials: Additional secondary analysis

**DOI:** 10.1111/wrr.12897

**Published:** 2021-02-08

**Authors:** Kristen A. Eckert, Marissa J. Carter

**Affiliations:** ^1^ Strategic Solutions, Inc Bozeman Montana USA

**Keywords:** randomized controlled trials, risk of bias, trial design, uncertainty of outcome, venous leg ulcers

## Abstract

In this secondary analysis of a previous systematic review, we assessed randomized controlled trials evaluating treatments of venous leg ulcers in terms of factors that affect risk of bias at the study level and thus uncertainty of outcomes obtained from the interventions. Articles that assessed the wound bed condition in venous leg ulcers and that were published in English between 1998 and May 22, 2018 were previously searched in PubMed, Embase, CINAHL, CENTRAL, Scopus, Science Direct, and Web of Science. Duplicates and retracted articles were excluded. The following data were extracted to assess the risk of bias: treatment groups; primary and secondary endpoints that were statistically tested between groups, including their results and *p* values; whether blinding of patients and assessors was done; whether allocation concealment was adequate; whether an intention‐to‐treat analysis was conducted; whether an appropriate power calculation was correctly done; and whether an appropriate multiplicity adjustment was made, as necessary. Pre‐ and post‐study power calculations were made. The step‐up Hochberg procedure adjusted for multiplicity. Results were analysed for all studies, pre‐2013 studies, and 2013/post‐2013 studies. We included 142 randomized controlled trials that evaluated 14,141 patients. Most studies lacked blinding (72.5–77.5%) and allocation concealment (88.7%). Only 49.3% of trials provided a power calculation, with 27.5% having an appropriate calculation correctly done. Adequate statistical power of the primary endpoint was found in 27.2% of trials. The lack of multiplicity adjustment in 98.6% of studies affected the uncertainty of outcomes in 20% of studies, with the majority of the secondary endpoints (67.7%) in those studies becoming non‐significant after multiplicity adjustment. Recent studies tended to weakly demonstrate improved certainty of outcomes. Venous leg ulcer randomized controlled trials have a high degree of uncertainty associated with treatment outcomes. Greater attention to trial design and conduct is needed to improve the evidence base.

## 1 INTRODUCTION

There is a glaring gap between evidence and clinical practice in wound care, with many clinicians relying solely on their clinical experience and a traditional approach to care.^1^, ^2^ The application of evidence‐based medicine (EBM) to wound care is further complicated by the diverse variation in wound types and treatment options. Consequently, many clinical practice guidelines and recommendations have been based on expert opinion.^2^ Limited evidence produced from wound care randomized controlled trials (RCTs) is a result of poorly designed studies that are underpowered with small sample sizes, have too short follow‐up periods to be able to properly assess wound outcomes, and employ poor analysis of endpoints.^3^, ^4^, ^5^ The lack of a sound and applicable evidence base in wound care results in great clinical uncertainty that clouds clinical decision‐making and can contribute to the use of suboptimal treatments, inequalities in care, and wasted resources.^3^, ^6^, ^7^, ^8^, ^9^ Uncertainty in outcome effects has also been a focal point of the GRADE system,^10^ which has now been extended to the concept of the threshold or ranges to rate certainty of the evidence for an individual outcome.^11^

Venous leg ulcers (VLUs) are among the most ubiquitous types of wound,^12^ with an annual incidence rate estimated to be greater than 2%, costing the United States up to $14.9 billion each year.^13^ Considered to be the highest level of evidence,^1^, ^2^ systematic reviews are the most relevant vehicle to evaluate the certainty of RCT outcomes. In health care, they are now used to develop clinical practice guidelines and are often required as a prerequisite to research funding.^14^, ^15^ A well‐conducted systematic review can produce more reliable, precise, and generalizable results with limited bias to be used by providers, payers, researchers, and policymakers for therapeutic advancements.^14^, ^16^ To be able to properly assess the bias, uncertainty of outcomes, and external validity of VLU RCTs, a systematic review must assess the randomization process, allocation concealment, blinding, power analysis, attrition rates, study group similarities, eligibility criteria, primary outcome measures, the inclusion of an intention‐to‐treat (ITT) analysis, and multiplicity adjustment of secondary endpoints.^1^, ^14^, ^17^, ^18^

In 2019, Gethin et al published a systematic review of 144 RCTs involved in the treatment of VLUs to assess the quality of reporting of data related to their external validity.^19^

Their results showed there was inadequate reporting of factors that aid the clinician in determining the applicability of research findings to their patient population, despite the recommendations from CONSORT being available for over 20 years. Generalizability of studies is 1 of the 5 key domains of the GRADE approach to conducting systematic reviews.^20^ The goal of our study was to assess the same RCTs in terms of other factors that could affect risk of bias at the study level and assess certainty of outcomes obtained from the interventions. We therefore sought to determine the uncertainty of outcomes for patients with VLUs treated with any drug, biologic, or device compared to standard of care or placebo.

## 2 MATERIALS AND METHODS

### 2.1 Study selection

We included the same studies selected by Gethin et al in their 2019 systematic review.^19^ They included 144 RCTs published in English between 1998 and May 22, 2018 that assessed the wound bed condition. They excluded any studies of non‐venous wounds and any studies that they did not have open access to from their search. The following online databases were searched: PubMed, Embase, CINAHL, CENTRAL, Scopus, Science Direct, and Web of Science. We screened the original article selection to confirm there were no duplicates and no retracted articles.

### 2.2 Data extraction

One of us (KAE) extracted the following data from the studies into Word files, which were used to assess the risk of bias at the study level: (1) treatment groups; (2) primary and secondary endpoints that were statistically tested between groups, including results of these endpoints for treatment groups and *p* values; (3) whether blinding of patients and outcomes assessment was done; (4) whether allocation concealment was adequate; (5) whether an ITT analysis was conducted for the primary endpoint; (6) whether a power calculation for the primary endpoint was reported that was appropriate and correctly done; and (7) whether an appropriate adjustment was made for multiplicity of statistical testing of secondary endpoints if more than 1 endpoint was tested. The other study author (M.J.C.) independently verified the data and collated it in Excel sheets for each study.

### 2.3 Assessments and definitions

Unlike traditional systematic reviews, we scored assessments as binary (yes or no); for example, if an ITT analysis was performed, this assessment was scored as ‘yes’ If any assessment was unclear, it was scored as a ‘no’; for example, the authors stated patient blinding was done but did not provide supporting evidence. While we know that unclear assessments in bias components do not automatically equate to high risk assessments,^21^ there has been no universal agreement on how to treat this issue. Consequently, it was decided to accept this loss of information in order to present the data in a simpler fashion.

If no primary endpoint(s) could be identified, the most relevant and/or prominent endpoint was chosen. Secondary endpoints were defined as any remaining endpoint that was tested statistically between treatment groups regardless of whether such endpoints were explicitly identified as such by the study authors. Evidence of successful blinding and allocation concealment had to be supported by detailed statements in the study reporting.

The ITT population was defined as all patients who were randomized to treatment groups. Exceptions were patients who were inappropriately randomized; that is, consent form not signed, or patient later found to be ineligible due to inclusion/exclusion criteria.

To be appropriate, a primary endpoint power calculation had to be congruent with the primary endpoint it supported, with reasonable assumptions, method(s), and sufficient data that a power calculation could be replicated. If the calculation was incorrectly performed by the study authors, the result was scored as a ‘no’.

Any discrepancies between our initial independent assessments were resolved by consensus.

### 2.4 Statistical analysis

Pre‐ and post‐study power calculations were made using Pass13 (NCSS, LLC, Kaysville, UT) software with (a) the figures supplied in the study, if a power calculation was done by the study authors and (b) figures based on the results of the primary endpoint for the ITT population, if available, or another population, if that was used by the study authors. Results were categorized in terms of statistical power: (a) <80; (b) 81–89; and (c) ≥90.

Attrition rates for all treatment groups—that is, those patients whose outcomes became right‐censored—were calculated based on the primary length of each study and expressed as a percentage of total patients in each treatment group. The overall attrition rate was calculated, as well as whether there was a difference of ≥20% between any treatment groups.^22^

Adjustment for multiplicity of statistical testing used the step‐up Hochberg procedure and was executed in Excel. The *p* values of all endpoints that were statistically tested with the exception of primary endpoint(s) were entered into the adjustment calculation. If there were co‐primary endpoints, these were entered in a separate calculation. If actual *p* values were not reported but it was clear from the text that a statistical test was carried out, the following conservative *p* value imputations were made: non‐significant: 0.06; <0.05; 0.04; <0.01; 0.009; <0.001; 0.0009.

### 2.5 Reporting

The percentage of studies in which patients were blinded was calculated for all studies, pre‐2013 studies, and 2013/post‐2013 studies. The same procedure was followed for blinded study assessment; adequate allocation concealment; ITT analysis (primary endpoint); reporting a study power calculation; appropriate power calculation; appropriate adjustment for multiplicity of statistical testing of secondary endpoints, if more than 1 endpoint was tested; the number of studies in which at least one secondary endpoint became statistically non‐significant after adjustment; and the percentage of secondary endpoints that became statistically non‐significant after adjustment.

The mean (standard deviation [SD]; range) of attrition rates across study breakpoints was also calculated.

## 3 RESULTS

We included 142/144 (98.6%) articles analysed in the Gethin et al systematic review in our analysis (Supporting Information S1). One study was excluded, because it was retracted by the journal (No. 40), and another article was excluded for being a duplicate study (No. 1). One reference from the original systematic review (No. 89) was replaced with another article (Iglesias et al, 2004) published in the same year that had more complete information and data for the purposes of our analysis. There were 14,141 patients evaluated in the included studies. Complete study characteristics and data are provided in Supporting Information S1. Among the included studies, 97 (68.3%) were published before 2013 and 45 (31.7%) were published in 2013 or later.

### 3.1 Risk of bias assessment

While blinding and adequate allocation concealment was not reported in the majority of studies (72–89%), almost 63% of studies conducted an ITT analysis on the primary endpoint (Table 1). Better trends were evident for all study characteristics for later year studies with the exception of patient blinding, which was lower; study assessor blinding increased by almost the same amount.

**TABLE 1:** Percentage of studies (*n*) with adequate blinding, allocation concealment, and ITT analysis conducted for the primary endpoint

Study characteristic	All studies (*n* = 142)	Studies published before 2013 (*n* = 97)	Studies published in 2013 and later (*n* = 45)
Patients blinded	22.5% (31)	24.7% (23)	17.8% (8)
Study assessors blinded	27.5% (38)	24.7% (23)	33.3% (15)
Allocation concealment	11.3% (16)	9.3% (9)	15.6% (7)
ITT analysis	62.7% (89)	61.9% (60)	64.4% (28)

Abbreviation: ITT, intention‐to‐treat.

Seventy (49.3%) studies reported a power calculation; 48 (49.5%) were studies published before 2013 and 22 (48.9%) were studies published in 2013 or later. Among all studies, 27.5% (39/142) had a power calculation that was appropriate and correctly done. Among all studies published before 2013, 28.9% (28/97) had a power calculation that was appropriate and correctly done compared to 24.4% (11/45) of all studies published in 2013 or later. Forty‐eight (33.8%) of all studies, 33 (34%) studies published before 2013, and 15 (33.3%) published in 2013 or later reported an initial power calculation of the primary endpoints. There were 136 (95.8%) total studies, 92 (94.8%) studies published before 2013, and 44 (97.8%) studies published in 2013 or later in which a post‐study calculation could be done regarding the primary endpoint. In the majority of trials, pre‐trial calculations were optimistic in terms of statistical power, with post‐trial results showing only 27.2% of trials having an adequate power (Table 2). Overall, there was a positive trend for pre‐trial power but a negative trend for post‐trial power in respect of newer versus older studies.

**TABLE 2:** Percentage of studies (*n*) for statistical power categories of the primary endpoint before and after the trial

Statistical power	All studies	Studies published before 2013	Studies published in 2013 and later
Initial power calculation			
≥90	18.8% (9)	21.2% (7)	13.3% (2)
80–89	53.1% (25)	48.5% (16)	66.7% (10)
<80	27.1% (13)	30.3% (10)	20.0% (3)
Final power calculation			
≥90	17.6% (23)	19.6% (18)	13.6% (6)
80–89	9.6% (13)	9.8% (9)	9.1% (4)
<80	72.8% (99)	70.7% (65)	77.3% (33)

Only two studies (1.4%) had an appropriate adjustment for multiplicity of statistical testing of secondary endpoints (if more than 1 endpoint was tested). Both studies were published in 2013 or later (4.4%). Nearly one fifth of studies were negatively affected by multiplicity of statistical testing with over two thirds of endpoints becoming statistically non‐significant (Table 3). Although the results varied little with respect to time, the percentage of endpoints that became statistically non‐significant was lower in newer versus older studies.

**TABLE 3:** Multiplicity adjustment analysis: (A) percentage of studies (*n*) in which one or more secondary endpoints became statistically non‐significant; and (B) percentage of the affected endpoints that became statistically non‐significant

Analysis	All studies	Studies published before 2013	Studies published in 2013 and later
(A) Percentage of studies (*n*)	19.7% (27)	20.6% (20)	17.8% (8)
(B) Percentage of affected endpoints (SD; range)	67.7% (32.1; 1–100)	71.0% (30.1; 17–100)	59.5% (37.4; 1–100)

Of all the studies, 127 (89.4%) had 2 treatment arms, 14 (9.6%) had 3 treatment arms, 7 (4.9%) had 4 treatment arms, 5 (3.5%) had 5 treatment arms and 1 (0.7%) had 10 treatment arms. Studies published in 2013 or later only had 2 treatment arms. The mean attrition rate for all 142 studies regardless of number of treatment arms was 10.9%, with attrition rates much better in later studies compared to more recent ones (Table 4). There were only eight studies (5.6%) in which there was greater than 20% difference reported between any treatment groups, equally divided into the two time periods. There were 29 studies (20.4%) with a total attrition rate of 20% or greater. Twenty‐five of these studies (25.8%) were published before 2013, and 4 studies (8.9%) were published in 2013 or later.

**TABLE 4:** Mean attrition rates (%) by treatment group (SD; range)

Group no.	All studies	Studies published before 2013	Studies published in 2013 and later
All groups combined	10.9 (12.0; 0.0–73.7)	12.4 (13.0; 0.0–73.7)	7.6 (8.5; 0.0–35.0)
Group 1	9.0 (11.7; 0.0–89.5)	9.5 (12.5; 0.0–89.5)	8.2 (10.1; 0.0–39.1)
Group 2	10.4 (12.6; 0.0–57.9)	12.4 (13.8; 0.0–57.9)	6.7 (9.3; 0.0–40.0)
Group 3	4.5 (6.9; 0.0–22.2)	5.3 (7.2; 0.0–22.2)	0.0^a^
Group 4	1.6 (4.2; 0.0–11.1)	2.2 (5.0; 0.0–11.1)	0.0^a^
Group 5	1.6 (3.6; 0.0–8.0)	2.7 (4.6; 0.0–8.0)	0.0^a^
Group 6‐10^a^	0.0	0.0	Not applicable

^a^ *n* = 1 study.

## 4 DISCUSSION

### 4.1 Risk of bias and outcome uncertainty

Assessing the certainty (or uncertainty) of treatment outcomes in a given population for a chronic disease is an important criterion needed by clinicians and payers to determine whether a specific treatment should be employed. Many factors can influence that degree of certainty and our assessment of several of those factors shows that the majority of the RCTs involving treatment of VLUs included in this study have one or more issues that affect the certainty of results. While meta‐analysis can provide higher statistical power through pooling of outcome data, heterogeneous trial design frequently limits this approach.^1^, ^23^, ^24^, ^25^, ^26^, ^27^, ^28^ Moreover, meta‐analysis ignores the many factors that increase risk of bias so the final result itself (point estimate and 95% confidence intervals) can still be biased.^24^, ^25^, ^26^, ^27^, ^28^ Metaregression or multilevel meta‐analysis can potentially adjust for study parameter differences but can be limited by small sample sizes and other issues, and it requires considerable expertise.^29^

High risk of bias featured prominently in both lack of blinding (about three quarters of trials) and lack of allocation concealment (approximately 9 out of 10 trials; Table 1). Although statistical power was consistent (Table 2), less than half of the trials evaluated (49.3%) provided a power calculation, and only 27.5% had an appropriate calculation that was correctly done, with sufficient information provided to replicate the calculation. More recent studies demonstrated a positive trend for pre‐trial power and a negative trend for post‐trial power compared to older studies. In particular, the finding that only 23% of studies had an adequately powered primary endpoint is disturbing. The lack of multiplicity adjustment in almost all studies (98.6%) ultimately affected the uncertainty of outcomes in 1 out of 5 studies, with the majority of the secondary endpoints (67.7%) in those studies becoming statistically non‐significant after we conducted multiplicity adjustment (Table 3). These figures improved slightly in newer studies.

The strengths of these trials were that ITT analysis was performed in the majority (62.7%) (Table 1), and attrition rates were generally low (Table 4), with recent studies reporting much lower attrition rates than older studies. While there were no drastic differences between newer and older studies, recent studies tended to weakly demonstrate improved certainty of outcomes. However, our analysis also shows that VLU RCTs predominantly demonstrate a high risk of bias and low certainty of outcomes.

### 4.2 Levels of evidence

Distilling these results into whether an individual RCT meets level 1 evidence requires a great deal of judgment. If we just take into account that an RCT has to be properly powered ≥80% and has <20% in regard to attrition (Oxford Centre for Evidence‐based Medicine),^30^ then only 13.7% of studies would be ranked at this level. Moreover, our analysis has demonstrated that many of these studies had serious design and/or reporting flaws that weakened their evidence and external validity. We concur with Gethin et al, that these findings are at least partially due to study authors not reporting trial information in sufficient detail,^19^, ^31^ and we know from firsthand experience how authors are limited to journal word counts that can lead to shortened descriptions of the randomization and allocation concealment methods, as an example. Authors from the trials in the review tended to use the sealed envelope method for allocation concealment, but they glossed over the details to ensure that the envelopes were not tampered or manipulated prior to subject allocation. Perhaps, where word limitations are enforced, authors could include a Supporting Information where they further elaborate their allocation concealment methods to ensure trial integrity. Another issue is that many studies suffer from English language issues, which can lead to ambiguity or misinterpretation of key points. Nevertheless, there needs to be a subcategory ranking for RCTs with weak design, as systematic reviews of RCTs of wound care consistently demonstrate that RCTs are poorly designed.^1^, ^3^, ^19^, ^32^

The clinical implication of our findings confirming uncertainty of outcomes in VLU RCTs is that the evidence base, which drives clinical practice guidelines, needs considerable improvement. While VLU guidelines universally promote high compression therapy for VLU management, beyond that recommendation, guidelines are marked by notable heterogeneity, lack of standardized definitions and criteria, conflicting recommendations, and poor applicability and uptake in clinical practice.^33^, ^34^, ^35^ Thus, there remains a great need to improve EBM in venous wound care, and to do so, better RCT design and conduct is essential.

### 4.3 Trial design

In wound care, robust RCT design is sometimes not possible, depending on the intervention or the condition studied. For example, in trials involving less common conditions (such as presence of an open abdomen in persons with a stoma) attaining the desired target can be problematic, while effective blinding can be hard to achieve in trials involving hyperbaric oxygen or negative pressure wound therapy, despite innovative approaches.^1^, ^36^, ^37^, ^38^ Ultimately, blinding in wound care RCTs may be best addressed by a third party blinded assessor,^39^ but our analysis revealed only a little over a quarter of studies had a blinded assessor. In many other areas of medicine, including plastic surgery, there are similar EBM issues to wound care and difficulties exist in designing trials with appropriate randomization and blinding.^3^, ^18^ A review of 72 RCTs published in the *Journal of Bone and Joint Surgery* from 1988 to 2000 found that 60% of those trials were of low quality due to inappropriate randomization, blinding, and nebulous exclusion criteria.^18^, ^40^ Researchers have to be more creative in how they tackle these issues in their trial design; for example, could inactive bioresorbable materials be made to approximate the appearance of any of the numerous cellular and/or tissue‐based products (CTPs) currently being used in wound care so that the current subject blinding issue is ameliorated? While this seems an outlandish suggestion (it would have to be demonstrated that any such material did not affect wound healing and might also need FDA approval) an industrial consortium approach in which all CTP manufacturers contribute could explore its feasibility. Finally, better use of adaptive designs or even hybrid designs would allow for more identification of more responsive subjects or more efficient determination of safety/efficacy, as well as better generalizability of outcomes with attendant lower uncertainty.^39^, ^41^ This last point refers to the inclusion of real‐world data in trial design and generation of real‐world evidence which can be patient‐centric and better reflect the real‐world clinical setting, by capturing patient preferences, perspectives, and harms versus treatment benefits that are not effectively captured by the traditional RCT design.^39^ This can be achieved via a pragmatic, large, simple trial design, which enrolls a broader patient population than the typical controlled trial and target clinically meaningful outcomes that are more relevant and applicable to the real‐world clinical setting.^39^, ^42^

### 4.4 Trial financial resources: problems and solutions

We recognize that sponsors of wound care clinical trials in general, including those cited in our study, often do not have the deep financial pockets of most pharmaceutical and biologics manufacturers. This is especially true of medical devices being used to treatment of VLUs, where such devices are often low tech and relatively cheap. This means that there are significant restrictions on the financial resources that companies are able to put into RCTs. Further, if devices follow the 510(k) approval pathway in the US, there is no strict requirement to provide relative efficacy data in postmarket trials, which often leads to sample sizes that are not necessarily related to the effect size of the device but rather a sample size that fits the resources available for the trial. Clearly, this has the effect of increasing the chance of a type II error and can also lead to non‐coverage by payers. We strongly recommend that authors spend a little time conducting sample size scenarios even when working with resource‐limited trials. For example, in this review, the 2011 Brizzio et al trial (No. 11) had a small sample size calculated of 52 subjects who were allocated to two different groups. Nonetheless, the power calculation was adequate and based on a previously determined effect of 0.39 regarding healing with a power of 0.8 at a two‐sided *p* <0.05.Furthermore, working through different adaptive designs, developing best practice statistical analysis plans, and thoroughly documenting these processes, particularly in trial publications, would go a long way towards better trial evaluation by peers and perhaps provide more realistic expectations of the trial before subject recruitment starts. This is the proverbial ‘an ounce of prevention is worth a pound of cure’.

Then, there are the problems of subject blinding. While it is often not practicable to make sham devices, especially for CTPs, it is encouraging to see that some manufacturers are becoming more creative in recent times. Among the articles selected in this review, there were several successful blinding examples using creative sham devices. Gupta et al (No. 46) evaluated the effect of low photon energy therapy versus a placebo therapy that used the same device, which applied light of the same colour to the VLUs (Supporting Information S1). Jünger et al (No. 58) evaluated electrical simulation versus a placebo therapy that used the same pulse generator emitting electrodes but with a nonconductive power cable (Supporting Information S1). Similar shams can be developed for laser therapy and ultrasound therapy. It certainly costs very little to brainstorm the issue rather than dismissing it out of hand. In terms of blinding the clinical assessors, while one researcher could act as a blind assessor, this requires two people to treat and assess the patient, which is not practical in many clinical situations, although is recommended for *trial* situations. Gould and Li recommend that wound care trials implement a standardized wound assessment methodology that tackles blinding, by using a blinded, on‐site assessor, who is not the treating clinician, *and* a blinded, remote adjudication panel of two to three wound care experts.^43^ Some products, such as CTPs, leave telltale marks in the wound area, which immediately inform an experienced assessor that a subject was treated with the intervention. This kind of problem can automatically invalidate blind assessment and is probably the most challenging aspect to assessing VLU treatment, but the use of artificial intelligence, such as computerized planimetry, and remote assessors to assess wounds could overcome these limitations.^43^

Clearly this is not an ideal world, but one that many researchers still have to inhabit. Nevertheless, spending more time to develop new avenues to solving old problems before the trial starts, rather than ignoring them, is likely to pay off even under financial constraints.

### 4.5 Study strengths and limitations

Rather than select a body of studies to examine a given treatment approach for our systematic review, we chose an existing review that focused on a particular assessment—study generalizability to broader populations—so we could add our results to visualize a bigger picture for one very common wound type. Consequently, our secondary analysis inherits some of the same limitations described by Gethin et al.^19^ We did not perform a new, updated search of trials from June 2018 onward. They only used English‐language articles that they could access freely, so their initial search may not have been the most comprehensive, and there is some publication bias acknowledged. Further, we did not individually assess the risk of bias for each trial; our study design was based on assessing the overall risk of bias of these studies, including the newer versus older articles. We did not perform a comprehensive analysis of every factor that could potentially influence bias and uncertainty of outcomes in RCTs; for example, publication bias and consistency of treatment effects were not analysed in this review. However, allocation concealment, blinding, power analysis, attrition rates, primary outcome measures, the inclusion of an ITT analysis, and multiplicity adjustment of secondary endpoints are all major factors influencing uncertainty of outcomes and external validity that were not considered in the systematic review by Gethin et al.^1^, ^14^, ^17^, ^18^, ^19^ Finally, given our bundling of unclear with negative assessment categories, we recognize that in some instances, our results may be seen as too conservative. Nevertheless, the large dataset compiled from 142 VLU RCTs is a major strength of our analysis, and the results of our study demonstrate that more critical analysis of the uncertainty of outcomes in wound care is needed for other wound types and outcomes.

## 5 CONCLUSIONS

VLU RCTs have high bias and poor uncertainty of outcomes incurred by lack of blinding and allocation concealment, insufficient statistical power associated with outcomes, and lack of multiplicity adjustment. Newer studies tend to very weakly demonstrate improved certainty of outcomes compared to older studies. Greater attention to the uncertainty of outcomes and trial design and conduct is needed to improve the evidence base in wound care.

## CONFLICT OF INTEREST

Kristen A. Eckert was a paid consultant of Strategic Solutions to this study; Marissa J. Carter: none to declare.

**ABBREVIATIONS**
EBM: evidence‐based medicine
ITT: intention‐to‐treat
RCT: randomized controlled trial
SD: standard deviation
VLU: venous leg ulcer

## Supporting information


**Appendix** S1: Supporting Information
