# Supplementary material for: Assessing the uncertainty of treatment outcomes in a previous systematic review of venous leg ulcer randomized controlled trials: Additional secondary analysis
Source: Wound Repair Regen. 2021 Feb 8;29(2):327–34. doi: 10.1111/wrr.12897 (PMC7986240; doi:10.1111/wrr.12897)
Supplement: Supplementary file 1 — Appendix S1: Supporting Information [file WRR-29-327-s001.pdf]

Supplemental 1: List of Venous Leg Ulcer Randomized Controlled Trials Evaluated for External Validity and Their Study Characteristics

1. Alvarez OM, Patel M, Booker J, Markowitz L. Effectiveness of a biocellulose wound dressing for the treatment of chronic venous leg ulcers: Results of a single center randomised study involving 24 patients. *Wounds.* 2004;16(7):224-233. **EXCLUDED FROM REVIEW, AS THIS IS THE SAME STUDY AS NO. 2 AND PRESENTS A DATA SUBSET ALREADY PUBLISHED IN NO.2.**

Study Groups: Group 1: Bio-cellulose dressing (BWD) + Standard of Care (SOC); Group 2: Nonadherent pertrolatum emulsion impregnated cellulose acetate gauze + SOC

Statistical results:

- ITT analysis done for primary endpoint(s): N
- **Primary endpoints:** Due to heavy censoring (missing data), it was not possible to compare survival curves for time to 75% granulation
  - Time to 75% granulation was not significant between groups (p = NS)
- Time to 50% reepithelialization: 57 days in Group 1 vs 85 days in Group 2, c2[1] = 2.210, p = .141.
- No marked differences between groups in leg edema, wound exudates, odor, maceration, pain upon dressing removal, or recurrence, p = NS

Power calculation:

- Was a power calculation for the primary endpoint reported, and if so, was it appropriate? N

Adjustment for multiplicity of statistical testing:

- Was an adjustment reported if there were more than 1 secondary endpoints statistically tested? N

Blinding:

- Were patients blinded from treatment? N
- Were assessing investigators blinded from patient treatment? N

Allocation concealment:

- Was allocation concealment carried out? N

Lost to follow-up/discontinued/withdrawn:

- 2/24 (8.3%) total
  - 2/12 (16.7%) withdrawn from Group 1
  - 0/12 (0%) withdrawn from Group 2

2. Alvarez OM, Phillips TJ, Menzoian JO, Patel M, Andriessen A. An RCT to compare a bio-cellulose wound dressing with a non-adherent dressing in VLUs. *J Wound Care*. 2012;21(9):448-453.

Study Groups: Group 1: Bio-cellulose dressing (BWD) + SOC; Group 2: SOC

Statistical results:

- ITT analysis done for primary endpoint(s): N
- **Primary endpoint:** Debridement efficacy: 15 VLUS (84%) in Group 1 achieved a 75%-100% clean, granulating wound bed vs 4 (26%) in Group 2 (p <.0001)

For Peer Review

- Mean PAR was 83% greater for Group 1 at week 6 (44% vs 24%) and 37% larger at Week 12 (74% vs 54%; p = .32)
- Significantly more patients in Group 1 reported no or mild pain at week 7 (p <.05)
- Effective exudate management reported in 42% of VLUs in the Group 1 vs 22% in Group 2 (p = .091)
- Group 1 had cleaner and healthier looking ulcer bed (p = .005)
- Investigators preferred BWD to SOC (7:1, p <.0001)

Power calculation:

- Was a power calculation for the primary endpoint reported, and if so, was it appropriate? Y, N

Adjustment for multiplicity of statistical testing:

- Was an adjustment reported if there were more than 1 secondary endpoints statistically tested? N

Blinding:

- Were patients blinded from treatment? N
- Were assessing investigators blinded from patient treatment? N

Allocation concealment:

- Was allocation concealment carried out? N

Lost to follow-up/discontinued/withdrawn:

- 15/48 (31.2%) total
    - 7/25 (28%) withdrawn from Group 1
    - 8/23 (33%) withdrawn from Group 2

3. Andersen KE, Franken CPM, Gad P, et al. A randomized controlled trial to compare the effectiveness of two foam dressings in the management of lower leg ulcers. *Ostomy Wound Manage*. 2002;48(8):34-41.

Study Groups: Group 1: nonadhesive polyurethane foam dressing; Group 2: hydrocellular dressing

Statistical results:

- ITT analysis done for primary endpoint(s): N
- **Primary endpoints are not identified.**
- Mean (SD) time to healing: 5.2 weeks (1.9) in Group 1 vs 5 weeks (1.7) in Group 2, p = NS
- Decrease in ulcer size from baseline to final evaluation: 5.19 cm^2 to 2.01 cm^2 in Group 1 (p = .005) and 7.23 cm^2 to 3.46 cm^2 in Group 2 (p <.0005)
    - Significant decrease in relative ulcer size also noted in both groups (p <.0005)
- Mean (SD) time to complete red healthy granulation tissue was 2.7 weeks (2.5) in Group 1 and 3.2 weeks (2.2) in Group 2, p = NS
- Dressing absorbency was rated as excellent in 76% (124/163) of dressing changes in Group 1 vs 7% (12/170) in Group 2, p <.0005
- Leakage of exudate was observed in 64% (198/309) of weekly assessments in Group 2 vs 48% (172/355) in Group 1, p <.0005
- Use of secondary absorbent dressing done in 72% (188/263) of Group 2 vs 43% (132/308) of Group 1, p < .0005

For Peer Review

- No. of dressing changes/week was 2.14 in Group 1 vs 3.34 in Group 2, p <.0005
- Special treatment of surrounding skin needed for 72% (212/296) of dressing changes in Group 2 vs 54% (191/352) in Group 1, p <.0005
- No significant differences were observed between groups in patient ratings of comfort during treatment, p = NS

Power calculation:

- Was a power calculation for the primary endpoint reported, and if so, was it appropriate? N

Adjustment for multiplicity of statistical testing:

- Was an adjustment reported if there were more than 1 secondary endpoints statistically tested? N

Blinding:

- Were patients blinded from treatment? N
- Were assessing investigators blinded from patient treatment? N

Allocation concealment:

- Was allocation concealment carried out? N

Lost to follow-up/discontinued/withdrawn:

- 19/118 (16.1%) total

For Peer Review

4. Arosio E, Ferrari G, Santoro L, Gianese F, Coccheri S. A placebo-controlled, double-blind study of mesoglycan in the treatment of chronic venous ulcers. *Eur j Vasc Endovasc Surg*. 2001;22(4):365-372.

Study Groups: Group 1: Mesoglycan; Group 2: Placebo (matching saline solution ampoules and excipient capsules)

Statistical results:

- ITT analysis done for primary endpoint(s): Y.
- **Primary:** Time to heal 25%, 50% (median), and 75% of patients was 36, 64, and 90 days in Group 1 vs 42, 70, and 136 days in Group 2, while the estimated healing rate at end of observation was 97% vs 82%, respectively (p <.05)
    - Relative risk for healing was 1.48 (95% CI: 1.05-2.09)
    - After adjusting for baseline area, the difference was still significant (p <.05) and the relative risk for healing in Group 1 was 1.43 (95% CI: 1.01-2.04)
    - No significant deviation from proportionality was detected in the relative risk for healing over time, p = NS
    - Treatment by center interaction was not significant, p = NS
- When ulcer healing events were attributed to the fixed dates scheduled per protocol, the difference between groups was still significant, p = .02; p = .03 after adjusting for baseline area
- Overall time-course for ulcer-associated pain scores did not differ significantly (p = .27)
- Initial decrease in pain from a mean (SD) baseline score of 4.4 (3.0) to 1.7 (2.4) at Week 3 in Group 1 vs 4.2 (2.8) to 2.1 (2.4) in Group 2, p <.01
- SF-36 differences between healed vs nonhealed patients
    - Physical functioning: 9 vs 8, p = NS

- Role-physical limitation: 19 vs 6, p <.05
- Bodily pain: 29 vs 7, p <.05
- General health: 6.5 vs -4.5, p < .05
- Vitality: 9 vs -1, p < .05
- Social functioning: 8.5 vs 0.5, p < .05
- Role limitation due to emotional problems: 18 vs -2, p <.05
- Mental health: 9.75 vs 0.1, p <.05

Power calculation:
- Was a power calculation for the primary endpoint reported, and if so, was it appropriate? Y, Y

Adjustment for multiplicity of statistical testing:
- Was an adjustment reported if there were more than 1 secondary endpoints statistically tested? N

Blinding:
- Were patients blinded from treatment? Y
- Were assessing investigators blinded from patient treatment? Y

Allocation concealment:
- Was allocation concealment carried out? Y

Lost to follow-up/discontinued/withdrawn:
- 15/183 (8.2%) total
  - 6/92 (6.5%) withdrawn from Group 1
  - 9/91 (9.9%) withdrawn from Group 2

5. Ashby RL, Gabe R, Ali S, et al. Clinical and cost-effectiveness of compression hosiery versus compression bandages in treatment of venous leg ulcers (Venous leg Ulcer Study IV, VenUS IV): a randomised controlled trial. *Lancet.* 2014;383(9920):871-879.

Study Groups: Group 1: 2-layer hosiery; Group 2: 4-layer bandage

Statistical results:
- ITT analysis done for primary endpoint(s): Y
- **Primary endpoint:** Median time to healing: 99 days (95% CI: 84-126) for Group 1 vs 98 days (95% CI: 85-112) for Group 1 and proportion of ulcer healing was 71% (163/230) for Group 1 vs 70% (157/223) for Group 2
  - After adjustment for ulcer area, duration, and mobility with shared center frailty effects, the HR was 0.99 (95% CI: 0.79-1.25, p = .96)
- Mean mental component summary score by 12 months: 49 (95% CI: 47-51.5) vs 51 (95% CI: 48-52.5), p = NS
- For physical component summary score, a transient effect occurred at 3 months, with a significant interaction of treatment with time (p = .018) in favor of Group 1 after adjustment for ulcer area, duration, participant mobility, center, and timepoint
- 38% (88/230) of Group 1 vs 28% (62/224) in Group changed to a nontrial treatment before their ulcers healed, p = .02
  - Higher rate of treatment change in Group 1 (HR: 1.59, 95% CI: 1.41-2.21, p = .005), in older patients (HR: 1.02, 95% CI: 1.00-1.03, p = .003), and in those with

For Peer Review

at least 1 nonserious AE (HR: 1.75, 95% CI: 1.18-2.59), p = .005

- 67% (154/230) in Group 1 vs 58% (130/224) in Group 2 had at least one nonserious AE, p = .05
    - No significant group difference in the no. of nonserious AEs (relative risk 1.12, 95% CI: 0.95-1.32)
- Ulcer recurrence rate: 14% (24/167) in Group 1 vs 23% (41/176) in Group 2, HR: 0.56, 95% CI: 0.33, p = .026)

Power calculation:

- Was a power calculation for the primary endpoint reported, and if so, was it appropriate? Y, Y

Adjustment for multiplicity of statistical testing:

- Was an adjustment reported if there were more than 1 secondary endpoints statistically tested? N

Blinding:

- Were patients blinded from treatment? N
- Were assessing investigators blinded from patient treatment? N

Allocation concealment:

- Was allocation concealment carried out? N

Lost to follow-up/discontinued/withdrawn:

- 160/457 (35%) total
    - 90/230 (39.1%) withdrawn from Group 1
    - 71/227 (31.3%) withdrawn from Group 2

6. Barwell JR, Davies CE, Deacon J, et al. Comparison of surgery and compression with compression alone in chronic venous ulceration (ESCHAR study): randomised controlled trial. *Lancet.* 2004;363(9424):1854-1859.

Study Groups: Group 1: compression + surgery; Group 2: compression alone

Statistical results:

- ITT analysis done for primary endpoint(s): Y.
- **Primary endpoint:** 24-week ulcer healing and 12 month ulcer recurrence rates
- Percentage (n) of 24-week ulcer healing rates by reflux pattern:
    - Isolated superficial: 65 (102) in Group 1 vs 66 (112) in Group 2, p = .46
    - Superficial and total deep: number too small to give accurate healing rates (20) in Group 1 vs (29) in Group 2,
    - Superficial and segmental deep: 56 (37) in Group 1 vs 57 (39) in Group 2, p = .62
- 341 patients had open ulcers at time of assessment and were analyzed with respect to ulcer healing rate: median follow-up was 12 weeks (IQR 5 – 33). 269 (79%) study legs healed during the period of study; 128/156 (82%) in Group 1 vs 141/185 (76%) in Group 2,
- 24 week ulcer healing rate: 65% in both arms (hazard 0.84 (95%CI 0.77 to 1.24), p = 0.85
- 12 months ulcer recurrence rate, %: 12 in Group 1 vs 28 in Group 2, hazard -2.76 (95 CI -1.78 to 4.27), p <.0001
- By Cox regression analysis, the effect of surgery on ulcer recurrence was not affected by

For Peer Review

presence of diabetes, p = 0.77 for the effect of diabetes; for the effect of surgery, p = .015

- Results on subgroup analysis showed that patients in Group 1 with isolated superficial reflux and with mixed superficial and segmental deep reflux had lower 12-month recurrence rates, %: 12 in group 1 vs 26 in Group 2, p <.0001 and 9 in Group 1 vs 25 in Group 2, p = .04, respectively
- No significant difference in recurrence rate was seen in patients with mixed superficial and total deep reflux, %: 19 in Group 1 vs 31 in Group 2, p = .42

Power calculation:

- Was a power calculation for the primary endpoint reported, and if so, was it appropriate? Y, Y

Adjustment for multiplicity of statistical testing:

- Was an adjustment reported if there were more than 1 secondary endpoints statistically tested? N

Blinding:

- Were patients blinded from treatment? N
- Were assessing investigators blinded from patient treatment? N

Allocation concealment:

- Was allocation concealment carried out? N

Lost to follow-up/discontinued/withdrawn:

- 40/500 (8%) total
    - 18/242 (7.4%) withdrawn from Group 1
    - 22/258 (8.2%) withdrawn from Group 2

7. Beckert S, Warnecke J, Zelenkova H, et al. Efficacy of topical pale sulfonated shale oil in the treatment of venous leg ulcers: a randomized, controlled, multicenter study. *J Vasc Surg.* 2006;43(1):94-100.

Study Groups: Group 1: Pale sulfonated shale oils (PSSO); Group 2: nonionic polyoxyethylene-polyoxypropylene block copolymer

Statistical results:

- ITT analysis done for primary endpoint(s): Y
- **Primary endpoint:** Cumulative relative reduction in ulcer area, cm^2 (SD): -4391 (4748.7) in Group 1 vs -231.9 (6283.6) in Group 2, p <.0001
    - Beginning at 6 weeks, the relative reduction in wound area was significantly increased in Group 1 vs Group 2: -47.4 (28.4) vs -23.8 (42.2), p < .001
    - Ulcer size cm^2 (SD): 15 (15.9) to 6.2 (12.9) in Group 1 vs 11.4 (14.5) to 10.8 (15.7) in Group 2, p = .0005
- **Primary endpoint:** There were no significant groups differences in complete granulation (p = .161) and epithelialization of by the end of 20 weeks (p = .177)
    - 53.2% (33) in Group 1 had complete granulation and 33.9% had complete epithelialization
    - 36.8% (21) in Group 1 had complete granulation and 22.8% (13) had complete epithelialization

For Peer Review

- There was no significant group difference in pain: baseline: 4.6 (2.9) vs 5.3 (3.2); study end: 1.8 (2.3) vs 2.6 (2.4), p = NS
- After 20 weeks, physicians and patients thought Group 1 treatment to be significantly more effective, p = .001 and p = .001)
- No significant group differences in AEs: 12.2% (9) in Group 1 vs 11.1% (7) in Group 2, p = NS
- No significant group differences in tolerability among physicians (p = .077) and patients (p = .154)

Power calculation:

- Was a power calculation for the primary endpoint reported, and if so, was it appropriate? Y, Y

Adjustment for multiplicity of statistical testing:

- Was an adjustment reported if there were more than 1 secondary endpoints statistically tested? 0

Blinding:

- Were patients blinded from treatment? N
- Were assessing investigators blinded from patient treatment? Y

Allocation concealment:

- Was allocation concealment carried out? Y

Lost to follow-up/discontinued/withdrawn:

- 18/119 (15.1%) total
  - 9/62 (14.5%) withdrawn from Group 1
  - 9/57 (15.8%) withdrawn from Group 2

8. Bianchi C, Cazzell S, Vayser D, Reyzelman AM, Dosluoglu H, Tovmassian G. A multicentre randomised controlled trial evaluating the efficacy of dehydrated human amnion/chorion membrane (EpiFix((R))) allograft for the treatment of venous leg ulcers. *Int Wound J.* 2018;15(1):114-122.

Study Groups: Group 1: Dehydrated human amnion/chorion membrane; Group 2: SOC

Statistical results:

- ITT analysis done for primary endpoint(s): N.
- **Primary endpoint:** Time to heal over 12 weeks: 31/52 (60%) in Group 1 healed vs 20/57 (35%) in Group 2, p = .0128
  - At 16 weeks: 37/52 (71%) healed in Group 1 vs 25/57 (44%) in Group 2, p =.00625
- After adjusting for baseline wound size, week 12 adjusted mean size was 2.82 cm^2 in Group 1 vs 4.81 cm^2 in Group 2, p = .0435
  - At 16 weeks: 2.28 cm^2 in Group 1 vs 4.90 cm^2 in Group 2, p = .0098
- Kaplan-Meier time to heal demonstrated superior wound-healing trajectory for Group 1 vs Group 2, with log-rank test of equality of the healing function producing a chi-square test statistic of 6.4597 with p = .011.
- Group 1 wounds were 2.26 more times likely to heal within 12 weeks than Group 2, with the following covariate healing associations from Cox Regression Model 1:

For Peer Review

- Treatment – Group 1: p = .01, HR = 2.71, 95% CI: 1.26-5.87
  - Age (years): p = .99, HR = 1.00, 95% CI: 0.97-1.03
  - BMI (Log): p = .98, HR = 1.02, 95% CI: 0.27-3.90
- Baseline wound size (Log): p = .00, HR = 0.53, 95% CI: 0.35-0.81
- Ulcer duration (Log): p = .03, HR = 0.70, 95% CI: 0.51-0.97
  - Race – White: p = .52, HR = 1.36, 95% CI: 0.53-3.49
  - Ethnicity – Hispanic: p = .84, HR = 1.10, 95% CI: 0.44-2.75
  - Sex – Male: p = .88, HR = 0.95, 95% CI: 0.46-1.96
- Hypertension – Yes: p = .05, HR = 2.51, 95% CI: 1.00-6.31
  - Diabetes – Yes: p = .32, HR = 1.57, 95% CI: 0.65-3.82
  - Smoking – Yes: p = .40, HR = 1.46, 95% CI: 0.60-3.51
  - Alcohol – Yes: p = .38, HR = 0.71, 95% CI: 0.33-1.53
  - VLU side – Left: p = .01, HR = 1.89, 95% CI: 0.89-4.03
  - VLU position – Malleolus: p = .58, HR = 1.54, 95% CI: 0.33-7.31
  - VLU position – Low Gaiter: p = .40, HR = 1.84, 95% CI: 0.45-7.56
  - VLU position – Lateral: p = .83, HR = 1.21, 95% CI: 0.22-6.77
  - VLU location – Anterior: p = .86, HR = 1.19, 95% CI: 0.17-8.14
  - VLU location – Medial: p = .90, HR = 1.11, 95% CI: 0.22-5.66
  - Hx of recurrent ulcers – Yes: p = .68, HR = 0.85, 95% CI: 0.39-1.84

- Cox regression Model 2:
  - Treatment – Group 1: p = .01, HR = 2.26, 95% CI: 1.25-4.10
  - Baseline wound size (Log): p = .00, HR = 0.53, 95% CI: 0.38-0.74
  - Ulcer duration (Log): p = .02, HR = 0.71, 95% CI: 0.53-0.95
  - Hypertension – Yes: p = .05, HR = 2.12, 95% CI: 0.99-4.53
- AEs: 35 (51.5%) in Group 1 vs 30 (44.1%) in Group 2, p = .171
  - Procedure-related: 0 in both groups, p = 1.0000
  - Product-related: 0 in both groups, p = 1.0000
  - Procedure- and product-related: 0 in both groups, p = 1.0000
  - Severe: 9 in Group 1 vs 4 in Group 2, p = .140
  - Cardiovascular: 7 (13.5%) in Group 1 vs 4 (7.0%) in Group 2, p = .3455
  - Digestive: 1 (1.9%) vs 0 (0%), p = .4771
  - Integumentary – non-target ulcer: 14 (26.9%) vs 15 (26.3%), p = 1.0000
  - Integumentary – target ulcer: 4 (7.7%) vs 3 (5.3%), p = .7070
  - Lymphatic: 6 (11.5%) vs 3 (5.3%), p = .3052
  - Muscular: 2 (3.8%) vs 2 (3.5%), p = 1.0000
  - Nervous system: 1 (1.9%) vs 1 (1.8%), p = 1.0000
  - Renal: 0 (0.0%) vs 2 (3.5%), p = .4964

Power calculation:

- Was a power calculation for the primary endpoint reported, and if so, was it appropriate? Y, Y

Adjustment for multiplicity of statistical testing:

- Was an adjustment reported if there were more than 1 secondary endpoints statistically tested? N

Blinding:

For Peer Review

- Were patients blinded from treatment? N
- Were assessing investigators blinded from patient treatment? Y

Allocation concealment:

- Was allocation concealment carried out? N

Lost to follow-up/discontinued/withdrawn:

- 19/128 (14.8%) total
  - 12/64 (18.8%) withdrawn from Group 1
  - 7/64 (10.9%) withdrawn from Group 2

9. Blecken SR, Villavicencio JL, Kao TC. Comparison of elastic versus nonelastic compression in bilateral venous ulcers: a randomized trial. *J Vascular Surg.* 2005;42(6):1150-1155.

Study Groups: Group 1: nonelastic compression (n = 12 legs); Group 2: elastic compression (n = 12 legs), 12 patients total

For Peer Review

Statistical results:

- ITT analysis done for primary endpoint(s): Y
- **Primary endpoints:** Ulcer reduction, cm^2/week: Group 1 HR: 0.56; Group 2 HR: 1; 95% CI: 0.33-0.96; p = .0173
- Limb circumference reduction rate (cm/wk): Group 1 HR: 2.36; Group 2 HR: 1; 95% CI: 0.30-18.52; p = .3580
- Mean (SE) initial ulcer area (cm2): 48.98 (14.13) in Group 1 vs 50.08 (18.30) in Group 2, 95% CI: -27.25 to 25.07, p = .9285
- Mean (SE) ulcer area reduction rate (cm^2/week): 2.93 (0.60) vs 2.30 (0.70), 95% CI: 0.05 to 1.21, p = .0369
- Mean (SE) initial limb circumference (cm): 35.59 (2.42) vs 35.59 (2.56), 95% CI: -2.17 to 2.17, p = 1.0000
- Mean (SE) limb circumference reduction rate (cm/week): 0.32 (0.14) vs 0.15 (0.18), 95% CI: -0.08 to 0.42, p = .1606
- Mean (SE) patient satisfaction score: 2.92 (0.08) vs 2.58 (0.15), 95% CI: -0.08 to 0.75, p = 1.039

Power calculation:

- Was a power calculation for the primary endpoint reported, and if so, was it appropriate? N

Adjustment for multiplicity of statistical testing:

- Was an adjustment reported if there were more than 1 secondary endpoints statistically tested? N

Blinding:

- Were patients blinded from treatment? N
- Were assessing investigators blinded from patient treatment? N

Allocation concealment:

- Was allocation concealment carried out? N

Lost to follow-up/discontinued/withdrawn: Not reported, but assume that none were lost to follow-up

10. Brehmer F, Haenssle HA, Daeschlein G, et al. Alleviation of chronic venous leg ulcers with a hand-held dielectric barrier discharge plasma generator (PlasmaDerm(®)) VU-2010): results of a monocentric, two-armed, open, prospective, randomized and controlled trial (NCT01415622). J Eur Acad Dermatol Venereol. 2015;29(1):148-155.

Study Groups: Group 1: plasma treatment; Group 2: SOC

Statistical results:

- ITT analysis done for primary endpoint(s): N
- **Primary endpoints:** No. of SAEs: 1 SAE in each group, p = .77
  - All 7 patients in Group 2 and 6 in Group 1 had at least 1 AE, p = 1.0
- There was a significant reduction in bacterial load in ulcers in Group 1 immediately after application as demonstrated by mean (SD) enlargement of 88.2 (51.09) mm^2 of bacteria-free area in the plates, p = .0313
  - By study end, bacterial strain types did not change nor differ between groups, p = NS
- There was no significant difference regarding ulcer size reduction (p = .42) between groups
- At 4-week follow-up, 2/5 in Group 1 and 3/6 in Group 2 showed increase in ulcer size, p = 1.0
- Pain did not differ between groups: p = .5046 during treatments, 0.4753 between treatments
- Plasma applicability was not rated inferior to SOC by patients (p = .94) and by physicians (p = 1.0)
- Plasma therapy was more time consuming by week 8, p = .22
- Patient satisfaction with healing process in favor of plasma, p = .23
- Physicians would recommend (p = .06) and repeat (p = .08) plasma therapy

Power calculation:

- Was a power calculation for the primary endpoint reported, and if so, was it appropriate? N

Adjustment for multiplicity of statistical testing:

- Was an adjustment reported if there were more than 1 secondary endpoints statistically tested? N

Blinding:

- Were patients blinded from treatment? N
- Were assessing investigators blinded from patient treatment? N

Allocation concealment:

- Was allocation concealment carried out? N

Lost to follow-up/discontinued/withdrawn:

- 3/15 (20.0%) total
  - 1 patient randomized (allocation not reported) and not treated
  - 1/7 (14.3%) withdrawn from Group 1
  - 1/7 (14.3%) withdrawn from Group 2

11. Brizzio E, Amsler F, Lun B, Blattler W. Comparison of low-strength compression stockings

For Peer Review

with bandages for the treatment of recalcitrant venous ulcers. *J Vasc Surg.* 2010;51(2):410-416.

Study Groups: Group 1: Stockings; Group 2: bandages

Statistical results:

- ITT analysis done for primary endpoint(s): N
- **Primary endpoint:** Proportion of healing within 90 days: 10 (36%) in Group 1 vs 13 (48%) in Group 2, p = .350
- Mean (SD) time to healing within 90 days: 46 (16) vs 48 (20), p = .750
- Mean (SD) size of ulcers not healed at 90 days (cm^2): 7.4 (9.8) vs 10.4 (14.8), p = .501
- No. of ulcers healed between 90 and 180 days: 4 (22%) vs 5 (36%), p = .400
- Cumulative no. of ulcers healed within 180 days: 14 (50%) vs 18 (67%), p = .210
- Mean (SD) cumulative time to healing within 180 days: 68 (40) vs 69 (39), p = .942
- Mean (SD) size of ulcers not healed at 180 days (cm^2): 10.0 (15.1) vs 16.1 (19.1), p = .406
- Nonhealing at 180 days was associated with time of presence (ln; r = 61, p <.001), ulcer size (ln; r = 0.52, p <.001), extent of wound secretion (r = 0.48, p <.001)
- Nonhealing at 90 days was associated with older age (r = 0.31, p <.05) and higher BMI (r = 0.30, p <.05)
- Mean (SD) pain in Group 1 decreased from 44.2 (33.1) to 17.7 (18.8) within first week (p <.001) and in Group 2 decreased from 45.8 (26.4) to 11.1 (15.6) (p <.01)
- Mean (SD) Chronic Venous Sufficiency Quality of Life (CIVIQ,
- 20-100, 20 = best and 100 = worst)
    - Before treatment: 53 (18) in Group 1 vs 53 (15) in Group 2, p = .999
    - At 35 days: 44 (16) in Group 1 vs 44 (19) in Group 2, p = .944
    - At 90 days in patients with healed ulcers: 44 (18) vs 45 (17), p = .825
    - At last assessment in patients with healed ulcers: 39 (18) vs 30 (17), p = .109
- Influence of sex on:
    - Healing at 90 days: p = .550
    - Healing at 180 days: p =.616
    - Time to healing: p = .242
    - CIVIQ at 35 days: p = .657
    - CIVIQ at last assessment: p = .753
- Influence of age on:
    - Healing at 90 days: p = .133
    - Healing at 180 days: OR = 0.91 p =. 028
    - Time to healing: p = .818
    - CIVIQ at 35 days: p = .880
    - CIVIQ at last assessment: p = .979
- Influence of BMI on:
    - Healing at 90 days: OR = 1.16, p = .032
    - Healing at 180 days: p =. 297
    - Time to healing: OR = 1.05, p = .057
    - CIVIQ at 35 days: p = .398
    - CIVIQ at last assessment: p = .980

For Peer Review

- Influence of initial ulcer size (Log) on:
    - Healing at 90 days: OR = 0.15, p = .001
    - Healing at 180 days: OR = 0.33, p = .033
    - Time to healing: OR = 0.58, p = .084
    - CIVIQ at 35 days: p = .880
    - CIVIQ at last assessment: p = .494
- Influence of presence of ulcer (Log) on:
    - Healing at 90 days: p = .229
    - Healing at 180 days: OR = 0.26, p = .002
    - Time to healing: p = .249
    - CIVIQ at 35 days: p = .532
    - CIVIQ at last assessment: p = .673
- Influence of recurrent ulcer on:
    - Healing at 90 days: p = .743
    - Healing at 180 days: OR = 7.16, p = .044
    - Time to healing: p = .695
    - CIVIQ at 35 days: p = .396
    - CIVIQ at last assessment: p = .665
- Influence of deep venous reflux on:
    - Healing at 90 days: p = .129
    - Healing at 180 days: p = .691
    - Time to healing: p = .634
    - CIVIQ at 35 days: p = .835
    - CIVIQ at last assessment: p = .309
- Influence of edema score on:
    - Healing at 90 days: p = .129
    - Healing at 180 days: p = .449
    - Time to healing: p = .219
    - CIVIQ at 35 days: p = .767
    - CIVIQ at last assessment: p = .517
- Influence of compression type on:
    - Healing at 90 days: OR = 2.11, p = .296
    - Healing at 180 days: OR = 7.42, p = .067
    - Time to healing: OR = 1.04, p = .907
    - CIVIQ at 35 days: OR = -0.01, p = .944
    - CIVIQ at last assessment: -0.01, p = .408
- Influence of compression type alone on:
    - Healing at 90 days: OR = 1.67, p = .351
    - Healing at 180 days: OR = 2.00, p = .213
    - Time to healing: OR = 0.98, p = .946
    - CIVIQ at 35 days: OR = -0.01, p = .944
    - CIVIQ at last assessment: -0.01, p = .408
- CIVIQ Pain:

For Peer Review

    - Mean (SD) Initial value: 48 (20) for healed ulcers vs 50 (29) for nonhealed ulcers, p = .803
    - Mean (SD) last value: 12 (11) for healed ulcers vs 18 (19) for nonhealed ulcers, p = .174
    - Difference between initial and last values for healed ulcers: p <.001
    - Difference between initial and last values for nonhealed ulcers: p <.001
- CIVIQ Physical:
    - Mean (SD) Initial value: 38 (27) for healed ulcers vs 43 (28) for nonhealed ulcers, p = .555
    - Mean (SD) last value: 17 (19) for healed ulcers vs 38 (31) for nonhealed ulcers, p = .012
    - Difference between initial and last values for healed ulcers: p = .001
    - Difference between initial and last values for nonhealed ulcers: p = .527
- CIVIQ Social:
    - Mean (SD) Initial value: 37 (28) for healed ulcers vs 44 (28) for nonhealed ulcers, p = .404
    - Mean (SD) last value: 22 (27) for healed ulcers vs 47 (23) for nonhealed ulcers, p = .001
    - Difference between initial and last values for healed ulcers: p = .020
    - Difference between initial and last values for nonhealed ulcers: p = .624
- CIVIQ Psychic:
    - Mean (SD) Initial value: 40 (23) for healed ulcers vs 36 (24) for nonhealed ulcers, p = .487
    - Mean (SD) last value: 20 (20) for healed ulcers vs 29 (25) for nonhealed ulcers, p = .208
    - Difference between initial and last values for healed ulcers: p = .003
    - Difference between initial and last values for nonhealed ulcers: p = .139
- CIVIQ Total:
    - Mean (SD) Initial value: 41 (20) for healed ulcers vs 41 (21) for nonhealed ulcers, p = .974
    - Mean (SD) last value: 18 (15) for healed ulcers vs 31 (21) for nonhealed ulcers, p = .025
    - Difference between initial and last values for healed ulcers: p <.001
    - Difference between initial and last values for nonhealed ulcers: p = .030

Power calculation:

- Was a power calculation for the primary endpoint reported, and if so, was it appropriate? Y, Y

Adjustment for multiplicity of statistical testing:

- Was an adjustment reported if there were more than 1 secondary endpoints statistically tested? N

Blinding:

- Were patients blinded from treatment? N
- Were assessing investigators blinded from patient treatment? N

For Peer Review

Allocation concealment:
- Was allocation concealment carried out? N

Lost to follow-up/discontinued/withdrawn:
- 55/60 (91.7%) total
  - 4/32 (12.5%) withdrawn from Group 1
  - 1/28 (3.6%) withdrawn from Group 2

12. Brown A, Augustin M, Junger M, et al. Randomized standard-of-care-controlled trial of a silica gel fibre matrix in the treatment of chronic venous leg ulcers. *Eur J Dermatol.* 2014;24(2): 210-216.

Study Groups: Group 1: Placebo phototherapy using Probe One (n = 11 ulcers); Group 2: Probe 2 phototherapy (n = 14); Group 3: Control (SOC only, n = 7).

Statistical results:
- ITT analysis done for primary endpoint(s)?: Y
- **Primary endpoints not identified.**
- Mean (SD) healing rate (%) at 30 days: 20 (0.20) in Group 1, 30 (0.23) in Group 2, -10 (0.38) in Group 3
  - Difference between Group 1 and Group 3, p = NS
  - Significant difference between Group 1 and Group 2, p <.01
  - Significant difference between Group 2 and Group 3, p <.01
- Mean (SD) healing rate (%) at 60 days: 30 (0.20) in Group 1, 40 (0.25) in Group 2, 10 (0.29) in Group 3
  - Difference between Group 1 and Group 3, p = NS
  - Difference between Group 1 and Group 2, p = NS
  - Significant difference between Group 2 and Group 3, p <.05
- Mean (SD) healing rate (%) at 90 days: 40 (0.31) in Group 1, 50 (0.22) in Group 2, -40 (0.33) in Group 3
  - Significant difference between Group 1 and Group 3, p <.05
  - Significant difference between Group 2 and Group 3, p <.01
  - Significant difference between Group 2 and Group 3, p <.001

Power calculation:
- Was a power calculation for the primary endpoint reported, and if so, was it appropriate? Y, Y

Adjustment for multiplicity of statistical testing
- Was an adjustment reported if there were more than 1 secondary endpoints statistically tested? N

Blinding
- Were patients blinded from treatment? N
- Were assessing investigators blinded from patient treatment? N

Allocation concealment
- Was allocation concealment carried out? N

Lost to follow-up/discontinued/withdrawn: None reported.

For Peer Review

13. Caetano KS, Frade MA, Minatel DG, Santana LA, Enwemeka CS. Phototherapy improves healing of chronic venous ulcers. *Photomed Laser Surgery.* 2009;27(1):111-118.

Study Groups: Group 1: low-fluence placebo red light phototherapy + SOC (n = 11 ulcers); Group 2: Phototherapy + SOC (n = 14 ulcers); Group 3: SOC (n = 7 ulcers)

Statistical results:

- ITT analysis done for primary endpoint(s): Y.
- **Primary endpoints not identified**
- Mean (SD; median) ulcer healing rate, %:
    - 30 days: 20 (0.20; 20) in Group 1 vs 30 (0.23; 20) in Group 2 vs (10; 0.38; 10) in Group 3
    - 60 days: 30 (0.20; 30) vs 40 (0.25; 40) vs 10 (0.29; 0)
    - 90 days: 40 (0.31; 50) vs 50 (0.22; 50) vs 40 (0.33; 30)
    - P (G2, G3) at day 30 < .01, at day 60 <.05, at day 90 <.001
    - P (G1, G3) at day 90 <.05
    - P (G2, G1) at days 30 and 90 <.01
    - Other combinations p >.05

Power calculation:

- Was a power calculation for the primary endpoint reported, and if so, was it appropriate? N

Adjustment for multiplicity of statistical testing:

- Was an adjustment reported if there were more than 1 secondary endpoints statistically tested? N

Blinding:

- Were patients blinded from treatment? N
- Were assessing investigators blinded from patient treatment? N

Allocation concealment:

- Was allocation concealment carried out? N

Lost to follow-up/discontinued/withdrawn: None reported.

14. Campos W, Torres IO, da Silva ES, Casella IB, Puech-Leao P. A prospective randomized study comparing polidocanol foam sclerotherapy with surgical treatment of patients with primary chronic venous insufficiency and ulcer. *Ann Vascular Surg.* 2015;29(6):1128-1135.

Study Groups: Group 1: Foam sclerotherapy; Group 2: surgical treatment

Statistical results:

- ITT analysis done for primary endpoint(s): N
- **There were no primary endpoints identified.**
- Ulcer healing was achieved in 91.3% (21/23) of Group 1 vs 100% (28/28) of Group 2, p = .19
- Mean (SD) time to ulcer healing was 56.4 (39.4) days in Group 1 vs 37.1 (22.1) days in Group 2, p = .008
- Mean (SD) Venous Clinical Severity Score
    - Group 1: 12.26 (3.05) at baseline, 4.26 (3.14) at follow-up, p <.001

For Peer Review

  - Group 2: 12.5 (1.64) at baseline, 3.39 (1.57) at follow-up, p <.001
  - Follow-up scores differences between groups: p = .58
- Mean (SD) Venous Disability Score
  - Group 1: 2.00 (0.067) at baseline, 1.09 (0.85) at follow-up, p <.001
  - Group 2: 2.14 (0.71) at baseline, 1.18 (0.9) at follow-up, p <.001
  - Follow-up scores differences between groups: p = .66
- Mean (SD) Aberdeen Varicose Veins Questionnaire Score
  - Group 1: 37.72 (18.17) at baseline, 15.95 (12.09) at follow-up, no p
  - Group 2: 40.31 (5.57) at baseline, 12.30 (7.87) at follow-up, no p
  - Follow-up scores differences between groups: p = .45
- Complications occurred in 13.0% in Group 1 vs 14.2% in Group 2, p = 1.0

Power calculation:

- Was a power calculation for the primary endpoint reported, and if so, was it appropriate? N

Adjustment for multiplicity of statistical testing:

- Was an adjustment reported if there were more than 1 secondary endpoints statistically tested? N

Blinding:

- Were patients blinded from treatment? N
- Were assessing investigators blinded from patient treatment? N

Allocation concealment:

- Was allocation concealment carried out? N

Lost to follow-up/discontinued/withdrawn:

- 7/58 (12.1%) total
  - 6/29 (20.6%) withdrawn from Group 1
  - 1/28 (3.6%) withdrawn from Group 2

15. Cavalcanti LM, Pinto FCM, Oliveira GM, Lima SVC, Aguiar JLA, Lins EM. Efficacy of bacterial cellulose membrane for the treatment of lower limbs chronic varicose ulcers: a randomized and controlled trial. *Rev Col Braso Cir.* 2017;44(1):72-80.

Study Groups: Group 1: Bacterial cellulose membrane dressings (n = 14); Group 2: tryglyceride oil (n =11)

Statistical results:

- ITT analysis done for primary endpoint(s): Y
- **Primary endpoint:** Healing rate at 120 days based on mean (SD) wound size (cm^2)
  - Initial area: 54.0 (57.0) in Group 1 vs 50.0 (59.0) in Group 2
  - Day 30: 55.0 (54.0) in Group 1 vs 31.0 (26.0) in Group 2
  - Day 120: 54.0 (49.0) vs 36.0 (27.0), p = .5748
  - No significant difference between groups when wounds were grouped by mean ulcer size at any of the evaluation times (p = .7120)
  - No. of healed wounds was 2 (14.3%) in Group 1 vs 3 (27.7%)
    - Significant difference between groups at initial evaluation (p = .0096), first (p = .0096), and in the second evaluations (p = .0156)

For Peer Review

- Amount (p = .9928) and quality (p = .0357) was not significant between groups
- Pain intensity during the second evaluation was lower in Group 1 vs Group 2, p = .0357, with no significant differences at any other timepoints (p = NS)
- In Group 1, 57.1% had subcutaneous tissue loss at baseline and, after 120 days, loss of epidermis was in 83.3% vs in Group 2 with respective values of 63.6% and 62.5%, p <.0001
- At day 120, in Group 1, 25% had both granulation and epithelial tissue at the second reassessment vs 12.5% in Group 2, p = .6946

Power calculation:

- Was a power calculation for the primary endpoint reported, and if so, was it appropriate? Y, N

Adjustment for multiplicity of statistical testing:

- Was an adjustment reported if there were more than 1 secondary endpoints statistically tested? N

Blinding:

- Were patients blinded from treatment? N
- Were assessing investigators blinded from patient treatment? N

Allocation concealment:

- Was allocation concealment carried out? N

Lost to follow-up/discontinued/withdrawn: None reported.

16. Charles H, Callicot C, Mathurin D, Ballard K, Hart J. Randomised, comparative study of three primary dressings for the treatment of venous ulcers. *Br J Community Nur.* 2002;7(6 Suppl):48-54.

Study Groups: Group 1: Cutinova; Group 2: Granuflex; Group 3: Comfeel

Statistical results:

- ITT analysis done for primary endpoint(s)? 1
- **No primary endpoints defined.**
- No statistical validity among ulcer size and healing rates, suggesting that the 3 dressings equally effective (p = NS)
    - 18/31 (58%) of Group 1 ulcers healed at 12 weeks, 17/31 (55%) of Group 2 healed, and 17/29 (59%) of Group 3 healed
    - Mean PAR at 12 weeks: Group 1: 32.7%, Group 2: 16.4%, and Group 3: 17.9%
- No statistically significant difference in reported pain prevalence or severity among groups (p = NS)
    - Approximately 69% of patients reported pain at baseline vs 29% at 2 weeks and 7% at 12 weeks
        - Pain Prevalence Baseline: Group 1: 79%; Group 2: 61%; Group 3: 63%
        - Pain Prevalence Week 2: Group 1: 45.5%; Group 2: 21%; Group 3: 45%
        - Pain Prevalence Week 12: Group 1: 10%; Group 2: 3%; Group 3: 7%
    - Mean VAS pain severity: 4.1 at baseline; 1.5 at week 2; 0.5 at week 12
        - Mean VAS pain at baseline: Group 1: 4.8; Group 2: 3.8; Group 3: 3.8
        - Mean VAS pain at 2 weeks: Group 1: 2.7; Group 2: 1.1; Group 3: 1.4

For Peer Review

        - Mean VAS pain at 12 weeks: Group 1: 0.5; Group 2: 0.15; Group 3: 0.6
- Group 1 removal of packaging easier to use at first application than Groups 2 and 3 (p = .006)
- No statistical differences in removal from packaging or application at 6 weeks of use and on last dressing (p = NS)
- Group 1 easier to remove after use than Groups 2 or 3 at 6 weeks and at last use (p = .021 and .037, respectively)

Power calculation:

- Was a power calculation for the primary endpoint reported, and if so, was it appropriate? N

Adjustment for multiplicity of statistical testing

- Was an adjustment reported if there were more than 1 secondary endpoints statistically tested? N

Blinding

- Were patients blinded from treatment? N
- Were assessing investigators blinded from patient treatment? N

Allocation concealment

- Was allocation concealment carried out? N

Lost to follow-up/discontinued/withdrawn:

- 6/91 (6.6%) total
    - 1/31 (3.2%) from Group 1
    - 4/31 (12.9%) from Group 2
    - 1/29 (3.4%) from Group 3

17. Coccheri S, Scondotto G, Agnelli G, Aloisi D, Palazzini E, Zamboni V. Randomised, double blind, multicentre, placebo controlled study of sulodexide in the treatment of venous leg ulcers. *Thromb Haemost.* 2002;87(6):947-952.

Study Groups: Group 1: sulodexide; Group 2: saline placebo vials and placebo capsules

Statistical results:

- ITT analysis done for primary endpoint(s): N
- **Primary endpoint:** Complete ulcer healing after 2 months in 35% (42/120; 95 CI: 27-44) in Group 1 vs 20.9% (23/11; 95% CI: 14-30) in Group 2, difference in healed (SE; 95% CI): 14.1% (6.8, 3 -26), p = .018
    - For compression bandaging population: 41/102 (difference in healed: 16.5% (6.6, 4-29), p = .014
    - At 3 months: 52.5% healed (63/120, 95% CI: 43-62) in Group 1 vs 32.7% (36/110; 95% CI: 27-42) in Group 2, difference in healed 19.8% (6.4; 7-32), p = .004
        - For compression bandaging population: 58.8% (60/102; 95% CI: 49-68) in Group 1 vs 38.7% (36/93; 29-49); difference in healed: 20.1% (7.0, 6-34), p = .005
- Time course changes of ulcer surface areas(linear regression of areas with time) were significant for Group 1 (p = .004) but not Group 2
    - Multivariate treatment effects on time course changes of area: p = .038

For Peer Review

- In multivariate analysis, the interaction drug treatment-compression bandaging on time course of healing was significant, p = .011
  - There were no significant effects on the rate of change of ulcer surface area by ulcer history, depth, use of compression bandaging, sex, treatment – ulcer size, treatment – ulcer depth, treatment – ulcer history interactions.
- The effect of sulodexide at 2 months was especially relevant in ulcers with initial area ≤10 cm^2 (p <.019) and of up to 12 months onset (p = .039), which was confirmed at 3 months.
- Fibrinogen levels were reduced (p = .006) by treatment end in Group 1 (from 346.2 [81.0] to 329.6 [81.4]), but were unchanged in Group 2.
- There was no center-related differences for any of the outcomes, p = NS.
- 19.1% (23) in Group 1 and 15.4% (17) in Group 2 had AEs, p = NS.

Power calculation:
- Was a power calculation for the primary endpoint reported, and if so, was it appropriate? Y, N

Adjustment for multiplicity of statistical testing:
- Was an adjustment reported if there were more than 1 secondary endpoints statistically tested? N

Blinding:
- Were patients blinded from treatment? Y
- Were assessing investigators blinded from patient treatment? Y

Allocation concealment:
- Was allocation concealment carried out? N

Lost to follow-up/discontinued/withdrawn:
- 60/235 (25.5%) total
  - 32/121 (26.4%) withdrawn from Group 1
  - 28/114 (24.6%) withdrawn from Group 2

18. Cullen BM, Serena TE, Gibson MC, Snyder RJ, Hanft JR, Yaakov RA. Randomized controlled trial comparing collagen/oxidized regenerated cellulose/silver to standard of care in the management of venous leg ulcers. *Adv Skin Wound Care.* 2017;30(10):464-468.

Study Groups: Group 1: collagen/oxidized regenerated cellulose/silver + SOC; Group 2: SOC

Statistical results:
- ITT analysis done for primary endpoint(s): Y
- **Primary endpoint:** wound healing at 12 weeks, but no p value reported
- Percent of subjects reporting at least 1 AE: 14% in Group 1 vs 26% in Group 2, p = .478

Power calculation:
- Was a power calculation for the primary endpoint reported, and if so, was it appropriate? N

Adjustment for multiplicity of statistical testing:
- Was an adjustment reported if there were more than 1 secondary endpoints statistically tested? N

For Peer Review

Blinding:

- Were patients blinded from treatment? N
- Were assessing investigators blinded from patient treatment? N

Allocation concealment:

- Was allocation concealment carried out? N

Lost to follow-up/discontinued/withdrawn:

- 5/49 (10.2%) total
  - 2/22 (9.1%) withdrawn from Group 1
  - 3/27 (11%) withdrawn from Group 2

19. Da Costa RM, Ribeiro Jesus FM, Aniceto C, Mendes M. Randomized, double-blind, placebo-controlled, dose-ranging study of granulocyte-macrophage colony stimulating factor in patients with chronic venous leg ulcers. *Wound Repair Regen.* 1999;7(1):17-25.

Study Groups: Group 1: recombinant human granulocyte-macrophage colony-stimulating factor (rhuGM-CSF) 200 µg; Group 2: rhuGM-CSF 400 µg; Group 3: placebo

Statistical results:

- ITT analysis done for primary endpoint(s): Y
- **Primary endpoint:** Complete healing at 12-14 weeks: Group 1: 12 (57%), Group 2: 11 (61.%); Group 3: 4 (19%); Groups 1 and 2 vs Group 3: p = .014
- **Primary endpoint:** Reduction of ≥50% in area at 12-14 weeks: Group 1: 17 (81%); Group 2: 14 (78%); Group 3: 11 (52%); Groups 1 and 2 vs Group 3: p = .04
- Difference in rhuGM-CSF dose between complete responders (46.6 µg/cm^2, SD 55.5) and non-complete responders (12.5 µg/cm^2, SD 17.1), p <.005
  - Difference in dose between partial responders (37.0 µg/cm^2, SD 50.4) and non-responders (15.4 µg/cm^2, SD 23.7), p = .052
  - Total dose between 100 and 150 µg/cm^2 for complete healing was the most cost-effective, R^2 = .22; p = .0007 (same conclusion for partial response)
- Probability of complete and partial response is dose dependent and reaches a plateau at about 1500 µg/cm^2
  - There was a Kaplan-Meier association between dose and cumulative probability of response (p = .002), which is significant also by the logrank test (p = .01)
- Among the factors of sex, age, ulcer duration time, and initial area only initial area was significant (p= .0001)
- Statistically significant elevations in leukocyte and neutrophil counts and prothrombin times were noted in Groups 1 and 2 compared to Group 3 (no data, p > .05), but these differences were not considered clinically significant

Power calculation:

- Was a power calculation for the primary endpoint reported, and if so, was it appropriate? Y, Y

Adjustment for multiplicity of statistical testing:

- Was an adjustment reported if there were more than 1 secondary endpoints statistically tested? N

Blinding:

For Peer Review

- Were patients blinded from treatment? Y
- Were assessing investigators blinded from patient treatment? Y

Allocation concealment:

- Was allocation concealment carried out? N

Lost to follow-up/discontinued/withdrawn:

- 5/61 (8.2%) total
    - 1/21 (4.8%) withdrawn from Group 1
    - 3/18 (16.7%) withdrawn from Group 2
    - 0/21 (0%) withdrawn from Group 3
    - 1 additional patient withdrawn after randomization but before treatment

20. Dale JJ, Ruckley CV, Harper DR, Gibson B, Nelson EA, Prescott RJ. Randomised, double blind placebo controlled trial of pentoxifylline in the treatment of venous leg ulcers. *BMJ.* 1999;319(7214):875-878.

Study Groups: Group 1: pentoxifylline 3 times daily; Group 2: matching placebo tablets

Statistical results:

- ITT analysis done for primary endpoint(s): Y
- **Primary endpoint:** complete healing of all ulcer by 24 weeks: at the 5% level, pentoxyfylline had a nonsignificant effect on healing rates of pure venous ulcers
    - 64% (65) in Group 1 and 53% (52) in Group 2 healed by 24 weeks

Power calculation:

- Was a power calculation for the primary endpoint reported, and if so, was it appropriate? Y, Y

Adjustment for multiplicity of statistical testing:

- Was an adjustment reported if there were more than 1 secondary endpoints statistically tested? N

Blinding:

- Were patients blinded from treatment? Y
- Were assessing investigators blinded from patient treatment? Y

Allocation concealment:

- Was allocation concealment carried out? N

Lost to follow-up/discontinued/withdrawn:

- 22/200 (11%) total
    - 11/101 (10.9%) withdrawn from Group 1
    - 11/99 (11.1%) withdrawn from Group 2

21. Damaso Fernandez-Gines F, Cortinas-Saenz M, et al. Efficacy and safety of topical sevoflurane in the treatment of chronic skin ulcers. *Am J Health Syst Pharm.* 2017;74(9):e176-e82.

Study Groups: Group 1: Sevoflurane instillations + SOC; Group 2: SOC

Statistical results:

- ITT analysis done for primary endpoint(s): N
- **Primary endpoint:** Mean (SD) pain VAS score:

For Peer Review

    - At baseline: 7.4 (1.6) in Group 1 vs 7.8 (1.8) in Group 2, p = .60
    - At 24 hr: 1.6 (0.5) vs 7.6 (0.4), p = .001
    - At 30 days: 1.5 (1.3) vs 6.4 (1.2), p = .001
    - At 60 days: 1.8 (1.1) vs 5.1 (1.9), p = .001
    - At 90 days: 0.6 (0.5) vs 4 (1.1), p = .001
- **Primary endpoint:** Mean (SD) ulcer surface area (cm^2):
    - At 30 days: 10.8 (4.3) in Group 1 vs 10.2 (2.3) in Group 2, p = .049
    - At 60 days: 8.6 (3.3) vs 8.9 (2.8), p = .049
    - At 90 days: 6.1 1(2.7) vs 9.0 (3.2), p = .030
- Mean (SD) posttreatment morphine sulfate usage (mg/24 hrs): 20.0 (5.4) in Group 1 vs 72.0 (18.5) in Group 2, p = .001
- Mean (SD) no. emergency admissions during treatment: 1.4 (0.5) in Group 1 vs 4.4 (0.5), p = .001
- Outcome per Patient Global Impression of Improvement Scale (no. patients), therapeutic success: 10 in Group 1 vs 0 in Group 2, p = .001
- Outcome per Clinical Global Impression of Improvement scale (no. patients), therapeutic success: 9 in Group 1 vs 1 in Group 2, p = .001
- Adverse effects (no. patients)
    - Systemic effects: 1 in Group 1 vs 4 in Group 2, p = .02
    - Pruritus and mild reddening: 4 in Group 1 vs 0 in Group 2, p = .35

Power calculation:
- Was a power calculation for the primary endpoint reported, and if so, was it appropriate? N

Adjustment for multiplicity of statistical testing:
- Was an adjustment reported if there were more than 1 secondary endpoints statistically tested? N

Blinding:
- Were patients blinded from treatment? N
- Were assessing investigators blinded from patient treatment? N

Allocation concealment:
- Was allocation concealment carried out? N

Lost to follow-up/discontinued/withdrawn:
- 1/16 (6.2%) total
    - 1/11 (9.1%) withdrawn from Group 1
    - 0/5 (0%) withdrawn from Group 2

22. de Abreu AM, de Oliveira BG. A study of the Unna Boot compared with the elastic bandage in venous ulcers: a randomized clinical trial. *Rev Lat Am Enfermagem.* 2015;23(4):571-577.

Study Groups: Group 1: Unna Boot; Group 2: Elastic bandage

Statistical results:
- ITT analysis done for primary endpoint(s): N
- **Primary endpoint:** Median ulcer area (range), cm^2, 1-13 weeks

For Peer Review

- Group 1: Week 1: 28.0 (13.5-82), Week 5: 19.0 (8.5-32), Week 9: 11.5 (5-42), Week 13: 9.0 (0-28)
  - Significant reduction ($p < .0001$) occurred from Week 1 to 5, Week 9 to 13, and Week 5 to 13.
- Group 2: Week 1: 15.0 (6-52.5), Week 5: 7.0 (3.5-59.5), Week 9: 9.0 (0-19.5), Week 13: 8.0 (0-61.5)
  - There was a tendency for area reduction ($p = .06$) only after week 5

- Granulation difference between groups, $p = .46$
  - Granulation, 75%-100%: 7 (77.8%) in Group 1 vs 5 (55.6%) in Group 2
  - Devitalized tissue, 26-50%: 1 (11.1%) in Group 1 vs 2 (22.2%) in Group 2
  - Hypergranulation: 0 (0%) in Group 1 vs 2 (22.2%) in Group 2
  - Hypergranulation/devitalized: 1 (11.1%) in Group 1 vs 0 (0%) in Group 2
- Exudate amount difference between groups, $p = .15$
  - Little: 0 (0%) in Group 1 vs 4 (44.4%) in Group 2
  - A lot: 9 (100%) in Group 1 vs 5 (55.5%) in Group 2
- Pain present: 7 (77.8%) in Group 1 vs 7 (77.8%) in Group 2, $p = .71$
- Edema present: 8 (88.9%) in Group 1 vs 8 (88.9%) in Group 2, $p = .76$

Power calculation:

- Was a power calculation for the primary endpoint reported, and if so, was it appropriate? N

Adjustment for multiplicity of statistical testing:

- Was an adjustment reported if there were more than 1 secondary endpoints statistically tested? N

Blinding:

- Were patients blinded from treatment? N
- Were assessing investigators blinded from patient treatment? N

Allocation concealment:

- Was allocation concealment carried out? N

Lost to follow-up/discontinued/withdrawn:

- 1/19 (5.3%) total
  - 0/9 (0%) withdrawn from Group 1
  - 1/10 (10%) withdrawn from Group 2

23. de Araujo IC, Defune E, Abbade LP, et al. Fibrin gel versus papain gel in the healing of chronic venous ulcers: a double-blind randomized controlled trial. *Phlebology.* 2017;32(7):488-495.

Study Groups: Group 1: fibrin gel (n = 21); Group 2: 8% papain gel (n = 19); Group 3: carbopol gel (control) (n =23)

Statistical results:

- ITT analysis done for primary endpoint(s): Y
- **Primary endpoints:** reduction of wound area, evaluation of the wound bed (presence of vitalized or devitalized tissue), signs of critical colonization, presence of exudate
  - Median (IQR, range) of ulcer area along time, cm^2:

For Peer Review

    - day 0: 26 (12-45, 2.5-240) in Group 1 vs 9 (6-24, 5-111) in group 2 vs 9 (5.5-39, 3.7-105) in Group 3, p = .38
    - day 15: 20 (12-36, 2-192) in Group 1 vs 11 (6-25, 2-105) in group 2 vs 11 (4-45, 1.2-125) in Group 3, p = .62
    - day 30: 18 (7-35, 0.5-105) in Group 1 vs 12 (3.8-22, 1-135) in group 2 vs 11 (2.2-25, 1.8-111) in Group 3, p = .62
    - day 45: 14 (6-32, 0.2-111) in Group 1 vs 7 (4-14, 0.2-16) in group 2 vs 10 (1.5-20, 0.2-117) in Group 3, p = .62
    - day 60: 14 (5-28, 0.2-115) in Group 1 vs 5 (2.5-13, 0.2-96) in group 2 vs 6 (0.4-14, 0.2-117) in Group 3, p = .62
    - Throughout the study there was a similar reduction in ulcer area inside each group, p = 0.14
    - There was an agreement among examiners on the characteristics of the ulcer wound bed (Kappa = 1.00)
    - In all groups there was presence of devitalized tissue at baseline and similar reduction of devitalized tissues, critical colonization signs, and presence of exudate in the wound bed along time. Edge epithelization increase in all groups at 60 days, with no difference, p <.05
- Median (IQR, range) of pixel % related to blood vessels in each histological field:
    - day 0: 3.1 (2.6-4.6, 1.2-7.0) in Group 1 vs 4 (3.2-4.8, 1.8-6.3) in Group 2 vs 4.3 (3.0-5.3, 2.5-6.5) in Group 3, p =.46
    - day 60: 5.4 (4.3-6.4, 2.5-10.3) in Group 1 vs 6.2 (5.0-6.5, 4.8-7.0) in group 2 vs 5.3 (4.8-6.0, 4.6-6.3) in Group 3, p =.46
- Percentage (No.) of complete ulcer healing), Yes; No:
    - Yes: 14.3 (3); No: 85.7 (18), n = 21 in Group 1 vs Yes: 21.1 (4); No: 78.9 (15), n = 19 in Group 2 vs Yes: 30.4 (7); No: 69.6 (16), n = 23 in group 3, p = .43

Power calculation:

- Was a power calculation for the primary endpoint reported, and if so, was it appropriate? Y, N

Adjustment for multiplicity of statistical testing:

- Was an adjustment reported if there were more than 1 secondary endpoints statistically tested? N

Blinding:

- Were patients blinded from treatment? Y
- Were assessing investigators blinded from patient treatment? Y

Allocation concealment:

- Was allocation concealment carried out? N

Lost to follow-up/discontinued/withdrawn: None reported.

24. De Caridi G, Massara M, Acri I, et al. Trophic effects of polynucleotides and hyaluronic acid in the healing of venous ulcers of the lower limbs: a clinical study. *Int Wound J.* 2016;13(5):754-758.

Study Groups: Group 1: polynucleotides and hyaluronic acid (n = 20); Group 2: hyaluronic acid

For Peer Review

(n = 19)

Statistical results:

- ITT analysis done for primary endpoint(s): Y
- **Primary endpoint:** complete healing at 45 days (%): 12 (60%) in Group 1 vs 4 (22%) in Group 2, p = .135

Power calculation:

- Was a power calculation for the primary endpoint reported, and if so, was it appropriate? N

Adjustment for multiplicity of statistical testing:

- Was an adjustment reported if there were more than 1 secondary endpoints statistically tested? N

Blinding:

- Were patients blinded from treatment? N
- Were assessing investigators blinded from patient treatment? N

Allocation concealment:

- Was allocation concealment carried out? N

Lost to follow-up/discontinued/withdrawn: None reported.

25. DePalma RG, Kowallek D, Spence RK, et al. Comparison of costs and healing rates of two forms of compression in treating venous ulcers. *Vasc Surg.* 1999;33(6):683-690.

Study Groups: Group 1: Thera-Boot; Group 2: Unna's boot

Statistical results:

- ITT analysis done for primary endpoint(s): N
- **There were no primary endpoints identified.**
- Mean (SD) time to healing (weeks): 8 (4.4) in Group 1 vs 9.7 (3.3) in Group 2, p = .41
  - Mean (SD) area healing rate (cm^2/day): 0.0433 (0.0910) in Group 1 vs 0.0239 (0.0534) in Group 2, p = .27
  - Mean (SD) % area healing rate (%/day): 2.0357 (1.9520) vs 1.0493 (1.5583), p = .56
  - Mean (SD) linear healing rate (cm/day): 0.0109 (0.0125) vs 0.0060 (0.0092), p = .27
- Mean (SD) clinician costs ($): 201.91 (131.17) in Group 1 vs 331.37 (255.75) in Group 2, p = .09
- Mean (SD) materials costs ($): 122.79 in Group 1 vs 160.86 (96.86) in Group 2, p = .14
- Mean (SD) Overhead (no. visits): 6.7 (3.8) in Group 1 vs 11.7 (6.4), p = .02
- Mean (SD) total costs ($): 559.41 (290.75) in Group 1 vs 901.73 (576.45) in Group 2, p = .05.
- Mean (SD) time spent by physician with patients to completion (hours): 1.5 (0.8) in Group 1 vs 2.6 (2.0) in Group 2, p = .04
- Mean (SD) time spent by nurses with patients to completion (hours): 1.6 (1.4) in Group 2 vs 2 (1.6) in Group 1, p = .53.

Power calculation:

- Was a power calculation for the primary endpoint reported, and if so, was it

For Peer Review

appropriate? N

Adjustment for multiplicity of statistical testing:

- Was an adjustment reported if there were more than 1 secondary endpoints statistically tested? N

Blinding:

- Were patients blinded from treatment? N
- Were assessing investigators blinded from patient treatment? N

Allocation concealment:

- Was allocation concealment carried out? N

Lost to follow-up/discontinued/withdrawn:

- 28/38 (73.7%) total
  - 17/19 (89.5%) withdrawn from Group 1
  - 11/19 (57.9%) withdrawn from Group 2

26. De Sanctis MT, Incandela L, Belcaro G, Cesarone MR. Topical treatment of venous microangiopathy in patients with venous ulceration with Essaven gel--a placebo-controlled, randomized study. *Angiology.* 2001;52(Suppl 3): S29-S34.

Study Groups: Group 1: Essaven Gel (n = 11); Group 2: placebo gel (n = 10); Group 3: control (elastic stockings) (n = 9)

Statistical results:

- ITT analysis done for primary endpoint(s): Y
- **Primary endpoint:** complete ulcer healing at 4 weeks and improvement in microcirculatory parameters
- Variation of Laser Doppler flowmetry (LDF), Flux units and PO2/PCO2, mm Hg during the 4 weeks of study:
- Mean (SD) LDF at Inclusion time, 4 weeks: 3.9 (0.2), 1.8 (0.1) in Group 1 vs 3.8 (0.2), 3.6 (0.2) in Group 2 vs 3.8 (0.2), 3.9 (0.2) in Group 3
- Mean (SD) PO_2 at Inclusion time, 4 weeks: 53 (4), 61 (7) in Group 1 vs 52 (3), 54 (6) in Group 2 vs 51 (4), 55 (4) in Group 3
- Mean (SD) PCO_2 at Inclusion time, 4 weeks: 33 (3), 28 (3) in Group 1 vs 34 (4), 31 (4) in Group 2 vs 33 (3), 33 (4) in Group 3
- Decrease in flux of CO_2 and Increase on PO_2 in Group 1 vs Group 2 and Group 3 were significant, p = .02
- The analogue scale line score

Power calculation:

- Was a power calculation for the primary endpoint reported, and if so, was it appropriate? N

Adjustment for multiplicity of statistical testing:

- Was an adjustment reported if there were more than 1 secondary endpoints statistically tested? N

Blinding:

- Were patients blinded from treatment? N
- Were assessing investigators blinded from patient treatment? N

Allocation concealment:

- Was allocation concealment carried out? N

Lost to follow-up/discontinued/withdrawn: None reported.

27. Dolibog P, Franek A, Taradaj J, et al. A comparative clinical study on five types of compression therapy in patients with venous leg ulcers. *Int J Med Sci.* 2014;11(1):34-43.

Study Groups: Group 1: Intermittent pneumatic compression (n = 28); Group 2: Stockings (n = 30); Group 3: multilayer compression (n = 29); Group 4: 2-layer short stretch bandages (n = 30); Group 5: Unna boots (n = 30)

Statistical results:

- ITT analysis done for primary endpoint(s): Y
- **Primary endpoint:** Mean (SD) total surface area, cm^2 before vs after treatment: Group 1: 25.2 (31.2) vs 10.1 (20.9), p = .01; Group 2: 24.4 (20.2) vs 9.7 (20), p = .01; Group 3: 22.1 (14) vs 8.1 (17.2), p = .01; Group 4: 22.4 (11.9) vs 16.3 (20.2), p = .03; Group 5: 21.9 (10.1) vs 15.8 (19.6), p = .03
- **Primary endpoint:** Mean (SD) length, cm before vs after treatment: Group 1: 5.8 (3) vs 3.2 (4.9), p = .02; Group 2: 5.1 (3.1) vs 3.2 (5.3), p = .02; Group 3: 4.6 (3.1) vs 3 (5.1), p = .02; Group 4: 4.9 (4) vs 3.9 (5.2), p = .03; Group 5: 4.8 (4) vs 3.7 (5), p = .03
- **Primary endpoint:** Mean (SD) width, cm before vs after treatment: Group 1: 3.8 (2.9) vs 2.5 (5), p = .02; Group 2: 3.3 (2.4) vs 2.3 (4.1), p = .02; Group 3: 3,2 (1.7) vs 2.4 (5), p = .02; Group 4: 3.3 (2) vs 2.9 (5.2), p = .03; Group 5: 3.1 (1.9) vs 2.7 (5.1), p = .03
- **Primary endpoint:** Mean (SD) volume, cm^3 before vs after treatment: Group 1: 2.4 (3.1) vs 0.38 (2.1), p = .01; Group 2: 2.4 (4) vs 0.41 (2.1), p = .01; Group 3: 1.8 (4.8) vs 0.42 (2.2), p = .01; Group 4: 1.7 (4) vs 0.39 (2.8), p = .01; Group 5: 1.4 (3.9) vs 0.41 (2.7), p = .01
- Healing rate: Group 1 vs 4: 57.1% vs 16.7%, p = .03; Group 2 vs 4: 56.7% vs 16.7%, p = .03; Groups 3 vs 4: 58.6% vs 16.7%, p = .03; Groups 1 vs 5: 57.1% vs 20.0%, p = .03; Groups 2 vs 5: 56.7% vs 20.0%, p = .03; Groups 3 vs 5: 58.6% vs 20.0%, p = .03
- Gilman Index (cm) comparison: Groups 1 and 4: 0.52 vs 0.20, p = .04; Groups 2 and 4: 0.49 vs 0.20, p = .04; Groups 3 and 4: 0.51 vs 0.20, p = .04; Groups 1 and 5: 0.52 vs 0.23, p = .04; Groups 2 and 5: 0.49 vs 0.23, p = .04; Groups 3 and 5: 0.51 vs 0.23, p = .04
- Mean PAR comparison: Groups 1 and 4: 48.1 vs 17.8%, p = .04; Groups 2 and 4: 41.2% vs 17.8%, p = .04; Groups 3 and 4: 49% vs 17.8%, p = .04; Groups 1 and 5: 48.1% vs 20.5%, p = .04; Groups 2 and 5: 41.2% vs 20.5%, p = .04; Groups 3 and 5: 49% vs 20.5%, p = .04

Power calculation:

- Was a power calculation for the primary endpoint reported, and if so, was it appropriate? N

Adjustment for multiplicity of statistical testing:

- Was an adjustment reported if there were more than 1 secondary endpoints statistically tested? N

Blinding:

- Were patients blinded from treatment N
- Were assessing investigators blinded from patient treatment? N

For Peer Review

Allocation concealment:

- Was allocation concealment carried out? Y

Lost to follow-up/discontinued/withdrawn: None reported.

28. Edwards H, Courtney M, Finlayson K, et al. Chronic venous leg ulcers: effect of a community nursing intervention on pain and healing. *Nurs Stand.* 2005;19(52):47-54.

Study Groups: Group 1: Leg Club community nursing intervention (n =28); Group 2: standard community nursing intervention at home (n = 28)

Statistical results:

- ITT analysis done for primary endpoint(s): Y
- **Primary endpoint:** Mean (SD) differences in levels of pain, based on Rand Medical Outcomes Study Pain Measures 1-5
  - Amount of pain at baseline vs week 12
    - Group 1: 4.04 (0.94) vs 3.09 (1.38)
    - Group 2: 3.39 (1.57) vs 3.29 (1.52)
    - Group difference: Z = 3.02, p = .001
  - Impact of pain on mood at baseline vs week 12
    - Group 1: 2.44 (1.25) vs 1.65 (0.89)
    - Group 2: 2.00 (1.22) vs 2.05 (1.12)
    - Group difference: Z = 2.65, p = .004
  - Impact of pain on sleep at baseline vs week 12
    - Group 1: 2.44 (1.12) vs 1.80 (1.04)
    - Group 2: 2.21 (1.17) vs 2.43 (1.34)
    - Group difference: Z = 2.71, p = .003
  - Impact of pain on normal work at baseline vs week 12
    - Group 1: 2.81 (1.18) vs 2.04 (1.17)
    - Group 2: 2.61 (1.67) vs 2.48 (1.41)
    - Group difference: Z = 1.94, p = .026
- **Primary endpoint:** Healing progression at 12 weeks
  - Mean ulcer area (cm^2) at baseline vs 12 weeks
    - Group 1: 10.3 vs 2.39
    - Group 2: 7.63 vs 6.8
    - Group difference: Z = 2.64, p = .004
  - Percent healed at 12 weeks: 46.2 (n = 12) in Group 1 vs 25.9 (n = 7) in Group 2, p chi squared = 2.36, p = .125
  - PAR >50%: 74.2% (n = 20) in Group 1 vs 59.1% (n = 16) in Group 2, chi squared = 11.11, p = .025
- Mean PUSH Score at baseline vs 12 weeks
  - Group 1: 10.45 vs 4.88
  - Group 2: 9.11 vs 6.92
  - Group difference: Z = 2.93, p = .002

Power calculation:

- Was a power calculation for the primary endpoint reported, and if so, was it

appropriate? Y, N

Adjustment for multiplicity of statistical testing:

- Was an adjustment reported if there were more than 1 secondary endpoints statistically tested? N

Blinding:

- Were patients blinded from treatment? N
- Were assessing investigators blinded from patient treatment? N

Allocation concealment:

- Was allocation concealment carried out? N

Lost to follow-up/discontinued/withdrawn: None reported.

29. Escamilla Cardenosa M, Dominguez-Maldonado G, Cordoba-Fernandez A. Efficacy and safety of the use of platelet-rich plasma to manage venous ulcers. *J Tissue Viability.* 2017;26(2):138-143.

Study Groups: Group 1: Plasma Rich in Growth Factors; Group 2: cures with saline (control)

Statistical results:

- ITT analysis done for primary endpoint(s): N
- **Primary endpoint:** Mean skin cicatrization (cm^2)
    - Baseline: 13.7 +/- 30 in Group 1 vs 16.7 +/- 23.9 in Group 2
    - After intervention: 10 +/- 30 in Group 1 vs 12.1 +/- 19.23 in Group 2
    - Difference among groups, p = .001
- Mean pain score
    - Baseline: 5.6 +/- 2.3 in Group 1 vs 5.5 +/- 2.6 in Group 2
    - After intervention: 3.2 (2.5) in Group 1 vs 4.9 (2.5) in Group 2
    - Difference among groups, p <.001

Power calculation:

- Was a power calculation for the primary endpoint reported, and if so, was it appropriate? Y, N

Adjustment for multiplicity of statistical testing:

- Was an adjustment reported if there were more than 1 secondary endpoints statistically tested? N

Blinding:

- Were patients blinded from treatment? N
- Were assessing investigators blinded from patient treatment? N

Allocation concealment:

- Was allocation concealment carried out? N

Lost to follow-up/discontinued/withdrawn:

- 3/61 (4.9%) patients total, but it makes no sense because there were 102 ulcers enrolled and 102 analyzed, even though there were only 58 patients analyzed.
    - 2/31 (6.4%) in Group 1
    - 1/30 (3.3% in Group 2)

30. Evangelista MT, Casintahan MF, Villafuerte LL. Simvastatin as a novel therapeutic agent

For Peer Review

for venous ulcers: a randomized, double-blind, placebo-controlled trial. *Br J Dermatol.* 2014; 170(5):1151-1157.

Study Groups: Group 1: simvastatin; Group 2: placebo tablets of the same size, color, and shape placed in identical bottles

Statistical results:

- ITT analysis done for primary endpoint(s): N
- **Primary endpoint:** proportion with complete healing: 90% in Group 1 vs 34% in Group 2, RR: 0.158, 95% CI: 0.053-0.474
  - Ulcers ≤5 cm, 100% in Group 1 vs 50% in Group 2, RR: 0.10, 95% CI: 0.0141-0.707
  - Ulcers >5 cm, 67% in Group 1 vs 0% in Group 2, RR: 0.33, 95% CI: 0.132-0.840
- Among ulcers ≤5 cm, mean (SD) healing times were 6.89 (0.78) weeks for Group 1 vs 8.40 (1.13) for Group 2, p = .001
- Mean healed area: 28.9 cm^2 in Group 1 vs 19.6 cm^2 , p = .030
- Comparison of mean (SD) age (years) on healing status:
  - Group 1 closed: 51.8 (14.2) vs open: 72.9 (3), p = .001
  - Group 2 closed: 55.2 (14.5) vs open: 56.4 (15.5), p = NS
- Comparison of diabetes, n (%) on healing status:
  - Group 1 closed: 2 (8) vs open: 1 (14), p = NS
  - Group 2 closed: 0 (0) vs open: 5 (22), p = NS
- Comparison of sex, male, n (%) on healing status:
  - Group 1 closed: 8 (32) vs open: 2 (29), p = NS
  - Group 2 closed: 4 (36) vs open: 9 (39), p = NS
- Comparison of mean (SD) duration of venous disease (years) on healing status:
  - Group 1 closed: 8.6 (2.9) vs open: 10.3 (5.5), p = NS
  - Group 2 closed: 7.0 (5.4) vs open: 9.9 (3.4), p = NS
- Comparison of mean (SD) duration of venous ulcer (years) on healing status:
  - Group 1 closed: 3.3 (2.3) vs open: 6 (0.8), p = .026
  - Group 2 closed: 2.4 (2.4) vs open: 4.5 (2.3), p = .025
- Comparison of mean (SD) ulcer surface area (cm^2) on healing status:
  - Group 1 closed: 30.9 (21) vs open: 60 (3.4), p = .012
  - Group 2 closed: 12.2 (3.3) vs open: 46.3 (17.9), p < .001
- Group 1 showed a significantly lower posttreatment Dermatology Life Quality Index score than Group 2, 3 vs 5.9, p <.001
  - Group 1 also showed a higher percentage reduction, 78.% vs 53.5%, p <.001
- Group 1 had significantly lower posttreatment total and LDL cholesterol (p < .010) and significantly higher posttreatment HDL cholesterol (p = .040).
- There were no significant differences in posttreatment serum glutamic pyruvic transaminase or serum glutamic oxaloacetic transaminase, p = NS.

Power calculation:

- Was a power calculation for the primary endpoint reported, and if so, was it appropriate? Y, N

Adjustment for multiplicity of statistical testing:

- Was an adjustment reported if there were more than 1 secondary endpoints statistically

For Peer Review

tested? N

Blinding:

- Were patients blinded from treatment? Y
- Were assessing investigators blinded from patient treatment? Y

Allocation concealment:

- Was allocation concealment carried out? Y

Lost to follow-up/discontinued/withdrawn:

- 5/66 (7.6%) total
    - 3/32 (9.4%) withdrawn from Group 1
    - 2/34 (5.9%) withdrawn from Group 2

31. Falanga V, Fujitani RM, Diaz C, et al. Systemic treatment of venous leg ulcers with high doses of pentoxifylline: efficacy in a randomized, placebo-controlled trial. *Wound Repair Regen*. 1999;7(4):208-213.

Study Groups: Group 1: pentoxifylline 400 mg; Group 2: pentoxyfilline 800 mg; Group 3: placebo tablet

Statistical results:

- ITT analysis done for primary endpoint(s): N
- **Primary endpoint:** Median time to complete healing, days: 83 for Group 1; 71 for Group 2; 100 for Group 3.
    - Group 2 healed significantly faster than Group 3, p = .043, Wilcoxon test
    - Group 1 did not heal significantly faster than Group 3, p = .191 Wilcoxon test
- % of ulcers healed by week 24: 71% in Group 1, 70% in 2, and 60% in Group 3
    - The visits by which over half of ulcers and references ulcers were healed were week 12 for Groups 1 and 2 and Week 16 for Group 3
    - Group 2 was superior to healing reference ulcers, p = .40, Wilcoxon test
- Ulcer size at baseline (<350 mm^2 or >350 mm^2) did not significantly affect healing, p = NS

Power calculation:

- Was a power calculation for the primary endpoint reported, and if so, was it appropriate? Y, Y

Adjustment for multiplicity of statistical testing:

- Was an adjustment reported if there were more than 1 secondary endpoints statistically tested? N

Blinding:

- Were patients blinded from treatment? Y
- Were assessing investigators blinded from patient treatment? Y

Allocation concealment:

- Was allocation concealment carried out? N

Lost to follow-up/discontinued/withdrawn:

- 34/131 (26%) total; 2 did not receive study treatment and were excluded from ITT
    - 11/41 (26.8%) withdrawn from Group 1
    - 11/43 (25.6%) withdrawn from Group 2
    - 10/45 (22.2%) withdrawn from Group 3

For Peer Review

32. Falanga V, Margolis D, Alvarez O, et al. Rapid healing of venous ulcers and lack of clinical rejection with an allogeneic cultured human skin equivalent. Human Skin Equivalent Investigators Group. *Arch Dermatol.* 1998;134(3):293-300.

Study Groups: Group 1: human skin equivalent (HSE) + SOC; Group 2: SOC (control)

Statistical results:

- ITT analysis done for primary endpoint(s): N
- **Primary endpoint:** No. (%) of patients with healed wounds by 6 mo: 92/146 (63.0%) in Group 1 vs 63/129 (48.8%) in Group 2, p = .02
- Median days to 50% wound closure (range): 23 (3-185) in Group 1 vs 29 (3-232) in Group 2, p = .02
- Median days to 75% wound closure (range): 30 (3-189) in Group 1 vs 50 (4-232) in Group 2, p = .001
- Median days to 100% wound closure (range): 61 (9-233) in Group 1 vs 181 (10-232) in Group 2, p = .003
- Group 1 had a 54% better changes for wound closure per unit time than Group 2 (p < .001; 95% CI: 1.275-1.855)
- Among subjects with ulcers >6 mos duration, Group 1 healed significantly faster than Group 2: median of 92 days vs 190 days, p = .001, log-rank test)
- Among subjects with ulcer <6 mos duration, Group 1 healed in median 46 days vs 89 days in Group 2, p > .05
- Among patients with stage III ulcers (down to muscle), Group 1 healed in median of 83 days vs 183 days, p = .003, log-rank test
- Among stage II ulcers (superficial), Group healed in median of 57 days vs 98 days, p >.05
- Among large ulcers (>1000 mm^2), Group 1 healed in median of 181 days vs 231 days, p = .02
- Among small ulcers (<1000 mm^2), Group 1 healed in median of 56 days vs 89 days, p = .04
- Ulcers recurred in 11 (12%) of 92 patients in Group 1 vs 10 (15.9%) of 63 patients in Group 2, p = .48
- No significant differences in the distribution of positive and negative proliferative responses to keratinocytes and fibroblasts between groups (p = NS)
- No significant changes in response from baseline to any time point (p >.05)
- AEs were similar in both groups; no significant differences between groups (p = NS)

Power calculation:

- Was a power calculation for the primary endpoint reported, and if so, was it appropriate? N

Adjustment for multiplicity of statistical testing:

- Was an adjustment reported if there were more than 1 secondary endpoints statistically tested? N

Blinding:

- Were patients blinded from treatment? N
- Were assessing investigators blinded from patient treatment? N

For Peer Review

Allocation concealment:

- Was allocation concealment carried out? N

Lost to follow-up/discontinued/withdrawn:

- 24/309 (11%) total
    - 22/146 (15.1%) withdrawn from Group 1
    - 33/129 (25.6%) withdrawn from Group 2

33. Ferrara F, Meli F, Raimondi F, et al. The treatment of venous leg ulcers: a new therapeutic use of iloprost. *Ann Surg.* 2007;246(5):860-865.

Study Groups: Group 1: iloprost; Group 2: placebo saline

Statistical results:

- ITT analysis done for primary endpoint(s): N
- **There were no primary endpoints identified.**
- Percentage of healed ulcers: 100 at 90 days in Group 1 vs 50 at 105 days in Group 2 and at 150 days, only 84.1% had healed in Group 2, $p < .05$
- Mean (SD) of dimensions of ulcers by time, cm^2:
    - Day 0: 19.4 (5.6) in Group 1 vs 19.8 (4.5) in Group 2, p = NS
    - Day 15: 15.6 (5.1) in Group 1 vs 17.2 (5.0) in Group 2, p = NS
    - Day 30: 11.6 (4.6) in Group 1 vs 15.0 (5.3) in Group 2, p = .001
    - Day 45: 7.6 (4.0) in Group 1 vs 12.6 (5.4) in Group 2, p < .0001
    - Day 60: 3.9 (3.2) in Group 1 vs 10.3 (5.5) in Group 2, p < .0001
    - Day 75: 1.4 (1.4) in Group 1 vs 8.2 (5.6) in Group 2, p < .0001
    - Day 90: 0 (0) in Group 1 vs 5.7 (5.1) in Group 2, p < .0001
    - Day 105: 0 (0) in Group 1 vs 3.4 (4.6) in Group 2, p < .0001
    - Day 120: 0 (0) in Group 1 vs 2.2 (3.7) in Group 2, p = .0003
    - Day 135: 0 (0) in Group 1 vs 1.1 (2.1) in Group 2, p = .001
    - Day 150: 0 (0) in Group 1 vs 0.133333 (0.547723) in Group 2, p = NS

Power calculation:

- Was a power calculation for the primary endpoint reported, and if so, was it appropriate? N

Adjustment for multiplicity of statistical testing:

- Was an adjustment reported if there were more than 1 secondary endpoints statistically tested? N

Blinding:

- Were patients blinded from treatment? Y
- Were assessing investigators blinded from patient treatment? N

Allocation concealment:

- Was allocation concealment carried out? N

Lost to follow-up/discontinued/withdrawn:

- 87 completed the study (43 in Group 1 vs 44 in Group 2), but there were 11 who did not and the authors are not clear if those 11 were in addition to the 87 (ie, were 98 originally randomized?)

For Peer Review

34. Finlayson KJ, Courtney MD, Gibb MA, O'Brien JA, Parker CN, Edwards HE. The effectiveness of a four-layer compression bandage system in comparison with Class 3 compression hosiery on healing and quality of life in patients with venous leg ulcers: a randomised controlled trial. *Int Wound J.* 2014;11(1):21-27.

Study Groups: Group 1: 4-layer compression; Group 2: Class 3 compression hosiery

Statistical results:

- ITT analysis done for primary endpoint(s): N
- **Primary endpoint:** proportion healed at 24 weeks: 84% in Group 1 vs 72% in Group 2, chi square = 2.16, p = .14
- Mean (SD) PAR: Group 1: 96 (15.6); Group 2: 93 (14.9), p = .27
- Median time to healing, weeks: Group 1: 10; Group 2: 15, p = .003
- Healing time was significantly delayed when ulcer duration >24 weeks (p <.001), baseline area >10 cm^2 (p = .03), PUSH score >10 (p = .005), Geriatric Depression Scale score >4 (p = .012)
- Healing time significantly shorter for those taking diuretics, p = .002
- No significant relationships between healing and age, gender, comorbidities, types of medications, restricted mobility, and dressing type, p = NS
- HR for healing – Cox proportional regression model: Beta, HR, 95% CI, and p value
    - Age: 0.015, 1.02, 0.99-1.03, p = .101
    - Taking diuretics: 0.311, 1.37, 0.76-2.43, p = 0.291
    - Ulcer area >10 cm^2: -0.677, 0.51, 0.22-1.00, p = .051
    - Ulcer duration >24 weeks: -0.950, 0.39, 0.22-0.67, p = .001
    - 4-layer bandage system: 0.91, 2.49, 1.44-4.29, p = .001
    - Depression score >4: -0.762, 0.47, 0.23-0.96, p = .037
- Mean (SD) QOL score (scale of 0 – 10) at baseline vs 24 weeks: 7.56 (2.20) in Group 1; 8.33 (1.72) in Group 2 vs 8.00 (2.36) in Group 1; 8.36 (2.43) in Group 2
    - Interaction effect: F = 1.19; p = .278
    - Main effect: F = 1.51; p = .223
- Mean (SD) Depression score (scale of 0 – 15) at baseline vs 24 weeks: 4.04 (2.75) in Group 1; 3.87 (3.84) in Group 2 vs 4.13 (3.58) in Group 1; 3.71 (3.76) in Group 2
    - Interaction effect: F = 0.02; p = .892
    - Main effect: F = 4.72; p = .035
- Mean (SD) pain score (scale of 0 – 100) at baseline vs 24 weeks: 51.8 (28.3) in Group 1; 50.0 (26.4) in Group 2 vs 23.0 (22.1) in Group 1; 34.0 (23.3) in Group 2
    - Interaction effect: F = 2.42; p = .124
    - Main effect: F = 35.2; p < .001

Power calculation:

- Was a power calculation for the primary endpoint reported, and if so, was it appropriate? Y, Y

Adjustment for multiplicity of statistical testing:

- Was an adjustment reported if there were more than 1 secondary endpoints statistically tested? N

Blinding:

For Peer Review

- Were patients blinded from treatment? N
- Were assessing investigators blinded from patient treatment? Y

Allocation concealment:

- Was allocation concealment carried out? N

Lost to follow-up/discontinued/withdrawn:

- 16/103 (15.5%) total
  - 8/53 (15.1%) withdrawn from Group 1
  - 8/50 (16.0%) withdrawn from Group 2

35. Frade MA, Assis RV, Coutinho Netto J, Andrade TA, Foss NT. The vegetal biomembrane in the healing of chronic venous ulcers. *An Bras Dermatol.* 2012;87(1):45-51.

Study Groups: Group 1: Vegetal biomembrane, n = 14; Group 2: collagenase (control), n = 7

Statistical results:

- ITT analysis done for primary endpoint(s): Y
- **Primary endpoints are not defined.**
- No significant difference between groups in terms of Ulcer Healing Index at 30 days, p = .39
- In terms of necrosis, inflammation, angiogenesis, elastosis, collagenic fibrosis, fibroblastic proliferation, only fibrine/debris had a significant decrease in Group 1 compared to Group 2, p = .013

Power calculation:

- Was a power calculation for the primary endpoint reported, and if so, was it appropriate? N

Adjustment for multiplicity of statistical testing:

- Was an adjustment reported if there were more than 1 secondary endpoints statistically tested? N

Blinding:

- Were patients blinded from treatment? N
- Were assessing investigators blinded from patient treatment? N

Allocation concealment:

- Was allocation concealment carried out? N

Lost to follow-up/discontinued/withdrawn: None reported.

36. Franek A, Chmielewska D, Brzezinska-Wcislo L, Slezak A, Blaszczak E. Application of various power densities of ultrasound in the treatment of leg ulcers. *J Dermatol Treat.* 2004;15(6):379-386.

Study Groups: Group 1: Ultrasound rated a 1 W/cm^2 (n = 22); Group 2: Ultrasound rate at 0.5 W/cm^2 (n = 21); Group 3: topical pharmacological treatment (n = 22)

Statistical results:

- ITT analysis done for primary endpoint(s): Y

There were no primary endpoints identified.

- Mean (median) changes of ulceration area, volume, and longest and widest dimensions:
  - Area before and after treatment, cm^2: 15.62 (12.51), 14 (11.14) in Group 1, p =

For Peer Review

.0001; 15.57 (6.71), 9.29 (3.78) in Group 2, p = .00006; 23.74 (11.72), 20.58 (9.86) in Group 3, p = .002

- Volume before and after treatment, cm^3: 3.62 (2.63), 3.06 (1.69) in Group 1, p = .0006; 2.86 (1.74), 0.77 (0.31) in Group 2, p = .0002; 8.26 (4.13), 4.87 (1.94) in Group 3, p = .00005
- Longest dimension before and after treatment, cm: 5.57 (5.30), 5.10 (4.95) in Group 1, p = .0002; 4.96 (3.60), 3.54 (2.20) in Group 2, p = .00006; 6.58 (5.40), 5.85 (5.00) in Group 3, p = .003
- Widest dimension before and after treatment, cm: 3.18 (3.00), 2.95 (2.80) in Group 1, p = .0008; 3.25 (2.00), 1.91 (1.30) in Group 2, p = .00006; 3.97 (3.30), 3.35 (2.85) in Group 2, p = .001

- Mean weekly rate of ulcer reduction, %: 7.6 in Group 1 vs 19.2 in Group 2 vs 9.3 in Group 3, p (1,2) = .0001, p (1,3) > .005, p (2,3) = .008
- Mean weekly rate of ulcer volume reduction, %: 9.6 in Group 1 vs 34.5 in Group 2 vs 22.6 in Group 3, p (1,2) = .0004, p (1,3) >.002, p (2,3) >.05
- Mean weekly rate of ulcer suppurate area reduction, %: 15.8 in Group 1 vs 42.8 in Group 2 vs 22.6 in Group 3, p (1,2) = .0001, p (1,3) >.05, p (2,3) = .003
- Percentage of relative ulcer suppurate area after treatment: 67.6 in Group 1 vs 22.2 in Group 2 vs 44.7 in Group 3, p (1,2) = .0005, p (1,3) = .03, p (2,3) = .03
- Percentage of relative granulated area change, before treatment (after treatment): 19.8 (30.9) in Group 1, p <.05 vs 29.3 (64) in Group 2, p = NS vs 23.5 (50.8) in Group 3, p < .03; p (1,2) = NS, p (1,3) = NS, p (2,3) = NS

Power calculation:

- Was a power calculation for the primary endpoint reported, and if so, was it appropriate? N

Adjustment for multiplicity of statistical testing:

- Was an adjustment reported if there were more than 1 secondary endpoints statistically tested? N

Blinding:

- Were patients blinded from treatment? N
- Were assessing investigators blinded from patient treatment? N

Allocation concealment:

- Was allocation concealment carried out? N

Lost to follow-up/discontinued/withdrawn: None reported

37. Franek A, Polak A, Kucharzewski M. Modern application of high voltage stimulation for enhanced healing of venous crural ulceration. *Med Eng Physics.* 2000;22(9):647-655.

Study Groups: Group 1: high voltage simulation (33); Group 2: topically applied medicine (32); Group 3: Unna's boot control (n = 14)

Statistical results:

- ITT analysis done for primary endpoint(s): Y
- **There were no primary endpoints identified.**
- Mean wound area before (after) treatment, cm^2: 22.7 (9.3) in Group 1 (n = 33), p <.001

vs 23.9 (15.6) in Group 2 (n = 32), p <.001 vs 10.5 (7.9) in Group 3 (n = 14), p <.001

- Mean tissue defect volume before (after) treatment, cm^3: 8.1 (1.1) in Group 1 (n = 31), p <.001 vs 7.5 (2.6) in Group 2 (n = 32), p <.001 vs 3.6 (1.0) in Group 3 (n = 13), p < .001
- Mean longest dimension before (after) treatment, cm: 6.5 (3.8) in Group 1 (n = 33), p < .001 vs 6.6 (5.2) in Group 2 (n = 32), p < .001 vs 5.3 (4.2) in Group 3 (n = 14), p <.001
- Mean widest dimension before (after) treatment, cm: 4.1 (2.0) in Group 1 (n = 33), p <.001 vs 4.2 (3.1) in Group 2 (n = 32), p <.001 vs 2.7 (2.1) in Group 3 (n = 14), p <.003
- Percentage weekly wound area change: 11.3 in group 1 vs 9 in Group 2 vs 8 in Group 3, p = NS
- Mean percent change of pus-covered area (after) treatment, cm^2: 15.5 (0.5) in Group 1, p <.05 vs 20.5 (3.2) in Group 2, p <.05 vs 8.0 (2.0) in Group 3, p <.05
- Mean weekly rate of change of pus-covered area, %: 53 in group 1 vs 26 in Group 2 vs 30.5 in Group 3,
    - The post hoc test determined there was significant difference in pus removal between Group 1 and group 2, in favor of Group 1, p <.0005
- Percentage of granulation relative area before, (and after treatment): 25 (97) in Group 1, p = NS vs 18 (84) in Group 2, p = NS vs 30 (84) in Group 3, p = NS
- Percentage of granulation relative area after 2 weeks of treatment: 85 in Group 1, vs 26 in Group 2 vs 63 in Group 3, p <.05

Power calculation:

- Was a power calculation for the primary endpoint reported, and if so, was it appropriate? N

Adjustment for multiplicity of statistical testing:

- Was an adjustment reported if there were more than 1 secondary endpoints statistically tested? N

Blinding:

- Were patients blinded from treatment? N
- Were assessing investigators blinded from patient treatment? N

Allocation concealment:

- Was allocation concealment carried out? N

Lost to follow-up/discontinued/withdrawn: None reported.

38. Franek A, Taradj J, Polak A, et al. Efficacy of high voltage stimulation for healing of venous leg ulcers in surgically and conservatively treated patients. *Phlébologie.* 2006;35(3):127-133.

Study Groups: Group 1: high voltage simulation (HSV) + compression and drug therapy, n = 28; Group 2: compression and drug therapy, n = 27; Group 3: surgical therapy + HSV + compression and drug therapy, n = 28; Group 4: surgical therapy + compression and drug therapy, n = 27

Statistical results:

- ITT analysis done for primary endpoint(s): Y
- **Primary endpoint:** complete ulcer healing, no. patients: 6 in Group 1, 2 in Group 2, 6 in Group 3, and 7 in Group 4
    - p (1,4) > .05; p (3,4) >.0; p (2,4) = .03; p (1,3) >.05; p (1,2) = .03; p (2,3) = .03

For Peer Review

- Mean relative change of wound length, %: 44 in Group 1, 26.2 in Group 2, 44 in Group 3, 41.9 in Group 4
    - p (1,4) >.05; p (3,4) >.05; p (2,4) = .03; p (1,3) >.05; p (1,2) = .02; p (2,3) = .02
- Mean relative change of wound width, %: 45 in Group 1, 33.8 in Group 2, 46.1 in Group 3, 45.1 in Group 4
    - p (1,4) >.05; p (3,4) >.05; p (2,4) = .04; p (1,3) >.05; p (1,2) = .04; p (2,3) = .04
- Mean relative change of surface area, %: 61.5 in Group 1, 44.1 in Group 2, 63.3 in Group 3, 60.2 in Group 4
    - p (1,4) >.05; p (3,4) >.05; p (2,4) = .03; p (1,3) >.05; p (1,2) = .03; p (2,3) = .03
- Mean relative change of surface area, %: 61.5 in Group 1, 44.1 in Group 2, 63.3 in Group 3, 60.2 in Group 4
    - p (1,4) >.05; p (3,4) >.05; p (2,4) = .03; p (1,3) >.05; p (1,2) = .03; p (2,3) = .03
- Mean relative change of wound volume, %: 86.5 in Group 1, 58.2 in Group 2, 87.1 in Group 3, 85.7 in Group 4
    - p (1,4) >.05; p (3,4) >.05; p (2,4) = .02; p (1,3) >.05; p (1,2) = .02; p (2,3) = .02
- Mean relative change of wound pus-covered area, %: 82.6 in Group 1, 71.1 in Group 2, 84.3 in Group 3, 64.3 in Group 4
    - p (1,4) =.01; p (3,4) =.01; p (2,4) > .05; p (1,3) >.05; p (1,2) = .01; p (2,3) = .01
- Mean relative change of wound pus-covered area after 2 weeks of treatment, %: 80.3 in Group 1, 40 in Group 2, 81.2 in Group 3, 45.2 in Group 4
    - p (1,4) =.003; p (3,4) = .003; p (2,4) > .05; p (1,3) >.05; p (1,2) = .003; p (2,3) = .003
- Mean relative change of wound granulation area, %: 83.2 in Group 1, 65.3 in Group 2, 85.6 in Group 3, 51.9 in Group 4
    - p (1,4) =.01; p (3,4) =.01; p (2,4) = .04; p (1,3) >.05; p (1,2) = .01; p (2,3) = .01
- Mean relative change of wound granulation area after 2 weeks of treatment, %: 74.2 in Group 1, 42.1 in Group 2, 79.3 in Group 3, 29 in Group 4
    - p (1,4) =.001; p (3,4) =.001; p (2,4) = .02; p (1,3) >.05; p (1,2) = .01; p (2,3) = .01

Power calculation:

- Was a power calculation for the primary endpoint reported, and if so, was it appropriate? N

Adjustment for multiplicity of statistical testing:

- Was an adjustment reported if there were more than 1 secondary endpoints statistically tested? N

Blinding:

- Were patients blinded from treatment? N
- Were assessing investigators blinded from patient treatment? N

Allocation concealment:

- Was allocation concealment carried out? N

Lost to follow-up/discontinued/withdrawn: None reported.

39. Franks PJ, Moody M, Moffatt CJ, et al. Randomized trial of cohesive short-stretch versus four-layer bandaging in the management of venous ulceration. *Wound Rep Regen.*

For Peer Review

2004;12(2): 157-162.

Study Groups: Group 1: cohesive short-stretch bandaging; Group 2: 4-layer bandaging

Statistical results:

- ITT analysis done for primary endpoint(s): Y
- **Primary endpoints:** healing rate: 56% healed at 12 weeks in both groups, which changed to 83% in Group 1 and 85% in Group 2 at 24 weeks, HR: 1.08 (95% CI: 0.63-1.85), p = .79
    - Among patients with a mobility deficit (n = 14 in Group 1 and n = 18 in Group 2), 75% healed in Group 1 at 24 weeks vs 70% in Group 2, HR = 1.35 (95% CI: 0.60 to 3.03), p = .46.

Power calculation:

- Was a power calculation for the primary endpoint reported, and if so, was it appropriate? Y, Y

Adjustment for multiplicity of statistical testing:

- Was an adjustment reported if there were more than 1 secondary endpoints statistically tested? N

Blinding:

- Were patients blinded from treatment? N
- Were assessing investigators blinded from patient treatment? N

Allocation concealment:

- Was allocation concealment carried out? N

Lost to follow-up/discontinued/withdrawn:

- 33/156 (21.2%) total
    - 17/82 (20.7%) withdrawn from Group 1
    - 16/74 (21.6%) withdrawn from Group 2

40. Gethin G, Cowman S. Manuka honey vs. hydrogel--a prospective, open label, multicentre, randomised controlled trial to compare desloughing efficacy and healing outcomes in venous ulcers. *J Clin Nurs.* 2009;18(3):466-474. **EXCLUDED FROM ANALYSIS DUE TO ARTICLE BEING RETRACTED BY JOURNAL IN 2015.**

41. Ghatnekar GS, Grek CL, Armstrong DG, Desai SC, Gourdie RG. The effect of a connexin43-based Peptide on the healing of chronic venous leg ulcers: a multicenter, randomized trial. *J Invest Dermatol.* 2015;135(1):289-298.

Study Groups: Group 1: connexin43 with ACT1 peptide + SOC; Group 2: SOC

Statistical results:

- ITT analysis done for primary endpoint(s): Y
- **Primary endpoint:** mean (SD) PAR at 12 weeks:
    - ITT: 79.3 (50.4) in Group 1 vs 36.3 (179.8) in Group 2
        - Difference between groups: 43%, p = .02
    - PP: 79.0 (51.1) in Group 1 vs 28.6 (189.2) in Group 2
        - Difference between groups: 50%, p = .01
- Incidence of 100% ulcer closure at 12 weeks, No. (%):
    - ITT: 26 (57%) in Group 1 vs 13 (28%) in Group 2, p = .01

For Peer Review

  - PP: 26 (74%) in Group 1 vs 10 (30%) in Group 2, p < .001
- Incidence of 50% ulcer closure at 12 weeks, No. (%):
  - ITT: 29 (63%) in Group 1 vs 20 (43%) in Group 2, p = .06
  - PP: 28 (8%) in Group 1 vs 17 (52%) in Group 2, p = .01
- Kaplan-Meier median weeks (90% CI) to 100% closure:
  - ITT: 6.0 (4.0-8.0) in Group 1 vs 12.1 (10.1-NA) in Group 2, p = .0006
  - PP: 6.0 (4.0-7.0) in Group 1 vs NA (12.0-NA) in Group 2, p < .0001
- Kaplan-Meier median weeks (90% CI) to 50% closure:
  - ITT: 2.9 (2.1-3.0) in Group 1 vs 6.9 (5.0-9.1) in Group 2, p <.001
  - PP: 2.9 (2.0-3.0) in Group 1 vs 8.0 (5.1-9.9) in Group 2, p < .001
- Cox HR (95% CI) for 100% closure:
  - ITT: 2.3 (1.0-5.0), p = .04
  - PP: 3.2 (1.4-7.3), p = .01
- There was no significance difference in the no. of responders center-wise between groups (ITT: p = .238; PP: p = .516, Cochran-Mantel-Haenszel test with Breslow-Day)
- Recurrence in 10 participants (5 in each group, 11% total)
  - Occurred during follow-up posttreatment in 2 participants in each group (4% total)
  - No significant difference in recurrence during/after study period between groups, p >1.00
- There was no statistical difference in mean (SD) intensity of pain during the study period between the groups
  - ITT: 0.46 (1.07) in Group 1 vs 0.34 (0.71) in Group 2, p = .839
  - PP: 0.18 (0.39) vs 0.48 (0.80), p = .119
- No. (%) of subjects with at least 1 AE in safety population, n = 92: 14 (30%) in Group 1 vs 14 (30%) in Group 2; 28 (30%) total, p > 1.00
  - Subjects reporting 1 AE: 8 (17%) vs 12 (26%); 20 (22%) total, p = .31
  - Subjects reporting >1 AE: 6 (13%) vs 2 (4%); 8 (9%) total, p = .27
  - Subjects reporting wound infection: 2 (4%, 2 AEs) vs 1 (2%, 2 AEs); 3 (3%, 4 AEs) total, p > 1.00
  - Subjects reporting wound complication: 7 (15%, 11 AEs) vs 5 (11%, 5 AEs); 12 (13%, 16 total), p = .54
  - Subjects reporting venous ulcer pain: 6 (13%, 6 AEs) vs 4 (9%, 4 AEs); 10 (11%, 10 AEs) total, p = .74
  - Total AEs in 28 subjects: 24 vs 16; 40 total, p = .21

Power calculation:

- Was a power calculation for the primary endpoint reported, and if so, was it appropriate? Y, Y

Adjustment for multiplicity of statistical testing:

- Was an adjustment reported if there were more than 1 secondary endpoints statistically tested? ?

Blinding:

- Were patients blinded from treatment? N

For Peer Review

- Were assessing investigators blinded from patient treatment? Y

Allocation concealment:

- Was allocation concealment carried out? N

Lost to follow-up/discontinued/withdrawn:

- 22/92 (23.9%) total
    - 12/46 (26.1%) withdrawn from Group 1
    - 10/46 (21.7%) withdrawn from Group 2

42. Gibbons GW, Orgill DP, Serena TE, et al. A prospective, randomized, controlled trial comparing the effects of noncontact, low-frequency ultrasound to standard care in healing venous leg ulcers. *Ostomy Wound Manage.* 2015;61(1):16-29.

Study Groups: Group 1: 40 kHz noncontact, low-frequency ultrasound + SOC (n = 41); Group 2: SOC (n = 40)

Statistical results:

- ITT analysis done for primary endpoint(s): Y
- **Primary endpoint:** Mean (SD, Median, IQR, Range) PAR at 4 weeks: Group 1: 61.6 (28.9, 65.7, 48.4-83.9, 2.0-99.2; Group 2: 45.0 (32.5, 44.4, 20.9-68.1, -23.6-100.0)
    - mean (median) p: .02 (.02); p adjusted for ulcer size and age: .01
- Mean (SD, Median, IQR, Range) absolute are reduction, cm^2, at 4 weeks: Group 1: 9.0 (9.0, 5.5, 3.3-11.3, 0.8-43.8; Group 2: 4.1 (4.1, 3.1, 1.5-6.2, -4.1-17.2)
    - mean (median) p: .003 (.01); p adjusted for ulcer size and age: .02
- Mean (SD) PAR in ulcers <6 months duration: Group 1: 79.4 (18.9); Group 2: 49.2 (29.3), p = .002
- Mean (SD) PAR in ulcers >6 months duration: Group 1: 37.6 (16.9); Group 2: 6.4 (36.4), p = .12
- Mean (SD) PAR in ulcers >10 cm^2: Group 1: 60.0 (32.2); Group 2: 31.9 (32.8), p = .01
- Mean (SD) PAR in ulcers <10 cm^2: Group differences were not significant, p = NS
- Negative correlation between mean PAR and ulcer size at randomization in Group 2, p = .03; no significant correlation observed in Group 1, p = NS
- Mean (SD, Median, IQR, Range) VAS score
    - At randomization: Group 1: 3.7 (3.3, 3.0, 0.8-6.1, 0.0-10.0); Group 2: 3.3 (2.6, 3.0, 0.6-5.2, 0.0-9.0); mean (median) p: .57 (.73)
    - At 4 weeks: Group 1: 2.0 (2.4, 0.6, 0.1-3.8, 0.0-9.1); Group 2: 3.4 (3.2, 2.4, 0.4-5.9, 0.0-10.0); mean (median) p: .03 (.04)
    - Reduction in VAS at 4 weeks: Group 1: 1.7 (3.0, 0.5, 0.0-2.9, -3.2-9.8); Group 2: 0.0 (2.3, 0.0, -0.8-1.3, -7.3-4.4); mean (median) p: .01 (.01)
- Among QOL scores, median bodily pain score was the only significant difference between groups: 45.9 in Group 1 vs 41.5 in Group 2, p = .04
- Subjects in Group 1 that had compression at a level of 30-40 mm Hg 30 days before enrollment resulted in a 67.0% median PAR vs 40.5% in Group 2, p = .0005
- Ulcers with a lateral location had a significantly higher median PAR of 81.3% in Group 1 vs 31.4% in Group 2, p = .01

Power calculation:

For Peer Review

- Was a power calculation for the primary endpoint reported, and if so, was it appropriate? Y, Y

Adjustment for multiplicity of statistical testing:

- Was an adjustment reported if there were more than 1 secondary endpoints statistically tested? N

Blinding:

- Were patients blinded from treatment? N
- Were assessing investigators blinded from patient treatment? Y

Allocation concealment:

- Was allocation concealment carried out? N

Lost to follow-up/discontinued/withdrawn:

- 7/81 (8.6%) *lost after primary endpoint analysis*

43. Gould DJ, Campbell S, Newton H, Duffelen P, Griffin M, Harding EF. Setopress vs Elastocrepe in chronic venous ulceration. *Br Journal Nurs.* 1998;7(2):66-70.

Study Groups: Group 1: long-stretch bandage; Group 2: short-stretch bandage

Statistical results:

- ITT analysis done for primary endpoint(s): N
- **There were no primary endpoints**
- No. ulcers that healed: 11 in Group 1 vs 7 in Group 2, p = .24
- No. ulcers that improved but did not heal: 6 in Group 1 vs 4 in Group 2, p = .34
- No. ulcers that deteriorated: 2 in Group 1 vs 9 in Group 2, p = .03
- No. of ulcers that healed or improved: 17 in Group 1 vs 11 in Group 2, p = .031
- Cochran-Armitage test shows a significantly positive trend in quality of ulcer outcome for Group 1 and a significantly negative trend in outcome for Group 2, p = .040
- Median time to heal combined with estimated healing time for ulcers that improved: 12 weeks in Group 1 vs 23 weeks in Group 2, p = .04

Power calculation:

- Was a power calculation for the primary endpoint reported, and if so, was it appropriate? N

Adjustment for multiplicity of statistical testing:

- Was an adjustment reported if there were more than 1 secondary endpoints statistically tested? N

Blinding:

- Were patients blinded from treatment? N
- Were assessing investigators blinded from patient treatment? Y

Allocation concealment:

- Was allocation concealment carried out? N

Lost to follow-up/discontinued/withdrawn:

- 7/39 patients with 46 ulcers (17.9%) total, leaving 32 patients with 39 ulcers (19 in Group 1 vs 20 in Group 2)

44. Guarnera G, DeRosa A, Camerini R. The effect of thymosin treatment of venous ulcers.

For Peer Review

Ann N Y Acad Sci. 2010;1194:207-212.

Study Groups: Group 1: Thymosin beta 4, 0.01% (n = 19); Group 2: Thymosin beta 4, 0.03% (n = 18); Thymosin beta 4, 0.1% (n = 18); Placebo (n = 17)

Statistical results:

- ITT analysis done for primary endpoint(s): Y.
- **There was no statistical analysis reported (no p values).**

Power calculation:

- Was a power calculation for the primary endpoint reported, and if so, was it appropriate? N

Adjustment for multiplicity of statistical testing:

- Was an adjustment reported if there were more than 1 secondary endpoints statistically tested? N

Blinding:

- Were patients blinded from treatment? Y
- Were assessing investigators blinded from patient treatment? Y

Allocation concealment:

- Was allocation concealment carried out? N

Lost to follow-up/discontinued/withdrawn: None reported.

45. Guest M, Smith JJ, Tripuraneni G, et al. Randomized clinical trial of varicose vein surgery with compression versus compression alone for the treatment of venous ulceration. *Phlebology.* 2003;18:130-136.

Study Groups: Group 1: Simple venous surgery + 4-layer bandaging (n =37); Group 2: 4-layer bandaging (n = 39)

Statistical results:

- ITT analysis done for primary endpoint(s): Y
- **There were no primary endpoints identified**
- Percent healed at 26 weeks: 68% (25/37) in Group 1 vs 64% (25/39) in Group 2, p = .75
- Median time to heal, days: 83 in Group 1 vs 98 in Group 2, long rank statistic = 0.69, p = .41
- HR from the Cox Regression was 0.80, 95% CI: 0.46-1.39, with slightly faster healing in Group 1, p = NS
    - After adjusting for ulcer duration, size, previous DVT, and no. of episodes of ulceration, there was still no significant difference in healing between the 2 groups: HR: 0.79, 95% CI: 0.45-1.39, p = NS
    - After adjusting for these factors in multivariate analysis, there was still no significant difference, p = NS
- Among those with deep venous incompetence, 65% healed (20/31) compared with 67% (30/45) with superficial incompetence, p = .84
- Among those with perforator incompetence, 63% healed (33/52), compared with 71% (17/24) of those without perforator incompetence, p = .47
- Mean SF36 and CXVUQ scores for Group 1
    - Physical functioning: 0 mos: 37.6; 3 mos: 42.2, p <.05; 6 mos: 44.8, p <.05

  - Role – physical: 0 mos: 38.1; 3 mos: 39.2, p = NS; 6 mos: 46.1, p <.05
  - Bodily pain: 0 mos: 45.5; 3 mos: 43.4, p = NS; 6 mos: 46.6, p = NS
  - General health: 0 mos: 41.9; 3 mos: 45.6, p = NS; 6 mos: 54.8, p <.05
  - Vitality: 0 mos: 59.9; 3 mos: 58.4, p = NS; 6 mos: 60.6, p = NS
  - Social functioning: 0 mos: 62.9; 3 mos: 64.1, p = NS; 6 mos: 62.1, p = NS
  - Role – emotional: 0 mos: 55.8; 3 mos: 55.3, p = NS; 6 mos: 54.8 , p = NS
  - Mental health: 0 mos: 71.2; 3 mos: 71.9, p = NS; 6 mos:72.3, p = NS
  - CXVUQ: 0 mos: 63.0; 3 mos: 50.2, p < .05; 6 mos: 41.1, p < .05
- Mean SF36 and CXVUQ scores for Group 2
  - Physical functioning: 0 mos: 36.7; 3 mos: 36.6, p = NS; 6 mos: 37.5, p = NS
  - Role – physical: 0 mos: 41.0; 3 mos: 43.0, p = NS; 6 mos: 48.8, p <.05
  - Bodily pain: 0 mos: 42.1; 3 mos: 45.1, p = NS; 6 mos: 54.1, p < .05
  - General health: 0 mos: 44.4; 3 mos: 43.7, p = NS; 6 mos: 45.9, p = NS
  - Vitality: 0 mos: 57.5; 3 mos: 57.7, p = NS; 6 mos: 58.4, p = NS
  - Social functioning: 0 mos: 63.2; 3 mos: 62.1, p = NS; 6 mos: 63.7, p = NS
  - Role – emotional: 0 mos: 53.9; 3 mos: 56.1, p = NS; 6 mos: 62.2, p < .05
  - Mental health: 0 mos: 68.8; 3 mos: 66.3, p = NS; 6 mos: 73.1, p = NS
  - CXVUQ: 0 mos: 60.4; 3 mos: 56.0, p = NS; 6 mos: 45.5, p < .05
  - The group differences in QOL scores were significant for physical functioning at 3 and 6 mos, bodily pain at 6 mos, general health at 6 mos, and role – emotional at 6 mos, p <.05
  - At 6 months, patients with healed ulcers had significantly better CXVUQ QOL than those with nonhealed ulcers in Group 1 (mean 34 vs 53, p <.05) and in Group 2 (mean 39 vs 56, p <.05)

Power calculation:
- Was a power calculation for the primary endpoint reported, and if so, was it appropriate? N

Adjustment for multiplicity of statistical testing:
- Was an adjustment reported if there were more than 1 secondary endpoints statistically tested? N

Blinding:
- Were patients blinded from treatment? N
- Were assessing investigators blinded from patient treatment? N

Allocation concealment:
- Was allocation concealment carried out? N

Lost to follow-up/discontinued/withdrawn: None reported.

46. Gupta AK, Filonenko N, Salansky N, Sauder DN. The use of low energy photon therapy (LEPT) in venous leg ulcers: a double-blind, placebo-controlled study. *Dermatol Surg.* 1998; 24(12):1383-1386.

Study Groups: Group 1: low energy photon therapy; Group 2: placebo

Statistical results:
- ITT analysis done for primary endpoint(s): N

For Peer Review

- **Primary: There were no primary endpoints identified, or, all 3 endpoints were primary.**
- Mean (SE) change in ulcer area compared with baseline
    - Week 3: 126.2 (51.2) in Group 1 vs -47.0 (12.1) in Group 2, p = .0003
    - Week 7: 185.2 (67.8) vs 23.3 (30.1), p = .0003
    - Week 10: 193.0 (70.4) vs 14.7 (51.2), p = .0002
- Mean (SE) percent of ulcer area that remains unhealed compared with baseline
    - Week 3: 45.2 (13.2) in Group 1 vs 124.0 (6.3) in Group 2, p = .005
    - Week 7: 27.0 (13.3) vs 83.4 (16.4), p = .04
    - Week 10: 24.3 (13.8) vs 84.7 (26.5), p = .008
- Mean (SE) rate of healing (mm^2/week)
    - Week 3: 42.1 (17.0) in Group 1 vs -15.7 (2.9) in Group 2, p = .024
    - Week 7: 26.4 (9.7) vs 3.3 (2.9), p = .056
    - Week 10: 19.3 (7.5) vs 1.5 (3.6), p = .055

Power calculation:
- Was a power calculation for the primary endpoint reported, and if so, was it appropriate? N

Adjustment for multiplicity of statistical testing:
- Was an adjustment reported if there were more than 1 secondary endpoints statistically tested? N

Blinding:
- Were patients blinded from treatment? Y
- Were assessing investigators blinded from patient treatment? Y

Allocation concealment:
- Was allocation concealment carried out? N

Lost to follow-up/discontinued/withdrawn: 1/9 (11.1%) total, drop-out from placebo group but no. of subjects and ulcers/group not reported. (Note there were 12 ulcers from 9 subjects enrolled).

47. Hammerle G, Strohal R. Efficacy and cost-effectiveness of octenidine wound gel in the treatment of chronic venous leg ulcers in comparison to modern wound dressings. *Int Wound J*. 2016;13(2):182-188.

Study Groups: Group 1: octenidine wound gel + modern wound-phase-adapted dressing (n = 17, 17 ulcers); Group 2: octenidine wound gel alone (n = 15, 15 ulcers); Group 3: modern wound-phase-adapted dressings alone (n = 15, 17 ulcers)

Statistical results:
- ITT analysis done for primary endpoint(s): Y
- **Primary endpoint:** Percent of granulation tissue (development) in the wound at day 42
    - Visit 0: 62 in Group 1 vs 52 in Group 2 vs 45 in group 3, p = NS
    - Visit 1: 63 in Group 1 vs 61 in Group 2 vs 53 in group 3, p = NS
    - Visit 2: 64 in Group 1 vs 73 in Group 2 vs 62 in group 3, p = NS
    - Visit 3: 76 in Group 1 vs 76 in Group 2 vs 68 in group 3, p = NS
    - Visit 4: 73 in Group 1 vs 84 in Group 2 vs 73 in group 3, p = NS

For Peer Review

    - Visit 5: 74 in Group 1 vs 96 in Group 2 vs 74 in group 3, p = NS
- Percentage of bioburden (reduction):
    - Visit 0: 35 in Group 1 vs 46 in Group 2 vs 55 in group 3, p = NS
    - Visit 1: 32 in Group 1 vs 37 in Group 2 vs 45 in group 3, p = NS
    - Visit 2: 27 in Group 1 vs 25 in Group 2 vs 33 in group 3, p = NS
    - Visit 3: 22 in Group 1 vs 22 in Group 2 vs 26 in group 3, p = NS
    - Visit 4: 23 in Group 1 vs 11 in Group 2 vs 24 in group 3, p = NS
    - Visit 5: 20 in Group 1 vs 2 in Group 2 vs 21 in group 3, p = NS
- Median (range) of wound area reduction, Visits 0; 1; 3; 5
    - 10.3 (1.3-91.5); 9.6 (1.0-91.4); 6.7 (0.2-27.1); 3.7 (0.0-11.7) in Group 1, n = 15
        - Percentage of reduction V0-V5: 64.1
    - 5.3 (0.8-24.9); 3.6 (0.8-22.8); 2.3 (0.0-29.6); 0.2 (0.0-17.7) in Group 2, n = 14
        - Percentage of reduction V0-V5: 96.2
    - 4.1 (1.3-76.2); 4.1 (1.3-74.5); 4.1 (0.8-71.1); 3.5 (0.3-63.4) in Group 3, n = 15 (V 0, 3, 5), n = 14 (V1)
        - Percentage of reduction V0-V5: 14.6
    - Group 1 vs Group 2, p = .845; Group 1 vs Group 3, p = .028; Group 2 vs Group 3, p = .028
- Frequency of infected wounds did not differ significantly between Group 1 and Group 2 vs group 3, p = .117 or p= .213, respectively
    - Significant differences in Group 1 vs Group 2, p = .038
- Patients perception of Group 1 and Group 2 was significant better vs Group 3, p = .001
    - Pleasantly cooling effect between group 1 and Group 2 was perceived positively without difference, p = .641

Power calculation:
Was a power calculation for the primary endpoint reported, and if so, was it appropriate? Y, N
Adjustment for multiplicity of statistical testing:
- Was an adjustment reported if there were more than 1 secondary endpoints statistically tested? N

Blinding:
- Were patients blinded from treatment? N
- Were assessing investigators blinded from patient treatment? N

Allocation concealment:
- Was allocation concealment carried out? N

Lost to follow-up/discontinued/withdrawn: None reported.

48. Hansson C. The effects of cadexomer iodine paste in the treatment of venous leg ulcers compared with hydrocolloid dressing and paraffin gauze dressing. Cadexomer Iodine Study Group. *Int J Dermatol.* 1998;37(5):390-396.

Study Groups: Group 1: Cadexomer iodine paste; Group 2: hydrocolloid; Group 3: paraffin gauze
Statistical results:
- ITT analysis done for primary endpoint(s): N

For Peer Review

- **Primary endpoint:** Mean (SD) PAR at endpoint: 61.6 (36.9) in Group 1 vs 40.7 (56.5) in Group 2 vs 23.9 (97.4) in Group 3, p = NS
    - At 12 weeks: 66.1 (25.4) in Group 1 vs 17.9 (51.6) in Group 2 vs 50.9 (53.2) in Group 3
        - P value between Group 1 and Group 2 = .0127; other group differences: p = NS
    - At 8 weeks: 60.0 (29.3) in Group 1 vs 29.3 (62.9) in Group 2 vs 9.7 (103.9) in Group 3, p = NS
    - At 4 weeks: 35.5 (40.3) in Group 1 vs 34.4 (47.7) in Group 2 vs 10.6 (80.4) in Group 3, p = NS
- There was no significant group difference in median time (days) to cease exudation: 55 in Group 1; 63 in Group 2; 85 in Group 3; p = .072
- Mean (SD; median) ulcer area reduction in cm^2 and in %, per week:
    - Group 1: 0.64 (1.01; 0.39) and 9 (8; 7.5)
    - Group 2: 0.97 (3.23; 0.35) and 8 (10; 7.9)
    - Group 3: 0.19 (0.32; 0.32) and 3 (14; 6.4)
    - Only Group 1 and Group 2 had a significant difference, p = .0353
- Percent of ulcers with slough; only Group 1 and Group 3 had significant differences at only Week 4 and Week 8, p < .05
    - Week 0: Group 1: 93; Group 2: 98; Group 3: 96
    - Week 4: 63; 64; 84
    - Week 8: 35; 59; 75
    - Week 12: 53; 86; 60
- Percent of ulcers with pain; there were no significant group differences, p = NS
    - Week 0: Group 1: 66; Group 2: 73; Group 3: 57
    - Week 4: Group 1: 59; Group 2: 50; Group 3: 47
    - Week 8: 19; 48; 19
    - Week 12: 29; 57; 15
- Percent of ulcers with granulation tissue; there were no significant group differences, p = NS
    - Week 0: Group 1: 66; Group 2: 75; Group 3: 71
    - Week 4: Group 1: 88; Group 2: 93; Group 3: 73
    - Week 8: 87; 74; 88
    - Week 12: 88; 93; 80
- Percent of ulcers with epithelialization; there were no significant group differences, p = NS
    - Week 0: Group 1: 21; Group 2: 21; Group 3: 20
    - Week 4: Group 1: 59; Group 2: 73; Group 3: 64
    - Week 8: 74; 74; 69
    - Week 12: 76; 86; 80

Power calculation:

- Was a power calculation for the primary endpoint reported, and if so, was it appropriate? N

For Peer Review

Adjustment for multiplicity of statistical testing:

- Was an adjustment reported if there were more than 1 secondary endpoints statistically tested? N

Blinding:

- Were patients blinded from treatment? N
- Were assessing investigators blinded from patient treatment? N

Allocation concealment:

- Was allocation concealment carried out? N

Lost to follow-up/discontinued/withdrawn:

- 45/153 (29.4%) total, but 28/153 (18.3%) excluded from analysis

49. Harding K, Gottrup F, Jawien A, et al. A prospective, multi-centre, randomised, open label, parallel, comparative study to evaluate effects of AQUACEL(R) Ag and Urgotul(R) Silver dressing on healing of chronic venous leg ulcers. *Int Wound J*. 2012;9(3):285-294.

Study Groups: Group 1: AQUACEL Ag silver dressing; Group 2: Urgotol Silver dressing

Statistical results:

- ITT analysis done for primary endpoint(s): Y
- **Primary endpoint:** Mean (SD) wound area RR at week 4: 38.2% (40.6%) in Group 1 vs 32.5% (48.9%) in Group 2, p = NS
  - Mean (SD; SE; range; 95% CI) ITT reduction in area: 49.6 (52.5; 4.4; -221.3 to 100; 40.9-53.4) in Group 1 vs 42.8 (60.0; 5.2; -217.7 to 100, 32.5-53.1) in Group 2
    - Group difference: 68.4 (56.3; 6.8; -6.56-20.2), p = .3158
  - Mean (SD; SE; range; 95% CI) PP reduction in area: 51.7 (52.9; 4.9; -212.3 to 100; 41.9-61.5) in Group 1 vs 45.1 (63.9; 6.3; -217.7 to 100, 32.6-57.5) in Group 2
    - Group difference: 6.6 (58.4; 7.9; -9.1-22.4), p = .406
- Mean (SD) wound area absolute reduction at week 8, cm^2: 8.8 (12.8) in Group 1 vs 7.2 (9.5) in Group 2, p = .2537
- No (%) with complete wound healing: 97/145 (66.9%) in Group 1 vs 69/133 (51.9%) in Group 2, p = .0108
- Mean (SD) change in overall wound closure rate at week 8: 0.17 (0.43) in Group 1 vs 0.14 (0.19) in Group 2, p = .4380
- % with ulcers that decreased by ≥40%: 66.2% in Group 1 vs 58.5% in Group 2, p = .1842
- By logistic regression, treatment group, age, BMI, and ulcer size at baseline were not significant healing factors (p = NS)
  - Duration of ulcer in years was a significant predictive factor (OR: 0.55, p = .0076; 95% CI: 0.35-0.85)
- % with clinical signs of heavy bacterial colonization at week 8: 39.5% in Group 1 vs 32.5% in Group 2, p = NS
- There was no significant difference in overall improvement of ulcers between groups, p = .0889

Power calculation:

- Was a power calculation for the primary endpoint reported, and if so, was it appropriate? Y, Y

For Peer Review

Adjustment for multiplicity of statistical testing:

- Was an adjustment reported if there were more than 1 secondary endpoints statistically tested? N

Blinding:

- Were patients blinded from treatment? N
- Were assessing investigators blinded from patient treatment? N

Allocation concealment:

- Was allocation concealment carried out? N

Lost to follow-up/discontinued/withdrawn:

- 27/281 (9.6%) total
  - 11/145 (7.6%) withdrawn from Group 1
  - 16/136 (11.7%) withdrawn from Group 2

50. Harding K, Sumner M, Cardinal M. A prospective, multicentre, randomised controlled study of human fibroblast-derived dermal substitute (Dermagraft) in patients with venous leg ulcers. *Int Wound J.* 2013;10(2):132-137.

Study Groups: Group 1: human fibroblast-derived dermal substitute + 4-layer compression therapy; Group 2: 4-layer compression therapy

Statistical results:

- ITT analysis done for primary endpoint(s): Y
- **Primary endpoint:** proportion completely healed at 12 weeks: 34% (64/186) in Group 1 vs 31% (56/180) in Group 2, p = .235, OR = 1.40, 95% CI: 0.80-2.41
  - Treatment by duration observed for Group 1: Group 2: OR = 0.99, 95% CI: 0.98-1.00
  - Proportion healed in ulcer durations ≤12 mos: 52% (49/94) in Group 1 vs 37% (36/97), p = .029, OR: 2.37, 95% CI: 1.08-5.14
  - Proportion healed with ulcers ≤10 cm^2, 47% (55/117) in Group 2 vs 39% (47/120), p = .223
- No significant group difference in time to heal: p = .660, HR = 1.07, 95% CI: 0.80-1.43
- % (No) of patients reporting AEs: 78% (146/187) in Group 1 vs 77% (138/179) in Group 2, p = .900
- There were no significant group differences in the no. of cases of wound infection, cellulitis, and skin ulcer, p = NS
  - % (no) of wound infection: 29.4% (55/187) in Group 1 vs 24.0% (43/179) in Group 2, p = NS
  - % (no) of study site infection: 23% (43/187) vs 26% (46/179), p = .62
  - % (no) of cellulitis: 6.4 (12/187) vs 10.1 (18/179)

Power calculation:

- Was a power calculation for the primary endpoint reported, and if so, was it appropriate? Y, Y

Adjustment for multiplicity of statistical testing:

- Was an adjustment reported if there were more than 1 secondary endpoints statistically tested? N

For Peer Review

Blinding:

- Were patients blinded from treatment? N
- Were assessing investigators blinded from patient treatment? N

Allocation concealment:

- Was allocation concealment carried out? N

Lost to follow-up/discontinued/withdrawn:

- 60/366 (16.4%) total
  - 19/186 (10%) withdrawn from Group 1
  - 41/180 (23%) withdrawn from Group 2

51. Harding KG, Krieg T, Eming SA, et al. Efficacy and safety of the freeze-dried cultured human keratinocyte lysate, LyphoDerm 0.9%, in the treatment of hard-to-heal venous leg ulcers. *Wound Repair Regen*. 2005;13(2):138-147.

Study Groups: Group 1: SOC + freeze-dried cultured human keratinocyte lysate, 0.9%; Group 2: SOC + Vehicle; Group 3: SOC without Vehicle

Statistical results:

- ITT analysis done for primary endpoint(s): N.
- **Primary endpoint:** Percentage of patients with complete healing within 24 weeks was 37% in Group 1 compared to 27% in Groups 2 and 3 (p = .137)
- Baseline ulcer area was highly significant for healing (p <.0001) and ulcer duration was almost significant (p = .054).
- Change in ulcer margin between screening and baseline visit (p =.001) and BMI (p =, .019 were also significant for healing
- Complete ulcer healing within 24 weeks: Group 1: 36/98 (37%), Group 2: 13/50 (26%), Group 3: 13/45 (29%), Groups 2 and 3: 26/95 (27%), p = .287
  - Group 2 vs 3: p = .626
  - Group 1 vs Groups 2 and 3: p = .137
- Enlarging ulcer that healed within 24 weeks: Group 1: 13/42 (31%); Group 2: 1/16 (6%); Group 3: 2/17 (12%); Groups 2 + 3: 3/33 (9%), p = . 014
  - Group 2 vs 3: p = .429
  - Group 1 vs Groups 2 + 3: p = .005
- Enlarging ulcer and area ≤5 cm^2 that healed within 24 weeks: Group 1: 8/14 (57%); Group 2: 0/6 (0%); Group 3: 1/5 (20%); Groups 2 and 3: 1/11 (9%), p = .023
  - Group 2 vs 3: p = .232
  - Group 1 vs Groups 2 + 3: p = .008
- Shrinking ulcer that healed within 24 weeks: Group 1: 23/56 (41%); Group 2: 12/34 (35%); Group 3: 11/28 (39%), Groups 2 and 3: 23/62 (37%), p = .987
  - Group 2 vs 3: p = .915
  - Group 1 vs Groups 2 + 3: p = .906
- Shrinking ulcer and area ≤5 cm^2 that healed within 24 weeks: Group 1: 18/35 (51%); Group 2: 8/18 (44%); Group 3: 9/15 (60%), Groups 2 and 3: 17/33 (52%), p = .354
  - Group 2 vs 3: p = .174
  - Group 1 vs Groups 2 + 3: p = .634

For Peer Review

- Complete ulcer healing within 12 weeks: Group 1: 27/98 (28%), Group 2: 10/50 (20%), Group 3: 12/45 (27%), Groups 2 and 3: 22/95 (23%), p = .467
  - Group 2 vs 3: p = .327
  - Group 1 vs Groups 2 and 3: p = .464
- Enlarging ulcer that healed within 12 weeks: Group 1: 10/42 (24%); Group 2: 1/16 (6%); Group 3: 2/17 (12%); Groups 2 + 3: 3/33 (9%), p = . 089
  - Group 2 vs 3: p = .451
  - Group 1 vs Groups 2 + 3: p = .037
- Enlarging ulcer and area ≤5 cm^2 that healed within 12 weeks: Group 1: 6/14 (43%); Group 2: 0/6 (0%); Group 3: 1/5 (20%); Groups 2 and 3: 1/11 (9%), p = .109
  - Group 2 vs 3: p = .241
  - Group 1 vs Groups 2 + 3: p = .046
- Shrinking ulcer that healed within 12 weeks: Group 1: 17/56 (30%); Group 2: 9/34 (26%); Group 3: 10/28 (36%), Groups 2 and 3: 19/62 (31%), p = .751
  - Group 2 vs 3: p = .502
  - Group 1 vs Groups 2 + 3: p = .717
- Shrinking ulcer and area ≤5 cm^2 that healed within 12 weeks: Group 1: 14/35 (40%); Group 2: 6/18 (33%); Group 3: 8/15 (53%), Groups 2 and 3: 14/33 (42%), p = .328
  - Group 2 vs 3: p = .137
  - Group 1 vs Groups 2 + 3: p = .909
- Mean (SE) time to complete ulcer healing was 139.7 (5.6) days for Group 1 vs 148.5 (5.6) days for Groups 2 and 3 (p =.199)
- Proportion of subjects without ulcer recurrence at trial end was 77.8% in Group 1 vs 80.8% in Groups 2 and 3 (p = .780).
- Ulcer area decreased with a median of 58% in all groups, while the ulcer perimeter decreased with a median 36% and 38% in Groups 2 and 3 and in Group 1, respectively (p >.05)
- Physical examinations and vital signs did not reveal any significant changes (p = NS)
- Subjects of Group 3 lost 1 kg compared to baseline (p < .05)
- Pain decreased in all groups without significant difference (from 5 to 4 mm in Group 1, from 7 to 1 mm in Group 2, and from 12 to 4 mm in Group 3, p = NS)
- Clinical evaluation of the study ulcer revealed no significant differences (p = NS)

Power calculation:

- Was a power calculation for the primary endpoint reported, and if so, was it appropriate? Y, Y

Adjustment for multiplicity of statistical testing:

- Was an adjustment reported if there were more than 1 secondary endpoints statistically tested? N

Blinding:

- Were patients blinded from treatment? N
- Were assessing investigators blinded from patient treatment? N

Allocation concealment:

- Was allocation concealment carried out? N

For Peer Review

Lost to follow-up/discontinued/withdrawn:

- 16/194 (8.2%) total

52. Harding KG, Vanscheidt W, Partsch H, Caprini JA, Comerota AJ. Adaptive compression therapy for venous leg ulcers: a clinically effective, patient-centred approach. *In Wound J*. 2016;13(3):317-325.

Study Groups: Group 1: Adaptive compression therapy; Group 2: 4-layer bandaging

Statistical results:

- ITT analysis done for primary endpoint(s): Y
- **Primary endpoint:** No. healed ulcers: 12 (32%) in Group 1 vs 22 (42%) in Group 2, p = .30
- Time to healing: 64.0 days in Group 1 vs 48.5 days in Group 2, p = .85
- Mean PAR was 50.2% for Group 1 and 65.2% for Group 2, p = .06
- Final EQ-5D-3L scores predicted to be 0.1025 higher for Group 1 than for Group 2, p = .0375
  - Patients with healed ulcers had a score 0.1774 higher than patients with unhealed ulcers, p = .0004
- Baseline mean (SD) pain ulcer score was significantly higher in Group 1 (4.6 [2.8]) than Group 2 (3.2 [2.2]), p = .0124.
  - Adjusting for baseline, there was no difference in the predicted final pain score among groups, difference = 0.2325, p = .6755.
- Mean (SD) patient ratings of treatment performance
  - Exudate management: 4.2 (3.4-5) in Group 1 vs 3.8 (3-4.6) in Group 2, p = .0422
  - Skin protection: 3.9 (2.9-4.95) vs 3.3 (2.2-4.4), p = .0091
  - Application ease: 4.1 (3.4-4.9) vs 3.9 (3.25-4.6), p = NS
  - Removal ease: 4.4 (3.7-5) vs 4.0 (3.5-4.6), p = .0007
  - Overall ease of use: 3.9 (2.8-4.9) vs 3.8 (3-4.6), p = NS
  - Showering and bathing: 4.5 (3.65-5) vs 2.3 (1.4-3.25), p < .0001
  - Sleep comfort: 4.0 (3-4.9) vs 3.5 (2.75-4.4), p = .0405
  - Discreteness under clothes: 3.4 (2.5-4.4) vs 3.5 (2.6-4.2), p = NS

Power calculation:

- Was a power calculation for the primary endpoint reported, and if so, was it appropriate? N

Adjustment for multiplicity of statistical testing:

- Was an adjustment reported if there were more than 1 secondary endpoints statistically tested? N

Blinding:

- Were patients blinded from treatment? N
- Were assessing investigators blinded from patient treatment? N

Allocation concealment:

- Was allocation concealment carried out? N

Lost to follow-up/discontinued/withdrawn:

- 17/90 (18.9%) total
  - 12/38 (31.6%) withdrawn from Group 1

For Peer Review

- 5/52 (9.6%) withdrawn from Group 2

53. Harrison MB, Vandenkerkhof EG, Hopman WM, Graham ID, Carley ME, Nelson EA. The Canadian Bandaging Trial: Evidence-informed leg ulcer care and the effectiveness of two compression technologies. *BMC Nurs.* 2011;10:20.

Study Groups: Group 1: Cotton short-stretch bandaging; Group 2: 4-layer component bandage system (control)

Statistical results:

- ITT analysis done for primary endpoint(s): Y
- **Primary endpoint:** Median time to heal: 77 days (95% CI: 63-91) in Group 1 vs 62 days (95% CI: 51-73) in Group 2 (log rank chi-squared = .001; p = .98).
- Center treatment effect on time to healing, bivariate analysis
  - Grouping 1: p <.01; HR: 1.0
  - Grouping 2: p <.01; HR (CI): 2.2 (1.6-3.1)
  - Grouping 3: p <.01; HR (CI): 2.8 (2.0-4.0)
  - Grouping 4: p < .01: HR (CI): 2.6 (1.8-3.7)
  - Grouping 5: p < .01: HR (CI): 4.2 (3.2-5.6)
- Center treatment effect on time to healing, multivariate analysis
  - Grouping 1: p <.01; HR: 1.0
  - Grouping 2: p <.01; HR (CI): 1.8 (1.2-2.6)
  - Grouping 3: p <.01; HR (CI): 2.3 (1.6-3.4)
  - Grouping 4: p < .01: HR (CI): 2.5 (1.7-3.7)
  - Grouping 5: p < .01: HR (CI): 3.7 (2.7-5.0)
- Gender treatment effect on time to healing, bivariate analysis
  - Male: No p value; HR: 1.0
  - Female: p = .34, HR (CI): 1.1 (0.90-1.4)
- Gender treatment effect on time to healing, multivariate analysis
  - Male: No p value; HR: 1.0
  - Female: p = .26, HR (CI): 1.1 (0.91-1.4)
- Age (decades) treatment effect on time to healing, bivariate analysis: p < .01; HR (CI): 1.1 (1.1-1.2)
- Age (decades) treatment effect on time to healing, multivariate analysis: p = .04; HR (CI): 1.1 (1.0-1.1)
- Living situation treatment effect on time to healing, bivariate analysis
  - Alone: No p value, HR: 1.0
  - With others: p = .09, HR (CI): 1.2 (0.97-1.5)
- Living situation treatment effect on time to healing, multivariate analysis
  - Alone: No p value, HR: 1.0
  - With others: p = .08, HR (CI): 1.2 (0.98-1.5)
- Comorbidities treatment effect on time to healing, bivariate analysis (for multivariate analysis, variable not selected for Cox regression final model)
  - None: p < .01, HR: 1.0
  - 1-2: p = .06, HR (CI): 1.3 (1.0-1.7)

For Peer Review

    - >3: p < .01, HR (CI): 2.2 (1.5-1.3)
- Pain on admission treatment effect on time to healing, bivariate analysis (for multivariate analysis, variable not selected for Cox regression final model)
    - Y: no p value, HR: 1.0
    - N: p = .02, HR (CI): 1.5 (1.1-2.1)
- Mobility treatment effect on time to healing, bivariate analysis (for multivariate analysis, variable not selected for Cox regression final model)
    - Fully mobile: no p value; HR: 1.0
    - Walks with assistance/immobile: p = .02, HR (CI): 1.4 (1.1-1.7)
- Ankle flexion treatment effect on time to healing, bivariate analysis (for multivariate analysis, variable not selected for Cox regression final model)
    - Full flexion: no p value, HR: 1.0
    - Impaired/no flexion: p = .10; HR: 1.3 (0.96-1.6)
- Edema treatment effect on time to healing, bivariate analysis (for multivariate analysis, variable not entered into Cox regression final model, p > .15)
    - Y: no p value, HR: 1.0
    - N: p = .86, HR: 1.0 (.77-1.4)
- ABPI treatment effect on time to healing, bivariate analysis (for multivariate analysis, variable not selected for Cox regression final model): p < .01, HR (CI): 5.7 (3.0-10.9)
- Previous ulcer treatment effect on time to healing, bivariate analysis (for multivariate analysis, variable not entered into Cox regression final model, p > .15)
    - Y: no p value, HR : 1.0
    - N: p = .64, HR (CI): 1.0 (0.86-1.3)
- Ulcer duration treatment effect on time to healing, bivariate analysis (for multivariate analysis, variable not selected for Cox regression final model)
    - ≤12 weeks: p < .01, HR (CI): 1.8 (1.5-2.2)
    - >12 weeks: no p value, HR: 1.0
- Ulcer size treatment effect on time to healing, bivariate analysis
    - ≤2.5 cm: p < .01, HR (CI): 2.7 (2.1-3.5)
    - >2.5 to ≤10 cm: p <.01, HR (CI): 1.6 (1.2-2.0)
    - >10 cm: p < .01, HR: 1.0
- Ulcer size treatment effect on time to healing, multivariate analysis
    - ≤2.5 cm: p < .01, HR (CI): 2.1 (1.5-2.7)
    - >2.5 to ≤10 cm: p <.01, HR (CI): 1.5 (1.1-2.0)
    - >10 cm: p < .01, HR: 1.0
- Allocated bandage treatment effect on time to healing, bivariate analysis
    - 4-layer: no p value, HR: 1.0
    - Short-stretch: p = .99, HR (CI): 1.0 (0.82-1.2)
- Allocated bandage treatment effect on time to healing, multivariate analysis
    - 4-layer: p = .77; HR: 1.0
    - Short-stretch: no p value, HR (CI): 1.0 (0.84-1.3)
- Proportion of people with a recurring ulcer at 1-year was 13% for Group 1 and 10% for Group 2 (p = NS)

For Peer Review

- At 3 months, there were no differences in pain with 26.7% in Group 1 having no pain vs 22.7% in Group 2; p = .335
- At 3 months, there were no differences in HRQL outcomes for SF-12 MCS: 55.8 reported in Group 1 vs 55.1 in Group 2 (p =.615) and for SF-12 PCS: 39.6 in Group 1 vs 39.0 in Group 2 (p =.675)
- 33% in Group 1 had an AE vs 29% in Group 2, p = NS
- AEs related to compression bandages (max. 30 mos):
    - Pressure damage: 13 (6.2%) in Group 1 vs 15 (7.0%) in Group 2, p = .85
    - Skin breakdown: 34 (16.3%) in Group 1 vs 27 (12.6%) in Group 2, p = .33
    - Ulcer deterioration: 32 (15.3%) in Group 1 vs 27 (12.6%) in Group 2, p = .48
    - Infection: 35 (16.7%) in Group 1 vs 28 (13.0%) in Group 2, p = .34
    - New ulcer: 22 (10.5%) in Group 1 vs 13 (6.0%) in Group 2, p = .11
    - Allergy/dermatitis related to bandaging: 14 (6.7%) in Group 1 vs 15 (7.0%) in Group 2, p = 1.00
    - Allergy/dermatitis related to ointment/cream: 10 (4.8%) in Group 1 vs 9 (4.2%) in Group 2, p = .82
    - Recurrence: 5 (2.4%) in Group 1 vs 2 (0.9%) in Group 2, p = .28
    - Limb compromise: 2 (1.0%) in Group 1 vs 2 (0.9%) in Group 2, p = 1.00
- Significantly greater discomfort with 4-layer bandaging (p =.05) and Group 2 felt it was applied to tight (p <.01)
- Patient-reported problems with bandaging at 1 month
    - Skin reactions: 56/199 (28.1%) in Group 1 vs 81/196 (41.3%) in Group 2, p = .01
    - Discomfort: 9/199 (4.5%) in Group 1 vs 16/196 (8.2%) in Group 2, p = .15
    - Skin breakdown: 33/199 (16.6%) in Group 1 vs 49/196 (25.0%) in Group 2, p = .05
    - Applied too tight: 10/199 (5%) in Group 1 vs 12/196 (6.1%) in Group 2, p = .67
    - Applied too loose: 10/199 (5%) in Group 1 vs 35/196 (17.9%) in Group 2, p = <.01
- Patient satisfaction with nurses' skills applying the bandage
    - Very satisfied: 171/199 (85.9%) in Group 1 vs 154/195 (79.0%) in Group 2, p = .10
    - Quite satisfied: 25/199 (12.6%) in Group 1 vs 40/195 (20.5%) in Group 2, no p
    - Neither satisfied or dissatisfied: 1/199 (0.5%) in Group 1 vs 1/195 (0.5%) in Group 2, no p
    - Quite dissatisfied: 2/199 (1%) in Group 1 vs 0/195 (0.0%) in Group 2, no p
- Bandage comfort
    - I have to take them off because they are too uncomfortable: 8/199 (4.0%) in Group 1 vs 13/195 (6.7%) in Group 2, p = .18
    - I wear them but they are very uncomfortable: 2/199 (1%) in Group 1 vs 6/195 (3.1%) in Group 2, no p
    - I wear them but they are slightly uncomfortable: 41/199 (20.6%) in Group 1 vs 47/195 (24.1%) in Group 2, no p
    - I have no trouble wearing them: 148/199 (74.4%) in Group 1 vs 129/195 (66.2%) in Group 2, no p
- The nurse gave information about how to care for leg ulcer: 178/199 (89.4%) in Group 1 vs 180/195 (92.3%) in Group 2, p = .38

For Peer Review

- The nurse talked about preventing recurrence: 164/198 (82.8%) in Group 1 vs 161/196 (82.1%) in Group 2, p = .90
    - The nurse gave written information about preventing recurrence: 113/196 (57.7%) in Group 1 vs 114/196 (58.2%) in Group 2, p = 1.00

Power calculation:

- Was a power calculation for the primary endpoint reported, and if so, was it appropriate? Y, Y

Adjustment for multiplicity of statistical testing:

- Was an adjustment reported if there were more than 1 secondary endpoints statistically tested? N

Blinding:

- Were patients blinded from treatment? N
- Were assessing investigators blinded from patient treatment? N

Allocation concealment:

- Was allocation concealment carried out? Y

Lost to follow-up/discontinued/withdrawn:

- 134/424 (31.6%) total
    - 54/209 (25.8%) withdrawn from Group 1
    - 80/215 (23.3%) withdrawn from Group 2

54. Hjerppe A, Saarinen JP, Venermo MA, Huhtala HS, Vaalasti A. Prolonged healing of venous leg ulcers: the role of venous reflux, ulcer characteristics and mobility. *J Wound Care*. 2010;19(11):474, 6, 8 passim.

Study Groups: Group 1: healed VLUs assessed with color-flow duplex imaging following SOC (n = 28); Group 2: unhealed VLUs assessed with color-flow duplex imaging following SOC (n = 28)

Statistical results:

- ITT analysis done for primary endpoint(s): Y
- **There were no primary endpoints identified**
- Reflux profiles: Significant differences between Group 1 (healed, n = 28) and Group 2 (hard to heal, n = 22), p = .00
    - Group 1: 5 (17.1%) walker or walker stick; 23 (82.1%) no utility in use
    - Group 2: 8 (36.3%) walker or walker stick; 14 (63.6%) no utility in use
- Wound size at baseline was associated with poor healing. The baseline mean (range) of wound size, cm^2: 5 (1 - 80) in Group 1 vs 11.2 (1 - 31) in Group 2, p = .41
- Mean (range) of pre-assessment ulcer duration, months: 7 (2 - 48) in Group 1 vs 26 (8 - 106) in Group 2, p = .001

Power calculation:

- Was a power calculation for the primary endpoint reported, and if so, was it appropriate? N

Adjustment for multiplicity of statistical testing:

- Was an adjustment reported if there were more than 1 secondary endpoints statistically tested? N

Blinding:

For Peer Review

- Were patients blinded from treatment? N
- Were assessing investigators blinded from patient treatment? N

Allocation concealment:

- Was allocation concealment carried out? N

Lost to follow-up/discontinued/withdrawn:

- 110 were enrolled and 20 withdrawn before treatment (18.1%)
- 40 additional healed and unhealed ulcers were then excluded from analysis
- 60/110 (54.5%) total

55. Hokkam E, El-Labban G, Shams M, Rifaat S, El-Mezaien M. The use of topical phenytoin for healing of chronic venous ulcerations. *Int J Surg.* 2011;9(4):335-338.

Study Groups: Group 1: topical phenytoin (n = 54); Group 2: normal saline placebo (control) (n = 50)

Statistical results:

- ITT analysis done for primary endpoint(s): Y
- **Primary endpoint:** complete healing in 35 (64.8%) in Group 1 vs 26 (52%) in Group 2, p = .04

Power calculation:

- Was a power calculation for the primary endpoint reported, and if so, was it appropriate? N

Adjustment for multiplicity of statistical testing:

- Was an adjustment reported if there were more than 1 secondary endpoints statistically tested? N

Blinding:

- Were patients blinded from treatment? N
- Were assessing investigators blinded from patient treatment? N

Allocation concealment:

- Was allocation concealment carried out? N

Lost to follow-up/discontinued/withdrawn: None reported.

56. Humbert P, Faivre B, Veran Y, et al. Protease-modulating polyacrylate-based hydrogel stimulates wound bed preparation in venous leg ulcers--a randomized controlled trial. *J Eur Acad Dermatol Venereol.* 2014;28(12):1742-1750.

Study Groups: Group 1: polyacrylic acid-containing dressing; Group 2: amorphous hydrogel

Statistical results:

- ITT analysis done for primary endpoint(s): Y.
- **Primary endpoint:** The proportion of ulcer area covered by slough and necroses had a mean (SD) percent reduction of 37.6 (29.9) in Group 1 vs 16.8 (23.0) at day 14, p = .004.
  - =relative decrease of 43.4 (36.7) in Group 1 vs 21.9 (39.4) in Group 2, p = .018
  - =relative change in % (CI) of 41.9 (28.6-55.1) in Group 1 vs 23.2 (11.0-35.4) in Group 2, p = .046
  - On day 14, 17 (50%) of Group 1 had <50% of their surface covered by slough vs 11 (26.8%) in Group 2, p = .0389

For Peer Review

    - The probability of reducing the amount of fibrin and necrotic tissue to <50% of the wound area was 2.7 times higher for Group 1 (OR = 2.72; 95% CI: 1.31-9.66)
    - Among hard-to-heal ulcers, 12/22 (54%) in Group 1 had <50 surface coverage by fibrin and necrotic tissue vs 7/30 (23.3%) in Group 2, p = .0209
- The proportion of ulcer are covered by granulation tissue increased by 36.0% (27.4) in Group 1 and 14.5 (22.0) in Group 2, p = .005
    - 16 (47.1%) in Group 1 had >50% granulation surface coverage vs 9 (23.1%) in Group 2, p = .0217
    - The probability of having >50% covered with granulation tissue on day 14 was 3 times higher for Group 1 (OR = 3.16, 95% CI: 1.16-8.59).
    - Among hard-to-heal ulcers: 11/22 (50%) in Group 1 had >50% granulation surface coverage vs 6/30 (20.0%) in Group 2, p = .0227.

Power calculation:

- Was a power calculation for the primary endpoint reported, and if so, was it appropriate? Y, N

Adjustment for multiplicity of statistical testing:

- Was an adjustment reported if there were more than 1 secondary endpoints statistically tested? N

Blinding:

- Were patients blinded from treatment? N
- Were assessing investigators blinded from patient treatment? Y

Allocation concealment:

- Was allocation concealment carried out? Y

Lost to follow-up/discontinued/withdrawn:

- 4/75 (5.3%) total
    - 2/34 (5.9%) withdrawn from Group 1
    - 2/41 (4.9%) withdrawn from Group 2

57. Jull A, Wadham A, Bullen C, Parag V, Kerse N, Waters J. Low dose aspirin as adjuvant treatment for venous leg ulceration: pragmatic, randomised, double blind, placebo controlled trial (Aspirin4VLU). *BMJ.* 2017;359:j5157.

Study Groups: Group 1: 150 mg aspirin with calcium lactate as a bulking agent; Group 2: placebo bulking agent

Statistical results:

- ITT analysis done for primary endpoint(s): Y
- **Primary endpoint:** Median Time to healing: 77 days in Group 1 vs 69 in Group 2, p = .089, chi sq = 1.53, P = .22, HR = 0.85 (95% CI: 0.64 to 1.13, P = .25)
    - Adjusted for center prognostic index, and covariate imbalances (age and first ulcer: HR = 0.88, 95% CI = 0.65 to 1.19, P = .39
    - Adjusted for ulcer size and ulcer duration to the prognostic index in the model: HR = 0.83, 95% CI = 0.61 to 1.12, P = .23
    - PP analysis: HR = 0.98, 95% CI = 0.69 to 1.38, P = .90
- Healed at endpoint, no. (%): 88 (70.4) in Group 1 vs 101 (80.2) in Group 2, Risk

For Peer Review

Difference (%) (95% CI): -9.8 (-20.4 to 0.9), P = .07
  - Sensitivity analysis: 81 (71.7) in Group 1 vs 94 (81.0) in Group 2, Risk Difference (%) (95% CI): -9.4 (-20.3 to 1.6), P = .10
  - Per protocol: 62 (77.5) in Group 1 vs 67 (81.7) in Group 2, Risk Difference (%) (95% CI): -4.2 (-16.6 to 8.2), P = .52
- Treatment adherence, no. (%): 92 (73.6) in Group 1 vs 92 (73.0) in Group 2, Risk Difference (%) (95% CI): 0.6 (-10.4 to 11.5), P = .92
- Capsule count only, no. (%): 77 (74.8) in Group 1 vs 74 (70.5) in Group 2, Risk Difference (%) (95% CI): 4.3 (-7.8 to 16.4), P = .49
- All adverse events, no.: 40 in Group 1 vs 37 in Group 2, Risk Difference (%) (95% CI): 1.1 (0.7 to 1.7), P = .71
- Serious adverse events, no.: 19 in Group 1 vs 12 in Group 2, Risk Difference (%) (95% CI): 1.6 (0.8 to 3.3), P = .21
- Bleeding events, no.: 9 in Group 1 vs 6 in Group 2, Risk Difference (%) (95% CI): 1.5 (0.5 to 4.3), P = .43
- Mean (SE) Changes in RAND-36
  - Physical functioning: 5.0 (2.2) in Group 1 vs 3.8 (2.2) in Group 2, Mean difference (95% CI): 1.1 (-5.0 to -7.2), P = .714
  - Role physical: 11.7 (3.4) in Group 1 vs 10.3 (3.4) in Group 2, Mean difference (95% CI): 1.4 (-8.0 to 10.8), P = .768
  - Bodily pain: 11.4 (2.2) in Group 1 vs 9.0 (2.2) in Group 2, Mean difference (95% CI): 2.3 (-3.7 to -8.4), P = .449
  - General health: -1.1 (1.4) in Group 1 vs -0.8 (1.4) in Group 2, Mean difference (95% CI): -0.3 (-4.3 to -362), P = .873
  - Vitality: 3.9 (1.6) in Group 1 vs -0.3 (1.6) in Group 2, Mean difference (95% CI): 4.2 (-0.1 to 8.5), P = .057
  - Social functioning: 5.8 (2.2) in Group 1 vs 4.9 (2.2) in Group 2, Mean difference (95% CI): 1.0 (-5.1 to -7.0), P = .756
  - Role emotional: 10.5 (3.1) in Group 1 vs 6.8 (3.1) in Group 2, Mean difference (95% CI): 3.7 (-4.9 to 12.3), P = .400
  - Mental health: -1.2 (1.4) in Group 1 vs 1.2 (1.4) in Group 2, Mean difference (95% CI): -2.3 (-6.2 to 1.5), P = .236
- Mean (SE) Changes in EQ-5D
  - Health state: 7.4 (1.6) in Group 1 vs 4.0 (1.7) in Group 2, Mean difference (95% CI): 3.4 (-1.3 to 8.0), P = .156
  - Utility value: 0.1 (0.0) in Group 1 vs 0.1 (0.0) in Group 2, Mean difference (95% CI): 0.0 (0.0 to 0.1), P = .459
- Mean (SE) Changes in CXVUQ
  - Social function: -5.6 (1.2) in Group 1 vs -6.2 (1.3) in Group 2, Mean difference (95% CI): -1.5 (-5.2 to 2.2), P = .438
  - Domestic activities: -6.2 (1.1) in Group 1 vs -6.6 (1.2) in Group 2, Mean difference (95% CI): -1.4 (-4.5 to 1.9), P = .408
  - Cosmesis: -5.4 (1.5) in Group 1 vs -6.1 (1.6) in Group 2, Mean difference (95% CI):

For Peer Review

- 1.3 (-5.6 to 3.1), P = .568
    - Emotional status: -6.5 (1.8) in Group 1 vs -9.4 (1.8) in Group 2, Mean difference (95% CI): -3.0 (-8.1 to 2.2), P = .257
    - Overall: -5.5 (1.2) in Group 1 vs -7.4 (1.2) in Group 2, Mean difference (95% CI):-1.9 (-5.2 to 1.5), P = .273

Power calculation:

- Was a power calculation for the primary endpoint reported, and if so, was it appropriate? Y, Y

Adjustment for multiplicity of statistical testing:

- Was an adjustment reported if there were more than 1 secondary endpoints statistically tested? N

Blinding:

- Were patients blinded from treatment? Y
- Were assessing investigators blinded from patient treatment? Y

Allocation concealment:

- Was allocation concealment carried out? Y

Lost to follow-up/discontinued/withdrawn:

- 9/251 (3.2%) total
    - 4/125 (2.8%) withdrawn from Group 1
    - 5/126 (4%) withdrawn from Group 2

58. Jünger M, Arnold A, Zuder D, Stahl HW, Heising S. Local therapy and treatment costs of chronic, venous leg ulcers with electrical stimulation (Dermapulse): a prospective, placebo controlled, double blind trial. *Wound Repair Regen.* 2008;16(4):480-487.

Study Groups: Group 1: electrical stimulation; Group 2: placebo treated with electrodes using same pulse generator but with nonconductive power cable, 39 enrolled in both groups, no information reported on how many per group

Statistical results:

- ITT analysis done for primary endpoint(s): Y
- **There were no primary endpoints reported**
- Median (range) of ulcer area reduction in 4 months, mm^2: 550 (115-3,692) to 80 (0-2,819) in group 1 (n = 3 healed ulcers), p = .03 vs 542 (66-2,819) to 346 (0-3,769) in group 2 (n = 2 healed ulcers), p = NS (paired t- test)
    - Group difference of ulcer area reduction, p = NS
- Mean (range) of ulcer size, mm^2 (n = 39):
    - 0 mo: 980 (0-980) in Group 1 vs 800 (800-1,550) in Group 2, p = NS
    - 0.5 mo: 820 (100-820) in Group 1 vs 800 (800-1,520) in Group 2, p = NS
    - 1 mo: 800 (-150 to 800) in Group 1 vs 800 (800 to 1,550) in Group 2, p = NS
    - 2 mos: 800 (-200 to 800) in Group 1 vs 800 (800 to 1,630) in Group 2, p = NS
    - 3 mos: 650 (-180 to 650) in Group 1 vs 830 (830 to 1,800) in Group 2, p = .05
    - 4 mos: 500 (-250 to 500) in Group 1 vs 700 (700 to 1,680) in Group 2, p = .03
- Mean (range) of pain reported, 5- point scale (n = 39):
    - 0 mo: 3 (1.8-3.0) in Group 1 vs 3 (3.0, 4.0) in Group 2, p = NS

For Peer Review

    - 0.5 mo: 2.7 (1.7, 2.7) in Group 1 vs 2.8 (2.8-3.9) in Group 2, p = NS
    - 1 mo: 2.4 (1.6-2.4) in Group 1 vs 2.7 (2.7-3.7) in Group 2, p = NS
    - 2 mos: 2.4 (1.5-2.4) in Group 1 vs 2.6 (2.6-3.8) in Group 2, p = NS
    - 3 mos: 2.5 (1.6-2.5) in Group 1 vs 2.5 (2.5-3.6) in Group 2, p = NS
    - 4 mos: 2.0 (1.4-2.0) in Group 1 vs 2.9 (2.9-4.0) in Group 2, p = .049
    - Significant pain reduction in Group 1 at: 1 mo: p = .02; 2 mos: p = .04; 4 mos: p = .01
- Mean (range) of capillary density in the ulcer; and in the ulcer border, no. capillaries/mm^2 (n = 39):
    - 0 mo: 32 (6-32) in Group 1 vs 25 (25-39) in Group 2, p = NS; 46 (19-46) vs 44 (44-61) , p = NS
    - 0.5 mo: 32 (14-32) in Group 1 vs 28 (28-45) in Group 2, p = NS; 45 (26-45) vs 45 (45-65), p = NS
    - 1 mo: 32 (10-32) in Group 1 vs 30 (30-57) in Group 2, p = NS; 44 (28-44) vs 47 (47-68), p = NS
    - 2 mos: 36 (15-36) in Group 1 vs 33 (33-56) in Group 2, p = .04; 46 (28- 46) vs 52 (52-72), p = NS
    - 3 mos: 45 (30-45) in Group 1 vs 33 (33-58) in Group 2, p = NS; 47 (34-47) vs 47 (47-67), p = NS
    - 4 mos: 37 (19-37) in Group 1 vs 34 (34-57) in Group 2, p = .01; 53 (35-53) vs 54 (54-77), p = NS
- Mean (range) of transcutaneous oxygen partial pressure in the ulcer edge; and at the forefoot, mmHg (n = 39):
    - 0 mo: 20 (5-20) in Group 1 vs 12 (12-25) in Group 2, p = NS; 36 (36-47) vs 42 (26-42), p = NS
    - 0.5 mo: 16 (6-16) in Group 1 vs 13 (13-26) in Group 2, p = NS; 39 (39-58) vs 41 (31-41), p = NS
    - 1 mo: 17 (7-17) in Group 1 vs 15 (15-31) in Group 2, p = NS; 45 (45-58) vs 42 (31-42), p = NS
    - Time 2: 15 (2-15) in Group 1 vs 16 (16-39) in Group 2, p = NS; 42 (42-56) vs 48 (36-48), p = NS
    - Time 3: 15 (3-15) in Group 1 vs 17 (17-37) in Group 2, p = NS; 42 (42-54) in Group 1 vs 42 (24-42) in Group 2, p = NS
    - Time 4: 15 (1-15) in Group 1 vs 20 (20-43) in Group 2, p = .02; 49 (49-56) in Group 1 vs 42 (25-42) in Group 2, p = .0006

Power calculation:

- Was a power calculation for the primary endpoint reported, and if so, was it appropriate? N

Adjustment for multiplicity of statistical testing:

- Was an adjustment reported if there were more than 1 secondary endpoints statistically tested? N

Blinding:

- Were patients blinded from treatment? Y

For Peer Review

- Were assessing investigators blinded from patient treatment? Y

Allocation concealment:

- Was allocation concealment carried out? N

Lost to follow-up/discontinued/withdrawn: None reported.

59. Jünger M, Wollina U, Kohnen R, Rabe E. Efficacy and tolerability of an ulcer compression stocking for therapy of chronic venous ulcer compared with a below-knee compression bandage: results from a prospective, randomized, multicentre trial. *Curr Med Res Opin.* 2004; 20(10):1613-1623.

Study Groups: Group 1: U-Stocking; Group 2: bandages

Statistical results:

- ITT analysis done for primary endpoint(s): N.
- **Primary endpoint:** Rate of complete healing after 12 weeks, no. (%): 29/61 (47.5) in Group 1 vs 19/60 (31.7) in Group 2, Group difference = 15.8%, 95% CI = 4.3% y 28.5, respectively, p = .0129
- Mean (SD) time to heal: 46 (20) days (range 10-83, median 47) in Group 1 vs 46 (22) (range 6-80, median 52) in Group 2, p = .8165
- Life-table analysis: Higher relative frequency of complete healing which accelerates after 4 weeks in Group 1, p = .0565
- Mean (SD, median, range) Surface area reduction: -74.8% (42.4, -98.4, -100 to 83.1) in Group 1 vs -51.4% (86.7, -82.9, -100 to 396.2), p = .0679
- Mean (SD, median, range) duration of therapy, days: 61 (26, 70, -10 to 100) in Group 1 vs 68 (25, 83, 6 to 92), p = .0297
- Bandages worn for at least 8 hours on more than 80% of the study days, no. (%): 57 (93.4%) in Group 1 vs 50 (84.8%) in Group 2, p = .1504
- Mean (SD, median) duration of compression therapy per day, hours: 12.7 (2.9, 12.2) in Group 1 vs 16.9 (5.7, 15.9) in Group 2, p = .0002
- Statements regarding difficulties in application, no. (%): mild: 11 (20) in Group 1 vs 12 (23) in Group 2; moderate: 4 (7) vs 6 (11); great difficulties: 2 (4%) vs 0, p = 9323
- Time required for application judged to be high, no. (%): 1/55 (1.8) in Group 1 vs 5/54 (9.3) in Group 2, p = .0915
- Patient assessment of Constriction
  - None, no. (%): 35/56 (63) in Group 1 vs 20/54 (37) in Group 2
  - Median all categories (quartiles): 0 (0-1) in Group 1 vs 1 (0-2) in Group 2, p = .0032
- Patient assessment of Tightness
  - None, no. (%): 31/56 (55) in Group 1 vs 25/52 (48) in Group 2
  - Median all categories (quartiles): 0 (0-1) in Group 1 vs 1 (0-1.5) in Group 2, p = .2313
- Patient assessment of restricted freedom of movement
  - None, no. (%): 40/56 (71) in Group 1 vs 22/53 (42) in Group 2
  - Median all categories (quartiles): 0 (0-1) in Group 1 vs 1 (0-1) in Group 2, p = .0009

For Peer Review

- Patient assessment of pain in the leg
    - None, no. (%): 31/56 (55) in Group 1 vs 21/54 (39) in Group 2
    - Median all categories (quartiles): 0 (0-1) in Group 1 vs 1 (0-2) in Group 2, p = .0700
- Patient assessment of burning in the leg
    - None, no. (%): 40/55 (73) in Group 1 vs 32/54 (59) in Group 2
    - Median all categories (quartiles): 0 (0-1) in Group 1 vs 0 (0-1) in Group 2, p = .1463
- Patient assessment of sweating under the dressing
    - None, no. (%): 41/56 (53) in Group 1 vs 25/53 (47) in Group 2
    - Median all categories (quartiles): 0 (0-1) in Group 1 vs 1 (0-2) in Group 2, p = .4770
- Patient assessment of heat sensation in the leg
    - None, no. (%): 29/55 (63) in Group 1 vs 20/54 (37) in Group 2
    - Median all categories (quartiles): 0 (0-1) in Group 1 vs 1 (0-2) in Group 2, p = .0032
- Patient assessment of itching of the skin on the leg
    - None, no. (%): 32/56 (57) in Group 1 vs 16/54 (30) in Group 2
    - Median all categories (quartiles): 0 (0-1) in Group 1 vs 1 (0-2) in Group 2, p = .0063
- Patient assessment of prickling of the leg
    - None, no. (%): 43/56 (77) in Group 1 vs 34/54 (63) in Group 2
    - Median all categories (quartiles): 0 (0-0) in Group 1 vs 0 (0-1) in Group 2, p = .1180
- Nursing assessment of effect of therapy
    - good, no. (%): 20 (34) in Group 1 vs 30 (52) in Group 2, p = 0.0132
- Nursing assessment of discomfort
    - moderate, no. (%): 2 (3) in Group 1 vs 3 (5) in Group 2, p = 0.0488
- Nursing assessment of support required at study site
    - moderate, no. (%): 10 (17) in Group 1 vs 13 (22) in Group 2, p = 0.2853
- Nursing assessment of compliance
    - moderate, no. (%): 1 (2) in Group 1 vs 7 (12) in Group 2, p = 0.3142
- Nursing assessment of satisfaction of nursing staff with treatment
    - Neither/nor, no. (%): 2 (3) in Group 1 vs 6 (10) in Group 2, p = 0.0123
- Mean (SD) nursing assessment of nursing time, minutes (median, quartiles):5.4 (5.4) (3, 2-5) in Group 1 vs 8.5 (6.5) (6, 5-10) in Group 2, p = 0.001

Power calculation:

- Was a power calculation for the primary endpoint reported, and if so, was it appropriate? Y, Y

Adjustment for multiplicity of statistical testing:

- Was an adjustment reported if there were more than 1 secondary endpoints statistically tested? N

Blinding:

For Peer Review

- Were patients blinded from treatment? N
- Were assessing investigators blinded from patient treatment? N

Allocation concealment:

- Was allocation concealment carried out? N

Lost to follow-up/discontinued/withdrawn:

- 13/134 (9.7%) total
    - 5/66 (7.6%) withdrawn from Group 1
    - 8/68 (11.8%) withdrawn from Group 2

60. Kapp S, Miller C, Donohue L. The clinical effectiveness of two compression stocking treatments on venous leg ulcer recurrence: a randomized controlled trial. *Int J Low Extrem Wounds.* 2013;12(3):189-198.

Study Groups: Group 1: 23-32 mm Hg compression; Group 2: 34-46 mm Hg compression

Statistical results:

- ITT analysis done for primary endpoint(s): N
- **Primary endpoint:** mean (SD) time to recurrence was 77.9 days (44.6), 74.9 (45.6) in Group 1 vs 83.2 (48.9) in Group 2, p = .651
    - 11 (11.8%) experienced a recurrence (14.3% of wounds in Groups 1 vs 9.1% in Group 2) with most recurring by 13 weeks (8.6%)
    - Other ulcer recurrence, % recurring: 6.1% in Group 1 vs 2.3% in Group 2
    - Other ulcer occurrence, % occurring: 14.3% in Group 1 vs 13.6% in Group 2, p = 1.000
- First block of Cox Regression Model 1, Moderate compression stocking level, high compression stocking reference did not predict time to recurrence, $G^2(1) = 0.581$, p = .446
    - Wald (df) = .571 (1); p = .450; HR (95% CI) = 1.606 (0.470-5.485)
- Cox Regression Model 2, Moderate compression stocking level, high compression stocking reference treatment including adherence and client education was significantly better than Model 1, $G^2(1) = 11.405$, p = .003
    - Treatment, Moderate compression stocking level, high compression stocking reference: Wald (df) = 3.205 (1); p = .073; HR (95% CI) = 3.175 (0.896-11.249)
    - Adherence (N/Y): Wald (df) = 7.917 (1); p = .005; HR (95% CI) = 9.652 (1.990-46.826)
    - Patient education (N/Y): Wald (df) = 7.917 (1); p = .073; HR (95% CI) = 2.025 (0.590-6.945)
    - HRs suggest that the risk recurrence was +9 times greater if not adhering to randomized compression stocking than when it was adhered to and 3 times greater if randomized to moderate compression vs high compression
    - For new occurring ulcers (n = 13), treatment, adherence, and patient education were not significant predictors: $G^2(3) = 0.929$, p = .819
- Adherence: 91.4% of participants wore 4LB for first 2 weeks posthealing with no difference between groups, p = .596
    - Nonadherence to 4LB posthealing was not related to recurrence (p = 1.000) or

For Peer Review

time to recurrence (p = .573)
    - 55.9% did not adhere to their randomized compression stocking treatment for more than half of the study: 61.4% in Group 2 vs 28.6% in Group 1 (p = .003)
    - Significant difference in the percentage of study time Group 1 adhered to treatment (65.8%) vs Group 2 (p = .004)
    - No difference between groups in the type of compression that was used by participants not adhering to their randomized treatment, p = .374
        - Most used low or moderate compression (56.5%) vs high compression (19.6%); 23.9% did not use any compression

Power calculation:
- Was a power calculation for the primary endpoint reported, and if so, was it appropriate? N

Adjustment for multiplicity of statistical testing:
- Was an adjustment reported if there were more than 1 secondary endpoints statistically tested? N

Blinding:
- Were patients blinded from treatment? Y
- Were assessing investigators blinded from patient treatment? Y

Allocation concealment:
- Was allocation concealment carried out? N

Lost to follow-up/discontinued/withdrawn:
- 4/100 (4.0%) total
    - 2/53 (3.8%) withdrawn from Group 1
    - 2/47 (4.3%) withdrawn from Group 2

61. Kerihuel JC. Effect of activated charcoal dressings on healing outcomes of chronic wounds. *J Wound Care.* 2010;19(5):208,10-2,14-5.

Study Groups: Group 1: activated charcoal dressing (Actisorb Silver 220) + compression bandaging; Group 2: control (Duoderm) + compression bandaging

Statistical results:
- ITT analysis done for primary endpoint(s): N
- **Primary endpoint:** Median (range) absolute reduction in wound area (cm^2):
    - At 1 week: -2.2 (-21.2 to 5.0) in Group 1 vs -0.1 (-23.4 to 18.3) in Group 2, p = .066
    - At 4 weeks: -4.5 (-30.9 to 22.5) vs -3.5 (-53.3 to 18.5), p = NS
- Median (range) PAR
    - At 1 week: -16.4 (-100 to 80) in Group 1 vs -0.9 (-84 to -82.9) in Group 2, p = .074
    - At 4 weeks: -35.6 (-100 to 182.1) vs -40.9 (-100 to 308.3), p = NS

Power calculation:
- Was a power calculation for the primary endpoint reported, and if so, was it appropriate? N

Adjustment for multiplicity of statistical testing:
- Was an adjustment reported if there were more than 1 secondary endpoints statistically

For Peer Review

tested? N

Blinding:

- Were patients blinded from treatment? N
- Were assessing investigators blinded from patient treatment? N

Allocation concealment:

- Was allocation concealment carried out? N

Lost to follow-up/discontinued/withdrawn:

- 7/60 (11.7%) total
  - 1/30 (3.3%) withdrawn from Group 1
  - 6/30 (20%) withdrawn from Group 2

62. Kirsner RS, Marston WA, Snyder RJ, Lee TD, Cargill DI, Slade HB. Spray-applied cell therapy with human allogeneic fibroblasts and keratinocytes for the treatment of chronic venous leg ulcers: a phase 2, multicentre, double-blind, randomised, placebo-controlled trial. *Lancet*. 2012;380(9846):977-985.

Study Groups: Group 1: HP802-247 spray-applied cell therapy $0.5x10^6$ cells in vehicle every 14 days; Group 2: $0.5x10^6$ cells in vehicle every 7 days; Group 3: $5.0x10^6$ cells in vehicle every 14 days; Group 4: $0.5x10^6$ cells in vehicle every 7 days; Group 5: vehicle alone; vehicle = human fibrinogen solution and a separate human thrombin solution based on a 1:14 diluted commercial tissue sealant

Statistical results:

- ITT analysis done for primary endpoint(s): N
- **Primary endpoint:** Mean PAR at 12 weeks: Group 1: 91% (SD 21%); Group 2: 87% (SD 20%); Group 3: 84% (SD 26%); Group 4: 87% (SD 23%); Group 5: 80% (SD 30%)
  - Groups 1-4 had a greater mean PAR than Group 1 during 12 weeks treatment period (p = .0446), with the following differences in PAR between active groups and vehicle:
    - Group 1: -16.0% (95% CI: -26.4 to -5.56), p = .0028
    - Group 2: -9.16% (95% CI: -19.8 to 1.44), p = .09
    - Group 3: -7.60% (95% CI: -18.2 to 2.99), p = .16
    - Group 4: -11.7% (95% CI: 22.2 to -1.17), p = .0295
  - For Groups 1-4, no significant effects for high vs low dose (p = .51), every 7 vs every 14 days (p = .79), or dose-by-regimen interaction (p = .12)
  - No differences in proportion achieving 50% PAR; 88%-98% reached this endpoint
  - After 1 application, mean PAR in Group 1 as 40% (SD: 32%) vs 23% (SD: 27%) in Group 5; maximum difference at week 7 [87% (SD 19%) vs 65% (SD 43%)]
    - Significant differences (p <.05) occurred at all weeks but weeks 4 and 12
- Proportion healed by week 12, compared to Group 5: Group 5: 23 (46%); Group 1: 32 (70%), p = .0028; Group 2: 25 (58%), p = .36; Group 3: 28 (65%), p = .09; Group 4: 29 (64%), p = .09.
  - Baseline wound duration significant inverse correlation with closure (p < .001), but this was not significant as a covariate compared with treatment
- Time to heal, compared to Group 5; Group 5: 71 (95% CI: 57 to NA); Group 1; 50 (95%

For Peer Review

CI: 37 to 66), p = .0211; Group 2: 64 (95% CI: 43 to NA), p =.59; Group 3: 57 (95% CI: 50 to 85), p = .26; Group 4: 57 (95% CI: 43 to 71), p = .10.
- Cox HR relative to Group 5: Group 1: 1.83 (95% CI: 1.09 to 3.04), p = .0212; Group 2: 1.19 (95% CI: 0.69 to 2.05), p = .53; Group 3: 1.43 (95% CI: 0.84 to 2.44), p = .19; Group 4: 1.64 (95% CI: 0.97 to 2.77), p = .06
- A greater reduction in pain was found in Groups 1-4 (from -3.8 to -8.7) than in Group 1 (0.2, p = .0464); except for a significant difference favoring Groups 1-4 at week 12 (p = .0251), pain score reductions were much the same between groups at weekly visits (no data)

Power calculation:
- Was a power calculation for the primary endpoint reported, and if so, was it appropriate? Y, Y

Adjustment for multiplicity of statistical testing:
- Was an adjustment reported if there were more than 1 secondary endpoints statistically tested? N

Blinding:
- Were patients blinded from treatment? N
- Were assessing investigators blinded from patient treatment? N

Allocation concealment:
- Was allocation concealment carried out? Y

Lost to follow-up/discontinued/withdrawn:
- 23/228 (10%) total
  - 8/46 (17.4%) withdrawn from Group 1
  - 1/43 (2.3%) withdrawn from Group 2
  - 5/44 (11.4%) withdrawn from Group 3
  - 5/45 (11.1%) withdrawn from Group 4
  - 4/50 (8%) withdrawn from Group 5

63. Klonizakis M, Tew GA, Gumber A, et al. Supervised exercise training as an adjunct therapy for venous leg ulcers: a randomized controlled feasibility trial. *B J Dermatol.* 2018;178(5):1072-1082.

Study Groups: Group 1: Exercise (3 supervised sessions/week) + compression; Group 2: compression (control)

Statistical results:
- ITT analysis done for primary endpoint(s): N
- **There were no primary endpoints identified.** There was no statistical analysis/p values.

Power calculation:
- Was a power calculation for the primary endpoint reported, and if so, was it appropriate? N

Adjustment for multiplicity of statistical testing:
- Was an adjustment reported if there were more than 1 secondary endpoints statistically tested? N

Blinding:

For Peer Review

- Were patients blinded from treatment? N
- Were assessing investigators blinded from patient treatment? Y

Allocation concealment:

- Was allocation concealment carried out? N

Lost to follow-up/discontinued/withdrawn:

- 2/39 (5.1%) total
  - 1/18 (5.6%) withdrawn from Group 1
  - 1/21 (4.8%) withdrawn from Group 2

64. Koksal C, Bozkurt AK. Combination of hydrocolloid dressing and medical compression stockings versus Unna's boot for the treatment of venous leg ulcers. *Swiss Med Wkly.* 2003; 133(25-26):364-368.

Study Groups: Group 1: hydrocolloid; Group 2: Unna boot

Statistical results:

- ITT analysis done for primary endpoint(s): N
- **There were no primary endpoints identified.**
- Complete healing rate at 4 months, % (no.): 80.76 (21/26) in Group 1 vs 74.07 (20/27) in Group 2, p >.05
- Mean (SD) time to heal, weeks: 6.65 (3.31) in Group 1 vs 6.85 (3.60) in Group 2, p >.05
- Mean (SD) of ease of use score: 17.27 (3.27) in Group 1 vs 9.04 (2.38) in Group 2, p <.0001
- Mean (SD) of pain during application: 1.88 (1.48) in Group 1 vs 3.69 (1.35) in Group 2, p <.0001
- Mean (SD) of pain at home: 1.88 (1.11) in Group 1 vs 3.27 (1.08) in Group 2, p <.0001
- Mean (SD) of application time, minutes: 134.54 (43.39) in Group 1 vs 150.59 (34.73) in Group 2, p = NS

Power calculation:

- Was a power calculation for the primary endpoint reported, and if so, was it appropriate? N

Adjustment for multiplicity of statistical testing:

- Was an adjustment reported if there were more than 1 secondary endpoints statistically tested? N

Blinding:

- Were patients blinded from treatment? N
- Were assessing investigators blinded from patient treatment? N

Allocation concealment:

- Was allocation concealment carried out? N

Lost to follow-up/discontinued/withdrawn:

- 7/60 (11.7%) total
  - 4/30 (13.3%) withdrawn from Group 1
  - 3/30 (10.0%) withdrawn from Group 2

65. Kopera D, Kokol R, Berger C, Haas J. Does the use of low-level laser influence wound

For Peer Review

healing in chronic venous leg ulcers? *J Wound Care.* 2005;14(8):391-394.

Study Groups: Group 1: low-lever laser; Group 2: placebo visible light of an incoherent polychromatic non-laser commercial LED light source with a red glass window; Group 3: control SOC

Statistical results:

- ITT analysis done for primary endpoint(s): N
- **Primary endpoint:** Percent reduction in wound size at day 28, n = 43
    - >20% reduction: Group 1: 58.8%; Group 2: 75.0%; Group 3: 50.0%
    - No change: Group 1: 17.6%; Group 2: 12.5%; Group 3: 40.0%
    - >20% increase: Group 1: 23.5%; Group 2: 12.5; Group 3: 10.0%
    - Difference baseline to day 28: Group 1: p = .492; Group 2: p = .023; Group 3: p = .047
    - Percent reduction in wound size at day 90, n = 37
        - >20% reduction: Group 1: 42.8; Group 2: 71.4%; Group 3: 66.6%
        - No change: Group 1: 28.5%; Group 2: 21.4%; Group 3: 22.2%
        - >20% increase: Group 1: 28.5%; Group 2: 7.1%; Group 3: 11.1%
        - Difference baseline to day 28: Group 1: p = .683; Group 2: p = .011; Group 3: p = .066
    - Change from day 28 to day 90: Group 1: p = .0861; Group 2: p = .223; Group 3: p = .086
- Ulcer diameters and circumferences were not significantly different between groups, p = NS

Power calculation:

- Was a power calculation for the primary endpoint reported, and if so, was it appropriate? Y, N

Adjustment for multiplicity of statistical testing:

- Was an adjustment reported if there were more than 1 secondary endpoints statistically tested? N

Blinding:

- Were patients blinded from treatment? N
- Were assessing investigators blinded from patient treatment? N

Allocation concealment:

- Was allocation concealment carried out? N

Lost to follow-up/discontinued/withdrawn:

- 7/44 (15.9%) total
    - 3/17 (17.6%) withdrawn from Group 1
    - 3/17 (17.6%) withdrawn from Group 2
    - 1/10 (10.0%) Withdrawn from Group 3

66. Krasowski G, Jawien A, Tukiendorf A, et al. A comparison of an antibacterial sandwich dressing vs dressing containing silver. *Wound Repair Regen.* 2015;23(4):525-530.

Study Groups: Group 1: antibacterial sandwich dressing; Group 2: silver dressing

Statistical results:

For Peer Review

- ITT analysis done for primary endpoint(s): N
- **Primary endpoint:** Mean (variation) reduction in surface area, cm^2/wk
    - All patients: 1.58 (0.77) in Group 1 vs 0.23 (0.88) in Group 2, t = 2.371, p = .0182
    - <10 cm^2: 0.33 (0.30) in Group 1 vs 0.25 (0.33) in Group 2, t = 0.297, p = .7768
    - >10 cm^2: 3.64 (1.14) in Group 1 vs 0.19 (1.51) in Group 2, t = 2.168, p = .0322
- Mean (SE) correlation between pain (VAS) and time, Poisson regression: Group 1: -0.011 (0.002), p = .0000; Group 2: -0.008 (0.001), p = .0000
- Microbiological eradication, chi-square test, 23 in Group 1 vs 32 in Group 2
    - Eradication, day 28: 5 (21.7%) in Group 1 vs 1 (3.1%) in Group 2, p = .08
    - Eradication, day 56: 16 (69.6%) vs 43.8%, p = .10

Power calculation:

- Was a power calculation for the primary endpoint reported, and if so, was it appropriate? Y, N

Adjustment for multiplicity of statistical testing:

- Was an adjustment reported if there were more than 1 secondary endpoints statistically tested? N

Blinding:

- Were patients blinded from treatment? N
- Were assessing investigators blinded from patient treatment? N

Allocation concealment:

- Was allocation concealment carried out? N

Lost to follow-up/discontinued/withdrawn:

- 4/80 (5%) total
    - 2/40 (5.0%) withdrawn from Group 1
    - 2/40 (5.0%) withdrawn from Group 2

67. Kucharzewski M, Franek A, Koziolek H. Treatment of venous leg ulcers with sulodexide. *Phlebologie.* 2003;32(5):115-120.

Study Groups: Group 1: sulodexide + Unna's boo (n = 23); Group 2: Unna's boot alone (n = 21)

Statistical results:

- ITT analysis done for primary endpoint(s): Y
- **There were no primary endpoints identified.**
- Mean (SD) ulceration area, cm^2
    - Week 0: Group 1: 16.2 (4.1); Group 2: 15.8 (3.1), p <.005
    - Week 1: Group 1: 15.8 (3.6); Group 2: 14.6 (3.0), p <.005
    - Week 2: Group 1: 13.9 (3.1); Group 2: 14.2 (2.9), p <.005
    - Week 3: Group 1: 12.5 (2.4); Group 2: 13.9 (2.7), p <.005
    - Week 4: Group 1: 11.0 (2.1); Group 2: 13.7 (2.6), p <.005
    - Week 5: Group 1: 9.5 (2.2); Group 2: 13.0 (2.6), p <.005
    - Week 6: Group 1: 7.9 (1.8); Group 2: 12.1 (2.4), p <.005
    - Week 7: Group 1: 6.3 (1.6); Group 2: 10.9 (2.2), p <.005
    - Week 8: Group 1: 4.7 (1.4); Group 2: 9.8 (2.0), p <.005
    - Week 9: Group 1: 2.4 (1.2); Group 2: 8.7 (2.1), p <.005

For Peer Review

- Week 10: Group 1: 0; Group 2: 7.7 (1.8), p <.005

Power calculation:

- Was a power calculation for the primary endpoint reported, and if so, was it appropriate? N

Adjustment for multiplicity of statistical testing:

- Was an adjustment reported if there were more than 1 secondary endpoints statistically tested? N

Blinding:

- Were patients blinded from treatment? N
- Were assessing investigators blinded from patient treatment? N

Allocation concealment:

- Was allocation concealment carried out? N

Lost to follow-up/discontinued/withdrawn: None reported.

68. Kucharzewski M, Slezak A, Franke A. Topical treatment of non-healing venous leg ulcers by cellulose membrane. *Phlebologie.* 2003;32(5):147-151.

Study Groups: Group 1: cellulose membrane (n = 27); Group 2: Unna's boot (n = 27)

Statistical results:

- ITT analysis done for primary endpoint(s): Y
- **There were no primary endpoints identified.**
- Mean (SD) ulceration area (cm^2) according to treatment duration
  - Day 0: 28.9 (4.1) in Group 1 vs 27.8 (3.3) in Group 2, t = 1.437, p <.5
  - Day 7: 25.4 (3.7) in Group 1 vs 25.3 (2.9) in Group 2, t = 0.959, p <.5
  - Day 14: 22.6 (3.1) in Group 1 vs 24.2 (2.5) in Group 2, t = 0.850, p <.5
  - Day 28: 20.1 (2.4) in Group 1 vs 23.2 (2.4) in Group 2, t = 4.162, p <.001
  - Day 56: 13.4 (1.5) in Group 1 vs 17.3 (1.7) in Group 2, t = 9.293, p <.001
  - Day 77: 6.2 (0.7) in Group 1 vs 12.4 (1.5) in Group 2, t = 21.252, p <.0001
  - Day 98: 0 (0) in Group 1 vs 8.6 (0.9) in Group 2, t = 44.105, p <.0001
  - Day 119: 0 (0) in Group 1 vs 4.5 (0.5) in Group 2, t = 45.38, p <.0001
  - Day 140: 0 (0) in Group 1 vs 0 (0) in Group 2, t = 0, p = 0

Power calculation:

- Was a power calculation for the primary endpoint reported, and if so, was it appropriate? N

Adjustment for multiplicity of statistical testing:

- Was an adjustment reported if there were more than 1 secondary endpoints statistically tested? N

Blinding:

- Were patients blinded from treatment? N
- Were assessing investigators blinded from patient treatment? N

Allocation concealment:

- Was allocation concealment carried out? N

Lost to follow-up/discontinued/withdrawn: None reported

For Peer Review

69. Kucharzewski M, Wilemska-Kucharjewska K, Kozka M, et al. Leg venous ulcer healing process after application of membranous dressing with silver ions. *Phlebologie.* 2013;42(6): 340-346.

Study Groups: Group 1: membranous dressings with silver ions; Group 2: hydrocolloid dressing Unna boot

Statistical results:

- ITT analysis done for primary endpoint(s): Y
- **There were no primary endpoints identified**
- Mean (SD) speed of decreasing in surface area, cm^2 per day
    - 1 week: 0.2838 (0.0744) in Group 1 (n = 28) vs 0.2031 (0.0349) in Group 2 (n = 28), p <.001
    - 2 week: 0.2033 (0.0805) in Group 1 (n = 25) vs 0.1336 (0.0262) in Group 2 (n = 28), p <.001
    - 3 week: 0.1936 (0.0759) in Group 1 (n = 20) vs 0.826 (0.0244) in Group 2 (n = 28), p <.001
    - 4 week: 0.2793 (0.0671) in Group 1 (n = 17) vs 0.366 (0.0240) in Group 2 (n = 22), p <.001
    - 5 week: 0.1668 (0.0481) in Group 1 (n = 10) vs 0.125 (0.0161) in Group 2 (n = 22), p <.001
    - 6 week: 0.1114 (0.0648) in Group 1 (n = 3) vs 0.075 (0.0243) in Group 2 (n = 22), p <.001
    - 7 week: 0.1160 (0.0251) in Group 1 (n = 7) vs 0.045 (0.0504) in Group 2 (n = 22), p <.001

Power calculation:

- Was a power calculation for the primary endpoint reported, and if so, was it appropriate? N

Adjustment for multiplicity of statistical testing:

- Was an adjustment reported if there were more than 1 secondary endpoints statistically tested? Y

Blinding:

- Were patients blinded from treatment? N
- Were assessing investigators blinded from patient treatment? N

Allocation concealment:

- Was allocation concealment carried out? N

Lost to follow-up/discontinued/withdrawn:

- 28/102 (27.4%) total
    - 8/52 (15.4%) withdrawn from Group 1
    - 20/50 (40%) withdrawn from Group 2

70. Lammoglia-Ordiales L, Vega-Memije ME, Herrera-Arellano A, et al. A randomised comparative trial on the use of a hydrogel with tepescohuite extract (Mimosa tenuiflora cortex extract-2G) in the treatment of venous leg ulcers. *Int Wound J.* 2012;9(4):412-418.

For Peer Review

Study Groups: Group 1: hydrogel with *Mimosa tenuiflora* cortex extract; Group 2: hydrogel alone (placebo)

Statistical results:

- ITT analysis done for primary endpoint(s): N
- **Primary endpoint:** Mean PAR (cm^2): 6.3 (95% CI: 3.3-9.3) in Group 1, p = .0001 vs 5.8 (95% CI: 3.6-8.1) in Group 2, p = .0001
    - Group difference, p = .815
- There were no significant group differences between percentage of granulation tissue (p = .219), fibrin (p = .321), maceration or necrosis (p = .223)
- The presence of epithelium islands was 58% in Group 1 vs 39% in Group 2, p = .02
- Complete closure: 4/22 in Group 1 vs 3/19 in Group 2, p = .334
- No. with necrosis from baseline to study end: 5 to 1 in Group 1 vs 5 to 4 in Group 2, p = .035
- In the initial and final biopsies, there were no group differences regarding fibrosis, vascular proliferation, granulation tissue, and perivascular fibrosis, p = NS
- In Group 1, the density of neutrophils decreased significantly compared to Group 2, p = .05
- The most common side effects were pain and burning in 4 patients in Group 1 vs 5 in Group 2, p = NS

Power calculation:

- Was a power calculation for the primary endpoint reported, and if so, was it appropriate? Y, Y

Adjustment for multiplicity of statistical testing:

- Was an adjustment reported if there were more than 1 secondary endpoints statistically tested? N

Blinding:

- Were patients blinded from treatment? Y
- Were assessing investigators blinded from patient treatment? Y

Allocation concealment:

- Was allocation concealment carried out? N

Lost to follow-up/discontinued/withdrawn:

- 9/41 (22%) total
    - 4/22 (18.2%) withdrawn from Group 1
    - 5/19 (26.3%) withdrawn from Group 2

71. Lazareth I, Meaume S, Sigal-Grinberg ML, Combemale P, Le Guyadec T, Zagnoli A. Efficacy of a silver lipidocolloid dressing on heavily colonised wounds: a republished RCT. *J Wound Care.* 2012;21(2):96-102.

Study Groups: Group 1: silver lipidcolloid dressing; Group 2: same contact dressing without silver, 102 patients enrolled

Statistical results:

- ITT analysis done for primary endpoint(s): N
- **Primary endpoint:** Mean (SD, median) wound area reduction, cm^2

For Peer Review

  - Week 4 absolute reduction: -6.5 (13.4, -4.2) in Group 1 vs -1.3 (9.0, -1.1)in Group 2, p = .023
  - Week 4 closure rate, cm^2/ day: 0.20 (0.42, 0.15) in Group 1 vs 0.08 (0.56, 0.04) in Group 2, p = .009
    - After switching to the non-silver containing dressing, this closure rate remained unchanged (0.135 in Group 1 vs 0.023 In Group 2, p = 0.01)
  - Week 8 absolute reduction: -8.1 (16.3, -5.9) in Group 1 vs -1.0 (12.0, -0.8) in Group 2, p = .002
  - Week 8 relative reduction, %: -36.6 (48.8, -47.9) in Group 1 vs -6.2 (80.2, -5.6) in Group 2, p = .036
  - Week 8 closure rate, cm^2/ day: 0.14 (0.27, 0.14) in Group 1 vs 0.10 (0.54, 0.02) in Group 2, p = .001
- PAR ≥ 40%: 55% in Group 1 vs 35% in Group 2, p = 0.051, OR = 2.7 (95%CI: 1.1 to 6.7, p = 0.038)
  - Age, BMI, ulcer duration, and baseline area did not have significant influence, p = NS
  - Treatment factor had significant influence, p = 0.034
- Percentage of ulcers without clinical signs of bacteria colonization:
  - Week 4: 39.2 in Group 1 vs 16.7 in group 2, p = .0097
  - Week 8: 35.2 in Group 1 vs 16.7 in group 2, p = .044
  - No. of clinical signs decreased significantly more in Group 1 vs Group 2 during the initial 4 weeks of the study (-2.5 ± 1.5 vs -1.0 ± 1.4, $p < .001$)
- 22 local adverse events (11 in each group) were reported in 20 patients; there were no differences in AEs among groups, p = NS
- Dressing acceptation was similar in both groups, and the interval between two dressing changes was a little bit longer in Group 1 vs Group 2, days: 2.12 vs 1.84, p = NS.

Power calculation:
- Was a power calculation for the primary endpoint reported, and if so, was it appropriate? Y, N

Adjustment for multiplicity of statistical testing:
- Was an adjustment reported if there were more than 1 secondary endpoints statistically tested? N

Blinding:
- Were patients blinded from treatment? N
- Were assessing investigators blinded from patient treatment? N

Allocation concealment:
- Was allocation concealment carried out? N

Lost to follow-up/discontinued/withdrawn: None reported.

72. Leach MJ, Pincombe J, Foster G. Clinical efficacy of horsechestnut seed extract in the treatment of venous ulceration. *J Wound Care.* 2006;15(4):159-167.

Study Groups: Group 1: horse chestnut seed abstract tablet; Group 2: placebo table
Statistical results:

For Peer Review

- ITT analysis done for primary endpoint(s): Y
- **Primary endpoint:** No. of ulcers healed by week 12: 12/27 in Group 1 vs 7/27 in Group 2, log rank = 1.76, p =.185
    - The no. of leg ulcers changed significantly over time for both groups: RM-ANOVA F = 4.85, p = .003
    - No interaction between treatment and week of assessment for healing: RM-ANOVA F = 1.12, p =.343F
- Treatment had no significant effect on healing rate after wound infection and dressing protocol were controlled: Cox regression = 1.34, p = .247
- Mean (SD) duration of trial participation, days: 67.5 (20.8) in Group 1 vs 75.0 (16.7) in Group 2, t = -1.45, p = .152
- Surface area changed significantly for both groups over 12 weeks: (RM-ANOVA F = 15.93, p <.001
    - No interaction between group and week of assessment for log surface area (RM-ANOVA F = 0.60, p = .618
- Significant change in log wound volume in both groups: RM-ANOVA F = 7.18, p <.001
    - Difference between groups: RM-ANOVA F = 1.72, p = .167
- Pain changed significantly over time in both groups: RM-ANOVA F = 8.16, p <.001
    - No interaction between group and week assessment for pain degree: RM-ANOVA F = 0.27, p = .847
- Group difference in the levels of exudate
    - Week 4: Fisher's exact test 1.59, p = .777
    - Week 8: Fisher's exact test 3.13, p = .478
    - Week 12: Fisher's exact test 2.43, p = .565
- For neither group did ankle (RM-ANOVA F = 0.25, p = .862) and calf diameter (RM-ANOVA F = 1.81, p = .149) change significantly
    - Group difference for ankle diameter: RM-ANOVA F = 1.24, p = 0.297
    - Group difference for calf diameter: RM-ANOVA F = 1.19, p = .316
- Square root of granulation did not change significantly for either group: RM-ANOVA F = 2.41, p = .070
    - No interaction between group and time: RM-ANOVA F = 0.10, p = .962
- Square root percentage of slough changed significantly in both groups: RM-ANOVA F = 5.05, p = .002
    - From baseline to 4 to 8 to 12 weeks: 8 to 6.5 to 5.0 to 5.3 in Group 1 vs 6.5 to 5.5 to 5.5 to 6.5 in Group 2, RM-ANOVA F = 2.76, p = .045
    - InteractionD between treatment and time was only significant in Group 1 and only between weeks 0 and 4 (t = 2.27, p = .025), 8 (t = 4.04, p <.001), and 12 (t = 3.21, p = .002)
- Mean (SD) no. of dressing changes per week, mean difference, 95% CI
    - Week 0: 2.07 (1.24) in Group 1 vs 2.41 (1.58) in Group 2, -0.33, -1.1 to 0.4, p = 391
    - Week 4: 2.00 (1.69) vs 2.30 (1.98), -0.30, -1.3 to 0.7, p = .556
    - Week 8: 1.33 (1.54) vs 1.89 (1.81), -0.56, -1.5 to 0.4, p = .230

For Peer Review

For Peer Review

    - Week 12: 1.11 (1.09) vs 2.48 (2.39), -1.37, -2.4 to -0.4, p = .009
- No. (%) with recurring ulcer during 12 week follow-up: 7/26 (27%) in Group 1 vs 6/25 (24%) in Group 2, chi squared = 0.10, p = 1.000
    - Group difference in residual ulcers: RM-ANOVA F = 0.45, p = .721
- During follow-up, more of Group 1 reported decrease in wound size, p = NS
    - There was a significant association between treatment and change in wound size at 4-weeks' follow-up in PP population: Fisher's exact test 9.69, p = .013
        - At 8 weeks: Fisher's exact test 6.30, p = .088
        - At 12 weeks: Fisher's exact test 3.14, p = .424
- No. subjects with AEs: 17/27 in Group 1 vs 8/27 in Group 2, chi squared = 6.03, p = .014
    - Group difference in severity of AEs: chi squared = 0.78, p = .714
    - Median (IQR) no. of AEs/subject: 1 (2) in Group 1 vs 0 (1) in Group 2, Fisher's exact test 6.90, p = .193
    - Most subjects in Group 1 experienced the AE for <24 hrs (14/22 reports) compared with 2-4 days in Group 2 (5/11 reports), Fisher's exact test 17.57, p = .002
    - 80% (20/25) of subjects in both groups with AE needed to reduce the dose of pharmaceutical intervention or cease treatment to resolve the symptom, no group difference: Fisher's exact test 2.64, p = .451
- Among the 33% (18/54) subjects not compliant with dressing protocol, reasons for protocol deviation did not differ significantly between groups: Fisher's exact test 4.54, p = .102
- Among the 39% (22/54) subjects not compliant with the intervention, factors reducing compliance were not different between groups: Fisher's exact test 5.41, p = .314.
- Most subjects in Group 1 (16/26) and Group 2 (19/27) uncertain which treatment they received during trial
    - Group difference in opinion about treatment received: Fisher's exact test 3.24, p = .214
    - Group difference in certainty of opinion: Fisher's exact test 1.33, p = .871

Power calculation:

- Was a power calculation for the primary endpoint reported, and if so, was it appropriate? Y, Y

Adjustment for multiplicity of statistical testing:

- Was an adjustment reported if there were more than 1 secondary endpoints statistically tested? N

Blinding:

- Were patients blinded from treatment? Y
- Were assessing investigators blinded from patient treatment? Y

Allocation concealment:

- Was allocation concealment carried out? N

Lost to follow-up/discontinued/withdrawn:

- 3/54 (5.6%) total
    - 1/27 (3.7%) withdrawn from Group 1

   - 2/27 (7.4%) withdrawn from Group 2

73. Leclere FM, Puechguiral IR, Rotteleur G, Thomas P, Mordon SR. A prospective randomized study of 980 nm diode laser-assisted venous ulcer healing on 34 patients. *Wound Repair Regen.* 2010;18(6):580-585.

Study Groups: Group 1: 980 nm diode laser; Group 2: control (SOC)

Statistical results:

- ITT analysis done for primary endpoint(s): Y
- **There were no primary endpoints identified.**
- Mean therapeutic time (except laser time), minutes: 9.8 in Group 1 vs 9.8 in group 2, Homogeneity p = .44 < p < 1
- Number of patients with complete healing: 3/18 in Group 1 vs 4/16 in group 2, Homogeneity p = .68
- Mean decrease in surface, %: 74.2 in Group 1 vs 94.3 in group 2, Homogeneity p = .60
   - 2 ulcers increased in surface in each Group, the increase was < 10 % of initial surface and the patients were not removed from the study. The frequency of increase in surface was not significant between both Groups, p = .67
- Mean VAS score between each therapy: 2.7 in Group 1 vs 3.8 in group 2, Homogeneity p = .13 < p < .86
   - Positive correlation between therapeutic time and ulcer size in both Groups for each session. The coefficient varies between .34 < r < .58, p = .0003 < p < .08
   - Mean VAS score (range): 1.8 (0.2-3.8) in Group 1 vs 3.8 (2.1-6.0) in Group 2, p = .08 < p < .67

Power calculation:

- Was a power calculation for the primary endpoint reported, and if so, was it appropriate? N

Adjustment for multiplicity of statistical testing:

- Was an adjustment reported if there were more than 1 secondary endpoints statistically tested? N

Blinding:

- Were patients blinded from treatment? N
- Were assessing investigators blinded from patient treatment? N

Allocation concealment:

- Was allocation concealment carried out? N

Lost to follow-up/discontinued/withdrawn:

- 1/34 (2.0%) total
   - 1/18 (5.6%) withdrawn from Group 1
   - 0/16 (0%) withdrawn from Group 2

74. Limova M. Evaluation of two calcium alginate dressings in the management of venous ulcers. *Ostomy Wound Manage.* 2003;49(9):26-33.

Study Groups: Group 1: 3M Tegagen HG High Gelling Alginate Dressing; Group 2: Sorbsan Topical Wound Dressing with alginate

For Peer Review

Statistical results:

- ITT analysis done for primary endpoint(s): N
- **There were no primary endpoints identified**
- Mean (range) of study days: 38.1 (21-42) in Group 1 vs 39.2 (28-42) in Group 2, p = NS
- Presence of foul odor, %: 15.7 in Group 1 vs 58.5 in Group 2, $p < .02$
- Presence of denuded periwound skin, %: 9.0 in Group 1 vs 31.9 in Group 2, $p < .04$
- Application of medication in denuded periwound skin, %: 31.3 in Group 1 vs 65.2 in Group 2, $p < .07$
- In Group 2, more wounds with medium to large amounts of exudate, purulent or serosanguinous drainage, necrotic tissue, or macerated periwound skin were observed, p = NS
- Wound assessment results with respect to mean improvement in wound condition score from baseline for the condition of the wounds edges, granular tissue, epithelialization, or necrotic tissue indicated no significant differences, p = NS
- Dressing assessment and performance): In Group 1 dressings, were rated significantly better than in Group 2, $p < .05$
- Average percent of dressing changes with adherence to wound bed also was significantly less in the Group 1, $p < .04$

Power calculation:

- Was a power calculation for the primary endpoint reported, and if so, was it appropriate? N

Adjustment for multiplicity of statistical testing:

- Was an adjustment reported if there were more than 1 secondary endpoints statistically tested? N

Blinding:

- Were patients blinded from treatment? N
- Were assessing investigators blinded from patient treatment? N

Allocation concealment:

- Was allocation concealment carried out? N

Lost to follow-up/discontinued/withdrawn:

- 1/20 (0.5%) total
  - 1/19 (0.5%) withdrawn from Group 1
  - 0/20 (0%) withdrawn from Group 2

75. Limova M, Troyer-Caudle J. Controlled, randomized clinical trial of 2 hydrocolloid dressings in the management of venous insufficiency ulcers. *J Vasc Nurs.* 2002;20(1):22-32.

Study Groups: Group 1: Hydrocolloid A (3M Tegasorb Hydrocolloid Dressing, 3M Health Care, St Paul, MN); Group 2: Hydrocolloid B (DuoDERM CGF, ConvaTec, Princeton, NJ)

Statistical results:

- ITT analysis done for primary endpoint(s): N
- **There were no primary endpoints identified.**
- Product-related dressing changes were performed: 2 in Group 1 vs 7 in Group 2, $p \leq .02$

For Peer Review

- Follow-up wound assessment: Average percentage of assessment, site 1; site 2; Total:
    - Debridement: 11 in Group 1 vs 0 in Group 2; 2 in Group 1 vs 13 in Group 2; 5 in Group 1 vs 10 in Group 2, p = .36, t = .94, df = 16, 12
    - Exudate amount- medium to large: 47 in Group 1 vs 83 in Group 2; 31 in Group 1 vs 46 in Group 2; 37 in Group 1 vs 55 in Group 2, p = .24, t = 1.22, df = 16, 12
    - Foul odor present: 2 in Group 1 vs 46 in Group 2; 4 in Group 1 vs 11 in Group 2; 3 in Group 1 vs 19 in Group 2, p = .05, t = 2.17, df = 16, 12
    - Exudate purulent: 2 in Group 1 vs 0 in Group 2; 0 in Group 1 vs 0 in Group 2; 1 in Group 1 vs 0 in Group 2, p = .33, t = 1.0, df = 16, 12
    - Macerated wound edges: 9 in Group 1 vs 54 in Group 2; 11 in Group 1 vs 11 in Group 2; 10 in Group 1 vs 21 in Group 2, p = .24, t = 1.20, df = 16, 12
    - Macerated peri-wound skin: 11 in Group 1 vs 54 in Group 2; 13 in Group 1 vs 25 in Group 2; 12 in Group 1 vs 32 in Group 2, p = .07, t = 1.88, df = 16, 12
    - Macerated peri-wound skin with medium to large amount of exudate: 15 in Group 1 vs 63 in Group 2; 24 in Group 1 vs 40 in Group 2; 20 in Group 1 vs 46 in Group 2, p = .10, t = 1.77, df = 10, 10
    - Necrotic tissue present: 13 in Group 1 vs 8 in Group 2; 7 in Group 1 vs 11 in Group 2; 9 in Group 1 vs 11 in Group 2, p = .88, t = 0.16, df = 16, 12
    - Eschar present: 0 in Group 1 vs 0 in Group 2; 0 in Group 1 vs 1 in Group 2; 0 in Group 1 vs 1 in Group 2, p = .34, t = 1.00, df = 16, 12
    - Skin irritation present: 30 in Group 1 vs 86 in Group 2; 39 in Group 1 vs 55 in Group 2; 36 in Group 1 vs 62 in Group 2, p = .05, t = 2.11, df = 16, 12
    - Eschar present: 0 in Group 1 vs 0 in Group 2; 0 in Group 1 vs 1 in Group 2; 0 in Group 1 vs 1 in Group 2, p = .34, t = 1.00, df = 16, 12
    - Mean of wound length at last visit, cm: 0.4 in Group 1 vs 0.9 in Group 2; 0.7 in Group 1 vs 2.2 in Group 2; 0.6 in Group 1 vs 1.9 in Group 2, p = .09, t = 1.84, df = 16, 12
    - Mean of wound width at last visit, cm: 0.4 in Group 1 vs 0.8 in Group 2; 0.3 in Group 1 vs 2.2 in Group 2; 0.3 in Group 1 vs 1.9 in Group 2, p = .15, t = 1.55, df = 16, 12
    - wound closure (100% of epithelialization): 57 in Group 1 vs 0 in Group 2; 60 in Group 1 vs 20 in Group 2; 59 in Group 1 vs 15 in Group 2, p = .03, Fisher exact test
    - Unscheduled dressing changes-product related: 29 in Group 1 vs 67 in Group 2; 20 in Group 1 vs 40 in Group 2; 12 in Group 1 vs 54 in Group 2, p = .02, Fisher exact test
- Mean change in wound length and width (prestudy to final visit):
    - Group 1:
        - Wound length, cm: -1.19, p = .0002, t = 4.74, df = 16
        - Wound width, cm: -1.03, p = .0001, t = 6.03, df = 16
    - Group 2:
        - Wound length, cm: -0.65, p = .048, t = 2.21, df = 12
        - Wound width, cm: -0.15, p = .657, t = 0.46, df = 12
- Interval dressing performance evaluations: %, site 1; site2, Total:

For Peer Review

  - Dressing remain in place: 98 in Group 1 vs 75 in Group 2; 100 in Group 1 vs 99 in Group 2; 99 in Group 1 vs 93 in Group 2, p = .32, t = 1.03, df = 16, 12
  - Dressing conformability to application site (good or satisfactory): 100 in Group 1 vs 100 in Group 2; 100 in Group 1 vs 100 in Group 2; 100 in Group 1 vs 100 in Group 2, statistics no computed, values are the same
  - Dressing absorbency of exudate (meets expectation or better than expected): 79 in Group 1 vs 42 in Group 2; 96 in Group 1 vs 84 in Group 2; 89 in Group 1 vs 74 in Group 2, p = .23, t = 1.23, df = 16, 12
  - Ease of dressing removal (just right, somewhat easy, or very easy): 94 in Group 1 vs 100 in Group 2; 98 in Group 1 vs 94 in Group 2; 96 in Group 1 vs 95 in Group 2, p = .88, t = 0.15, df = 16, 12
  - Patient discomfort upon dressing removal (no pain): 96 in Group 1 vs 100 in Group 2; 88 in Group 1 vs 70 in Group 2; 91 in Group 1 vs 77 in Group 2, p = .20, t = 1.34, df = 16, 12
  - Residue on skin after dressing removal: 6 in Group 1 vs 29 in Group 2; 25 in Group 1 vs 36 in Group 2; 18 in Group 1 vs 35 in Group 2, p = .15, t = 1.51, df = 16, 12
  - Ease of new dressing application: 100 in Group 1 vs 100 in Group 2; 100 in Group 1 vs 100 in Group 2; 100 in Group 1 vs 100 in Group 2, statistics no computed, values are the same
- Final clinical dressing performance evaluation, mean score (SD): (rating scale: 1 = very good, 2 = good, 3 = acceptable, 4 = poor, and 5 = very poor)
  - Ease of application: 1.5 (0.6) in Group 1 vs 2.2 (0.7) in group 2, p = .0148, t = 2.60, df = 16, 12
  - Adhesion to skin: 1.4 (0.6) in Group 1 vs 2.2 (0.7) in group 2, p = .0044, t = 3.10, df = 16, 12
  - Conformability: 1.5 (0.7) in Group 1 vs 2.2 (0.7) in group 2, p = .0079, t = 2.86, df = 16, 12
  - Exudate absorption: 1.9 (1.0) in Group 1 vs 3.1 (1.0) in group 2, p = .0034, t = 3.20, df = 16, 12
  - Barrier properties: 1.7 (0.9) in Group 1 vs 2.7 (1.2) in group 2, p = .0156, t = 2.57, df = 16, 12
  - Transparency: 2.0 (1.0) in Group 1 vs 4.7 (0.5) in group 2, p = .0001, t = 9.73, df = 16, 12
  - Adhesive residue: 2.5 (0.6) in Group 1 vs 3.1 (1.3) in group 2, p = .1454, t = 1.53, df = 16, 12
  - Patient comfort: 1.6 (0.8) in Group 1 vs 2.8 (1.2) in group 2, p = .0039, t = 3.15, df = 16, 12

Power calculation:

- Was a power calculation for the primary endpoint reported, and if so, was it appropriate? N

Adjustment for multiplicity of statistical testing:

- Was an adjustment reported if there were more than 1 secondary endpoints statistically tested? N

For Peer Review

Blinding:

- Were patients blinded from treatment? N
- Were assessing investigators blinded from patient treatment? N

Allocation concealment:

- Was allocation concealment carried out? N

Lost to follow-up/discontinued/withdrawn:

- 1/31 (9.7) total
  - 0/17 (0) withdrawn from Group 1
  - 3/14 (21.4%) withdrawn from Group 2

76. Mariani F, Mattaliano G, Mosti G, et al. The treatment of venous leg ulcers with a specifically designed compression stocking kit: comparison with bandaging. *Phlebologie.* 2008;37(4):191-197.

Study Groups: Group 1: Stocking system with understocking exerting moderate pressure and second stocking donned over first for day time use; Group 2: usual compression therapy

Statistical results:

- ITT analysis done for primary endpoint(s): N
- **Primary endpoints:**
  - Mean (%) prevalence
    - When applied: 0.00 (0.00)in Group 1 vs 0.23 (0.43) in Group 2, chi square = 2.76, p = .008
    - When removed: 0.00 (0.00)in Group 1 vs 0.20 (0.41) in Group 2, chi square = 2.50, p = .015
    - When walking: 0.15 (0.37)in Group 1 vs 0.43 (0.50) in Group 2, chi square = 2.34, p = .023
    - When wearing shoes: 0.08 (0.27)in Group 1 vs 0.27 (0.45) in Group 2, chi square = 1.87, p = .066
  - Mean (%) of pain at donning and removal: 0.00 (0.00)in group 1 vs 0.22 (0.31) in Group 2, chi square = 3.52, p = .001
  - Mean (%) of discomfort
    - When asleep: 0.00 (0.00)in Group 1 vs 0.57 (0.77) in Group 2, chi square = 3.73, p = .000
    - In the morning: 0.00 (0.00)in Group 1 vs 0.30 (0.65) in Group 2, chi square = 2.35, p = .023
    - In the afternoon: 0.62 (1.06)in Group 1 vs 0.63 (0.72) in Group 2, chi square = 0.07, p = .941
  - Mean (%) of scores of daytime discomfort, pain: 0.26 (0.39)in Group 1 vs 0.46 (0.45) in Group 2, chi square = 1.75, p = .08
  - Mean (%) of hindrance
    - Of normal activities: 0.58 (0.76)in Group 1 vs 1.23 (1.14) in Group 2, chi square = 2.50, p = .015
    - Of climbing stairs: 0.23 (0.43)in Group 1 vs 0.30 (0.47) in Group 2, chi square = 0.57, p = .568

For Peer Review

        - Of visiting friends: 0.12 (0.33)in Group 1 vs 0.23 (0.43) in Group 2, chi square = 1.14, p = .258
    - Mean (%) of scores of inhibition of activities: 0.35 (0.49)in group 1 vs 0.73 (0.73) in Group 2, chi square = 2.30, p = .025
    - Mean (%) of number of pain and discomfort problems: 1.31 (1.16)in group 1 vs 3.40 (1.65) in Group 2, chi square = 5.40, p = .000
    - Pain and daytime discomfort was associated with older age r = 0.27, p = 0.04; while younger age was strongly correlated with inhibition of activities r = -0.68, p <.0001
    - Complete healing within 4 months
        - Mean (%) of unhealed: 1 (3.8)in group 1 vs 9 (30.0) in Group 2, total n = 10 (17.9%), chi square = 6.49, p = .011
        - Mean (%) of healed: 25 (96.2)in group 1 vs 21 (70.0) in Group 2, total n = 46 (82.1%), chi square = 6.49, p = .011
    - Mean (SD) of time to heal, days: 56 (29.1)in group 1 vs 61.1 (22.7) in Group 2, total 58.4 (26.2), t = 0.65, p = .520
    - Mean (SD) no. of visits until healing.: 7.2 (4.6)in group 1 vs 9.1 (4.0) in Group 2, total 8.0 (4.4), t = 1.44, p = .157
    - Mean (SD) no. of intervals between visits: 8.2 (1.8)in group 1 vs 6.7 (1.0) in Group 2, total 7.5 (1.6), t = 3.28, p = .002
- Pearson correlation of healing (n = 56) and time to healing (n = 46) adjusted for age:
- -.31, p = .022; 0.33, p = .026
- Pearson correlation of healing (n = 56) and time to healing (n = 46) adjusted for sex: 0.08, p = .535; 0.10, p = .493
- Pearson correlation of healing (n = 56) and time to healing (n = 46) adjusted for PTS vs CVI: -0.01, p = .960; 0.40, p = .006
- Pearson correlation of healing (n = 56) and time to healing (n = 46) adjusted recurrence: -0.03, p = .849; 0.39, p = .007
- Pearson correlation of healing (n = 56) and time to healing (n = 46) adjusted for duration: -0.25, p = .064; 0.22, p = .136
- Pearson correlation of healing (n = 56) and time to healing (n = 46) adjusted for pain: -0.47, p = .000; 0.54, p = .000
- Pearson correlation of healing (n = 56) and time to healing (n = 46) adjusted for diameter: -0.15, p = .280; 0.54, p = .000
- Pearson correlation of healing (n = 56) and time to healing (n = 46) adjusted for ulcer ground: -0.20, p = .145; 0.47, p = .001
- Pearson correlation of healing (n = 56) and time to healing (n = 46) adjusted for exudation: -0.31, p = .022; 0.41, p = .004
- Pearson correlation of healing (n = 56) and time to healing (n = 46) adjusted for skin aspect: -0.27, p = .048; 0.45, p = .002

Power calculation:

- Was a power calculation for the primary endpoint reported, and if so, was it appropriate? N

For Peer Review

Adjustment for multiplicity of statistical testing:

- Was an adjustment reported if there were more than 1 secondary endpoints statistically tested? N

Blinding:

- Were patients blinded from treatment? N
- Were assessing investigators blinded from patient treatment? N

Allocation concealment:

- Was allocation concealment carried out? N

Lost to follow-up/discontinued/withdrawn:

- 9/60 (15.0%) total

77. Martinez ML, Escario E, Poblet E, et al. Hair follicle-containing punch grafts accelerate chronic ulcer healing: A randomized controlled trial. *J Am Acad Dermatol.* 2016;75(5):1007-1114.

Study Groups: Group 1: Hair grafting + SOC; Group 2: Graft without hair + SOC

Statistical results:

- ITT analysis done for primary endpoint(s): N
- **Primary endpoint:** Mean (SD) PAR at 18 weeks: 75.2% (23) in Group 1 vs 33.1% (46.2) in Group 2, p = .002
  - Mean reduction in total ulcer (both halves from each group together) was 55.7% (29.2), p <.001
  - Mean absolute reduction was 13 cm^2 (14.3), p = .013

Power calculation:

- Was a power calculation for the primary endpoint reported, and if so, was it appropriate? N

Adjustment for multiplicity of statistical testing:

- Was an adjustment reported if there were more than 1 secondary endpoints statistically tested? N

Blinding:

- Were patients blinded from treatment? N
- Were assessing investigators blinded from patient treatment? N

Allocation concealment:

- Was allocation concealment carried out? N

Lost to follow-up/discontinued/withdrawn:

- 1/12 (8.3%) total (note half of each ulcer randomized to each group, so each group had one less included in the analysis).

78. McDaniel JC, Szalacha L, Sales M, Roy S, Chafee S, Parinandi N. EPA + DHA supplementation reduces PMN activation in microenvironment of chronic venous leg ulcers: A randomized, double-blind, controlled study. *Wound Rep Regen.* 2017;25(4): 680-690.

Study Groups: Group 1: eicosapentaenoic acid (EPA) and docosahexaenoic acid (DHA); Group 2: placebo (opaque soft gels with 2.5 mL mineral oil)

For Peer Review

Statistical results:

- ITT analysis done for primary endpoint(s): N
- **Primary endpoint:** reductions in activated polymorphonuclear leukocytes (PMNs) counts in blood plasma, reductions in MPP-8 levels and human neutrophil elastase (HNE) levels, PAR on Day 28 and Day 56
- Mean (SD) of percentage of cells of Polymorphonuclear leukocytes (PMN) markers (CD15+ and CD66b+), during Day 0; Day 28; Day 56:
  - CD15+:
    - Blood:
      - Day 0: 42.74 (15.42) in Group 1 vs 46.05 (17.04) in Group 2, p = NS
      - Day 28: 41.64 (12.68), difference from Day 0, p = NS in Group 1 vs 42.77 (18.37) in Group 2, difference between Groups, p = NS
      - Day 56: 32.62 (24.44), difference from Day 0, p = .05, and Day 28, p = NS in Group 1 vs 41.38 (11.99) in Group 2, difference between Groups, p = NS
    - Wound fluid:
      - Day 0: 43.43 (32.58) in Group 1 vs 48.46 (24.17) in Group 2, p = NS
      - Day 28: 38.64 (31.26), difference from Day 0, p = NS in Group 1 vs 22.72 (19.41) in Group 2 (Difference Group 2 in day 0 and day 28, p = .004), difference between Groups, $p \leq .05$
      - Day 56: 21.17 (21.21), difference from Day 0, p = .04, and Day 28, p = NS in Group 1 vs 33.76 (32.51) in Group 2 (Difference Group 2 in day 0 and day 28, p = .03), difference between Groups, $p \leq .05$
  - CD66b+:
    - Blood:
      - Day 0: 2.45 (7.12) in Group 1 vs 8.87 (18.24) in Group 2, p = NS
      - Day 28: 2.72 (6.76), difference from Day 0, p = NS in Group 1 vs 8.25 (17.32) in Group 2, difference between Groups, p = NS
      - Day 56: 5.75 (16.35), difference from Day 0, p = NS, and Day 28, p = NS in Group 1 vs 5.60 (9.48) in Group 2, difference between Groups, p = NS
    - Wound fluid:
      - Day 0: 19.89 (20.71) in Group 1 vs 23.71 (25.82) in Group 2, p = NS
      - Day 28: 7.88 (11.90), difference from Day 0, p = NS in Group 1 vs 11.04 (18.90) in Group 2, difference between Groups, p = NS
      - Day 56: 4.23 (7.77), difference from Day 0, p = .02, and Day 28, p = .05 in Group 1 vs 15.49 (26.13) in Group 2, difference between Groups, p = NS
  - Ratio CD15+/CD66b+:
    - Blood:
      - Day 0: 57.45 (35.06) in Group 1 vs 49.96 (35.98) in Group 2, p = NS
      - Day 28: 50.27 (36.95), difference from Day 0, p = NS in Group 1 vs 69.85 (28.50) in Group 2, difference between Groups, p = NS

For Peer Review

            - Day 56: 47.57 (35.61), difference from Day 0, p = NS, and Day 28, p = NS in Group 1 vs 57.40 (34.01) in Group 2, difference between Groups, p = NS
        - Wound fluid:
            - Day 0: 48.44 (41.57) in Group 1 vs 58.71 (34.07) in Group 2, p = NS
            - Day 28: 44.87 (38.17), difference from Day 0, p = NS in Group 1 vs 43.26 (38.86) in Group 2, difference between Groups, p = NS
            - Day 56: 15.19 (28.21), difference from Day 0, p =.01, and Day 28, p = .06 in Group 1 vs 31.89 (41.00) in Group 2 (Difference Group 2 in day 0 and day 28, p = .03), difference between Groups, p ≤.05
- Mean (SEM) of levels of matrix metalloproteinase (MMP-8) and Human Neutrophil elastase (HNE) in wound fluid of venous leg ulcers, ng/mg of albumin (Group 1, n = 16; Group 2, n = 19) Students' t test:
    - MMP-8:
        - Day 0: 8.2 (4.0 - 8.2) in Group 1 vs 4.0 (2.6 - 5.5) in Group 2, p = NS
        - Day 28: 5.2 (2.1 - 3.8), difference from Day 0, p = NS in Group 1 vs 1.5 (0.5 - 2.2) in Group 2, difference between Groups, p = NS
        - Day 56: 3.0 (1.8 – 4.1), difference from Day 0, p = NS, and Day 28, p = NS in Group 1 vs 10.5 (7.0 - 14.2) in Group 2, difference between Groups, p = .09
    - HNE:
        - Day 0: 72 (44 - 104) in Group 1 vs 48 (27 - 62) in Group 2, p = NS
        - Day 28: 40 (23 - 55), difference from Day 0, p = NS in Group 1 vs 18 (10 - 27) in Group 2, difference between Groups, p = NS
        - Day 56: 30 (21 - 42), difference from Day 0, p = NS, and Day 28, p = NS in Group 1 vs 68 (43 - 95) in Group 2, difference between Groups, p = NS
    - Although the between group analyses of levels of MMP-8 and HNE at day0, 28, and 56, had no significant differences at the study time points the Cohen's effect size values for MMP-8 at day 56 (d = 0.63), and HNE at day 56 (d = 0.80) indicate a medium to large treatment effect that maybe clinically meaningful.
- Mean (SEM) of percentage of reduction wound area (Group 1, n = 16; Group 2, n = 19) Students' t test:
    - Day 28: 56 (49 - 67) in Group 1 vs 34 (27 - 44) in Group 2, p = .09
    - Day 56: 74 (67 - 83), difference from Day 28, p = NS in Group 1 vs 59 (51 - 68) in Group 2, difference between Groups, p = .17
    - Between-group analysis indicated that the mean percent reduction in wound area was greater at both day 28 and 56 in Group 1, but differences were not significant (p = .09 at day 28), the Cohen's effect size value (d= 0.60 at 28 days and d = 0.50 at 56 days) suggest a medium practical significance
    - The within-group analysis of wound healing in Group 1 detected a significant increase in percent reduction of wound area at day 28 vs day 0 (t = 6.304, p <.001), at day 56 vs Day 0 (t = 9.228, p <.001), and day 56 vs day 28 (t = 2.951, p = .01.), Similar pattern in Group 2, day 28 vs day 0 (t = 4.005, p = .001), at day 56

For Peer Review

vs Day 0 (t = 7.032, p <.001), and day 56 vs day 28 (t = 2.858, p = .01.)

- Spearman's correlation between plasma fatty acids, markers (independent variables) at day 28, and 56 vs (Dependent variable) Percentage of reduction wound area (p value of correlation coefficient) at day 28 and day 56, in total patients in both Groups , n = 35:
    - EPA-DHA:
        - Day 28: PAR at day 28 = 0.27 (p = .13), and PAR at day 56 = 0.20 (p = .25)
        - Day 56: PAR at day 28 = 0.34 (p = .05), and PAR at day 56 = 0.25 (p = .16)
    - EPA:
        - Day 28: PAR at day 28 = 0.18 (p = .32), and PAR at day 56 = 0.30 (p = .09)
        - Day 56: PAR at day 28 = 0.28 (p = .10), and PAR at day 56 = 0.28 (p = .11)
    - DHA:
        - Day 28: PAR at day 28 = 0.35 (p = .045), and PAR at day 56 = 0.18 (p = .30)
        - Day 56: PAR at day 28 = 0.34 (p = .046), and PAR at day 56 = 0.24 (p = .16)
    - Total n-3 PUFA's:
        - Day 28: PAR at day 28 = 0.31 (p = .07), and PAR at day 56 = 0.23 (p = .18)
        - Day 56: PAR at day 28 = 0.36 (p = .03), and PAR at day 56 = 0.28 (p = .11)
    - CD15 + cells in WF:
        - Day 28: PAR at day 28 =- 0.18 (p = .33), and PAR at day 56 = -0.53 (p = .003)
        - Day 56: PAR at day 28 = -0.45 (p = .008), and PAR at day 56 =- 0.33 (p <.001)
    - CD66b + cells in WF:
        - Day 28: PAR at day 28 =- 0.28 (p = .14), and PAR at day 56 = -0.45 (p = .01)
        - Day 56: PAR at day 28 = -0.35 (p = .04), and PAR at day 56 =- 0.45 (p = .009)
    - CD66b+/ CD15 + cells in WF:
        - Day 28: PAR at day 28 =- 0.15 (p = .43), and PAR at day 56 = -0.44 (p = .02)
        - Day 56: PAR at day 28 = -0.35 (p = .05), and PAR at day 56 =- 0.43 (p = .01)
    - MMP-8:
        - Day 28: PAR at day 28 =- 0.21 (p = .30), and PAR at day 56 = -0.36 (p = .06)
        - Day 56: PAR at day 28 = -0.29 (p = .17), and PAR at day 56 =- 0.41 (p = .04)
    - HNE:
        - Day 28: PAR at day 28 =- 0.28 (p = .29), and PAR at day 56 = -0.02 (p = .96)
        - Day 56: PAR at day 28 = -0.28 (p = .23), and PAR at day 56 =- 0.22 (p = .34)
- Mean (SD) of percentage of amounts of plasma fatty acids, during Day 0; Day 28; Day 56 (Group 1 n = 16; Group 2 , n = 19), analysis Student's t test:
    - EPA:
        - Day 0: 0.52 (0.22) in Group 1 vs 0.52 (0.26) in Group 2, p = NS
        - Day 28: 2.21 (1.25), difference from Day 0, p <.05 in Group 1 vs 0.46 (0.24) in Group 2, difference between Groups, p <.05
        - Day 56: 2.33 (0.98), difference from Day 0, p <.05, and Day 28, p <.05 in Group 1 vs 0.48 (0.25) in Group 2, difference between Groups, p <.05
            - Plasma PUFA levels were significantly higher for EPA at day 28 (t =

For Peer Review

5.9, p <.001) and at day 56 (t = 7.6, p <.001), related to day 0

- DHA:
  - Day 0: 1.47 (0.59) in Group 1 vs 1.50 (0.44) in Group 2, p = NS
  - Day 28: 2.77 (0.84), difference from Day 0, p <.05 in Group 1 vs 1.46 (0.50) in Group 2, difference between Groups, p <.05
  - Day 56: 3.09 (0.99), difference from Day 0, p <.05, and Day 28, p <.05 in Group 1 vs 1.49 (0.52) in Group 2, difference between Groups, p <.05
    - Plasma Polyunsaturated fatty acids (PUFA) levels were significantly higher for DHA at day 28 (t = 5.3, p <.001) and at day 56 (t = 6.8, p <.001), related to day 0
- AA (Arachidonic acid):
  - Day 0: 7.83 (2.45) in Group 1 vs 8.38 (2.00) in Group 2, p = NS
  - Day 28: 6.82 (1.83), difference from Day 0, p <.05 in Group 1 vs 8.37 (2.49) in Group 2, difference between Groups, p <.05
  - Day 56: 6.80 (2.16), difference from Day 0, p <.05, and Day 28, p <.05 in Group 1 vs 8.83 (2.61) in Group 2, difference between Groups, p <.05
- Total n-6:
  - Day 0: 38.02 (4.32) in Group 1 vs 37.46 (3.81) in Group 2, p = NS
  - Day 28: 35.47 (4.78), difference from Day 0, p = NS in Group 1 vs 36.51 (4.68) in Group 2, difference between Groups, p = NS
  - Day 56: 35.99 (5.77), difference from Day 0, p = NS, and Day 28, p = NS in Group 1 vs 37.27 (4.10) in Group 2, difference between Groups, p = NS
    - Plasma PUFA levels n-6 were significantly lower for AA at day 28 (t = 2.9, p =.01) and at day 56 (t = 3.4, p <.001), related to day 0
- Total n-3:
  - Day 0: 2.89 (0.55) in Group 1 vs 3.05 (0.71) in Group 2, p = NS
  - Day 28: 6.16 (1.83), difference from Day 0, p <.05 in Group 1 vs 2.88 (0.50) in Group 2, difference between Groups, p <.05
  - Day 56: 6.55 (1.61), difference from Day 0, p <.05, and Day 28, p <.05 in Group 1 vs 2.99 (0.67) in Group 2, difference between Groups, p <.05
- Ratio AA: EPA:
  - Day 0: 13.75 (6.66) in Group 1 vs 16.28 (8.05) in Group 2, p = NS
  - Day 28: 4.19 (3.27), difference from Day 0, p <.05 in Group 1 vs 15.39 (7.70) in Group 2, difference between Groups, p <.05
  - Day 56: 3.46 (1.80), difference from Day 0, p <.05, and Day 28, p <.05 in Group 1 vs 15.81 (8.40) in Group 2, difference between Groups, p <.05
    - Plasma PUFA levels were significantly lower for ratio AA:EPA at day 28 (t = 4.8, p =.001) and at day 56 (t = 7.1, p <.001), related to day 0
- Ratio n-6: n-3:
  - Day 0: 15.21 (6.96) in Group 1 vs 12.69 (2.14) in Group 2, p = NS
  - Day 28: 8.35 (8.62), difference from Day 0, p <.05 in Group 1 vs 12.90 (2.29) in Group 2, difference between Groups, p <.05

For Peer Review

        - Day 56: 8.04 (8.83), difference from Day 0, p <.05, and Day 28, p <.05 in Group 1 vs 12.91 (2.62) in Group 2, difference between Groups, p <.05
    - This results suggesting that the EPA study dose resulted in significantly increased proportions of EPA in Plasma that occurred partly at expenses of AA.

Power calculation:

- Was a power calculation for the primary endpoint reported, and if so, was it appropriate? Y, Y

Adjustment for multiplicity of statistical testing:

- Was an adjustment reported if there were more than 1 secondary endpoints statistically tested? N

Blinding:

- Were patients blinded from treatment? Y
- Were assessing investigators blinded from patient treatment? Y

Allocation concealment:

- Was allocation concealment carried out? N

Lost to follow-up/discontinued/withdrawn:

- 5/40 (12.5%) total
    - 5/21 (23.8%) withdrawn from Group 1
    - 0/19 (0%) withdrawn from Group 2

79. Meaume S, Truchetet F, Cambazard F, et al. A randomized, controlled, double-blind prospective trial with a Lipido-Colloid Technology-Nano-OligoSaccharide Factor wound dressing in the local management of venous leg ulcers. *Wound Repair Regen.* 2012;20(4):500-511.

Study Groups: Group 1: Lipido-colloid technology dressing impregnated with nano-oligosaccharide factor; Group 2: 1: Lipido-colloid technology dressing impregnated without nano-oligosaccharide factor

Statistical results:

- ITT analysis done for primary endpoint(s): Y
- **Primary endpoint:** Mean (SD) and median (range) of relative WAR, %: -45.2 (47.9) and -58.3 (-100.0; 173.0) in Group 1 vs -21.4 (81.0) and -31.6 (-100.0; 571.0) in Group 2, p = 0.002; 95% CI for median difference: -38.3 to -15.1, p = 0.002
    - Mean (SD) and median (range) of absolute PAR, cm^2: -6.9 (11.4) and -6.1 (-55.5; 31.7) in Group 1 vs -2.2 (11.9) and -3.2 (-33.1; 74.4) in Group 2, p = 0.003
    - PAR >40%, %: 65.6 in Group 1 vs 39.4 in Group 2, OR = 2.9, 95% CI = 1.6; 5.3, p = .0003
    - Median time to reach PAR >40%, days (95% CI): 43 (37.2-48.8) in Group 1 vs 63 (57.8-68.1) in Group 2, p = .002
    - Relative PAR ≥60%: OR = 2.2, 95% CI = 1.2; 4.0, p = .013
- Efficacy outcomes in patients, Group 1 n= 93, Group 2 n= 94
    - Mean (SD) and median (range) of wound edge progression , mm: -1.15 (1.20) and -1.15 (-3.96; 2.20) in Group 1 vs -0.56 (1.19) and -056 (-3.43; 6.97) in Group 2, p = 0.001

For Peer Review

  - Mean (SD) and median (range) of heal rate, mm^2/ day: -13.32 (24.56) and -10.83 (-158.32; 57.59) in Group 1 vs -4.54 (23.20) and -5.15 (-60.19; 132.87) in Group 2, p = 0.005
- Using a binary logistic regression method that includes basal wound area (10 cm^2 cutoff), ulcer recurrence (yes/no), and duration (<1, 1-2, and >2 years) in the model, OR of PAR >40% is 3.3 (95% CI: 18.8-6.1, p <.001) in favor of Group 1. Only the basal area was significant in this model (OR area < 10 cm^2 vs ≥ 10 cm^2: 2.1; 95% CI: 1.1-4.0, p = .020)
- 81.4% (70/86) ulcers were considered improved in the Group 1 vs 65.9% (54/82) in Group 2 , p = .022

Power calculation:

- Was a power calculation for the primary endpoint reported, and if so, was it appropriate? Y, N

Adjustment for multiplicity of statistical testing:

- Was an adjustment reported if there were more than 1 secondary endpoints statistically tested? N

Blinding:

- Were patients blinded from treatment? Y
- Were assessing investigators blinded from patient treatment? N

Allocation concealment:

- Was allocation concealment carried out? Y

Lost to follow-up/discontinued/withdrawn:

- 10/187 (5.3%) total
  - 4/93 (4.3%) withdrawn from Group 1
  - 6/94 (6.4%) withdrawn from Group 2

80. Meyer FJ, Burnand KG, Lagattolla NR, Eastham D. Randomized clinical trial comparing the efficacy of two bandaging regimens in the treatment of venous leg ulcers. *Br J Surg.* 2002; 89(1):40-44.

Study Groups: Group 1: elastic bandaging system; Group 2: inelastic bandaging system

Statistical results:

- ITT analysis done for primary endpoint(s): N
- **Primary endpoints:**
  - No. (%) Complete healing within 26 weeks: 33 (58) in Group 1, n= 57 vs 34(62) in Group 2, n= 55, chi square = 0.23, p = .623
  - Mean time to heal (range, 95% CI, median), week: 10 (2-23, 8-12, 9) in Group 1 vs 11 (3-25, 9-13, 9.5) in Group 2, U = 560.5, p = NS
  - No. (%) Complete healing within 40 weeks: 34 (60) in Group 1 vs 35(64) in Group 2, p = NS
  - Patients with large venous ulcer were significantly less likely to heal within 26 weeks than patients with small or medium-sized ulcers, chi square = 18.05, p <.001. This effect was not dependent on the type of bandage used.

Power calculation:

For Peer Review

- Was a power calculation for the primary endpoint reported, and if so, was it appropriate? N

Adjustment for multiplicity of statistical testing:

- Was an adjustment reported if there were more than 1 secondary endpoints statistically tested? N

Blinding:

- Were patients blinded from treatment? N
- Were assessing investigators blinded from patient treatment? N

Allocation concealment:

- Was allocation concealment carried out? N

Lost to follow-up/discontinued/withdrawn:

- 25/112 (22.3%) total
    - 12/57 (21%) withdrawn from Group 1
    - 13/55 (23.6%) withdrawn from Group 2

81. Meyer FJ, McGuinness CL, Lagattolla NR, Eastham D, Burnand KG. Randomized clinical trial of three-layer paste and four-layer bandages for venous leg ulcers. *Br J Surg.* 2003;90(8):934-940.

Study Groups: Group 1: 3-layer paste bandages; Group 2: 4-layer bandages

Statistical results:

- ITT analysis done for primary endpoint(s): Y
- **Primary endpoint:** complete ulcer healing and time to heal
    - No. (%) healed at 52 weeks: 51/64 (80%) in Group 1 vs 45/69 (65%) in Group 2, p = .031
    - Median (95% CI) times to heal, weeks: 12 (10-15) in Group 1 vs 16 (13-21) in Group 2, p = .040
        - For ITT, group difference in time to heal was p = .036 (data not shown)
- Large venous ulcers were significantly less likely to heal than small/medium ulcers, p <.05
- In both groups, ankle circumference decreased, while scores for comfort, pain on bandaging, and ease of putting shoes over bandages increased over time, p = NS
- Mean time to apply bandages, minutes: 4.6 in Group 1 vs 5.5 in Group 2, p = .008
    - Group 1 was not significantly quicker to apply at any given time point, p = NS
- % with post-thrombotic calf veins: 79% in Group 1 vs 54% in Group 2, p = .042
- % with postthrombotic changes in the proximal veins: 38% in Group 1 vs 47% in Group 2, p = NS
- Mean below-knee half-refilling times, seconds: 11.7 in both groups, p =NS
- Presence of previous thrombosis or popliteal reflux did not significantly influence healing (p = NS): no thrombosis 87% healed, postthrombotic 73% healed, no popliteal reflux 85% healed, popliteal reflux 75% healed

Power calculation:

- Was a power calculation for the primary endpoint reported, and if so, was it appropriate? Y, Y

Adjustment for multiplicity of statistical testing:

- Was an adjustment reported if there were more than 1 secondary endpoints statistically tested? N

Blinding:

- Were patients blinded from treatment? N
- Were assessing investigators blinded from patient treatment? N

Allocation concealment:

- Was allocation concealment carried out? N

Lost to follow-up/discontinued/withdrawn:

- 15/133 (31.2%) total
    - 10/64 (15.6%) withdrawn from Group 1
    - 11/69 (15.9%) withdrawn from Group 2

82. Michaels JA, Campbell B, King B, Palfreyman SJ, Shackley P, Stevenson M. Randomized controlled trial and cost-effectiveness analysis of silver-donating antimicrobial dressings for venous leg ulcers (VULCAN trial). *Br J Surg*. 2009;96(10):1147-1156.

Study Groups: Group 1: silver-donating dressing; Group 2: non-antimicrobial (control) dressing

Statistical results:

- ITT analysis done for primary endpoint(s): N
- **Primary endpoint:** % (no.) (95% CI) of ulcers healed at 12 weeks: 59.6 (62/104) (50.2 to 69.1) in Group 1 (n = 107) vs 56.7 (59/104) (47.2 to 66.3) in Group 2 (n = 106), and 58.2 (121/208) (51.5 to 64.9) in Total (n = 213), p = .673
    - The RR of healing for the Group 1 vs Group 2 at 12 weeks: 1.06 (95% CI = 0.80 to 1.40), p = NS
- Percentage (no.) (95% CI) of ulcers healed at 6 months: 85.3 (87/102) (78.4 to 92.2) in Group 1 (n = 107) vs 77.2 (78/101) (69.1 to 85.4) in Group 2 (n = 106), and 81.3 (165/203) (75.9 to 86.7) in Total (n = 213), p = .141
    - The RR of healing for the Group 1 vs Group 2 at 6 months : 1.34 (95% = CI 0.88 to 2.03), p = NS
- Percentage (no.) (95% CI) of ulcer healed at 1 year: 96 (95/99) (92 to 100) in Group 1 (n = 107) vs 96 (90/94) (92 to 100) in Group 2 (n = 106), and 95.9 (185/193) (93.0 to 98.7) in Total (n = 213), p = .940
    - The RR of healing for the Group 1 vs Group 2 at 1 year: 1.03 (95% CI = 0.51 to 2.08), p = NS
- Overall median time to heal, days: 67 (95% CI = 54 to 80) in Group 1 vs 58 (95% CI = 43 to 73), p = .408
    - Large ulcers healed more slowly than small ulcers, media of 101 days (95% CI = 43 to 73) and 52 (46 to 57), respectively for those above 3 cm and up to 3 cm in initial diameter, p = 0.050
- Results of the binary logistic regression analysis to predict healing
    - Coefficient (SE, OR) of dressing: 0.074 (0.296, 1.077), p = NS
    - Coefficient (SE, OR) of Co-morbidity: 0.222 (0.362, 1.248), p = NS
    - Coefficient (SE, OR) of location: -0.675 (0.307, 0.509), p = .028

For Peer Review

  - Coefficient (SE, OR) of age:- 0.018 (0.012, 0.982), p = NS
  - Coefficient (SE, OR) of sex: 0.768 (0.309, 2.156), p = .013
  - Coefficient (SE, OR) of ulcer size: -0.855 (0.322, 0.4259), p = .008
  - Coefficient (SE, OR) of Constant: 1.579 (0.836, 4.852), p = NS
- Health-related quality of life
  - Calculation of the mean single index utility score, EQ-5D and SF-6D, showed no significant differences between the Groups at any of the follow-up times, p = NS
  - Mean (n) of EQ-5D health state values:
    - at baseline: 0.6446 (98) in Group 1 vs 0.6536 (94) in Group 2, difference 0.0090, p = NS
    - at 1 month: 0.6963 (82) in Group 1 vs 0.6973 (72) in Group 2, difference 0.0010, p = NS
    - at 3 months: 0.7255 (81) in Group 1 vs 0.7004 (76) in Group 2, difference -0.0251, p = NS
    - at 6 months: 0.7214 (70) in Group 1 vs 0.6808 (64) in Group 2, difference -0.0406, p = NS
    - at 12 months: 0.7526 (61) in Group 1 vs 0.6752 (58) in Group 2, difference -0.0774, p = NS
  - Mean (n) of SF-6D health state values:
    - at baseline: 0.6544 (89) in Group 1 vs 0.6792 (83) in Group 2, difference 0.0248, p = NS
    - at 1 month: 0.68293 (74) in Group 1 vs 0.7016 (67) in Group 2, difference 0.0187, p = NS
    - at 3 months: 0.6864 (73) in Group 1 vs 0.7029 (68) in Group 2, difference 0.0165, p = NS
    - at 6 months: 0.6890 (67) in Group 1 vs 0.6764 (49) in Group 2, difference -0.0126, p = NS
    - at 12 months: 0.7092 (55) in Group 1 vs 0.6662 (53) in Group 2, difference -0.0430, p = NS
- Cost-effectiveness analysis was based on the responses of 141 participants who provided EQ-5D at 3 months: 74 in Group 1 vs 67 in Group 2. Differences between these patients and those who did not return their questionnaires showed that the self-selected group had a higher proportion of patients who ulcer had healed at 12 weeks, chi-square= 6.14, d.f. = 1, p = .013
- Group 1 had significantly more visits than Group 2 (8.00 vs 5.61, respectively), p <.05
- The number of community nurse home visits, general practitioner contacts, chiropody contacts, prescriptions for hosiery, antibiotics, and other medicines showed no significant differences between groups (p = NS)
- The biggest contributor to total cost for both groups was the cost of attending ulcer clinics, accounting for between 61% and 66% of total cost.
  - Ulcer costs were significantly higher in the group 1 vs Group 2, p <.05
  - Mean cost per clinic visit was £275.39 (95% CI = 236.83 to 313.95) in Group 1 vs £196.06 (95% CI = 156.95 to 235.18) in Group 2, p <.05

For Peer Review

- Mean cost of dressing was £30.62 (95% CI = 25.47 to 35.78) in Group 1 vs £5.73 (95% CI = 2.96 to 8.49) in Group 2, $p < .05$
- Mean total cost per patient was £417.97 (95% CI = 375.01 to 460.93) in Group 1 vs £320.12 (95% CI = 277.42 to 362.82) in Group 2, $p < .05$

Power calculation:

- Was a power calculation for the primary endpoint reported, and if so, was it appropriate? Y, Y

Adjustment for multiplicity of statistical testing:

- Was an adjustment reported if there were more than 1 secondary endpoints statistically tested? N

Blinding:

- Were patients blinded from treatment? N
- Were assessing investigators blinded from patient treatment? N

Allocation concealment:

- Was allocation concealment carried out? N

Lost to follow-up/discontinued/withdrawn:

- 13/213 (6.1%) total
    - 8/107 (7.5%) withdrawn from Group 1
    - 5/106 (4.7%) withdrawn from Group 2

83. Milic DJ, Zivic SS, Bogdanovic DC, et al. A randomized trial of the Tubulcus multilayer bandaging system in the treatment of extensive venous ulcers. *J Vasc Surg.* 2007;46(4):750-755.

Study Groups: Group 1: multilayer bandaging system comprised of a tubular compression device and medium-stretch compression bandage; Group 2: 2 elastic medium-stretch compression bandages (control)

Statistical results:

- ITT analysis done for primary endpoint(s): N
- **Primary endpoint:** Complete ulcer healing at 500 days: 93% in Group 1 vs 51% in Group 2, $p < .01$
- Recurrence rate during 1-year follow-up period: 24% (16/67) in Group 1 vs 53% (18/34) in Group 2, $p < .05$.

Power calculation:

- Was a power calculation for the primary endpoint reported, and if so, was it appropriate? Y, N

Adjustment for multiplicity of statistical testing:

- Was an adjustment reported if there were more than 1 secondary endpoints statistically tested? N

Blinding:

- Were patients blinded from treatment? N
- Were assessing investigators blinded from patient treatment? N

Allocation concealment:

- Was allocation concealment carried out? N

For Peer Review

Lost to follow-up/discontinued/withdrawn:

- 12/150 (8.0%) total
    - 3/75 (4.0%) withdrawn from Group 1
    - 9/75 (12.0%) withdrawn from Group 2

84. Milio G, Mina C, Cospite V, Almasio PL, Novo S. Efficacy of the treatment with prostaglandin E-1 in venous ulcers of the lower limbs. *J Vasc Surg.* 2005;42(2):304-308.

Study Groups: Group 1: Prostaglandin E-1; Group 2: placebo venous infusion of a saline solution

Statistical results:

- ITT analysis done for primary endpoint(s): N
- **Primary endpoint:** 100% healed in Group 1 (40/40) vs 84.2% in Group 2 (32/38) (p <.05)
- Estimated time of healing of 25%, 50%, and 75% for Group 1 was 23, 49, and 72 days, respectively, vs 52, 80, and 108 days for Group 2 (p <.001)
- Median ulcer area (IQR), cm^2: 0 days: 13.4 (3.2-25.7) in Group 1 vs 11.7 (4.0-20.6) in Group 2 (p = NS); 20 days: 11.2 (2.9-22.3) in Group 1 vs 10.1 (3.8-19.8) in Group 2 (p = NS); 40 days: 8.3 (3.1-17.1) vs 9.7 (3.6-17.3) (p <.05); 60 days: 6.1 (2.7-12.1) vs 8.9 (3.5-15.4) (p <.05); 80 days: 3.2 (2.0-8.6) vs 7.1 (3.2-14.5), (p<.05); 100 days: 5.2 (2.9-11.4) in Group 1; 120 days: 3.9 (1.9-8.6) in Group 1

Power calculation:

- Was a power calculation for the primary endpoint reported, and if so, was it appropriate? Y, N

Adjustment for multiplicity of statistical testing:

- Was an adjustment reported if there were more than 1 secondary endpoints statistically tested? N.

Blinding:

- Were patients blinded from treatment? Y
- Were assessing investigators blinded from patient treatment? N

Allocation concealment:

- Was allocation concealment carried out? N

Lost to follow-up/discontinued/withdrawn:

- Unclear for totals.
    - 5/44 (11.4%) withdrawn from Group 1
    - 4/43 (9.3%) withdrawn from Group 2
    - Also report that 3 withdrew consent and 4 lost to follow-up but unclear in which groups they belonged

85. Moffatt CJ, Edwards L, Collier M, et al. A randomised controlled 8-week crossover clinical evaluation of the 3M Coban 2 Layer Compression System versus Profore to evaluate the product performance in patients with venous leg ulcers. *Int Wound J.* 2008;5(2):267-279.

Study Groups: Group 1: 2-layer compression bandage system; Group 2: 4-layer compression bandage system; both groups crossed over to other bandage system at 4 weeks

Statistical results:

For Peer Review

- ITT analysis done for primary endpoint(s): Y.
- **Primary endpoint:** Mean bandage slippage (SEM), cm
    - Day 1: 2.3 (1.8-2.8) in Group 1 vs 2.8 (2.3-3.3) in Group 2, p = NS
    - Day 2: 2.3 (1.9-2.75) vs 3.1 (2.6-3.5), p = NS
    - Day 3: 2.4 (2.6-2.8) vs 3.4 (3.0-3.8), p <.001
    - Day 4: 2.4 (2.05-2.8) vs 3.7 (3.4-4.05), p <.001
    - Day 5: 2.5 (2.1-2.8) vs 4.0 (3.7-4.4), p <.001
    - Day 6: 2.5 (2.2-2.8) vs 4.3 (4.0-4.6), p <.001
    - Day 7: 2.5 (2.2-2.85) vs 4.6 (4.3-5.0), p <.001
- Wear times, days: 5.72 in Group 1 vs 5.75 in Group 2, p = .721
- There were no significant differences in any of the wound healing endpoints, p = NS (data not shown)
- Of the 9/79 wounds (11.4%) that healed prior to crossover, 6 were in Group 1 and 3 in Group 2, p = .30
- Median (range) PAR: 27.8% (-233.3 to 100.0) in Group 1 vs 42.2% (-272.1 to 100.0) in Group 2, p = .88
- Median (range) linear healing rate, cm/week: 0.04 (-0.16 to 0.40) in Group 1 vs 0.04 (-0.27 to 0.19) in Group 2, p = .94
- Mean (SD) changes in HRQoL patient scores during the pre-crossover period
    - Well-being: 4.1 (16.0) in Group 1 vs 3.4 (13.3) in Group 2
        - Mean (SD) group difference, 95% CI: 0.74 (14.6), -6.0 to 7.5, p = .827
    - Physical symptoms and daily living: 8.7 (17.9) in Group 1 vs 1.2 (13.8) in Group 2
        - Mean (SD) group difference, 95% CI: 7.5 (15.7), 0.1 to 14.9, p = .046
    - Social life: 6.8 (15.2) in Group 1 vs 1.3 (14.9) in Group 2
        - Mean (SD) group difference, 95% CI: 5.6 (15.1), -1.4 to 12.6, p = .118
    - Overall HRQoL: 0.6 (2.0) in Group 1 vs 0.2 (1.8) in Group 2
        - Mean (SD) group difference, 95% CI: 0.4 (1.9), -0.5 to 1.3, p = .376
    - Patient satisfaction with overall HRQoL: 0.4 (2.4) in Group 1 vs 0.3 (2.3) in Group 2
        - Mean (SD) group difference, 95% CI: 0.1 (2.4), -1.0 to 1.2, p = .860
- Mean (SD) changes in HRQoL patient scores during the post-crossover period
    - Well-being: 1.5 (14.5) in Group 1 vs -2.9 (14.5) in Group 2
        - Mean (SD) group difference, 95% CI: -4.4 (14.5), -12.1 to 3.3, p = .258
    - Physical symptoms and daily living: 2.8 (12.9) in Group 1 vs -2.6 (16.4) in Group 2
        - Mean (SD) group difference, 95% CI: -5.4 (14.5), -13.2 to 2.4, p = .169
    - Social life: -1.8 (13.6) in Group 1 vs -5.0 (19.7) in Group 2
        - Mean (SD) group difference, 95% CI: -3.2 (16.4), -12.1 to 5.7, p = .478
    - Overall HRQoL: 0.6 (1.2) in Group 1 vs 0.3 (1.9) in Group 2
        - Mean (SD) group difference, 95% CI: -0.3 (1.5), -1.1 to 0.5, p = .494
    - Patient satisfaction with overall HRQoL: 0.5 (1.5) in Group 1 vs -0.4 (2.8) in Group 2
        - Mean (SD) group difference, 95% CI: -0.9 (2.2), -2.1 to 0.3, p = .172
- Patient preference, n = 68: 49 (72%) preferred 2-layer compression, 15 (22%) preferred

For Peer Review

4-layer compression, and 4 (6%) had no preference, with results similar regardless of randomization order p >0.99

- There was no significant difference in mobility between times that the subjects wore the 2- and 4-layer bandages (p = .82)
  - Analysis of the data from the single, more reliable site confirmed this (p = .23)

Power calculation:

- Was a power calculation for the primary endpoint reported, and if so, was it appropriate? Y, Y

Adjustment for multiplicity of statistical testing:

- Was an adjustment reported if there were more than 1 secondary endpoints statistically tested? N

Blinding:

- Were patients blinded from treatment? N
- Were assessing investigators blinded from patient treatment? N

Allocation concealment:

- Was allocation concealment carried out? N

Lost to follow-up/discontinued/withdrawn:

- 6/81 (7.4%) total
  - 5/39 (12.8%) withdrawn from Group 1
  - 1/42 (2.4%) withdrawn from Group 2

86. Moffatt CJ, McCullagh L, O'Connor T, et al. Randomized trial of four-layer and two-layer bandage systems in the management of chronic venous ulceration. *Wound Repair Regen.* 2003;11(3):166-171.

Study Groups: Group 1: 4-layer bandage system; Group 2: 2-layer bandage system

Statistical results:

- ITT analysis done for primary endpoint(s): N
- **Primary endpoint:** Over 24 weeks, the HR for complete closure was 1.19 (95% CI: 0.69-2.02, p = .55
  - Closure at 12 weeks: 40 (70%) in Group 1 vs 30 (58%) in Group 2, OR = 4.23, 95% CI: 1.29-13.86, p = .02
  - At 24 weeks: 47 (82%) in Group 1 vs 24 (46%) in Group 2, difference = 36%, 95% CI: 18-55, p <.001
- Among 112 patients followed up on at 24 weeks, 5 (9%) in Group 1 had device-related AEs vs 15 (28%) in Group 2, p = .01
- Among 28 withdrawn from Group 2, 16 (57%) healed at 24 weeks; among the 7 withdrawn from Group 1, 3 (43%) healed, p = NS
- Mean number of weekly dressing changes was 1.1 in Group 1 vs 1.5 in Group 2, p = .0002

Power calculation:

- Was a power calculation for the primary endpoint reported, and if so, was it appropriate? Y, Y

Adjustment for multiplicity of statistical testing:

For Peer Review

- Was an adjustment reported if there were more than 1 secondary endpoints statistically tested? N

Blinding:
- Were patients blinded from treatment? N
- Were assessing investigators blinded from patient treatment? N

Allocation concealment:
- Was allocation concealment carried out? N

Lost to follow-up/discontinued/withdrawn:
- 35/109 (32.1%) total
    - 7/57 (12.3%) withdrawn from Group 1
    - 28/52 (53.8%) withdrawn from Group 2
    - Group difference: $p < .001$

87. Moody M. Comparison of Rosidal K and SurePress in the treatment of venous leg ulcers. *Br J Nurs.* 1999;8(6):345-355.

Study Groups: Group 1: short-stretch compression bandaging; Group 2: long-stretch compression bandaging

Statistical results:
- ITT analysis done for primary endpoint(s): Y
- **Primary endpoint:** Mean PAR, but there was no statistical analysis done (no p value)

Power calculation:
- Was a power calculation for the primary endpoint reported, and if so, was it appropriate? N

Adjustment for multiplicity of statistical testing:
- Was an adjustment reported if there were more than 1 secondary endpoints statistically tested? N

Blinding:
- Were patients blinded from treatment? N
- Were assessing investigators blinded from patient treatment? N

Allocation concealment:
- Was allocation concealment carried out? N

Lost to follow-up/discontinued/withdrawn:
- 15/48 (31.2%) total
    - 7/25 (28%) withdrawn from Group 1
    - 8/23 (33%) withdrawn from Group 2

88. Mostow EN, Haraway GD, Dalsing M, Hodde JP, King D. Effectiveness of an extracellular matrix graft (OASIS Wound Matrix) in the treatment of chronic leg ulcers: a randomized clinical trial. *J Vasc Surg.* 2005;41(5):837-843.

Study Groups: Group 1: Pig small intestine submucosa + SOC; Group 2: SOC

Statistical results:
- ITT analysis done for primary endpoint(s): Y
- **Primary endpoint:** Complete healing in 55% of Group 1 (34/62) vs 34% in Group 2

For Peer Review

(20/58) (p = .0196)
    - Group 1 was 2.3 times more likely to heal compared to Group 2
- At 12 weeks, 63% estimated to heal in Group 1 vs 40% in Group 2 (p = .0226)
- More healed in Group 1 even when taking into account vascular disease (p = .0253), diabetes (p = .0214), endocrine disease (p = .0272), and hypertension (p = .0204)
    - After adjusting for baseline ulcer size, Group 1 was 3 times more likely to heal than Group 2 (p = .0067; OR = 2.996)
    - More in Group 1 healed whether or not baseline debridement was done (p = .0215), but when done, then Group 1 was 4 times more likely to heal
        - 19 (63%) of 30 ulcers in Group 1 who had baseline debridement healed compared to 8 (30%) of 27 in Group 2 (p = .0167, OR: 4.10)
- 23 complications observed; 8 in Group 1 (12.9%) and 15 in Group 2 (25.9%) (p = .1920)
    - There was no significant difference in infection-related AEs among the groups (2 in Group 1 vs 7 in Group 2; p = .2611)
        - 1 (1.6%) had a study wound infection in Group 1 vs 5 (8.6%) in Group 2 (p = .1057)

Power calculation:
- Was a power calculation for the primary endpoint reported, and if so, was it appropriate? Y, N

Adjustment for multiplicity of statistical testing:
- Was an adjustment reported if there were more than 1 secondary endpoints statistically tested? N

Blinding:
- Were patients blinded from treatment? N
- Were assessing investigators blinded from patient treatment? N

Allocation concealment:
- Was allocation concealment carried out? Y

Lost to follow-up/discontinued/withdrawn:
- 24/120 (20%) total
    - 12/62 (19.4%) withdrawn from Group 1
    - 12/58 (20.7%) withdrawn from Group 2

89. Nelson EA, Iglesias CP, Cullum N, Torgerson DJ. Randomized clinical trial of four-layer and short-stretch compression bandages for venous leg ulcers (VenUS I). *Br J Surg.* 2004; 91(10):1292-9. **Data come from: Iglesias C, Nelson EA, Cullum NA, Torgerson DJ; on behalf of the Ven US team. VenUS I: a randomized controlled trial of two types of bandage for treating venous leg ulcers. *Health Technol Assess.* 2004;8(29)**.

Study Groups: Group 1: 4-layer bandaging (4LB); Group 2: short-stretch bandages

Statistical results:
- ITT analysis done for primary endpoint(s): Y
- **Primary endpoint:** Unadjusted median (95%) time to heal, days: 92 (71-113) in Group 1 vs 126 (95-157) in Group 2, log-rank = 2.46, p = .12
- Unadjusted proportion healed: 80.5% (n = 157) in Group 1 vs 76.5% (n = 147) in Group 2;

For Peer Review

p >.05, 95% CI: -4 to 15

- At 12 weeks, 46% healed in Group 1 vs 37% in Group 2, with an absolute difference of 9%, p >.05, 95%CI: -20 to 0
- At 24 weeks, 68% healed in Group 1 vs 55% in Group 2, with an absolute difference of 13%, p >.05, 95% CI: 2 to 22

- For adjusted Cox regression model analysis for treatment effect, the difference in the -2log*L* statistic was 2.43 (3186.98 in null model and 3184.54 in null model + arm),p = .12
    - According to the null model, the HR of healing associated with Group 1 vs Group 2 was 0.84, 95% CI: 0.67 to 1.05
- Using stepwise procedure, when treatment effect was included into the model, the -2log*L* statistic was reduced by 7.57 with p = .01
    - First stage: -2log*L,* difference, p-value
        - None: 3186.98
        - Duration months (Dur): 3127.87, 59.11, p = .00
        - Area (Are): 3150.04, 36.94, p = .00
        - Center (Cen): 3151.47, 35.50, p = .00
        - Ankle (Ank): 3162.78, 24.19, p = .00
        - Mobility (Mob): 3168.57, 18.41, p = .00
        - Episodes (Epi): 3171.32, 15.66, p = .00
        - Weight (Wei): 3181.70, 5.27, p = .02
        - Age: 3184.44, 2.53, p = .11
        - APBI: 3186.83, 0.15, p = .70
    - Second stage: -2log*L,* difference, p-value
        - Dur + Are + Cen + Ank + Mob + Epi: 3066.44
        - Are + Cen + Ank + Mob + Epi: 3093.98, 27.55, p = .00
        - Dur + Cen + Ank + Mob + Epi: 3077.72, 11.29, p = .00
        - Dur + Are + Ank + Mob + Epi: 3088.33, 21.90, p = .01
        - Dur + Are + Cen + Mob + Epi: 3071.62, 5.18, p = .02
        - Dur + Are + Cen + Ank + Epi: 3066.81, 0.38, p = .54
        - Dur + Are + Cen + Ank + Mob: 3974.23, 7.79, p = .01
    - Third stage: -2log*L,* difference, p-value
        - Dur + Are + Cen + Ank + Epi: 3066.81
        - Are + Cen + Ank + Epi: 3095.31, 28.50, p = .00
        - Dur + Cen + Ank + Epi: 3078.21, 11.40, p = .00
        - Dur + Are + Ank + Epi: 3089.41, 22.59, p = .00
        - Dur + Are + Cen + Epi: 3076.12, 9.30, p = .00
        - Dur + Are + Cen + Ank: 3074.71, 7.89, p = .01
    - Fourth stage: -2log*L,* difference, p-value
        - Dur + Are + Cen + Ank + Epi: 3066.81
        - Dur + Are + Cen + Ank + Epi + Age: 3066.81, 0, p = 1
        - Dur + Are + Cen + Ank + Epi + Wei: 3059.19, 7.62, p = .01
        - Dur + Are + Cen + Ank + Epi + ABPI: 3066.22, 0.59, p = .45
    - Final model, -2log*L*: Dur + Are + Cen + Ank + Epi + Wei: 3059.19
    - The estimated coefficient associated with the indicate variable for bandage

For Peer Review

treatment, arm (0.72), suggested that the hazard of healing for Group 2 was smaller relative to Group 1, o <.05, 95%CI for exponential of the coefficient associated with a covariate: 0.57 to 0.91
    - By including an interaction term between treatment and center, the -2log*L* stat was reduced by 19.0, p <.02, so the treatment effect varied by center
    - Using only the 4 original participating centers, there was still a significant treatment effect the 15.06 difference in the -2log*L* stat was significant, p = .00
        - When the interaction between treatment effect and center was tested, the 2.05 difference in the -2log*L* stat was not significant, p = .98, so there was no significant interaction between center and treatment
- No of recurring ulcers: Group 1: 27/157 vs Group 2: 29/147
    - No of censored cases: 130 in Group 1 vs 118 in Group 2
    - Log rank test analysis of time to recurrence: 1.51, p = .22
- Proportion of recurring ulcers in Group 1 was 36.2% vs 39.1% in Group 2, p >.05, 95%CI: 20 to 26
    - After 12 months, 13.1% reoccurred in Group 1 vs 25.4% in Group 2, p <.05, 95%CI: 2 to 23

Power calculation:
- Was a power calculation for the primary endpoint reported, and if so, was it appropriate? Y, Y

Adjustment for multiplicity of statistical testing:
- Was an adjustment reported if there were more than 1 secondary endpoints statistically tested? N

Blinding:
- Were patients blinded from treatment? N
- Were assessing investigators blinded from patient treatment? N

Allocation concealment:
- Was allocation concealment carried out? Y

Lost to follow-up/discontinued/withdrawn:
- 113/387 (29.2%) total
    - 47/195 (24.1%) withdrawn from Group 1
      66/192 (34.4%) withdrawn from Group 2

90. Nelzén O, Fransson I; Swedish SEPS Study Group. Early results from a randomized trial of saphenous surgery with or without subfascial endoscopic perforator surgery in patients with a venous ulcer. *Br J Surg.* 2011;98(4):495-500.

Study Groups: Group 1: great saphenous vein (GSV) surgery + subfascial endoscopic perforator surgery; Group 2: saphenous surgery alone

Statistical results:
- ITT analysis done for primary endpoint(s): Y
- **Primary endpoint:** Recurrence rate based on power calculation, but there were no recurrence rates reported
- GSV surgery prolonged by median of 15 min in Group 1 [median (range) duration of

operation 75 (25-245) min vs 60 (20 – 120) min in Group 2, p = .003

- No. minor complaints at 1 week follow-up: 23/37 in Group 1 vs 21/38 in Group 2, p = .534
    - No. with hematoma in the lower leg: 11 in Group 1 vs 3 in Group 2, p = .015
    - No. with thigh hematoma: 8 vs 18, p = .018
- No. of complications within 30 days: 12/37 in Group 1 vs 6/38 in Group 2, p = .101
    - Wound infection: 6 vs 3, p = .305
    - Delayed wound healing: 3 vs 1, p = .318
    - Pain: 6 vs 1, p = .051
    - Swelling: 1 vs 1, p = .983
- No. with minor residual symptoms: 10/37 in Group 1 vs 6/38 in Group 2, p = .507
- No. incompetent perforators (IPs) at baseline and 6-9 mos after surgery in 36 patients in Group 1 vs 37 in Group 2: 75 to 25 (67% reduction) in Group 1 vs 74 to 60 (19% reduction) in Group 2, p <.001
    - No of legs free from IPs after 6-9 months: 21 in Group1 vs 7 in Group 2, p <.001
    - No of legs free from original IPs after 6-9 months: 27 in Group vs 7 in Group 2 2, p <.001
    - Based on the original IPs, a significantly higher proportion had been occluded in Group 1 (65/75 vs 31/74 in the Group 2), p <.001
- Both groups were significantly improved in terms of ability to perform daily activities and symptoms from the operated legs, p <.001
    - There were no differences between groups in improvement at 3 and 12 months, p = NS

Power calculation:

- Was a power calculation for the primary endpoint reported, and if so, was it appropriate? Y, Y

Adjustment for multiplicity of statistical testing:

- Was an adjustment reported if there were more than 1 secondary endpoints statistically tested? N

Blinding:

- Were patients blinded from treatment? Y
- Were assessing investigators blinded from patient treatment? N

Allocation concealment:

- Was allocation concealment carried out? N

Lost to follow-up/discontinued/withdrawn:

- 1/75 (1.3%) total
    - 1/37 (2.7%) withdrawn from Group 1
    - 0/38 (0%) withdrawn from Group 2

91. Nikolovska S, Arsovski A, Damevska K, Gocev G, Pavlova L. Evaluation of two different intermittent pneumatic compression cycle settings in the healing of venous ulcers: a randomized trial. *Med Sci Monit.* 2005;11(7):Cr337-Cr343.

Study Groups: Group 1: Intermittent pneumatic compression (IPC) set at rapid inflation, short

For Peer Review

period of maintained compression; Group 2: IPC set at rapid deflation vs slow inflation

Statistical results:

- ITT analysis done for primary endpoint(s): Y
- **Primary endpoint:** No. of patients healed by 6 mo: 45/52 (86%) in Group 1 vs 32/52 (61%) in Group 2 (p = .004); absolute difference in healing rates was 25%
    - At 24 weeks the difference in ulcers healed was p = .003
- Median days to complete ulcer healing: 59 in Group 1 vs 100 in Group 2 (p = .001)
- Adjusting for ulcer size and duration, significant treatment effect in Group 1 (HR: 0.74, 95% CI: 0.56-0.90).
- Mean (SD) daily healing rate, cm^2 per day: 0.09 (0.09) in Group 1 vs 0.04 (0.03) in Group 2 (p = .0002)

Power calculation:

- Was a power calculation for the primary endpoint reported, and if so, was it appropriate? Y, Y

Adjustment for multiplicity of statistical testing:

- Was an adjustment reported if there were more than 1 secondary endpoints statistically tested? N

Blinding:

- Were patients blinded from treatment? N
- Were assessing investigators blinded from patient treatment? Y

Allocation concealment:

- Was allocation concealment carried out? N

Lost to follow-up/discontinued/withdrawn:

- 13/104 (12.5%)
    - 3/52 (5.8%) withdrawn from Group 1
    - 10/52 (19.2%) withdrawn from Group 2

92. O'Brien J, Edwards H, Stewart I, Gibbs H. A home-based progressive resistance exercise programme for patients with venous leg ulcers: a feasibility study. *Int Wound J.* 2013;10(4):389-396.

Study Groups: Group 1: home-based progression resistance exercise program; Group 2: usual care

Statistical results:

- ITT analysis done for primary endpoint(s): N
- **Primary endpoint:** Percentage of complete healed: 50 in Group 1 vs 40 in Group 2, chi square = 0.11, p = .74
- Percentage of reduction in ulcer area size: 77 in Group 1 vs 45 in Group 2, p = .34
- Repeated-measures ANOVA showed no significant difference between groups over time for the PUSH score at week 12, p = .35
    - Ejection fraction had and interaction effect between group and time, $F_{1,5}$ = 6.7, p = .05; and main effect for time, $F_{1,5}$ = 10.17, p = .02
        - Interaction effect between baseline and week 12: Group 1 had a significant increase, t (3) = -3.5, p = .03 vs Group 2 t (2) = -0.96, p = .44

For Peer Review

- Residual volume fraction had and interaction effect between group and time, $F_{1,5}$ = 7.02, p = .04; and main effect for time, $F_{1,5}$ = 10.54, p = .02
  - Interaction effect between baseline and week 12: Group 1 had a significant decrease, t (3) = -3.63, p = .03 vs Group 2 t (2) = -0.9, p = .46
- Venous filling index had no interaction effect between group and time, $F_{1,5}$ = 0.029, p = .61; and no main effect for group, $F_{1,5}$ = 0.004, p = .95, or time, $F_{1,5}$ = 2.57, p = .17
- Range of ankle motion had an interaction effect between group and time, $F_{1,5}$ = 7.02, p = .04; and main effect for time, $F_{2,7}$ = 11.49, p = .01; and main effect for time, $F_{2,7}$ = 11.49, p = .01
  - Interaction effect between base line and week 12: Group 1 had a significant increase, t (9) = -3.2, p = .01; but not observed at week 6, t(9) = -2.0, p = .07; or week 6 and week 12, t(9) = -1.6, p = .15

Power calculation:
- Was a power calculation for the primary endpoint reported, and if so, was it appropriate? Y, N

Adjustment for multiplicity of statistical testing:
- Was an adjustment reported if there were more than 1 secondary endpoints statistically tested? N

Blinding:
- Were patients blinded from treatment? N
- Were assessing investigators blinded from patient treatment? N

Allocation concealment:
- Was allocation concealment carried out? Y

Lost to follow-up/discontinued/withdrawn:
- 2/13 (15.4%) total
  - 2/7 (28.6%) withdrawn from Group 1
  - 0/6 (0%) withdrawn from Group 2

93. O'Brien JF, Grace PA, Perry IJ, Hannigan A, Clarke Moloney M, Burke PE. Randomized clinical trial and economic analysis of four-layer compression bandaging for venous ulcers. *Br J Surg.* 2003;90(7):794-798.

Study Groups: Group 1: 4-layer bandaging; Group 2: SOC

Statistical results:
- ITT analysis done for primary endpoint(s): Y
- **Primary endpoint:** proportion healed at 3 months: 54% in Group 1 vs 34% in Group 2, p <.001
  - Healing rates were still significantly different between groups after adjusting for age, baseline ulcer area and duration, and history of DVT, rheumatoid arthritis, and diabetes (p = .015)
- The rate of healing was throughout the 3 months was significantly better for Group 1 (p = .006)
- Mean reduction in ulcer size was not significantly different between groups: -1.1 (95%

For Peer Review

CI: -2.9 to 0.7) cm^2, p = NS
- Median (IQR) cost per leg healed, Euros: 209.7 (137.5-269.4) vs 234.6 (168.2-345.1), p = .04

Power calculation:
- Was a power calculation for the primary endpoint reported, and if so, was it appropriate? Y, Y

Adjustment for multiplicity of statistical testing:
- Was an adjustment reported if there were more than 1 secondary endpoints statistically tested? N

Blinding:
- Were patients blinded from treatment? N
- Were assessing investigators blinded from patient treatment? N

Allocation concealment:
- Was allocation concealment carried out? N

Lost to follow-up/discontinued/withdrawn:
- 3/200 (1.5%) total
    - 3/100 (3.0%) withdrawn from Group 1
    - 0/100 (0.0%) withdrawn from Group 2

94. Ogrin R, Darzins P, Khalil Z. The use of sensory nerve stimulation and compression bandaging to improve sensory nerve function and healing of chronic venous leg ulcers. *Curr Aging Sci.* 2009;2(1):72-80.

Study Groups: Group 1: low-frequency sensory nerve stimulation (LF-SNS); Group 2: placebo, LF-SNS machine without electrical stimulation

Statistical results:
- ITT analysis done for primary endpoint(s): N
- **Primary endpoints:** complete wound healing and epithelialization rate, but no statistical analysis was provided
- No significant differences in age between Groups, $F_{1,27} = 0.255$, p = .62, but those that healed in both Groups were significantly younger than those did not heal, $F_{1,27} = 4.59$, $p < .05$, r = 0.54
- There was no differences in ages in the Group 1 in participants that healed, $F_{1,17} = 0.02$, p = .88
- Duration of ulceration in participants who healed was less than those who did not heal by 12 weeks, , $F_{1,28} = 0.33$, p = .6
- In ulcers that healed in 12 weeks, there was no difference in ages in Group 1 (73.4 years, SD = 3.5) vs Group 2 (72.7 years, SD = 3.2), but 3 clinical aspects noted:
    - Duration of ulceration in healed participants, months (SD): 44.38 (13.9) in Group 1 vs 38.07 (15.19) in Group 2 ,$F_{1,217} = 2.97$, p = .10
    - Mean (SD) of ulcer size in time 0 of trial in patients that healed, cm^2: 7.5 (8.5) in Group 1 vs 3.2 (2.9) in Group 2, $F_{1,17} = 2.27$, p = .15
    - Healing rates (SD) cm^2/week: 1.1 (0.3) in Group 1 vs 0.6 (0.2) in Group 2, $F_{1,17} = 2.0$, p = .18

For Peer Review

- Microvascular blood flow,cm^2 (SD); $TcpO_2$ at 44°C,mmHg (SD); $TcpO_2$ at 39°C,mmHg (SD); 5Hz ECPT, mA (SD); Flare response to capsaicin, cm^2 (SD)
    - Total Group 1 (n =15):
        - Stimulated leg and Control leg: 25.8 (23.9) and 0.4 (5.3), $t_{1,6}$ = 1.1, p = .29; 7.5 (3.7) and -0.9 (4.8), $t_{1,6}$ = 1.33, p = .21; 5.1 (2.0) and -2.6 (4.9), $t_{1,6}$ = 1.65, p = .15; -0.3 (0.5) and -0.2 (0.3) $t_{1,6}$ = 1.18, p = .86; 9.1 (4.1) and 5.6 (1.9),$t_{1,6}$ = 1.19, p = .32
    - Participants that healed in Group 1 (n= 8):
        - Stimulated leg and Control leg: -1.9 (33.9) and -2.3 (5.5), $t_{1,7}$ = 0.01, p = .99; 5.9 (6.1) and 6.6 (6.2), $t_{1,7}$ = 0.81, p = .94; 4.8 (1.7) and 4.8 (5.9), $t_{1,3}$ = 0.00, p = 1.0; -0.9 (0.6) and -0.2 (0.2) $t_{1,7}$ = 1.48, p = .18; 11.8 (8.1) and 6.0 (3.4),$t_{1,1}$ = 2.55, p = .24
    - In Total Group 2 (n =15):
        - Stimulated leg and Control leg: 16.9 (42.1) and -15.4 (60.1), $t_{1,14}$ = 0.75, p = .47; 13.9 (5.7) and 18.7 (7.3), $t_{1,14}$ = 2.34, p = <.05; 5.5 (3.6) and -4.4 (9.1), $t_{1,7}$ = 1.09, p = .31; -0.4 (0.3) and -0.4 (0.4) $t_{1,14}$ = 0.3, p = .77; 1.7 (2.0) and 2.4 (5.0),$t_{1,4}$ = 0.84, p = .45
    - In Group 2 including only participant that healed (n= 10):
        - Stimulated leg and Control leg: 39.8 (60.1) and -23.6 (31.1), $t_{1,9}$ = 1.04, p = .33; 18.7 (7.3) and -0.1 (5.4), $t_{1,9}$ = 2.29, p = .05; 3.3 (4.23) and -5.33 (5.86), $t_{1,5}$ = 1.10, p = 0.32; -0.4 (0.4) and -0.7 (0.5) $t_{1,9}$ = 0.81, p = .44; 2.4 (5.0) and 2.4 (1.8),$t_{1,3}$= 0.02, p = .99
    - Mean (SD) difference from baseline between stimulated and control leg in 5 Hz ECPT, mA: - 0.30 (-0.72 to no value) and - 0.20 (from -0.50 to no value), p = NS, more negative result, more improvement in sensation occurred.
    - Mean (SD) difference from baseline between stimulated and control leg in $TcpO_2$ at 44°C, mmHg: 7.3 (from 11.0 to no value) and -0.70 (from -0.60 to no value), p = NS
    - Mean (SD) difference from baseline between stimulated and control leg in Capsaicin, cm^2: 9.0 (from no value to 13.0) and 5.0 (from no value to 7.5), p = NS
    - Mean (SD) difference from baseline between healed and unhealed limbs in actively stimulated limb in 5 Hz ECPT, mA: - 0.9 (from -1.3 to no value) and 0.55 (from no value to 1.3), p = NS, more negative result, more improvement in sensation occurred.
    - Mean (SD) difference from baseline between healed and unhealed limbs in actively stimulated limb in Capsaicin, cm^2: 11.7 (from no value to 16.5) and 4.2 (from no value to 11.5), p = NS

Power calculation:

- Was a power calculation for the primary endpoint reported, and if so, was it appropriate? N

Adjustment for multiplicity of statistical testing:

- Was an adjustment reported if there were more than 1 secondary endpoints statistically tested? N

Blinding:

For Peer Review

- Were patients blinded from treatment? Y
- Were assessing investigators blinded from patient treatment? Y

Allocation concealment:

- Was allocation concealment carried out? N

Lost to follow-up/discontinued/withdrawn:

- 3/29 (10.3%) total (no per-group data provided)

95. O'Hare JL, Earnshaw JJ. Randomised clinical trial of foam sclerotherapy for patients with a venous leg ulcer. *Eur J Vasc Endovasc Surg.* 2010;39(4):495-499.

Study Groups: Group 1: foam sclerotherapy + 4-layer compression bandages; Group 2: 4-layer compression bandages (control)

Statistical results:

- ITT analysis done for primary endpoint(s): N
- **Primary endpoint:** At 24 weeks, 12/13 (92%) healed in Group vs 17/20 (85%) in Group 2, with no significant difference in time to healing, p = .72
    - Median healing times 95% CI: 0.17-1.12

Power calculation:

- Was a power calculation for the primary endpoint reported, and if so, was it appropriate? Y, Y

Adjustment for multiplicity of statistical testing:

- Was an adjustment reported if there were more than 1 secondary endpoints statistically tested? N

Blinding:

- Were patients blinded from treatment? N
- Were assessing investigators blinded from patient treatment? N

Allocation concealment:

- Was allocation concealment carried out? N

Lost to follow-up/discontinued/withdrawn:

- 7/40 (17.5%) total
    - 2/22 (9.1%) withdrawn from Group 1
    - 5/18 (27.8%) withdrawn from Group 2

96. Oliveira MGd, Abbade LPF, Miot HA, Ferreira RR, Deffune E. Pilot study of homologous platelet gel in venous ulcers. *An Bras Dermatol.* 2017; 92(4):499-504.

Study Groups: Group 1: Platelet Gel (n = 9); Group 2: Hydrocolloid dressing (n = 12)

Statistical results:

- ITT analysis done for primary endpoint(s): Y
- **No primary endpoints are identified.**
- Both groups had a significant mean PAR (69%) in 90 days, p <.01
    - Significant difference between groups favoring Group 2 for progressive reduction of ulcer areas (70% vs 64%, p <.01)
    - When adjusted for age, varicose veins, and recurrence, variables in which the groups differed at p <.20, statistical difference was maintained (p <.01) favoring

For Peer Review

Group 2

- Biopsies of 3 VLUs in Group 1 and 7 in Group 2 were analyzed for neovascularization between days 0 and 90 and no statistical difference was found (p = .67)

Power calculation:

- Was a power calculation for the primary endpoint reported, and if so, was it appropriate? N

Adjustment for multiplicity of statistical testing:

- Was an adjustment reported if there were more than 1 secondary endpoints statistically tested? N

Blinding:

- Were patients blinded from treatment? N
- Were assessing investigators blinded from patient treatment? N

Allocation concealment:

- Was allocation concealment carried out? N

Lost to follow-up/discontinued/withdrawn: None reported

97. Olyaie M, Samiee Rad F, Elahifar MA, et al. High-frequency and noncontact low-frequency ultrasound therapy for venous leg ulcer treatment: A randomized, controlled study. *Ostomy Wound Manage.* 2013;59(8):14-20.

Study Groups: Group 1: high-frequency ultrasound (HFU), n = 30; Group 2: noncontact, low-frequency ultrasound (NCLFU), n = 30; Group 3: SOC, n =30

Statistical results:

- ITT analysis done for primary endpoint(s): Y.
- **Primary endpoint:** There are no primary endpoints identified.
- Means (SD) of ulcer size
    - at baseline, cm^2: 9.86 (3.95) in Group 1 vs 10.01 (4.58) in Group 2 vs 9.60 (5.54) in Group 3, n = 30 each group, p = .94
    - at 2 months, cm^2: 4.50 (3.49) in Group 1 vs 6.28 (3.28) in Group 2 vs 6.61 (4.18) in Group 3, n = 30 each group, p = .06
    - at 4 months, cm^2: 3.23 (2.39) in Group 1 vs 2.72 (2.16) in Group 2 vs 4.28 (2.80) in Group 3, n = 30 each group, p = .0, p =.0
- Mean (SD) time to heal, months: 6.86 (2.04) in Group 1 vs 6.65 in Group 2 vs 8.50 (2.17) in Group 3, p= .001
- Number (%) of changes in edema (staging: 1+,2+, 3+, 4+)
    - at baseline: 12 (40), 7 (23.3), 8 (26.6), 3 (10) in Group 1 vs 9 (30), 4 (13.3), 9 (30), 8 (26.6) in Group 2 vs 10 (33.3), 5 (16.6), 8 (26.6), 7 (23.3) in Group 3, p = .28
    - at 2 months: 14 (46.6), 9 (30), 4 (13.3), 3 (10) in Group 1 vs 13 (43.3), 7 (23.3), 4 (13.3), 6 (20) in Group 2 vs 11 (36.6), 8 (26.6), 5 (16.6), 6 (20) in Group 3, p = .54
    - at 4 months: 24 (80), 5 (16.6), 0 (0), 1 (3.3) in Group 1 vs 22 (73.3), 2 (6.6), 2 (6.6), 4 (13.3) in Group 2 vs 8 (26.6), 11 (36.6), 6 (20), 5 (16.6) in Group 3, p = .00, p =.00
- Means (SD) of change in pain score
    - at baseline, cm^2: 6.0 (1.59) in Group 1 vs 6.16 (1.51) in Group 2 vs 6.20 (1.64) in

For Peer Review

Group 3, n = 30 each group, p = .58
  - at 2 months, cm^2: 5.40 (2.07) in Group 1 vs 5.70 (1.95) in Group 2 vs 5.86 (1.43) in Group 3, n = 30 each group, p = .15
  - at 4 months, cm^2: 3.96 (2.88) in Group 1 vs 3.26 (3.06) in Group 2 vs 5.10 (1.88) in Group 3, n = 30 each group, p = .02

Power calculation:

- Was a power calculation for the primary endpoint reported, and if so, was it appropriate? N

Adjustment for multiplicity of statistical testing:

- Was an adjustment reported if there were more than 1 secondary endpoints statistically tested? N

Blinding:

- Were patients blinded from treatment? N
- Were assessing investigators blinded from patient treatment? N

Allocation concealment:

- Was allocation concealment carried out? N

Lost to follow-up/discontinued/withdrawn: None reported.

98. Partsch H, Damstra RJ, Tazelaar DJ, et al. Multicentre, randomised controlled trial of four-layer bandaging versus short-stretch bandaging in the treatment of venous leg ulcers. *Vasa.* 2001;30(2):108-113.

Study Groups: Group 1: 4-layer bandaging; Group 2: short-stretch bandaging

Statistical results:

- ITT analysis done for primary endpoint(s): N
- **Primary endpoint:** complete healing and time to complete healing, n = 112 patients: 53 in Group 1 vs 59 in Group 2
  - The proportional hazard model for treatment and center gave strong evidence of different levels of healing between centers, p = .003
    - Number (%) of patients healed by center:
      - Vienna: 4/5 (80) in Group 1 vs 6/9 (67) in Group 2,
      - Graz: 4/9 (44) in Group 1 vs 3/10 (30) in Group 2,
      - Drachten: 12/15 (80) in Group 1 vs 13/15 (87) in Group 2,
      - Sneek: 4/6 (67) in Group 1 vs 7/7 (100) in Group 2,
      - Heerenveen: 9/10 (90) in Group 1 vs 9/10 (90) in Group 2,
      - Nijmegen: 0/6 (0) in Group 1 vs 4/6 (67) in Group 2,
      - Leiden: 0/2 (0) in Group 1 vs 1/2 (50) in Group 2,
  - Proportional hazard models were re-fitted including terms for initial ulcer area and duration of ulceration there was no evidence of center effect, p = .79
  - There was no significant difference between treatments in the primary survival analysis, p = .49, with a HR of 1.19 and 95% CI (0.73 to 1.91), representing a higher healing rate in Group 2 vs Group 1
  - A secondary survival analysis with no adjustment for covariates gave a similar result with no significant difference between Groups , p = .54

For Peer Review

Power calculation:

- Was a power calculation for the primary endpoint reported, and if so, was it appropriate? Y, Y

Adjustment for multiplicity of statistical testing:

- Was an adjustment reported if there were more than 1 secondary endpoints statistically tested? N

Blinding:

- Were patients blinded from treatment? N
- Were assessing investigators blinded from patient treatment? N

Allocation concealment:

- Was allocation concealment carried out? N

Lost to follow-up/discontinued/withdrawn:

- 26/116 (22.4%) total
    - 4/116 (3.4%) excluded from analysis
    - 12/53 (22.6%) withdrawn from Group 1
    - 10/59 (16.9%) withdrawn from Group 2
    - Difference in group withdrawal rates: p = .45

99. Planinsek Rucigaj T. Effectiveness of non-alcohol film forming skin protector on the skins isles inside the ulcers and the healing rate of venous leg ulcers. Randomized clinical study. *EWMA J.* 2007;7(1):23-25.

Study Groups: Group 1: non-alcohol film forming skin protector + hydrocolloid dressings (n =14); Group 2: hydrocolloid dressings alone (control) (n = 13)

Statistical results:

- ITT analysis done for primary endpoint(s): Y
- **Primary endpoint:** There are no primary endpoints identified and there was no statistical analysis done of any data.

Power calculation:

- Was a power calculation for the primary endpoint reported, and if so, was it appropriate? N

Adjustment for multiplicity of statistical testing:

- Was an adjustment reported if there were more than 1 secondary endpoints statistically tested? N

Blinding:

- Were patients blinded from treatment? N
- Were assessing investigators blinded from patient treatment? N

Allocation concealment:

- Was allocation concealment carried out? N

Lost to follow-up/discontinued/withdrawn: None reported.

100. Polignano R, Bonadeo P, Gasbarro S, Allegra C. A randomised controlled study of four-layer compression versus Unna's Boot for venous ulcers. *J Wound Care.* 2004;13(1):21-24.

For Peer Review

Study Groups: Group 1: 4-layer bandage system; Group 2: Unna's boot

Statistical results:

- ITT analysis done for primary endpoint(s): Y
- **There were no primary endpoints identified.**
- No. (%) healed by 24 weeks: 29/39 (74%) in Group 1 vs 66% (19/29) in Group 2, p = .42; estimate of difference: 0.09; 95% CI: -0.13 to 0.31
- Median (range) time to heal, days: 51 (7-175) in Group 1 vs 49 (7-168) in Group 2, p = .13, HR: 1.62 (95% CI: 0.87-3.02)
  - Larger ulcers took longer to heal, p = .01
  - Ulcer duration no effect on time to heal, p = .12
- Mean (SD, median, range) PAR: 79.1 (65.7, 100.0, -283.3 to 100.0) in Group 1 vs 24.6 (165.5, 100.0, -489.3, 100.0) in Group 2, p = .30
- There was no group difference in in change in pain score from initial to final assessment, p = .32
  - % (n) with no change in pain: 35% (12/34) in Group 1 and 13% (3/24) in Group 2
  - % (n) with reduced pain: 62% (21/34) vs 79% (19/24)
  - % (n) with increased pain: 3% (1/34) vs 8% (2/24)
- There was a significant group difference with respect to smoothness at final application, p = .013
  - % (n) rated as excellent: 34% (11/32) in Group 1 vs 5% (1/21) in Group 2
  - % (n) rated as good: 66% (21/32) vs 95% (20/21)
- There was a significant group difference in bandage appearance at initial application, p = .04
  - % (n) with excellent appearance: 30% (11/37) in Group 1 vs 11% (3/28) in Group 2
  - % (n) with good appearance: 70% (26/37) vs 86% (24/28)
  - % (n) with average appearance: 0% (0) vs 4% (1/28)
- There was no group difference in bandage appearance at final application, p = .18
  - % (n) with excellent appearance: 30% (9/30) in Group 1 vs 20% (2/10) in Group 2
- There was no group difference in comfort at the initial assessment, p = .93
  - % (n) with excellent comfort: 10% (4/39) in Group 1 vs 7% (2/28) in Group 2
- There was no group difference in comfort at the final assessment, p = .74
  - % (n) with excellent comfort: 26% (9/35) in Group 1 vs 4% (1/23) in Group 2

Power calculation:

- Was a power calculation for the primary endpoint reported, and if so, was it appropriate? N

Adjustment for multiplicity of statistical testing:

- Was an adjustment reported if there were more than 1 secondary endpoints statistically tested? N

Blinding:

- Were patients blinded from treatment? N
- Were assessing investigators blinded from patient treatment? N

Allocation concealment:

For Peer Review

- Was allocation concealment carried out? N

Lost to follow-up/discontinued/withdrawn:

- 8/68 (11.8%) total
  - 4/39 (10.2%) withdrawn from Group 1
  - 4/29 (13.8%) withdrawn from Group 2

101. Rivera-Arce E, Chavez-Soto MA, Herrera-Arellano A, et al. Therapeutic effectiveness of a Mimosa tenuiflora cortex extract in venous leg ulceration treatment. *J Ethnopharmacol.* 2007;109(3):523-8.

Study Groups: Group 1: phytodrug developed from *Mimosa tenuiflora* bark; Group 2: tinted hydrogel placebo

Statistical results:

- ITT analysis done for primary endpoint(s): N
- **There were no primary endpoints identified.**
- Absolute frequencies, relative frequencies (%) of ulcers ≥80% healed
  - Week 1: 0/20, 0% in Group 1 vs 0/20, 0%, p = NS
  - Week 2: 1/20, 5.0% vs 0/19, 0%, p = .32
  - Week 3: 5/19, 26.3% vs 0/19, 0%, p = .01
  - Week 4: 11/19, 57.9% vs 0/19, 0%, p = .0001
  - Week 5: 14/19, 73.7% vs 0/19, 0%, p = .0001
  - Week 6: 17/19, 89.5% vs 1/18, 5.6%, p = .0001
  - Week 7: 18/19, 94.7% vs 1/18, 5.6%, p = 0001
  - Week 8: 19/19, 100.0% vs 1/17, 5.9%, p = .0001
  - Week 9: 19/19, 100.0% vs 1/15, 6.7%, p =.0001
  - Week 10: 19/19, 100.0% vs 1/15, 6.7%, p = .0001
  - Week 11: 19/19, 100.0% vs 2/11, 18.2%, p = .0001
  - Week 12: 19/19, 100.0% vs 2/11, 18.2%, p = .0001
- There were no differences in the groups' therapeutic safety, hepatic, and renal parameters, p = NS

Power calculation:

- Was a power calculation for the primary endpoint reported, and if so, was it appropriate? N

Adjustment for multiplicity of statistical testing:

- Was an adjustment reported if there were more than 1 secondary endpoints statistically tested? N

Blinding:

- Were patients blinded from treatment? Y
- Were assessing investigators blinded from patient treatment? Y

Allocation concealment:

- Was allocation concealment carried out? N

Lost to follow-up/discontinued/withdrawn:

- 10/40 (25.0%) total
  - 1/20 (5.0%) withdrawn from Group 1

For Peer Review

  - 9/20 (45.0%) withdrawn from Group 2
  - Difference between groups: p = .003

102. Robson MC, Hanfnt J, Garner WG. Healing of chronic venous ulcers is not enhanced by the addition of topical repifermin (KGF-2) to standardized care. *J Applied Res.* 2004;4(2):302-311.

Study Groups: Group 1: Repifermin 60 ug/cm^2; Group 2: Repifermin 120 ug/cm^2; Group 3: placebo

Statistical results:

- ITT analysis done for primary endpoint(s): Y
- **Primary endpoint:** Complete closure by 20 weeks: 58.5% in Group 1 (72/123); 51.8% in Group 2 (58/112); 61.5% in Group 3 (72/117); p = .6351 and .1362 comparing Groups 1 and 2 to Group 3
- Complete closure by 26 weeks: 65.0% in Group 1 (80/123); 58.0% in Group 2 (65/112); 66.7% in Group 3 (78/117); p = .7906 and .1774 comparing Groups 1 and 2 to Group 3
- Complete closure by 16 weeks: 49.6% in Group 1 (61/123); 48.2% in Group 2 (54/112); 53.0% in Group 3 (62/117); p = .5986 and .4697 comparing Groups 1 and 2 to Group 3
- Time to heal difference between Group 1 and 3: p = .9849; between Group 2 and 3: p = .3932
- Mean PAR not statistically different for 3 groups (p > .05)
- At least one AE: Group 1: 67.5% (83/123); Group 2: 70.5% (79/112); Group 3: 76.1% (89/117), p =.3270
- At least one related AE: Group 1: 10.6% (13/123); Group 2: 8.9% (10/112); Group 3: 7.7% (9/117), p =.7390
- At least one serious AE: Group 1: 10.6% (13/123); Group 2: 8.9% (10/112); Group 3: 16.2% (19/117), p =.2070
- At least one severe AE: Group 1: 7.3% (9/123); Group 2: 10.7% (12/112); Group 3: 10.3% (12/117), p =.6097

Power calculation:

- Was a power calculation for the primary endpoint reported, and if so, was it appropriate? Y, Y

Adjustment for multiplicity of statistical testing:

- Was an adjustment reported if there were more than 1 secondary endpoints statistically tested? N

Blinding:

- Were patients blinded from treatment? Y
- Were assessing investigators blinded from patient treatment? Y

Allocation concealment:

- Was allocation concealment carried out? N

Lost to follow-up/discontinued/withdrawn:

- 53/352 (15%) total
  - 18/123 (14.6%) withdrawn from Group 1
  - 20/112 (16.7%) withdrawn from Group 2

For Peer Review

- 15/117 (12.8%) withdrawn from Group 3

103. Robson MC, Phillips TJ, Falanga V, et al. Randomized trial of topically applied repifermin (recombinant human keratinocyte growth factor-2) to accelerate wound healing in venous ulcers. *Wound Repair Regen.* 2001;9(5):347-352.

Study Groups: Group 1: repifermin 20 ug/cm^2; Group 2: repifermin 60 ug/cm^2; Group 3: placebo

Statistical results:

- ITT analysis done for primary endpoint(s): N
- **Primary endpoint:** complete wound healing (n = 31 in Group 1 vs 32 in Group 2 vs 31 in Group 3)
  - 100% healed, %: 32 in group 1 vs 38 in Group 2 vs 29 in Group 3, p = .57
  - 90% healed, %: 55 in group 1 vs 53 in Group 2 vs 36 in Group 3, p = .093
  - 75% healed, %: 71 in group 1 vs 63 in Group 2 vs 45 in Group 3, p = .047
  - 50% healed, %: 81 in group 1 vs 75 in Group 2 vs 61 in Group 3, p = .095
  - There were differences statistically significant for both 90% combined healed group, p = .028 and 75% combined healed group end points, p = .007
- The percentage decrease from baseline in area: Group 1 (75%) and Group 2 (73%) vs Group 3 (56%), p = 0.59
- Percentage of subjects with wounds ≤15 cm^2 in area and wound age ≤18 months were 81 in Group 1 and 89 in Group 2 vs 63% in Group 3, p = .029
  - Percentage of subjects with wounds ≤15 cm^2 in area and wound age ≤18 months that were 50% healed were 81 in Group 1 and 82 in Group 2 vs 69% in Group 3, p = NS
  - Percentage of subjects with wounds ≤15 cm^2 in area and wound age ≤18 months that were 75% healed were 81 in Group 1 and 82 in Group 2 vs 50% in Group 3, p = .007
  - Percentage of subjects with wounds ≤15 cm^2 in area and wound age ≤18 months that were 90% healed were 58 in Group 1 and 60 in Group 2 vs 45% in Group 3, p = .028
  - Percentage of subjects with wounds ≤15 cm^2 in area and wound age ≤18 months that were 100% were 40 in Group 1 and 82 in Group 2 vs 40% in Group 3, p = NS
- There were no clinically or statistically significant differences between the 3 Groups for any of the safety laboratory test para meters evaluated, p = NS
- Number (%) of treatments-emergent adverse events occurring in 10% or more subjects (n = 31 in Group 1 vs 32 in Group 2 vs 31 in Group 3):
  - Leg pain: 5 (16) in Group 1 vs 9 (28) in Group 2 vs 8 (26) in Group 3, p = .70
  - Pruritus: 4 (13) in Group 1 vs 8 (25) in Group 2 vs 2 (6) in Group 3, p = .09
  - Skin ulcer: 6 (19) in Group 1 vs 7 (22) in Group 2 vs 7 (23) in Group 3, p = .83
  - Rash: 4 (13) in Group 1 vs 8 (25) in Group 2 vs 4 (13) in Group 3, p = .45
  - Abrasion: 3 (10) in Group 1 vs 4 (13) in Group 2 vs 1 (3) in Group 3, p = .16
  - Venous ulcer reopen: 1 (3) in Group 1 vs 5 (16) in Group 2 vs 4 (13) in Group 3, p = .62

For Peer Review

- Investigator suspected study ulcer infection: 2 (6) in Group 1 vs 4 (13) in Group 2 vs 8 (26) in Group 3, p = .04

Power calculation:

- Was a power calculation for the primary endpoint reported, and if so, was it appropriate? Y, Y

Adjustment for multiplicity of statistical testing:

- Was an adjustment reported if there were more than 1 secondary endpoints statistically tested? N

Blinding:

- Were patients blinded from treatment? Y
- Were assessing investigators blinded from patient treatment? Y

Allocation concealment:

- Was allocation concealment carried out? N

Lost to follow-up/discontinued/withdrawn:

- 5/94 (5.3%) total
  - 3/31 (9.7%) withdrawn from Group 1
  - 1/32 (3.3%) withdrawn from Group 2
  - 1/31 (3.2%) withdrawn from Group 3

104. Rodrigues AL, de Oliveira BG, Futuro DO, Secoli SR. Effectiveness of papain gel in venous ulcer treatment: randomized clinical trial. *Rev Lat Am Enfermagem.* 2015;23(3):458-465.

Study Groups: Group 1: 2% papain gel; Group 2: 2% carboxymethyl cellulose (control)

Statistical results:

- ITT analysis done for primary endpoint(s): N
- **Primary endpoint:** Reduction in area for Group 1 significantly greater between treatment weeks (p = .006); not significant for Group 2 (p =.408)
  - Using Dunn's test and correction of the adjusted p-value, the main variation in area was found for Group 1 to be between the 5th and 12 weeks (adjusted p = .032)
- Slough tissue showed a significant reduction by 12 weeks in Group 1 (p =.001) and in Group 2 (p = .004)
- Granulation tissue showed a significant increase by 12 weeks in Group 1 (p = .021) and in Group 2 (p =.031)
- Epithelialized tissue showed a significant increase in Group 1 (p = .004) vs Group 2 (p = .063)
- Amount of exudates in Group 1: Most ulcers (68.8%) had a small amount at first visit, while at the last visit, 37.5% had a small amount, 37.5% had a moderate amount, 12.5% had a large amount, and 12.5% had none (p = .727)
- Amount of exudates in Group 2: 50% had a small amount, 25% moderate, and 25% large at first visit, while at the last visit, 58.5% had a small amount (p = .750)

Power calculation:

- Was a power calculation for the primary endpoint reported, and if so, was it appropriate? N.

For Peer Review

Adjustment for multiplicity of statistical testing:
- Was an adjustment reported if there were more than 1 secondary endpoints statistically tested? N

Blinding:
- Were patients blinded from treatment? N
- Were assessing investigators blinded from patient treatment? N

Allocation concealment:
- Was allocation concealment carried out? N

Lost to follow-up/discontinued/withdrawn:
- 3/21 (14.3%) total
  - 1/11 (9.1%) withdrawn from Group 1
  - 2/10 (20%) withdrawn from Group 2

For Peer Review

105. Romanelli M, Macchia M, Panduri S, Paggi B, Saponati G, Dini V. Clinical evaluation of the efficacy and safety of a medical device in various forms containing Triticum vulgare for the treatment of venous leg ulcers - a randomized pilot study. *Drug Des Devel Ther.* 2015;9:2787-2792.

Study Groups: *Triticum vulgare* applied using 5 vehicles: Group 1: cream, n = 10; Group 2: soaked gauzes, n =10, Group 3: foam, n = 10, Group 4: hydrogel, n = 10; Group 5= dressing gel, n = 9

Statistical results:
- ITT analysis done for primary endpoint(s): Y
- **Primary endpoint:** Mean (95% CI) of ulcer surface area, cm^2: differences vs baseline value through the study (Visit 2; Visit 3; visit 4; Visit 5):
  - Group 1: -2.45 (-5.0 to 0.1); -3.07 (-5.9 to -0.3); -6.03 (-10.3 to -1.8); -11.02 (-18.06 to -3.4), Wilcoxon p = .0059, Student´s t p = .0095
  - Group 2: -3.78 (-9.8 to 2.2); -5.64 (-12.6 to 1.3); -5.40 (-12.7 to 1.9); -5.59 (-12.8 to 1.7), Wilcoxon p = .16, Student´s t p = .11
  - Group 3: -0.77 (-1.6 to 0.1); -0.67 (-3.1 to 1.8); -2.25 (-5.7 to 1.2); -4.9 (-7.4 to -0.8), Wilcoxon p = .0039, Student´s t p = .021
  - Group 4: -1.72 (-3.1 to -0.4); -4.50 (-7.5 to -1.5); -6.51 (-10.0 to -3.0); -8.12 (-12.2 to -4.1), Wilcoxon p = .0020, Student´s t p = .0014
  - Group 5: -3.74 (-7.2 to -0.3); -3.65 (-6.8 to -0.5); -6.10 (-10.8 to -1.4); -6.31 (-10.2 to -2.4), Wilcoxon p = .0039, Student´s t p = .0018
- Total symptoms score decrease in all groups: -60 in Group 1 vs -90 in Group 2 vs -39 in Group 3 vs -55 in group 4 vs -18 in Group 5. The greatest efficacy on signs/symptoms was observed in patients treated with gauzes, p values: Student´s t = 0.031; Wilcoxon = 0.023

Power calculation:
- Was a power calculation for the primary endpoint reported, and if so, was it appropriate? N.

Adjustment for multiplicity of statistical testing:
- Was an adjustment reported if there were more than 1 secondary endpoints statistically

tested? N

Blinding:

- Were patients blinded from treatment? N
- Were assessing investigators blinded from patient treatment? N

Allocation concealment:

- Was allocation concealment carried out? N

Lost to follow-up/discontinued/withdrawn: None reported.

106. Romanelli M, Mulder G, Paggi B, Macchia M, Panduri S, Dini V. The use of a collagen matrix in hard-to-heal venous leg ulcers. *J Wound Care.* 2015;24(11):543-4, 6-7.

Study Groups: Group 1: collagen + alginate dressing; Group 2: alginate dressing alone (control), 40 total, but unknown how many randomized to each group

Statistical results:

- ITT analysis done for primary endpoint(s): Y
- **Primary endpoint:** there were no primary endpoints identified.
- Granulation tissue formation at 12 weeks: 65% in Group 1 vs 38% in Group 2, $p < .001$
- Mean PAR at 12 weeks: 45% in Group 1 vs 20% in Group 2, $p < .001$
- There was no statistical difference in time to heal among groups, p = NS

Power calculation:

- Was a power calculation for the primary endpoint reported, and if so, was it appropriate? N

Adjustment for multiplicity of statistical testing:

- Was an adjustment reported if there were more than 1 secondary endpoints statistically tested? N

Blinding:

- Were patients blinded from treatment? N
- Were assessing investigators blinded from patient treatment? N

Allocation concealment:

- Was allocation concealment carried out? N

Lost to follow-up/discontinued/withdrawn: None reported

107. Rowland J. Intermittent pump versus compression bandages in the treatment of venous leg ulcers. *Aus N Z J Surg.* 2000;70(2):110-113.

Study Groups: Group 1: intermittent pneumatic compression pump; Group 2: high-stretch bandaging, crossed over to other treatment after initial treatment period of 2 or 3 months

Statistical results:

- ITT analysis done for primary endpoint(s): Y
- **Primary endpoint:** There were no primary endpoints identified.
- Ulcer size (cm^2?): ITT: 275 at 1 month in Group 1 vs 215 in Group 2 to 190 at 3 months vs 137, Rao R (2, 29) = 5.17, $p < .012$
  - PP: Rao R(2, 19) = 3.95, $p < .0369$
- Lower limb volume (cm^3?) was shown to decrease significantly with time in both ITT and PP from 2,800 cm at 1 month in Group 1 to 2,500 at 3 months vs 2,400 to 1,400 in

Group 2, p = .00001
  - Multivariate model showed a significant difference between Groups 1 and 2 on the censored data (p = .0346) which was not seen in the ITT analysis

Power calculation:
- Was a power calculation for the primary endpoint reported, and if so, was it appropriate? N

Adjustment for multiplicity of statistical testing:
- Was an adjustment reported if there were more than 1 secondary endpoints statistically tested? N

Blinding:
- Were patients blinded from treatment? N
- Were assessing investigators blinded from patient treatment? N

Allocation concealment:
- Was allocation concealment carried out? N

Lost to follow-up/discontinued/withdrawn:
- 5/16 (31.2%) total
  - 3/? withdrawn from Group 1
  - 2/? withdrawn from Group 2

108. Sánchez-Vazquez R, Briseño-Rodríguez G, Cardona-Muñoz EG, et al. Isosorbide dinitrate spray as therapeutic strategy for treatment of chronic venous ulcers. *Angiology.* 2008; 59(1):64-71.

Study Groups: Group 1: isorbide dinitrate (n = 15); Group 2: placebo spray (n = 30)

Statistical results:
- ITT analysis done for primary endpoint(s): Y
- **There were no primary endpoints identified**
- PAR
  - 2 weeks: 20.8% in Group 1 vs 9.3% in Group 2, p = .02
  - 4 weeks: 40.3% in Group 1 vs 28.3% in Group 2, p = .06
  - 6 weeks: 52.4% in Group 1 vs 37.3% in Group 2, p = .02
  - 8 weeks: 61.0%% in Group 1 vs 40.2% in Group 2, p = .01
  - 10 weeks: 66.4% in Group 1 vs 49.4% in Group 2, p = .04
  - 12 weeks: 71.3% in Group 1 vs 54.3% in Group 1, p = .05
- Histopathological analyses showed a significant increase in the hyperplasic capillaries in Group 1, p <.05

Power calculation:
- Was a power calculation for the primary endpoint reported, and if so, was it appropriate? N

Adjustment for multiplicity of statistical testing:
- Was an adjustment reported if there were more than 1 secondary endpoints statistically tested? N

Blinding:
- Were patients blinded from treatment? Y

For Peer Review

- Were assessing investigators blinded from patient treatment? Y

Allocation concealment:

- Was allocation concealment carried out? N

Lost to follow-up/discontinued/withdrawn: None reported.

109. Scalise A, Campitiello F, Della Corte A, et al. Enzymatic debridement: is HA-collagenase the right synergy? Randomized double-blind controlled clinical trial in venous leg ulcers. *Eur Rev Med Pharmacol Sci.* 2017;21(6):1421-1431.

Study Groups: Group 1: low molecular weight hyaluronic acid; Group 2: identical placebo ointment

Statistical results:

- ITT analysis done for primary endpoint(s): Y
- **Primary endpoint:** Mean (SD, median, range) debridement rate (% change), total: 67.5 (6.7, 86.9, -148 to 100) in Group 1 vs 59.0 (7.6, 74.4, -253 to 100) in Group 2, p = .1385
  - Mean (SD range) debridement rate at day 7,%: 45 (43 to 52) in Group 1 vs 35 (27 to 40) in Group 2, p = .2734
  - Mean (SD range) debridement rate at day 15,%: 65 (64 to 71) in Group 1 vs 54 (50 to 59) in Group 2, p = .0436
  - Mean (SD range) debridement rate at day 21,%: 69 (73 to 78) in Group 1 vs 63 (59 to 61) in Group 2, p = .2271
  - Mean (SD range) debridement rate at day 30,%: 76 (75 to 83) in Group 1 vs 65 (60 to 71) in Group 2, p = .1385
  - The group difference between the adjusted mean was 11.8% (95% CI: 0.35 to 23.3%) in favor of Group 1, p = .0436
  - PP analysis: 79.5 % in Group 1 vs 66.9 % in Group 2, p = .0031
- The number (%) of ulcers that achieved complete wound debridement by 15 days post-baseline: 20 (39.2 %) in Group 1 vs 6 (12.5%) in Group 2, p = .0025
  - PP analysis confirmed superiority of Group 1 at 15 days, p = .0002, and the other times points, with 18.9% of ulcers debrided at day 7 in the Group 1 vs 4.8% in Group 2, p = .0481, and 63.2% at the final visit in Group 1 vs 39.5% in Group 2, p = .0338
- Reduction in total area ≥50%, ≥75% and equal to 100% was observed in higher percentages of patients in group 1 vs Group 2, at any time point in overall sample, but differences between the Groups were not statistically significant in either ITT or PP population.
- Percentage of patients with full debridement rate in PP population
  - Day 7: 18.9 in Group 1 vs 4.8 in Group 2, p = .0481
  - Day 15: 47.4 in Group 1 vs 9.5 in Group 2, p = .0002
  - Day 21: 52.6 in Group 1 vs 25.6 in Group 2, p = .0124
  - Day Last: 63.2 in Group 1 vs 39.5 in Group 2, p = .0338
- The frequency of adverse events and serious adverse events was very similar in both Groups, no significant difference between Groups, p = NS

Power calculation:

For Peer Review

- Was a power calculation for the primary endpoint reported, and if so, was it appropriate? Y, N

Adjustment for multiplicity of statistical testing:

- Was an adjustment reported if there were more than 1 secondary endpoints statistically tested? N

Blinding:

- Were patients blinded from treatment? Y
- Were assessing investigators blinded from patient treatment? Y

Allocation concealment:

- Was allocation concealment carried out? N

Lost to follow-up/discontinued/withdrawn:

- 14/113 (12.4%) total
  - 8/58 (13.8%) withdrawn from Group 1
  - 6/55 (10.9%) withdrawn from Group 2

110. Schmutz JL, Meaume S, Fays S, et al. Evaluation of the nano-oligosaccharide factor lipido-colloid matrix in the local management of venous leg ulcers: results of a randomised, controlled trial. *Int Wound J.* 2008;5(2):172-182.

Study Groups: Group 1: nano-oligosaccharide factor; Group 2: collagen-oxidized regenerated cellulose

Statistical results:

- ITT analysis done for primary endpoint(s): Y
- **Primary endpoint:** median wound area relative reduction, ITT (n = 117) In ITT population (n = 117) median of area reduction was 54.4 in Group 1 vs 12.9 in Group 2, p = 0.286
  - Median of the wound area relative reduction, %, PP (n = 99): 61.1 in Group 1 vs 7.7 in Group 2, the mean difference between Groups was 33.6 (SD = 15) with a unilateral 95% CI lower limit 0f 8.6 not including the null value, p = .0059 Wilcoxon superiority test.
- Mean (SD, median) of wound area absolute reduction, cm^2, 2.3 (10.2, 4.2) in Group 1 vs 0.2 (10.4, 1.0) in Group 2, p = 0.01
- Mean (SD, median) wound of healing rate, cm^2/day: -0.016 (0.285,-0.056) in Group 1 vs 0.075 (0.475,-0.015), p = 0.029
- Percentage of patients that reached 40% of wound area reduction
  - 56 in Group 1 vs 35 in Group 2, p = .022 (18 ulcers were reepithelialized, 10 in Group 1 vs 8 in Group 2)
  - Ulcers ≤6 months: 58 in Group 1 vs 48 in Group 2, p = .488
  - Ulcers >6 months: 55 in Group 1 vs 26 in Group 2, p = .016
  - Logistic regression, including parameter of ulcer etiology, ulcer duration, extent of baseline granulation tissue and perilesional skin aspect in the model, Group 1 vs Group 2 odd ratio (OR) = 2.4 (95% CI = 1.1 to 5.3), p = .026, in favor of Group 1
  - Median time to reach 40% of WAR, days: 42 in Group 1 vs 84 in Group 2, p = 0.06 log-rank test

For Peer Review

    - Ulcer duration was a significant predictive factor with OR = 2.2 (95% CI = 1.0 to 4.9), p = .043 for ulcers of ≤6 months compared to >6 months
- Mean (SD, median) of wound healing parameters according to ulcer duration:
    - Ulcer duration ≤6 months: (Group 1, n = 26 vs Group 2, n = 25)
        - Ulcer area base line, cm^2: 8 (5.8, 5.9) in Group 1 vs 9.5 (8.1, 5.8), p = .435
        - Relative reduction, %: -36.5 (80.2, -63.5) in Group 1 vs -21.6 (86, -28.3), p = .608
        - Absolute reduction, cm^2: -3.4 (5.8, -4.6) in Group 1 vs -1.7 (11.4, -1.9), p = .559
        - Healing rate, cm^2/day: -0.057 (0.156, -0.055) in Group 1 vs 0.017 (0.487, -0.034), p = .224
        - No. (%) of ulcer with ≥ 40 WAR: 15 (57) in Group 1 vs 12 (48), p = .488
    - Ulcer duration >6 months: (Group 1, n = 31 vs Group 2, n = 35)
        - Ulcer area base line, cm^2: 14.4 (12.0, 11.0) in Group 1 vs 11.0 (8.7, 8.6), p = .193
        - Relative reduction, %: -26.5 (72.9, -44.3) in Group 1 vs 1.0 (70.3, -7.7), p = .044
        - Absolute reduction, cm^2: -1.3 (12.9, -4.1) in Group 1 vs 0.9 (9.6, 0.6), p = .019
        - Healing rate, cm^2/day: 0.358 (0.017, -0.059) in Group 1 vs 0.116 (0.469, -0.007), p = .027
        - No. (%) of ulcer with ≥ 40 WAR: 17 (54.8) in Group 1 vs 9 (25.7), p = .016

Power calculation:

- Was a power calculation for the primary endpoint reported, and if so, was it appropriate? Y, N

Adjustment for multiplicity of statistical testing:

- Was an adjustment reported if there were more than 1 secondary endpoints statistically tested? N

Blinding:

- Were patients blinded from treatment? N
- Were assessing investigators blinded from patient treatment? N

Allocation concealment:

- Was allocation concealment carried out? N

Lost to follow-up/discontinued/withdrawn:

- 41/117 (35.0%) total
    - 17/57 (29.8%) withdrawn from Group 1
    - 24/60 (40.0%) withdrawn from Group 2

111. Schumann H, Calow T, Weckesser S, Muller ML, Hoffmann G. Water-filtered infrared A for the treatment of chronic venous stasis ulcers of the lower legs at home: a randomized controlled blinded study. *Br J Derm.* 2011;165(3):541-551.

Study Groups: Group 1: Water-filtered infrared A radiation + visible light; Group 2: Visible light alone (control)

For Peer Review

Statistical results:

- ITT analysis done for primary endpoint(s): Y
- **Primary endpoints:** The integral of the relative ulcer area for each patient over 13 weeks standardized to an individual area of 1 showed a trend towards a smaller integral in Group 1 vs Group 2: median (IQR, range): 9.29 (5.3 to 12.3, 2.5 to 16.4) vs 10.67 (7.2 to 13.7, 2.4 to 13.7); median difference: -1.65; CI: -4.73 to 0.98; p = 0.21
    - Mean absolute reduction of wound area (cm^2) was much larger for Group 1 vs Group 2
        - Within 9 weeks: -151 vs -49 (median: -3.0, range: -37 to 4.5 vs -1.1, range: -34.4 to 20.5; median difference: 2.5; 95% CI: -6.2 to 0.2, p = .07)
    - Mean relative PAR (IQR, range) at:
        - 1 week: -6 (-10 to 0; -48 to 70) in Group 1 vs -5 (-8 to 5; -38 to 55) in Group 2, p = .63
        - 3 weeks: -20 (-28 to 0; -71 to 60) vs -8 (-33 to 3; -66 to 210); p = .37
        - 5 weeks: -28 (-57 to 0; -100 to 24) vs -25 (-55 to 7; -90 to 210), p = .58
        - 7 weeks: -39 (-75 to -8; -100 to 28) vs -19.5 (-59 to 5; -100 to 210), median difference: -20.5%, 95% CI: -49 to -4, p = .10
        - 9 weeks: -41 (-90 to -23; 110 to 23) vs -24 (-79 to 13; 110 to 210), p = .10
        - 13 weeks: -50 (-93 to -24; -100 to 49) vs -32 (-90 to 9; -100 to 210), p = .13
    - Mean relative PAR (IQR, range) of wounds with an initial wound size <10 cm^2 at:
        - 1 week: -8 (-24 to 0, -48 to 80) in Group 1 vs -7 (-12 to 3; -42 to 55) in Group 2, p = .48
        - 3 weeks: -28 (-43 to 10; -73 to 61) vs -19 (-42 to 10; -68 to 210), p = .28
        - 5 weeks: -35 (-78 to -2; -100 to 12) vs -35 (-65 to 23; -93 to 210), p = .62
        - 7 weeks: -60 (-92 to -21; -100 to 7) vs -21 (-75 to 19; -100 to 210), p = .14
        - 9 weeks: -84 (-105 to -20; -100 to 210)) vs -38.5 (-80 to 29; -100 to 210), p = .12
        - 13 weeks: -92 (-100 to 128; -100 to 27) vs -47 (-81 to 30; -199 to 210), median difference: -30%, 95% CI: -68 to 0, p = .11
    - Median overall wound assessment, based on a VAS of 0 to 100, (IQR, range) at:
        - Baseline: 50 (40-60; 10-74) in Group 1 vs 50 (46-60; 31-69) in Group 2, p = .74
        - 1 week: 60 (50-66; 41-79) vs 54 (46-63; 27-74), p = .39
        - 3 weeks: 63 (59-80; 50-90) vs 54 (40-71; 27-83), p =.047
        - 5 weeks: 70 (39-83; 50-100) vs 57.5 (40-74; 30-93), p = .20, median difference: 15, 95% CI: 0 to 25
        - 7 weeks: 74 (64-85; 43-100) vs 65 (40-77; 19-100), p = .018
        - 9 weeks: 85 (68-92; 50-100) vs 67.5 (40-82; 14-100), p = .012, median difference: 15, 95% CI: 3-30
        - 13 weeks: 85 (72-92; 10-100) vs 67.5 (40-85; 10-100), p= .017, median difference: 15, 95% CI: 2-35
    - Tendency to heal, % of patients at:
        - 1 week: 68 in Group 1 vs 42 in Group 2, p = .12

For Peer Review

        - 3 weeks: 72 vs 56, p = .44
        - 5 weeks: 75 vs 56, p = .28
        - 7 weeks: 75 vs 56, p = .28
        - 9 weeks: 84 (21/25) vs 50 (13/26), p = .023
        - 13 weeks: 75 vs 56, p = .28
    - Median (IQR, range) granulation, based on a VAS of 0-100 at:
        - Baseline: 20 (7-48; 0-95) in Group 1 vs 30 (16-49; 0-69) in Group 2, p = .41
        - 1 week: 30 (15-64; 0-94) vs 43 (23-49; 0-84), p = .69
        - 3 weeks: 60 (27-75; 0-100) vs 45 (20-73; 0-100), p = .45
        - 5 weeks: 70 (35-80; 15-100) vs 50 (20-78; 0-100), p = .20, median difference: 15, 95% CI: -5 to 35
        - 7 weeks: 75 (37-95; 10-100) vs 65 (20-79; 0-100), p = .036
        - 9 weeks: 90 (50-100; 15-100) vs 80 (20-89; 0-100), p = .036, median difference: 10, 95% CI: 0-30
        - 13 weeks: 90 (62-100; 30-100) vs 80 (20-95; 0-100), p = .047, median difference: 10, 95% CI: 0-40
    - Median (IQR, range) exudation, based on a VAS of 0-100 at:
        - Baseline: 50 (30-69; 0-100) in Group 1 vs 62 (30-75; 20-100) in Group 2, p = .62
        - 1 week: 50 (30-69; 0-80) vs 54 (30-69; 20-100), p = .77
        - 3 weeks: 38 (27-59; 10-100) vs 51 (30-62; 10-100), p = .49
        - 5 weeks: 30 (20-53; 0-80) vs 55 (30-69, 5-100), p = .075
        - 7 weeks: 30 (16-60; 0-80) vs 50 (30-60; 0-100), p = .19
        - 9 weeks: 30 (16-50; 0-80) vs 35 (22-54; 0-100), p = .32
        - 13 weeks: 25 (7-60; 0-100) vs 42.5 (18-60; 0-100), p = .27
- % of patients with increasing epithelialization; % with decreasing epithelialization at:
    - 1 week: 64; 13 in Group 1 vs 42; 21 in Group 2, p = .29
    - 3 weeks: 54; 9 vs 52; 21, p = .28
    - 5 weeks: 60; 12 vs 52; 26, p = .38
    - 7 weeks: 64; 9 vs 49; 31, p = .12
    - 9 weeks: 60; 31 vs 35; 8, p = .076
    - 13 weeks: 50; 17 vs 49; 22, p = .80
- Median (IQR, range) coatings/crusts, based on a VAS of 0-100 at:
    - Baseline: 50 (30-70; 0-100) in Group 1 vs 54 (30-70; 21-100) in Group 2, p = .89
    - 1 week: 30 (16-65; 0-100) vs 45 (30-71; 10-100), p = .26
    - 3 weeks: 37 (22-63; 0-100) vs 43 (34-70; 0-100), p = .34
    - 5 weeks: 20 (13-55; 0-85) vs 40 (30-70; 0-100), p = .080
    - 7 weeks: 30 (10-55, 0-100) vs 37.5 (28-61, 0-100), p = .14
    - 9 weeks: 20 (5-62; 0-100) vs 32.5 (20-50; 0-100), p = .42
    - 13 weeks: 18 (0-64; 0-100) vs 29 (17-61; 0-100), p = .30
- Median of the maximum pain (VAS) within the last 22 hours at
    - Baseline: 30 in Group 1 vs 25 in Group 2, p = NS
    - 13 weeks: 0 in both groups, p = NS
    - At 1 week, 6/25 (24%) in Group 1 and 0/26 in Group 2 experienced an acute

For Peer Review

reduction between the pain perceived immediately before and after the irradiation, p = .18

Power calculation:

- Was a power calculation for the primary endpoint reported, and if so, was it appropriate? N

Adjustment for multiplicity of statistical testing:

- Was an adjustment reported if there were more than 1 secondary endpoints statistically tested? N

Blinding:

- Were patients blinded from treatment? Y
- Were assessing investigators blinded from patient treatment? N

Allocation concealment:

- Was allocation concealment carried out? N

Lost to follow-up/discontinued/withdrawn:

- 6/51 (11.8%) total
  - 1/25 (4.0%) withdrawn from Group 1
  - 5/26 (19.2%) withdrawn from Group 2

112. Scondotto G, Aloisi D, Ferrari P, Martini L. Treatment of venous leg ulcers with sulodexide. *Angiology.* 1999;50(11):883-889.

Study Groups: Group 1: sulodexide + SOC (n = 52); Group 2: (n = 42)

Statistical results:

- ITT analysis done for primary endpoint(s): Y
- **Primary endpoint:** percent healed after 2 months:30 (58%) in Group 1 vs 15 (36%) in Group 2, p = .03
- Mean (SD) time to heal, days: 72 (64) in Group 1 vs 110 (137) in Group 2, p =.003; CI: -5 to 80
  - Mean (SD) time to heal for epidermal ulcers: 25 (20) in Group 1 vs 51 (21) in Group 2, p = .0001; CI: 12.1 to 39.3
  - Mean (SD) time to heal for dermal ulcers: 86 (66) in Group 1 vs 133 (51) in Group 2, p = .02; CI: 7.68 to 86.8
- Multiple linear analysis effect of variables on ulcer-healing times, Beta, T, p
  - Sex: -0.122297, -1.605, .11
  - Age: -0.091292, -1.193, .23
  - Duration of ulcer: 0.333304, 4.065, .0001
  - Etiology: -0.077937, -0.987, 0.32
  - Depth: -0.477954, 5.555, 0.0001
  - Group: -0.251753, -3.128, 0.0018

Power calculation:

- Was a power calculation for the primary endpoint reported, and if so, was it appropriate? N

Adjustment for multiplicity of statistical testing:

- Was an adjustment reported if there were more than 1 secondary endpoints statistically

For Peer Review

tested? N

Blinding:

- Were patients blinded from treatment? N
- Were assessing investigators blinded from patient treatment? N

Allocation concealment:

- Was allocation concealment carried out? N

Lost to follow-up/discontinued/withdrawn: None reported

113. Scriven JM, Taylor LE, Wood AJ, Bell PR, Naylor AR, London NJ. A prospective randomised trial of four-layer versus short stretch compression bandages for the treatment of venous leg ulcers. *Ann Royal Col Surg Eng.* 1998;80(3):215-220.

Study Groups: Group 1: Four-layer compression; Group 2: short stretch compression

Statistical results:

- ITT analysis done for primary endpoint(s): Y
- **Primary endpoint:** Ulcer healing rate at 1 year was 55% in Group 1 and 57% in Group 2 (chi-squared = 0.0, df = 1, p = 1.0)
- Data from groups were then combined to analyze associations with complete healing
    - Ulcer area >10 cm^2: 49% healed (17/35) vs ulcer area <10 cm^2: 62% (18/29); chi-squared = 1.17, p = .28
    - Ulcer duration >6 mos: 57% healed (27/47) vs duration <6 mos: 47% (8/17); chi-squared = 0.27, p = .60
    - Evidence of previous DVT: 72% healed (8/11) vs no evidence: 51% (27/53); chi squared = 1.74; p = .19
    - Deep venous reflux: 48% healed (15/31) vs no DVR: 61% (20/33); chi squared = 0.96, p = .33.
- Leg volumes at baseline: 2.44 in Group 1 vs 2.75 in Group 2, which decreased to 1.92 (8.8%) and 2.33 (13.1%), respectively, at 4 weeks, p =.34.

Power calculation:

- Was a power calculation for the primary endpoint reported, and if so, was it appropriate? N

Adjustment for multiplicity of statistical testing:

- Was an adjustment reported if there were more than 1 secondary endpoints statistically tested? N

Blinding:

- Were patients blinded from treatment? N
- Were assessing investigators blinded from patient treatment? N

Allocation concealment:

- Was allocation concealment carried out? N

Lost to follow-up/discontinued/withdrawn:

- 3/53 (5.7%)
    - 1/32 (3.1%) withdrawn from Group 1
    - 1/32 (3.1%) withdrawn from Group 2
    - 1 additional patient died, but does not say in which group

For Peer Review

114. Senet P, Bause R, Jorgensen B, Fogh K. Clinical efficacy of a silver-releasing foam dressing in venous leg ulcer healing: a randomised controlled trial. *Int Wound J.* 2014;11(6):649-655.

Study Groups: Group 1: Biatan Ag foam dressing that releases silver; Group 2: Biatan foam dressing only

Statistical results:

- ITT analysis done for primary endpoint(s): Y
- **Primary endpoints not defined.**
- Taking baseline area, age, and BMI as covariates into account and fixed effects: sex, ulcer age, effect of country and treatment into account, the mean relative reduction in wound area at 6 weeks was 42% for Group 1 vs 35% for Group, p = .0853
  - Strong significant effect of country was observed, p <.0001
  - At 10 weeks, the estimated treatment difference between Groups 1 and 2 increased to 13.6% with a significant country effect, p <.0001
- At 6 weeks, the mean Gilman healing rate was 0.67 mm/week for Group 1 vs 0.53 mm/week for Group 2
  - Estimated treatment difference taking into account the above covariates and fixed effects was 0.23 mm/week (p = .0852), for which the country effect was significant (p = .0015)
- French patients had a mean relative reduction in ulcer area of 44% for Group 1 and 22% for Group 2 with an estimated significant treatment difference of 22% at 6 weeks, p =.023
  - Their mean Gilman healing rate at 6 weeks was 0.63 mm/week for Group 1 vs 0.33 mm/week for Group 2, p = .0021
    - Taking covariates and fixed effects into account, the estimated treatment difference in Gilman rate was 0.41 mm/week in favor of Group 1 (p = .0021)

Power calculation:

- Was a power calculation for the primary endpoint reported, and if so, was it appropriate? Y, N

Adjustment for multiplicity of statistical testing:

- Was an adjustment reported if there were more than 1 secondary endpoints statistically tested? N

Blinding:

- Were patients blinded from treatment? Y
- Were assessing investigators blinded from patient treatment? y

Allocation concealment:

- Was allocation concealment carried out? N

Lost to follow-up/discontinued/withdrawn:

- 29/182 (15.9%) total
  - 11/88 (12.5%) withdrawn from Group 1
  - 18/94 (11.7%) withdrawn from Group 2

For Peer Review

115. Senet P, Bon FX, Benbunan M, et al. Randomized trial and local biological effect of autologous platelets used as adjuvant therapy for chronic venous leg ulcers. *J Vasc Surg.* 2003; 38(6):1342-1348.

Study Groups: Group 1: topically applied frozen autologous platelets; Group 2: placebo normal saline solution

Statistical results:

- ITT analysis done for primary endpoint(s): Y
- **Primary endpoint not identified**
- Group 1 had no significant influence on venous ulcer healing, p = NS
- Mean (SD) of baseline of ulcer diameter, cm^2: 13.7 (7.9) in Group 1 vs 10.9 (8.4) in Group 2, p = .9
- Number of healed ulcers: 1 in Group 1 (week 12) vs 1 in Group 2 (week 4), p >.05, no relapse occurred during 4 weeks after the end of treatment
- Mean (SD) linear healing rate, cm^2/day: 0.0033 (0.0061) in group 1 vs 0.0021 (0.0058) in Group 2, p = .47
- Linear healing of wound edge not statistically different between Groups at any time of study, p = NS
- Mean PAR: 26.2 in group 1 vs 15.2 in Group 2, p = .94
- Decrease in ulcer area tended to be greater in Group 1, but not statistically significant, p = NS
- Incidence of AEs: Not statistically significant, p = NS, including 1 wound infection in Group 1 vs 0 wound infection in Group 2, 1 thrombophlebitis during blood sample in Group 1 vs 0 in Group 2, and irritable dermatitis around wound: 2 in Group 1 vs 2 in Group 2, p =NS
- There was no evidence of adverse effects related to use treatment of Group 1, p = NS
- Concentration of growth factor and TIMP-1 (Tissue inhibitor of metalloproteinase) in wound fluid did not differ significantly between Groups during inclusion day or study, p = NS
- Concentration of IL-8 (Interleukin-8), KGF (Keratinocyte growth factor), VEFG (Vascular endothelial growth factor) were not significantly modified during study between Groups, p =NS
- TIMP-1 levels in wound fluid increase significantly during study in Group 1, p = .04
- Levels of VEGF, TIMP-1 or KGF, no significant difference was found between healed (n = 7) and unhealed (n = 8) ulcers in either Group, p = NS
- IL-8 levels were not significantly different between healed and unhealed in the beginning of study, p = NS, but at the end was lower in the healed group (29.8, SD = 10.4 ng/mg) vs unhealed group (69.7, SD = 35.7 ng/mg), p = .04
- Mean (SD, n) of concentrations in wound fluid of IL-8, VEGF, TIMP-1, KGF:
    - IL-8, ng/mg: Day 0: 97.2 (60.1, 7) in group 1 vs 57.0 (48.4, 6) in Group 2; Day 28: 67.1 (33.0, 8) vs 38.7 (18.3, 5); Day 56: 74.9 (91.4, 7) vs 24.5 (0.1, 2); Day 84: 52.3 (26.8, 7) vs 59.7 (50.1, 4); p = NS
    - VEGF, pg/mg: Day 0: 369 (123, 7) in Group 1 vs 379 (95, 6) in Group 2; Day 28:

For Peer Review

507 (408, 8) vs 351 (221, 5); Day 56: 520 (630, 8) vs 256 (20, 2); Day 84: 373 (250, 7) vs 579 (365, 4); p = NS
  - TIMP-1, ng/mg: Day 0: 7.4 (2.9, 7) in Group 1 vs 13.9 (7.2, 3) in Group 2; Day 28: 14.2 (7.3, 8) vs 20.5 (11.9, 5); Day 56: 13.7 (6.3, 7) vs 20.0 (0.8, 2); Day 84: 15.1 (7.9, 7) vs 18.1 (4.6, 3); p = NS
  - KGF, pg/mg: Day 0: 41 (37, 6) in group 1 vs 57 (53, 3) in Group 2; Day 28: 43 (18, 5) vs 46 (32, 4); Day 56: 81 (55, 7) vs 61 (18, 2); Day 84: 69 (26, 4) vs 78 (38, 2) in; p = NS

Power calculation:

- Was a power calculation for the primary endpoint reported, and if so, was it appropriate? N

Adjustment for multiplicity of statistical testing:

- Was an adjustment reported if there were more than 1 secondary endpoints statistically tested? N

Blinding:

- Were patients blinded from treatment? Y
- Were assessing investigators blinded from patient treatment? Y

Allocation concealment:

- Was allocation concealment carried out? N

Lost to follow-up/discontinued/withdrawn:

- 2/15 (13.3%) total
  - 1/8 (12.5%) withdrawn from Group 1
  - 1/7 (14.3%) withdrawn from Group 2

116. Serena TE, Carter MJ, Le LT, Sabo MJ, DiMarco DT. A multicenter, randomized, controlled clinical trial evaluating the use of dehydrated human amnion/chorion membrane allografts and multilayer compression therapy vs. multilayer compression therapy alone in the treatment of venous leg ulcers. *Wound Repair Regen.* 2014;22(6):688-693.

Study Groups: Group 1: 1 application of dehydrated human amnion/chorion membrane allograft (dHACA) + multilayer compression therapy (MLCT); Group 2: 2 applications of dHACA + MLCT; Group 3: MLCT alone

Statistical results:

- ITT analysis done for primary endpoint(s): N
- **Primary endpoint:** ≥40% reduction in wound size occurred in significantly more patients in Groups 1 and 2 vs Group 2 (33/53 [62%] vs 10/31 [32%], p = .005).
  - No statistical difference in size reduction for Groups 1 vs 2 (62% [16/26] vs 63/0% [17/27], p = NS)
  - Compared to Group 3 proportions, there was statistical significance in favor of Group 1 (p = .019) and Group 2 (p = .027)

Power calculation:

- Was a power calculation for the primary endpoint reported, and if so, was it appropriate? Y, Y

For Peer Review

Adjustment for multiplicity of statistical testing:

- Was an adjustment reported if there were more than 1 secondary endpoints statistically tested? Y

Blinding:

- Were patients blinded from treatment? N
- Were assessing investigators blinded from patient treatment? N

Allocation concealment:

- Was allocation concealment carried out? Y

Lost to follow-up/discontinued/withdrawn:

- 5/84 (6%) total
  - 2/53 (5.7%) withdrawn from Groups 1 and 2
  - 3/31 (9.7%) withdrawn from Group 3

117. Serra R, Buffone G, Molinari V, et al. Low molecular weight heparin improves healing of chronic venous ulcers especially in the elderly. *Int Wound J.* 2015;12(2):150-153.

Study Groups: Group 1: nadroparin (low molecular weight heparin) + SOC (n = 142); Group 2: SOC (n = 142)

Statistical results:

- ITT analysis done for primary endpoint(s): Y
- **There were no primary endpoints identified.**
- Healed ulcers at 12 months: 119 (83.8%) in Group 1 vs 86 (60.6%) in Group 2
  - Mean area ulcer healing per week, cm^2: 1.3 vs 0.87
  - Non-healing ulcers: 23 (16.2%) vs 58 (40.8%)
  - P <.00001
- Healing rates by age group:
  - 48-64 years, healed: 30/42 (71.4%) in Group 1 vs 37/39 (94.9%) in Group 2
    - Unhealed: 12/42 (28.6%) vs 9/39 (23.1%)
    - P <.322
  - 65-79 years, healed: 54/62 (87.1%) in Group 1 vs 39/67 (58.2%) in Group 2
    - Unhealed: 8/62 (12.9%) vs 31/67 (46.3%)
    - P <.0001
  - 80+ years, healed: 35/38 (91.1%) in Group 1 vs 10/36 (27/8%) in Group 2
    - Unhealed: 3/38 (7.9%) vs 17/36 (47.2%)
    - P <.00001
- Recurrence rates at 5 years
  - 48-64 years: 18/42 (42.8%) in Group 1 vs 17/39 (43.6%) in Group 2, p <.003
  - 65-79 years: 12/62 (19.4%) vs 38/67 (56.7%), p <.002
  - 80+ years: 8/38 (21%) vs 29/36 (80.6%), p <.002

Power calculation:

- Was a power calculation for the primary endpoint reported, and if so, was it appropriate? N

Adjustment for multiplicity of statistical testing:

- Was an adjustment reported if there were more than 1 secondary endpoints statistically

For Peer Review

tested? N

Blinding:

- Were patients blinded from treatment? N
- Were assessing investigators blinded from patient treatment? N

Allocation concealment:

- Was allocation concealment carried out? N

Lost to follow-up/discontinued/withdrawn: None reported

118. Serra R, Gallelli L, Buffone G, et al. Doxycycline speeds up healing of chronic venous ulcers. *Int Wound J.* 2015;12(2):179-184.

Study Groups: Group 1: doxycycline 20 mg b.i.d. for 3 months + basic treatment, n = 32; Group 2: basic treatment, n = 32

Statistical results:

- ITT analysis done for primary endpoint(s): Y
- **There were no primary endpoints identified.**
- Mean plasma levels (ng/ml) of MMP-9 evaluated via ELISA test: significantly lower levels in both plasma of Group 1 compared to Group 2 (p< .01), with significantly higher effects in high healing ulcers compared to low healing ulcers (p < .01)
    - Group 1: High-healing ulcers: T0: 124; T1: 83, T2: 78, T3: 55
        - Low-healing ulcers: T0: 122, T1: 108, T2: 90, T3: 80
    - Group 2: High-healing ulcers: T0: 139; T1: 114, T2: 109, T3: 100
        - Low-healing ulcers: T0: 121, T1: 120, T2: 109, T3: 108
- Mean plasma levels (ng/ml) of neutrophil gelatinase–associated lipocalin evaluated via ELISA test: significantly lower levels in Group 1 compared to Group 2 (p< .01), with significantly higher effects in high healing ulcers compared to low healing ulcers (p < .01)
    - Group 1: High-healing ulcers: T0: 139; T1: 90, T2: 78, T3: 61
        - Low-healing ulcers: T0: 140, T1: 120, T2: 89, T3: 79
    - Group 2: High-healing ulcers: T0: 131; T1: 120, T2: 92, T3: 99
        - Low-healing ulcers: T0: 139, T1: 130, T2: 111, T3: 100
- Mean wound fluid levels (ng/mcg total protein) of MMP-9 evaluated via ELISA test: significantly lower levels in Group 1 compared to Group 2 (p< .01), with significantly higher effects in high healing ulcers compared to low healing ulcers (p < .01)
    - Group 1: High-healing ulcers: T0: 247; T1: 200, T2: 155, T3: 100
        - Low-healing ulcers: T0: 250, T1: 222, T2: 200, T3: 138
    - Group 2: High-healing ulcers: T0: 247; T1: 246, T2: 255.5, T3: 199
        - Low-healing ulcers: T0: 250, T1: 249, T2: 245, T3: 244
- Mean wound fluid levels (ng/mcg total protein) of neutrophil gelatinase–associated lipocalin evaluated via ELISA test: significantly lower levels in Group 1 compared to Group 2 (p< .01), with significantly higher effects in high healing ulcers compared to low healing ulcers (p < .01)
    - Group 1: High-healing ulcers: T0: 251; T1: 205, T2: 150, T3: 54
        - Low-healing ulcers: T0: 250, T1: 225, T2: 195, T3: 95
    - Group 2: High-healing ulcers: T0: 251; T1: 237, T2: 236.5, T3: 205

For Peer Review

    - Low-healing ulcers: T0: 210, T1: 247, T2: 246, T3: 225
- Mean tissue expression of MMP-9 (arbitrary unit) evaluated via Western blot analysis: significantly lower levels in Group 1 compared to Group 2 (p< .01), with significantly higher effects in high healing ulcers compared to low healing ulcers (p < .01)
  - Group 1: High-healing ulcers: T0: 96; T1: 70, T2: 60, T3: 41
    - Low-healing ulcers: T0: 96, T1: 87, T2: 76, T3: 70
  - Group 2: High-healing ulcers: T0: 95; T1: 88, T2: 85, T3: 77
    - Low-healing ulcers: T0: 100, T1: 90, T2: 89, T3: 88
- Mean tissue expression of neutrophil gelatinase–associated lipocalin (arbitrary unit) evaluated via Western blot analysis: significantly lower levels in Group 1 compared to Group 2 (p< .01), with significantly higher effects in high healing ulcers compared to low healing ulcers (p < .01)
  - Group 1: High-healing ulcers: T0: 95; T1: 70, T2: 60, T3: 37
    - Low-healing ulcers: T0: 100, T1: 88, T2: 77, T3: 60
  - Group 2: High-healing ulcers: T0: 91; T1: 82, T2: 81, T3: 61
    - Low-healing ulcers: T0: 100, T1: 92, T2: 86, T3: 82
- Mean plasma levels (pg/ml) of VEGF evaluated via ELISA test: significantly lower levels in Group 1 compared to Group 2 (p< .01), with significantly higher effects in high healing ulcers compared to low healing ulcers (p < .01)
  - Group 1: High-healing ulcers: T0: 550; T1: 497, T2: 245, T3: 100
    - Low-healing ulcers: T0: 590; T1: 500, T2: 330, T3: 300
  - Group 2: High-healing ulcers: T0: 550, T1: 475, T2: 465, T3: 375
    - Low-healing ulcers: T0: 600, T1: 555, T2: 500, T3: 499
- Mean wound fluid levels (pg/ml) of VEGF evaluated via ELISA test: significantly lower levels in Group 1 compared to Group 2 (p< .01), with significantly higher effects in high healing ulcers compared to low healing ulcers (p < .01)
  - Group 1: High-healing ulcers: T0: 600; T1: 499, T2: 249, T3: 100
    - Low-healing ulcers: T0: 640, T1: 540, T2: 350, T3: 345
  - Group 2: High-healing ulcers: T0: 597; T1: 500, T2: 599, T3: 445
    - Low-healing ulcers: T0: 650, T1: 643, T2: 550, T3: 500

Power calculation:
- Was a power calculation for the primary endpoint reported, and if so, was it appropriate? N

Adjustment for multiplicity of statistical testing:
- Was an adjustment reported if there were more than 1 secondary endpoints statistically tested? N

Blinding:
- Were patients blinded from treatment? N
- Were assessing investigators blinded from patient treatment? N

Allocation concealment:
- Was allocation concealment carried out? N

Lost to follow-up/discontinued/withdrawn: None reported.

For Peer Review

119. Smeets R, Ulrich D, Unglaub F, Woltje M, Pallua N. Effect of oxidised regenerated cellulose/collagen matrix on proteases in wound exudate of patients with chronic venous ulceration. *Int Wound J.* 2008;5(2):195-203.

Study Groups: Group 1: oxidized regenerated cellulose (ORC)/collage matrix + hydrocolloid dressings (n = 17); Group 2: hydrocolloid dressings (control, n = 10)

Statistical results:

- ITT analysis done for primary endpoint(s): Y
- **There were no primary endpoints identified.**
- MMP-2 concentrations, pg/ml/mg Protein (outlier?)
    - Day 0: 265 (301) in Group 1 vs 360 (625) in Group 2, p = NS
    - Day 5: 260 (301) in Group 1 vs 300 (550) in Group 2, p = NS
    - Day 14: 265 (303) in Group 1 vs 205 (400) in Group 2, p = NS
    - Day 28: 215 (260) in Group 1 vs 303 (125) in Group 2, p = NS
    - Day 42: 215 (245) in Group 1 vs 310 (120) in Group 2, p = NS
    - Day 56: 180 (200) in Group 1 vs 125 (307) in Group 2, p = NS
- Gelatinase activities, %/ml/mg Protein (outlier?)
    - Day 0: 58 (68) in Group 1 vs 62 (75) in Group 2, p = NS
    - Day 5: 38 (49) in Group 1* vs 68 (82) in Group 2, p = NS
    - Day 14: 28 (32) in Group 1* vs 50 (75) in Group 2, p = NS
    - Day 28: 30 (30) in Group 1* vs 50 (70) in Group 2, p = NS
    - Day 42: 36 (52) in Group 1* vs 46 (64) in Group 2, p = NS
    - Day 56: 44 (64) in Group 1* vs 43 in Group 2, p = NS
    - P <.05 versus Day 0 for Group 1 only
- Elastase activities, µU/ml/mg Protein (outlier?)
    - Day 0: 360 (450) in Group 1 vs 405 (625) in Group 2, p = NS
    - Day 5: 155 (203) in Group 1* vs 395 (560) in Group 2, p <.05
    - Day 14: 150 (200) in Group 1* vs 399 (555) in Group 2, p <.05
    - Day 28: 110 (155) in Group 1* vs 405 (580) in Group 2, p = NS
    - Day 42: 110 (50) in Group 1* vs 340 (480) in Group 2, p <.05
    - Day 56: 140 (150) in Group 1* vs 250 (360) in Group 2, p <.05
    - P <.05 versus Day 0 for Group 1 only
- Plasmin activities, µU/ml/mg Protein (outlier?)
    - Day 0: 105 (140) in Group 1 vs 120 (230) in Group 2, p = NS
    - Day 5: 90 (115) in Group 1 vs 85 (149) in Group 2, p = NS
    - Day 14: 51 (68) in Group 1 vs 75 (140) in Group 2, p = NS
    - Day 28: 45 (53) in Group 1 vs 85 (160) in Group 2, p = NS
    - Day 42: 44 (51) in Group 1 vs 70 (180) in Group 2, p = NS
    - Day 56: 40 (49) in Group 1 vs 65 (150) in Group 2, p = NS
- The wound sizes in Groups 1 and 2 were not significantly different during the study, p = NS

Power calculation:

- Was a power calculation for the primary endpoint reported, and if so, was it appropriate? N

For Peer Review

Adjustment for multiplicity of statistical testing:

- Was an adjustment reported if there were more than 1 secondary endpoints statistically tested? N

Blinding:

- Were patients blinded from treatment? N
- Were assessing investigators blinded from patient treatment? N

Allocation concealment:

- Was allocation concealment carried out? N

Lost to follow-up/discontinued/withdrawn: None reported.

120. Solovăstru LG, Stîncanu A, De Ascentii A, Capparé G, Mattana P, Vâţă D. Randomized, controlled study of innovative spray formulation containing ozonated oil and alpha-bisabolol in the topical treatment of chronic venous leg ulcers. *Adv Skin Wound Care.* 2015;28(9):406-409.

Study Groups: Group 1: ozonated sunflower oil and alpha-bisabolol spray formulation (n = 15 patients, 20 ulcers); Group 2: standard epithelialization cream containing Vitamin A, vitamin E, talc, and zinc oxide (n = 14 patients, 17 ulcers)

Statistical results:

- ITT analysis done for primary endpoint(s): Y
- **Primary endpoint:** complete ulcer healing after 30 days of treatment, but no p value reported.
- Mean of ulcer surface area, cm^2:
    - Day 0: 4.36 in Group 1 vs 4.50 in Group 2, p = NS
    - Day 7: 3.14 in Group 1 vs 4.60 in Group 2, p = NS
    - Day 14: 2.19 in Group 1 vs 4.20 in Group 2, $p < .05$
    - Day 30: 1.85 in Group 1 vs 5.40 in Group 2, $p < .05$
    - There is a significant mean reduction in Group 1 comparing values in day 7 vs 0, 14 vs 7, and 30 vs 14, $p < .05$
    - There is not a significant mean reduction in Group 2 comparing values in day 7 vs 0, 14 vs 7, and 30 vs 14, p = NS
- Mean of wound area ratio, %:
    - Day 0: 100 in Group 1 vs 100 in Group 2, p = NS
    - Day 7: 64 in Group 1 vs 95 in Group 2, $p < .05$
    - Day 14: 42 in Group 1 vs 92 in Group 2, $p < .05$
    - Day 30: 24 in Group 1 vs 87 in Group 2, $p < .05$
    - There is a significant mean reduction in Group 1 comparing values in day 7 vs 0, 14 vs 7, and 30 vs 14, $p < .05$
    - There is not a significant mean reduction in Group 2 comparing values in day 7 vs 0, 14 vs 7, and 30 vs 14, p = NS

Power calculation:

- Was a power calculation for the primary endpoint reported, and if so, was it appropriate? N

Adjustment for multiplicity of statistical testing:

For Peer Review

- Was an adjustment reported if there were more than 1 secondary endpoints statistically tested? N

Blinding:

- Were patients blinded from treatment? N
- Were assessing investigators blinded from patient treatment? N

Allocation concealment:

- Was allocation concealment carried out? N

Lost to follow-up/discontinued/withdrawn: None reported.

121. Somani A, Rai R. Comparison of efficacy of autologous platelet-rich fibrin versus saline dressing in chronic venous leg ulcers: a randomised controlled trial. *J Cutan Aesthet Surg.* 2017; 10(1):8-12.

Study Groups: Group 1: Platelet rich fibrin, n = 9; Group 2: saline dressings, n = 6

Statistical results:

- ITT analysis done for primary endpoint(s): Y
- **There were no primary endpoints identified. Most endpoints were not analyzed with statistical tests.**
- Mean (SD) percent reduction in ulcer size at 4 weeks: 85.5% (20.4%) in Group 1 vs 42.7% (18.5) in Group 2, $t = 4.11$, $p < .001$

Power calculation:

- Was a power calculation for the primary endpoint reported, and if so, was it appropriate? N

Adjustment for multiplicity of statistical testing:

- Was an adjustment reported if there were more than 1 secondary endpoints statistically tested? N

Blinding:

- Were patients blinded from treatment? N
- Were assessing investigators blinded from patient treatment? N

Allocation concealment:

- Was allocation concealment carried out? N

Lost to follow-up/discontinued/withdrawn: None reported.

122. Stacey MC, Mata SD, Trengove NJ, Mather CA. Randomised double-blind placebo controlled trial of topical autologous platelet lysate in venous ulcer healing. *Eur J Vasc Surg*. 2000;20(3):296-301.

Study Groups: Group 1: Platelet lysate; Group 2: placebo

Statistical results:

- ITT analysis done for primary endpoint(s): Y
- **Primary endpoint:** Ulcer healing was identified in power calculation, but no p values given.
- Covariates' influence on healing using Cox regression analysis
    - Lysate or placebo: $\beta = -0.126$, $p = .37$
    - Ulcer area: $\beta = -0.053$, $p = .02$

For Peer Review

  - Duration of current ulcer: β = -0.027, $p = .10$
  - Duration of ulcer history: β = -0.001, $p = .11$
  - No. ulcer episode: β = -0.038, $p = .21$
  - Longest unhealed ulcer: β = -0.006, $p = .41$
  - Leg: β = 0.14, $p = .29$
  - Sex: β = 0.12, $p = .39$

Power calculation:

- Was a power calculation for the primary endpoint reported, and if so, was it appropriate? Y, N

Adjustment for multiplicity of statistical testing:

- Was an adjustment reported if there were more than 1 secondary endpoints statistically tested? N

Blinding:

- Were patients blinded from treatment? Y
- Were assessing investigators blinded from patient treatment? Y

Allocation concealment:

- Was allocation concealment carried out? N

Lost to follow-up/discontinued/withdrawn:

- 11/86 (12.8%) total
  - 5/42 (11.9%) withdrawn from Group 1
  - 6/44 (13.6%) withdrawn from Group 2

123. Szewczyk MT, Jawien A, Cierzniakowska K, Cwajda-Bialasik J, Moscicka P. Comparison of the effectiveness of compression stockings and layer compression systems in venous ulceration treatment. *Arch Med Sci.* 2010;6(5):793-799.

Study Groups: Group 1: elastic compression class II in the form of knee-length compression stockings (n = 15); Group 2: 2-layer compression (cotton wool and cotton band plus compression bandage [short stretch] (n = 16); Group 3: 4-later compression (cotton wool and cotton band, crepe bandage, elasticated bandage – long-stretched, cohesive bandage) (n = 15)

Statistical results:

- ITT analysis done for primary endpoint(s): Y
- **No primary endpoints identified**
- Mean loss of ulcer area during 12 weeks was 0.42 cm^2/week ($t_{par} = 6.28$, $p < .001$); changes of ulcer area in each group were significant ($p < .001$), but inter-group differences were not found ($H = 3.512$, $p > .05$ after 2 weeks; $H = 3.601$, $p > .05$ after 4 weeks; $H = 3.71$, $p > .05$ after 8 weeks; $H = 4.45$, $p > .05$ after 12 weeks)
  - Ulcer area decreased on average by 1.01 cm^2/week during first 2 weeks, 0.94 cm^2/week during the next 2 weeks, and 0.56 cm^2/week after 8 weeks
  - After 2 weeks, cm^2/week reduction: Group 1: 0.84, $t_{par} = 4.227$, $p < .001$; Group 2: 1.18, $t_{par} = 5.903$, $p < .001$; Group 3: 1.3, $t_{par} = 4.33$, $p < .001$
  - After 4 weeks, cm^2/week reduction: Group 1: 0.71, $t_{par} = 4.77$, $p < .01$; Group 2: 1.01, $t_{par} = 5.18$, $p < .001$; Group 3: 1.1, $t_{par} = 5.77$, $p < .001$
  - After 8 weeks, cm^2/week reduction: Group 1: 0.52, $t_{par} = 5.02$, $p < .001$; Group 2:

For Peer Review

0.64 , t_{par} = 4.867, p <.001; Group 3: 0.66, t_{par} = 5.85, p <.001
    - After 12 weeks, cm^2/week reduction: Group 1: 0.44, t_{par} = 4.867, p <.001; Group 2: 0.55, t_{par} = 5.11, p <.001; Group 3: 0.63, t_{par} = 6.156, p <.001
    - Greatest weekly area loss in Group 3 with a mean loss of area during the 12 weeks at 0.63 cm^2/week compared to 0.55 cm^2/week in Group 2 and 0.44 cm^2/week in Group 1
- Difference in proportion of healed area was 1.3%-8.7% after 2 weeks; 4.3%-16.2% after 4 weeks, 8.6%-17% after 8 weeks, and 4.2%-15% after 12 weeks
    - Mean PAR after 2 weeks: Group 1: 35.9%, Group 2: 44.6%, Group 3: 43.3%
    - Mean PAR after 4 weeks: Group 1: 60.5%, Group 2: 76.7%, Group 3: 72.4%
    - Mean PAR after 8 weeks: Group 1: 75.3%, Group 2: 92.3%, Group 3: 83.7%
    - Mean PAR after 12 weeks: Group 1: 83.1%, Group 2: 98.1%, Group 3: 93.9%
    - There were no significant differences in area changes among groups
- Complete healing achieved in 8/15 (53.3%) in Group 1; 10/16 (62.5%) in Group 2, and 9/15 (60%) in Group 3 (p > .05) at 12 weeks.
    - No. healed ulcers after 2 weeks: 0 in all groups
    - No. healed ulcers after 4 weeks: Group 1: 1 (6.7%); Group 2: 1 (6.2%); Group 3: 0.
    - No. healed ulcers after 4 weeks: Group 1: 2 (13.3%); Group 2: 4 (25%); Group 3: 2 (13.3%).
- Difference in mean CEAP C-6 score of all patients was 3.6 points (from 11.9 to 8.3, p <.001)
    - Differences were significant in each group: 3.5 points gained in Group 1, 3.5 points gained in Group 2, and 4.3 points gained in Group 3
        - Group 1: from 11.7 to 8.2, t_{par} = 11.16 (p <.001)
        - Group 2: from 11.25 to 7.75, t_{par} = 10.63 (p <.001)
        - Group 3: from 12.6 to 8.3, t_{par} = 11.72 (p <.001)
    - t_{par} = 15.64 (p <.001)
    - There were no significant differences among groups (H = 3.102, p > .05)

Power calculation:
- Was a power calculation for the primary endpoint reported, and if so, was it appropriate? N

Adjustment for multiplicity of statistical testing:
- Was an adjustment reported if there were more than 1 secondary endpoints statistically tested? N

Blinding:
- Were patients blinded from treatment? N
- Were assessing investigators blinded from patient treatment? N

Allocation concealment:
- Was allocation concealment carried out? N

Lost to follow-up/discontinued/withdrawn: None reported.

124. Taddeucci P, Pianigiani E, Colletta V, Torasso F, Andreassi L, Andreassi A. An evaluation of Hyalofill-F plus compression bandaging in the treatment of chronic venous ulcers. *J*

For Peer Review

Wound Care. 2004;13(5):202-204.

Study Groups: Group 1: partial benzyl ester derivative of hyaluronan + compression; Group 2: paraffin gauze + compression

Statistical results:

- ITT analysis done for primary endpoint(s): Y
- **Primary endpoint:** Mean PAR (cm^2 decrease) at 8 weeks: 33% (8.1) in Group 1 vs 1.8% (0.4) in Group 2, with a group difference of 7.7 cm^2, p = .0019
- Mean (SD) epithelialization rate per day, cm^2: -0.129 (0.17) in Group 1 vs -0.008 (0.05) in Group 2, p = .033
- % healed at week 8: 100% in Group 1 vs 42% in Group 2, p = .005
- % with leveling of wound margins: 100% in Group 1 vs 50% in Group 2, p = .044
- Maceration, present in 50% of ulcers at baseline, gone by week 8 in 100% of Group 1 vs 58% of Group 2, p = .005
- There were significant group differences in pain intensity (p = .004) and frequency (p = .002), favoring Group 1
- There were significant group differences in overall efficacy (p = .017), interaction with the wound bed (p = .001), interaction with exudate (p = .009), and tolerability (p = .001), favoring Group 1

Power calculation:

- Was a power calculation for the primary endpoint reported, and if so, was it appropriate? N

Adjustment for multiplicity of statistical testing:

- Was an adjustment reported if there were more than 1 secondary endpoints statistically tested? N

Blinding:

- Were patients blinded from treatment? N
- Were assessing investigators blinded from patient treatment? N

Allocation concealment:

- Was allocation concealment carried out? N

Lost to follow-up/discontinued/withdrawn:

- 6/24 (25.0%) total
    - 1/12 (8.3%) withdrawn from Group 1
    - 5/12 (41.7%) withdrawn from Group 2

125. Taradaj J, Franek A, Brzezinska-Wcislo L. Randomized trial of medical compression stockings versus two-layer short-stretch bandaging in the management of venous leg ulcers. *Phlebologie.* 2009;38(4):157-163.

Study Groups: Group 1: Compression stockings providing 25-32 mmHg, n =40 ; Group 2: short-stretch bandaging, n = 40

Statistical results:

- ITT analysis done for primary endpoint(s): Y
- **There were no primary endpoints identified.**
- Mean (SD) Gilman index (cm):

For Peer Review

  - Group 1: 0.18 (0.13) at baseline vs 0.89 (0.55) posttreatment, p = .001
  - Group 2: 0.19 (0.12) at baseline vs 0.55 (0.34) posttreatment, p = .001
- Mean (SD) total area (cm^2):
  - Group 1: 20.6 (14) at baseline vs 10 (9.7) posttreatment, p = .001
  - Group 2: 20.3 (13.1) at baseline vs 15 (13.9) posttreatment, p = .001
- Mean (SD) length (cm):
  - Group 1: 6.2 (2) at baseline vs 3.4 (3) posttreatment, p = .001
  - Group 2: 6.1 (2.1) at baseline vs 4.3 (3.1) posttreatment, p = .001
- Mean (SD) width (cm):
  - Group 1: 4.1 (1.5) at baseline vs 2.7 (2.1) posttreatment, p = .001
  - Group 2: 4.1 (1.4) at baseline vs 3.4 (2.1) posttreatment, p = .001
- Mean (SD) volume (cm^3):
  - Group 1: 3.7 (4) at baseline vs 0.73 (1.1) posttreatment, p = .001
  - Group 2: 3.3 (4.1) at baseline vs 0.58 (1.3) posttreatment, p = .001
- Mean (SD) pus-covered area (cm^2):
  - Group 1: 12.5 (8.4) at baseline vs 0.52 (0.99) posttreatment, p = .001
  - Group 2: 12 (9.8) at baseline vs 3.3 (4.2) posttreatment, p = .001
- Mean (SD) granulation area (cm^2):
  - Group 1: 8 (6.2) at baseline vs 9.5 (12) posttreatment, p = .01
  - Group 2: 8.3 (5.8) at baseline vs 11.8 (13) posttreatment, p = .01
- 15/40 (37.5%) in Group 1 vs 5/40 (12.5%) in Group 2 completely healed, p ≤.001
  - For patients with isolated superficial reflux, healing rates at 2 months were 45.4% (10/22) in Group 1 vs 18.2% (4/22) in Group 2, p ≤.01
  - For patients with superficial + deep reflux, the healing rates were 27.8% (5.18) in Group 1 and 5.6% (1/18) in Group 2, p ≤.01
- Relative change in total surface area (61.6% in Group 1 vs 23.7% in Group 2), length (41.7% in 1 vs 28% in 2), width (46.2% in 1 vs 29.3% in 2), and volume (82% in 1 vs 40% in 2) were significantly different in favor of Group 1, p ≤.01
- Statistically more decrease of pus (p ≤.0001) and greater promotion of granulation degree (p ≤.01) were observed in Group 1 (ΔR = 96.2%, ΔZ = 66%) than in Group 2 (ΔR = 50.1%, ΔZ = 40.2%)
- Correlation between changes of total area and length: R = 0.83, p <.001
- Correlation between changes of total area and width, R = 0.80, p<.001
- Correlation between changes of total area and volume, R = 0.66, p = .002
- In Group 2, the correlation between change of total area and length was 0.65 (p ≤.01), total area and width was 0.64 (p ≤.01), total area and volume was 0.56 (p ≤.01). This was more significant in Group 1 (no data provided).

Power calculation:

- Was a power calculation for the primary endpoint reported, and if so, was it appropriate? N

Adjustment for multiplicity of statistical testing:

- Was an adjustment reported if there were more than 1 secondary endpoints statistically tested? N

Blinding:
- Were patients blinded from treatment? N
- Were assessing investigators blinded from patient treatment? N

Allocation concealment:
- Was allocation concealment carried out? Y

Lost to follow-up/discontinued/withdrawn: None reported.

126. Taradaj J, Franek A, Brzezinska-Wcislo L, Blaszczak E, Polak A. The use of therapeutic ultrasound in venous leg ulcers: a randomized, controlled clinical trial. *Phlebology.* 2008;23(4):178-183.

Study Groups: Group 1: surgery + ultrasound + compression stockings + drug therapy (n = 21); Group 2: surgery + compression + drug therapy (n = 20); Group 3: ultrasound + compression + drug therapy (n = 20); Group 4: compression + drug therapy (n = 20)

Statistical results:
- ITT analysis done for primary endpoint(s): Y
- **Primary endpoints:** change in surface area and volume
    - Mean relative change of total surface area, %: 58.21 in Group 1 vs 58.36 in Group 2 vs 56.67 in Group 3 vs 36.09 in Group 4, significant differences between Groups 1 vs 4, p = .004; 2 vs 4, p = .004; 3 vs 4, p = .004; in favor of 1, 2, 3, respectively. There were no differences in Groups 1 vs 2, p >.05; 1 vs 3, p >.05; 2 vs 3, p >.05
    - Mean relative change of volume, %: 70.23 in Group 1 vs 69.21 in Group 2 vs 71.22 in Group 3 vs 46.88 in Group 4, significant differences between Groups 1 vs 4, p = .01; 2 vs 4, p = .01; 3 vs 4, p = .01; in favor of 1, 2, 3, respectively. There were no differences in Groups 1 vs 2, p >.05; 1 vs 3, p >.05; 2 vs 3, p >.05
- Mean (SD) ulcer size at 7 weeks, cm^2: 12.66 (3.04) in Group 1, n = 6 vs 13.87 (4.01) in Group 2, n = 6 vs 10.97 (34.21) in Group 3, n = 6 vs 9.79 (3.66) in Group 4, n = 3, significant differences between Groups 1 vs 4, p = .02; 2 vs 4, p = .02; 3 vs 4, p = .02; in favor of 1, 2, 3, respectively
- Mean Gilman index, cm: 0.92 in Group 1 vs 0.91 in Group 2 vs 0.92 in Group 3 vs 0.61 in Group 4, significant differences between Groups 1 vs 4, p = .002; 2 vs 4, p = .002; 3 vs 4, p = .002; in favor of 1, 2, 3, respectively. There were no differences in Groups 1 vs 2, p >.05; 1 vs 3, p >.05; 2 vs 3, p >.05
- Mean relative change of length, %: 39.99 in Group 1 vs 41.21 in Group 2 vs 38.89 in Group 3 vs 26.64 in Group 4, significant differences between Groups 1 vs 4, p = .01; 2 vs 4, p = .01; 3 vs 4, p = .01; in favor of 1, 2, 3, respectively. There were no differences in Groups 1 vs 2, p >.05; 1 vs 3, p >.05; 2 vs 3, p >.05
- Mean relative change of width, %: 42.55 in Group 1 vs 43.13 in Group 2 vs 40.78 in Group 3 vs 28.63 in Group 4, significant differences between Groups 1 vs 4, p = .01; 2 vs 4, p = .01; 3 vs 4, p = .01; in favor of 1, 2, 3, respectively. There were no differences in Groups 1 vs 2, p >.05; 1 vs 3, p >.05; 2 vs 3, p >.05

Power calculation:
- Was a power calculation for the primary endpoint reported, and if so, was it

appropriate? Y, N

Adjustment for multiplicity of statistical testing:

- Was an adjustment reported if there were more than 1 secondary endpoints statistically tested? N

Blinding:

- Were patients blinded from treatment? N
- Were assessing investigators blinded from patient treatment? N

Allocation concealment:

- Was allocation concealment carried out? N

Lost to follow-up/discontinued/withdrawn: None reported.

127. Taradaj J, Franek A, Cierpka L, Brzezinska-Wcislo L, Blaszczak E, Polak A. Early and Long-term results of physical methods in the treatment of venous leg ulcers: randomized controlled trial. *Phlebology.* 2011;26(6):237-245.

Study Groups:

- Group 1: surgical operation + high-voltage stimulation (HVS) + drug therapy, n = 40
- Group 2: surgical operation + ultrasound (U/S) therapy + drug therapy, n = 33
- Group 3: surgical operation + low-level laser therapy (LLLT) + drug therapy, n = 30
- Group 4: surgical operation + compression + drug therapy, n = 35
- Group 5: surgical operation + drug therapy, n = 37
- Group 6: conservative treatment + HSV+ drug therapy, n = 32
- Group 7: conservative treatment + U/S + drug therapy, n = 20
- Group 8: conservative treatment + LLLT + drug therapy, n = 21
- Group 9: conservative treatment + compression + drug therapy, n = 30
- Group 10: conservative treatment + drug therapy, n = 27
- For follow-up and outcome measurements, patients with healed ulcers from Group 4 and 9 were divided into 4 subgroups: 4a and 9a who finished compression therapy at 7 weeks time and 4b and 9b who continued compression therapy for 2 years.

Statistical results:

- ITT analysis done for primary endpoint(s): Y
- **Primary:** Percent healed, differences between groups
    - Groups 1 and 4: 37.3% vs 53.1, p =.03
    - Groups 2 and 4: 30.1% vs 53.1%, p = .03
    - Groups 3 and 4: 32.3% vs 53.1%, p = .03
    - Groups 4 and 5: 53.1% vs 32.2%, p = .03
    - Groups 4 and 6: 53.1% vs 30.2%, p = .03
    - Groups 4 and 7: 53.1% vs 30.1%, p = .03
    - Groups 4 and 8: 53.1% vs 13.5%, p = .002
    - Groups 4 and 9: 53.1% vs 30.8%, p = .03
    - Groups 4 and 10: 53.1% vs 11.4%, p = .002
    - Percentage healed for patients with isolated superficial reflux in Groups 1-5: Group 1: 38.2%, Group 2: 38.9%, Group 3: 44.1%, Group 4: 66.7%, Group 5: 42.1%

For Peer Review

        - P (1,2) > .05; P (1,3) >. 05; P (1,4) = .01; P (1,5) > .05; P (2,3) >.05; P (2,4) = .01; P (2,5) > .05; P (3,4) = .01; P (3,5) > .05; P (4,5) = .01
    - Percentage healed for patients with superficial and deep reflux in Groups 1-5: Group 1: 36.4%, Group 2: 36%, Group 3: 20.3%, Group 4: 39.0%, and Group 5: 22.2%
        - P (1,2) >.05; P (2,4) >0.05; P (1,3) = .03; P (2,5) = .03; P (1,4) >.05; P (3,5) = .03; P (1,5) = .03; P (3,5) >.05; P (2,3) = .03; P (4,5) = .03
    - Percentage healed for patients with isolated superficial reflux in Groups 6-10: Group 6: 40.5%, Group 7: 39%, Group 8: 16.9%, Group 9: 42.8%, Group 10: 15.3%
        - P (6,7) > .05; P (6,8) =.003; P (6,9) >.05; P (6,10) = .003; P (7,8) = .003; P (7, 9) >.05; P (7,10) = .003; P (8,9) = .003; P (8,10) > .05
    - Percentage healed for patients with superficial and deep reflux in Groups 6-10: Group 6: 19.8%, Group 7: 20%, Group 8: 9.9%, Group 9: 18.7%, and Group 10: 7.1%
        - P (6,7) > .05; P (7,9) >.05; P (6,8) = .003; P (7, 10) = .003; P (6,9) >.05; P (8,9) = .003; P (6,10) = .003; P (8,9) >.05; P (7,8) = .003; P (8,9) = .003
- **Primary:** Ulcer recurrence rates at 2 years were significantly lower in Group 4b (18.7%) compared with other groups: 1: 25.9%, p = .01; 2: 29.7%, p =.01; 3: 32.1%, p =.01; 4a: 34.7%, p =.01; 5: 37.5%, p = .01; 9b: 37.5%, p = .01; and 6, 7, 8, 9a, and 10: 57.5%, p <.001.
    - For patients with isolated superficial reflux, recurrence rates at 2 years were much lower in Groups 1 (14.3%), 2 (16.6%), 3 (14.3%), 4a (25%), 4b (25%) and 5 (25%) than in Groups 6 (40%), 7 (40%), 8 (50%), 9a (40%), 9b (25%) and 10 (50%), p =.03
        - There were no statistical differences in recurrence rates among groups 1-5 (p >.05)
    - For patients with superficial and reflux, recurrence rates at 2 years were much lower in Group 4b (13%) compared with Groups 1 (37.5%), 2 (42.7%), 3 (50%), 4a (44%), 5 (50%), and 9b (50%), p = .01 and compared with Groups 6 (75%), 7 (75%), 8 (75%), 9a (75%), and 10 (75%), p <.001.

Power calculation:

- Was a power calculation for the primary endpoint reported, and if so, was it appropriate? N

Adjustment for multiplicity of statistical testing:

- Was an adjustment reported if there were more than 1 secondary endpoints statistically tested? N

Blinding:

- Were patients blinded from treatment? N
- Were assessing investigators blinded from patient treatment? N

Allocation concealment:

- Was allocation concealment carried out? Y

Lost to follow-up/discontinued/withdrawn: None reported.

For Peer Review

128. Taradaj J, Franek A, Cierpka L. Failure of low-level laser therapy to boost healing of venous leg ulcers in surgically and conservatively treated patients. *Phlebologie.* 2008;37:241-246.

Study Groups: Group 1: surgery + low-level laser therapy (LLLT) + compression stockings + drug therapy (n =22); Group 2: surgery + compression and drug therapy (n = 20); Group 3: LLLT + compression + drug therapy (n = 21); Group 4: compression + drug therapy (n = 20)

Statistical results:

- ITT analysis done for primary endpoint(s): Y
- **There were no primary endpoints identified.**
- No (%) healed at 7 weeks: 8 (36.4%) in Group 1; 7 (35%) in Group 2; 3 (14.3%) in Group 3; 3 (15%) in Group 4, with significant differences favoring Groups 1 and 2 between:
    - Groups 1 and 3, p = .02
    - Groups 1 and 4, p = .02
    - Groups 2 and 3, p = .02
    - Groups 2 and 4, p = .02
- Group differences in relative change of total surface area:
    - Groups 1 and 3: 60.1% vs 27.9%, p = .002
    - Groups 1 and 4: 60.1% vs 28%, p = .002
    - Groups 2 and 3: 60% vs 27.9%, p = .002
    - Groups 2 and 4: 60% vs 28%, p = .002
- Group differences in relative change of volume:
    - Groups 1 and 3: 69.9% vs 41.9%, p = .01
    - Groups 1 and 4: 69.9% vs 41%, p = .01
    - Groups 2 and 3: 68.2% vs 41.9%, p = .01
    - Groups 2 and 4: 68.2% vs 41%, p = .01
- Group differences in relative change of length:
    - Groups 1 and 3: 43.4% vs 29.9%, p = .01
    - Groups 1 and 4: 43.4% vs 29.8%, p = .01
    - Groups 2 and 3: 43% vs 29.9%, p = .01
    - Groups 2 and 4: 43% vs 29.8%, p = .01
- Group differences in relative change of width:
    - Groups 1 and 3: 46.6% vs 30.9%, p = .006
    - Groups 1 and 4: 46.6% vs 31.4%, p = .006
    - Groups 2 and 3: 47% vs 30.9%, p = .006
    - Groups 2 and 4: 47% vs 31.4%, p = .006
- There were no statistical differences in all of the examined parameters between Groups 1 and 2 (p >.05) and Groups 3 and 4 (p >.05)

Power calculation:

- Was a power calculation for the primary endpoint reported, and if so, was it appropriate? N

Adjustment for multiplicity of statistical testing:

- Was an adjustment reported if there were more than 1 secondary endpoints statistically

For Peer Review

tested? N

Blinding:

- Were patients blinded from treatment? N
- Were assessing investigators blinded from patient treatment? N

Allocation concealment:

- Was allocation concealment carried out? N

Lost to follow-up/discontinued/withdrawn: None reported.

129. Toledo RR, Santos M, Schnaider TB. Effect of pycnogenol on the healing of venous ulcers. *Ann Vasc Surg.* 2017;38:212-219.

Study Groups: Group 1: pycnogenol; Group 2: diosmin/hesperidin

Statistical results:

- ITT analysis done for primary endpoint(s): N
- **There were no primary endpoints identified.**
- Mean (median) of ulcer area, cm^2: Baseline = 7.02 (4.78), Day 90 = 4.31 (1.78) in Group 1, n = 14, Friedman ANOVA, x2 = 72.94, p <.0001 vs Baseline = 6.77 (4.01), Day 90 = 4.64 (1.44) in Group 2, n = 13, Friedman ANOVA, x2 = 37.65, p <.0001
- A significant decrease in ulcer area from baseline was observed earlier in Group 1 after 45 days of treatment: Baseline = 7.02 (4.78), Day 45 = 5.53 (3.12), p <.05 vs Group 2 after 60 days of treatment: Baseline = 6.77 (4.01), Day 45 = 5.27 (2.38), p <.05
- Group 1 showed a trend toward a more favorable treatment effect on ulcer healing compared with Group 2, but without significant differences between all groups at all time points, p = NS
- Mean (median) of circumference of third proximal of ankle, cm: Baseline = 36.64 (36.5), Day 90 = 33.35 (33.00) in Group 1, n = 14, Friedman ANOVA, x2 = 33.17, p <.0001 vs Baseline = 37.46 (37.00), Day 90 = 35.38 (35.00) in Group 2, n = 13, Friedman ANOVA, x2 = 16.22, p = .0126
- Mean (median) of circumference of and distal third of the ankle, cm: Baseline = 23.85 (24.00), Day 90 = 22.00 (21.00) in Group 1, n = 14, Friedman ANOVA, x2 = 20.41, p = .0023 vs Baseline = 25.07 (24.00), Day 90 = 23.07 (23.00) in Group 2, n = 13, Friedman ANOVA, x2 = 16.15, p = .0130
- A significant decrease from baseline in the circumference of the proximal third of the ankle was observed earlier in Group 1 after 45 days of treatment: Baseline = 36.64 (36.50), Day 45 = 34.67 (33.75), p <.0001 vs Group 2 after 90 days of treatment: Baseline = 37.46 (4.01), Day 90 = 35.38 (35.00), p =0.0126
    - Without significant differences between all groups at all time points in the circumference and distal third of the ankle, p = NS

Power calculation:

- Was a power calculation for the primary endpoint reported, and if so, was it appropriate? Y, N

Adjustment for multiplicity of statistical testing:

- Was an adjustment reported if there were more than 1 secondary endpoints statistically tested? N

Blinding:

- Were patients blinded from treatment? N
- Were assessing investigators blinded from patient treatment? N

Allocation concealment:

- Was allocation concealment carried out? N

Lost to follow-up/discontinued/withdrawn:

- 3/30 (10.0%) total
    - 1/15 (6.7%) withdrawn from Group 1
    - 2/15 (13.3%) withdrawn from Group 2

130. Tumino G, Masuelli L, Bei R, Simonelli L, Santoro A, Francipane S. Topical treatment of chronic venous ulcers with sucralfate: a placebo controlled randomized study. *Int J Mol Med.* 2008;22(1):17-23.

Study Groups: Group 1: Topical sucralfate; Group 2: Placebo

Statistical results:

- ITT analysis done for endpoint(s): N
- **The only endpoints identified were variation in ulcer size (%) and healing rate at 90 days. They were not identified as primary vs secondary.**
- 43/45 (95.6%) in Group 1 healed vs 5/46 (10.9%) in Group 2, chi squared = 65.45, $p < 10^{-5}$
- Good/excellent efficacy in 42/45 (93.3%) of Group 1 vs 4/46 (8.7%) in Group 2 (chi square = 68.89, p <.00001)
- Mean (SD) inflammation at baseline and end treatment, PP analysis: Group 1: 1.27 (0.5) to 0.154 (0.37) vs Group 2: 1.26 (.49) to 0.756 (0.43), p <.00001 in favor of Group 1
- Mean (SD) exudates at baseline and end treatment, PP analysis: Group 1: 0.978 (0.5) to 0.103 (0.5) vs Group 2: 0.978 (.26) to 0.267 (0.45), p = .0102 in favor of Group 1
- Mean (SD) swelling at baseline and end treatment, PP analysis: Group 1: 0.889 (0.49) to 0.026 (0.16) vs Group 2: 0.957 (.36) to 0.222 (0.15), p = NS
- Mean (SD) pain at baseline and end treatment, PP analysis: Group 1: 1.07 (0.81) to 0.282 (0.51) vs Group 2: 0.935 (.65) to 0.600 (0.5), p = .0023 in favor of Group 1
- Mean (SD) burning at baseline and end treatment, PP analysis: Group 1: 0.778 (0.79) to 0.128 (0.34) vs Group 2: 0.609 (0.65) to 0.311 (0.47), p = .0463 in favor of Group 1
- Group 1 had significantly better variation in ulcer size from 100% at baseline to approximately 2% at End vs Group 2 from 100% to approximately 77% (p = NS)
- Group 1 had significantly better score difference in granulation from 0 at baseline to 2.5 at End vs Group 2 from 0 to 1 (p = NS)

Power calculation:

- Was a power calculation for the primary endpoint reported, and if so, was it appropriate? N

Adjustment for multiplicity of statistical testing:

- Was an adjustment reported if there were more than 1 secondary endpoints statistically tested? N

Blinding:

- Were patients blinded from treatment? Y

For Peer Review

- Were assessing investigators blinded from patient treatment? Y

Allocation concealment:

- Was allocation concealment carried out? N

Lost to follow-up/discontinued/withdrawn:

- 9/100 (9%)
  - 5/50 (10%) withdrawn from Group 1
  - 4/50 (8%) withdrawn from Group 2

131. Ukat A, Konig M, Vanscheidt W, Munter KC. Short-stretch versus multilayer compression for venous leg ulcers: a comparison of healing rates. *J Wound Care.* 2003;12(4):139-143.

Study Groups: Group 1: multilayer elastic compression; Group 2: short-stretch compression

Statistical results:

- ITT analysis done for primary endpoint(s): Y
- **Primary endpoint:** complete healing by 84 days: Group 1 healed faster than Group 2, p = .03, HR: 2.9, 95% CI: 1.1-7.5 with 13 (30%) in Group 1 healed and 10 (22%) in Group 2 healed by week 12
- Younger wounds healed faster than older wounds, p = .01
- Wounds with unaffected periwound skin healed faster than those those with edema, dermatosclerosis, or erythema, p = .03
- Healing times were shorter in Hamburg than in Freiburg, p = .001
  - There was no significant relationship between treatment and center, p = .713, -2 log L criterion

Power calculation:

- Was a power calculation for the primary endpoint reported, and if so, was it appropriate? Y, N

Adjustment for multiplicity of statistical testing:

- Was an adjustment reported if there were more than 1 secondary endpoints statistically tested? N

Blinding:

- Were patients blinded from treatment? N
- Were assessing investigators blinded from patient treatment? N

Allocation concealment:

- Was allocation concealment carried out? N

Lost to follow-up/discontinued/withdrawn:

- 23/89 (25.8%) total
  - 8/44 (18.2%) withdrawn from Group 1
  - 15/45 (25.8%) withdrawn from Group 2

132. van Gent WB, Hop WC, van Praag MC, Mackaay AJ, de Boer EM, Wittens CH. Conservative versus surgical treatment of venous leg ulcers: a prospective, randomized, multicenter trial. *J Vasc Surg.* 2006;44(3):563-571.

Study Groups: Group 1: Subfascial endoscopic perforating vein surgery, when indicated, combined with superficial venous system surgery + compression therapy; Group 2:

For Peer Review

Compression therapy

Primary Endpoints:

- Ulcer-free period (total percentage of days during follow-up a patient had no ulceration) from 0 to 36 months divided into intervals of 3 months each

Statistical results:

- ITT analysis done for primary endpoint(s): N
- **Primary endpoint:** Patients with ulcers existing ≤4 months before randomization had a longer ulcer-free rate during follow-up (p<.001)
    - Age, sex, ulcer size, diabetes, deep vein thrombosis, and ulcer location did not influence ulcer-free period (p = NS)
    - Median ulcer-free period was 72% in Group 1 vs 53% in Group 1 (p=.11)
    - When individual patient-follow was assessed, no significant difference between groups (p = .14)
    - First-time ulcer and one center had a positive effect on the ulcer-free period for both groups (p <.001 and p = .02); DVI had no effect (p = NS)
    - Recurrent ulceration in Group 1 had a higher ulcer-free rate (62% vs 33%, p = .02)
    - Medially located ulcers in Group 1 had a higher ulcer-free rate (78% vs 24%, p =.02)
    - Patients >75 years in Group 1 had a higher ulcer-free rate (70% vs 24%, p = .004), but this was related to recurrent ulceration
    - Patients with recurrent ulceration and/or medially located ulcers in Group 1 had a higher ulcer-free rate (p = .045)
- 83/94 (88.3%) ulcers in Group 1 healed vs 73/102 (71.6%), p = NS
    - Median and mean time to heal were 11 and 4.2 mos in Group 1 vs 15 and 5.7 mos in Group 2 (p = NS)
    - In both groups, healing rates significantly influenced by first-time ulcers (p <.001), ulcers of >4 mos duration (p <.001), ulcer size (p <.001), DVI (p = .03), DVT in the medical history (p =.03), and center (p = .002)
    - Medial and recurrent ulcers had significantly higher healing rates in Group 1 (p =.04 and p = .01)
- No significant difference was found in recurrence rates (22% in Group 1 vs 23% in Group 2) (p = NS)
    - First-time ulcers had significantly lower recurrence rates than recurrent ulcers (p =.01)
    - Sex, age, DVI, diabetes, DVT, ulcer location, surgery, ulcer size, and center had no significant effect on recurrence (p = NS)

Power calculation:

- Was a power calculation for the primary endpoint reported, and if so, was it appropriate? Y, N

Adjustment for multiplicity of statistical testing:

- Was an adjustment reported if there were more than 1 secondary endpoints statistically tested? N

For Peer Review

Blinding:

- Were patients blinded from treatment? N
- Were assessing investigators blinded from patient treatment? N

Allocation concealment:

- Was allocation concealment carried out? N

Lost to follow-up/discontinued/withdrawn among 200 ulcerated legs (170 patients):

- 41/200 (20.5%) total
    - 23/97 (23.7%) withdrawn from Group 1
    - 18/103 (17.5%) ulcers withdrawn from Group 2

133. Vanscheidt W, Harding K, Teot L, Siebert J. Effectiveness and tissue compatibility of a 12-week treatment of chronic venous leg ulcers with an octenidine based antiseptic--a randomized, double-blind controlled study. *Int Wound J.* 2012;9(3):316-323.

Study Groups: Group 1: octenidine dihydrochloride/phenoxyethanol; Group 2: Ringer solution

Statistical results:

- ITT analysis done for primary endpoint(s): N
- **Primary endpoint:** median time to heal, days: 92 in Group 1 vs 87 in Group 2, p = .952
- Percentage of complete healed in 12 weeks: 30.6 in Group 1 (n = 49) vs 32.0 in Group 2 (n = 50), p = .882
- Percentage of responders at visit 1
    - ≤6 cm^2: 34.6 in Group 1 (n = 26) vs 44.8 in Group 2 (n = 29), p = .440
    - >6 cm^2: 26.1 in Group 1 (n = 23) vs 14.3 in Group 2 (n = 21), p = .332
- Percentage of responders at visit 1
    - ≤6 months: 29.6 in Group 1 (n = 27) vs 42.9 in Group 2 (n = 28), p = .308
    - >6 months: 31.8 in Group 1 (n = 22) vs 18.2 in Group 2 (n = 22), p = .296
- Percentage of responders at visit 1
    - ≤6 cm^2 and ≤6 months: 36.8 in Group 1 (n = 19) vs 45.0 in Group 2 (n = 20), p = .605
    - ≤6 cm^2 and >6 months: 28.6 in Group 1 (n = 7) vs 44.4 in Group 2 (n = 9), p = .515
    - >6 $cm^2$and ≤6 months: 12.5 in Group 1 (n = 8) vs 37.5 in Group 2 (n = 8), p = .248
    - >6 $cm^2$and >6 months: 33.3 in Group 1 (n = 15) vs 0.0 in Group 2 (n = 13), p = .022
    - The wound size (≤6 cm^2 or >6 cm^2) and duration of the target ulcer (≤6 months or >6 months) had no significant influence on the time to complete wound closure in both Groups, p = .947 and p = .978, respectively
- Mean (SD) surface area of the target ulcer, cm^2 at visit 1: 6.68 (4.76) in Group 1 vs 6.98 (5.51) in Group 2, p = NS
- Percentage of mean surface area between baseline (V1) and last visit (V6): Decreased 37.9 (-2.53 cm^2) in Group 1 vs 40.3 (-2.81 cm^2)in Group 2, p = .769
- At Visit 4 mean change in wound surface area was more pronounced in Group 2 (4.37 cm^2) vs Group 1 (5.01 cm^2), p= NS
- At Visit 5 mean change in wound surface area was more pronounced in Group 1 (3.77 cm^2) vs Group 2 (4.4 cm^2), p= NS

- At Visit 6 mean change in wound surface area was more pronounced in Group 1 (3.97 cm^2) vs Group 2 (4.23 cm^2), p= .843
- Mean (SD) reduction in wound surface area and wound perimeter, cm^2/ week
  - Wound surface area: -1.12 (1.50) in Group 1 vs -0.61 (0.63) in Group 2, p = .237
  - Wound perimeter: -0.11 (0.09) in Group 1 vs -0.06 (0.05) in Group 2, p = .108
- Physicians´ global assessment of efficacy, very good and good, %: 57.1 vs 60%, p = .874
- Patients´ global assessment of efficacy, very good and good, %: 65.3 vs 58.0%, p = .24

Power calculation:

- Was a power calculation for the primary endpoint reported, and if so, was it appropriate? Y, N

Adjustment for multiplicity of statistical testing:

- Was an adjustment reported if there were more than 1 secondary endpoints statistically tested? N

Blinding:

- Were patients blinded from treatment? Y
- Were assessing investigators blinded from patient treatment? Y

Allocation concealment:

- Was allocation concealment carried out? N

Lost to follow-up/discontinued/withdrawn:

- 18/126 (14.3%) total
  - 7/60 (11.7%) withdrawn from Group 1
  - 11/66 (16.7%) withdrawn from Group 2

134. Vanscheidt W, Sibbald RG, Eager CA. Comparing a foam composite to a hydrocellular foam dressing in the management of venous leg ulcers: a controlled clinical study. *Ostomy Wound Manage.* 2004;50(11):42-55.

Study Groups: Group 1: Foam composite dressing; Group 2: hydrocellular foam dressing

Statistical results:

- ITT analysis done for primary endpoint(s): N
- **Primary endpoints were not identified**
- 13 patients (29%) had 19 possibly dressing-related AEs in Group 1 vs 15 patients (29%) having 18 in Group 2 p = 1.00
- Mean (SD) dressing changes and wear time differences between groups, p = NS
  - Group 1: treated for 58.6 (28.3) days, dressings changed 11.4 (6.5) times for a wear time of 5.6 (1.3) days
  - Group 2: treated for 52.8 (26.3) days, dressings changed 10.1 (6.3) times for a wear time of 5.6 (1.2) days
- Investigator-reported satisfaction: Group 1 performed significantly better than Group 2 in terms of conformability (p = .05), absence of sensitizing reactions (p = .02), and ease of application (p = .01). There were no significant differences (p = NS) in terms of exudate absorption, protection of surrounding skin, nontraumatic dressing removal, and ease of removal
- Ulcer healing:

For Peer Review

  - Median rate of healing (cm^2/wk): Group 1: 0.41; Group 2: 0.43, p = .13
  - Median percentage change per week: Group 1: 7.3%; Group 2: 6.1%, p = .27
  - Percentage of ulcers completely healed by study completion: Group 1: 38.2%; Group 2: 38.5%, p = 0.96
  - Reported maceration of surrounding skin: 22% of Group 1 dressing changes; 27% of Group 2, p = NS
  - Mean (SD) time to complete healing: Group 1: 66 (3.4); Group 2: 72.6 (3.1), p = .47
- Surrounding skin had healed or shown marked improved in 30 (54%) of Group 1 and 19 (36%) of Group 2, p = .03
  - 37 (67%) of Group 1 and 33 (63%) of Group 2 healed or showed marked improvement (p = NS)
- 65.5% of Group 1 and 42.9% of Group 2 reported reduced pain, p = NS
  - Cochran-Mantel-Haenszel test adjusted for number of no response/uncertain pain outcomes ad found a significantly greater percentage of Group 1 reported reduced pain (p = .0075)
- 7.3% of Group 1 and 18.4% of Group 2 reported increased pain, p = .10

Power calculation:
- Was a power calculation for the primary endpoint reported, and if so, was it appropriate? N

Adjustment for multiplicity of statistical testing:
- Was an adjustment reported if there were more than 1 secondary endpoints statistically tested? N

Blinding:
- Were patients blinded from treatment? N
- Were assessing investigators blinded from patient treatment? N

Allocation concealment:
- Was allocation concealment carried out? N

Lost to follow-up/discontinued/withdrawn: 31/107 (29%) total

135. Vanscheidt W, Ukat A, Horak V, et al. Treatment of recalcitrant venous leg ulcers with autologous keratinocytes in fibrin sealant: a multinational randomized controlled clinical trial. *Wound Rep Regen.* 2007;15(3):308-315.

Study Groups: Group 1: autologous keratinocytes in fibrin seal + compression therapy (n = 116); Group 2: SOC (n = 109)

Statistical results:
- ITT analysis done for primary endpoint(s): Y
- **Primary endpoint:** Median (95% CI) time to complete healing up to 6 months (days)
  - ITT population: Group 1: 176 (114-184); Group 2: not reached (201-infinity); HR: 0.33 (0.19-0.55); p <.0001
  - PP population: Group 1: 158 (112-184); Group 2: not reached (201-infinity); HR: 0.33 (0.20-0.55); p <.0001
  - Subgroup – age of ulcer ≤12 months: Group 1: 184 (158-infinity); Group 2: 206

For Peer Review

(193-infinity); HR: 0.26 (0.12-0.58); p = .0004
  - Subgroup – age of ulcer >12 months: Group 1: 112 (79-infinity); Group 2: not reached (133-infinity); HR: 0.47 (0.24-0.92); p = .0224
  - Subgroup – size of ulcer (cm^2), size 2-10: Group 1: 154 (86-182); Group 2: 206 (193-infinity); HR: 0.34 (0.19-0.63); p = .0003
  - Subgroup – size of ulcer (cm^2), size >10: Group 1: not reached (149-infinity); Group 2: not reached (–); HR: 0.19 (0.05-0.69); p = .0051
- Number of patients with complete healing
  - ITT population: Group 1: 44 (38.3%); Group 2: 24 (22.4%); HR (95% CI): 0.47 (0.26-0.84); p =.0106
  - Subgroup – age of ulcer ≤12 months: Group 1: 17 (38.3%); Group 2: 10 (15.4%); HR (95% CI): 0.46 (0.19-1.10); p =.0788
  - Subgroup – age of ulcer >12 months: Group 1: 25 (51.0%); Group 2: 14 (35.9%); HR (95% CI): 0.54 (0.23-1.27); p =.1560
  - Subgroup – size of ulcer (cm^2), size 2-10: Group 1: 32 (47.8%); Group 2: 21 (31.3%); HR (95% CI): 0.50 (0.25-1.01); p =.0520
  - Subgroup – size of ulcer (cm^2), size >10: Group 1: 12 (25.5%); Group 2: 3 (7.7%); HR (95% CI): 0.24 (0.06-0.94); p =.0300

Power calculation:
- Was a power calculation for the primary endpoint reported, and if so, was it appropriate? Y, N

Adjustment for multiplicity of statistical testing:
- Was an adjustment reported if there were more than 1 secondary endpoints statistically tested? N

Blinding:
- Were patients blinded from treatment? N
- Were assessing investigators blinded from patient treatment? N

Allocation concealment:
- Was allocation concealment carried out? N

Lost to follow-up/discontinued/withdrawn: 12 (5.3%) total were excluded from PP analyses.

136. Vitse J, Bekara F, Byun S, Herlin C, Teot L. A double-blind, placebo-controlled randomized evaluation of the effect of low-level laser therapy on venous leg ulcers. *Int J Low Extrem Wounds.* 2017;16(1):29-35.

Study Groups: Group 1: low-level laser therapy (n = 13); Group 2: sham light instead of laser (placebo) (n = 11)

Statistical results:
- ITT analysis done for primary endpoint(s): Y
- **Primary endpoint:** No. (%) of complete wound closure by 12 weeks: 3 (23) in Group 1 vs 2 (18%)in Group 2, p = 1.0
- Mean (SD) of decrease in ulcer area from day 1, day 7 of the pre-procedure wound care phase, cm^2; mean of percentage of area reduction: 10.13 (6.23) to 8.55 (4.54); 13.00 (12.73) in Group 1, p = .02 vs 11.30 (7.55) to 9.93 (6.57); 10.93 (13.09) in Group 2, p =

For Peer Review

.06; 10.67 (6.74) to 9.11 (5.48); 12.01 (12.65) in All Groups (n = 24), p = .002
    - No patients in both Groups demonstrated a decrease in ulcer area of 30% or greater in this phase
    - Without significant differences in the change of VAS (Visual Analogue Scale) in this phase , Group 1, p = .32; Group 2, p = .62; and overall, p = .26 (Only baseline values, none at day 7)
- Mean (SD) of ulcer area at baseline, 12 weeks, absolute area reduction, cm^2; Mean of percentage area reduction: 8.55 (4.54), 2.28 (2.78), 6.26 (3.66); 77.10 (25.70) in Group 1, p <.0001 vs 9.93 (6.57), 3.21 (5.54), 6.72 (5.06); 69.20 (40.03) in Group 2, p = .0013. And overall without significant difference, p = .80 (No values)
- Mean (SD) of ulcer pain VAS rating at baseline, 12 weeks, absolute ulcer VAS rating reduction; Mean of percentage Ulcer VAS rating reduction: 44.69 (23.93), 1.15 (4.16), 43.54 (25.93); 86.23 (33.87) in Group 1, p <.0001 vs 30.45 (20.04), 4.73 (8.59), 25.73 (20.56); 70.94 (45.87) in Group 2, p = .002.
    - Mean (SD) of ulcer pain VAS rating at 4 weeks; absolute VAS increase from end of treatment to 4 weeks; absolute VAS reduction from baseline of treatment to 4 weeks: 7.69 (13.36); 6.54, p >.05; -37.00, p <.01 in Group 1 vs 15.27 (17.88); 10.54, p >.05; -15.18, p <.05 in Group 2.

Power calculation:
- Was a power calculation for the primary endpoint reported, and if so, was it appropriate? N

Adjustment for multiplicity of statistical testing:
- Was an adjustment reported if there were more than 1 secondary endpoints statistically tested? N

Blinding:
- Were patients blinded from treatment? Y
- Were assessing investigators blinded from patient treatment? Y

Allocation concealment:
- Was allocation concealment carried out? N

Lost to follow-up/discontinued/withdrawn: None reported

137. Vowden P, Romanelli M, Peter R, Bostrom A, Josefsson A, Stege H. The effect of amelogenins (Xelma) on hard-to-heal venous leg ulcers. *Wound Repair Regen.* 2006;14(3):240-246.

Study Groups: Group 1: amelogenin protein (n = 62); Group 2: aqueous carrier alone (control) (n = 61)

Statistical results:
- ITT analysis done for primary endpoint(s): N
- **Primary endpoint:** Mean PAR, but no p values reported in any of the results.

Power calculation:
- Was a power calculation for the primary endpoint reported, and if so, was it appropriate? Y, N

Adjustment for multiplicity of statistical testing:

For Peer Review

- Was an adjustment reported if there were more than 1 secondary endpoints statistically tested? N

Blinding:

- Were patients blinded from treatment? N
- Were assessing investigators blinded from patient treatment? Y

Allocation concealment:

- Was allocation concealment carried out? N

Lost to follow-up/discontinued/withdrawn: None reported.

138. Vowden P, Romanelli M, Price P. Effect of amelogenin extracellular matrix protein and compression on hard-to-heal venous leg ulcers. *J Wound Care*. 2007;16(5):189-195.

Study Groups: Group 1: Amelogenin + high compression therapy; Group 2: high compression therapy

Statistical results:

- ITT analysis done for primary endpoint(s): Y
- **Primary endpoint:** Mean PAR (SD) for Group 1 was 33.1% (49.7%) vs 11.1% (46.6%) (p = .06) and the percentage of improved ulcers at the final visit was 47.5% in Group 1 vs 19.5% in Group 2 (p = .01)
    - Multiple regression revealed statistically significant reduction in PAR in Group 1 (p = .03); the mean and 95% CI for the difference between groups was 22.04 (1.01% to 43.1%) (p = .03)
    - For PP data, Group 1 Mean PAR was 50.33% (:41.9) and Group @ was 21.2% (50.5%) (p = .03)
- Group 1 had greater pain reduction (p = .01); the mean and 95% CI between groups at final visit was -1.59 (-2.8 to -0.3)
- There was a significant reduction in the number of patients with moderate to high exudate levels during the 12 weeks in Group 1 (p = .01) but not in Group 2.

Power calculation:

- Was a power calculation for the primary endpoint reported, and if so, was it appropriate? Y, Y

Adjustment for multiplicity of statistical testing:

- Was an adjustment reported if there were more than 1 secondary endpoints statistically tested? N

Blinding:

- Were patients blinded from treatment? N
- Were assessing investigators blinded from patient treatment? N

Allocation concealment:

- Was allocation concealment carried out? N

Lost to follow-up/discontinued/withdrawn:

- 24/83 (28.9%) total
    - 9/42 (21.4%) withdrawn from Group 1
    - 15/41 (36.6%) withdrawn from Group 2

For Peer Review

139. Weller CD, Evans SM, Staples MP, Aldons P, McNeil JJ. Randomized clinical trial of three-layer tubular bandaging system for venous leg ulcers. *Wound Repair Regen.* 2012;20(6):822-829.

Study Groups: Group 1: 3-layer tubular bandaging; Group 2: short-stretch bandaging

Statistical results:

- ITT analysis done for primary endpoint(s): Y
- **Primary endpoint:** Mean (SD, median, range) of reduction of ulcer size at 12 weeks, %: Group 1 n = 23, Group 2 n = 22
  - 82.4 (32.4, 100, -11 to 100) in Group 1 vs 70.1 (37.5, 89, -8 to 100) in Group 2, p = .23
  - Mean (SD) of reduction in size, log (Area 2/Area 1), where area 1 is size of ulcer at baseline, Area 2 is Ulcer size at end of treatment: 2.0 (1.4) in Group 1 vs 1.7 (1.5) in Group 2, p = .52
- No. (%) of healed at ≤ 12 weeks: 17 (74) in Group 1 vs 10 (46) in Group 2, p = .056
- Mean (SD) of Percentage of treatment visits with tolerability = Very comfortable/ comfortable: 95 (21) in Group 1 vs 89 (29) in Group 2, p = .13
- No. (%) reported tolerance at all treatment visits: 21 (91) in Group 1 vs 16 (73) in Group 2, p = .10
- Mean (SD) percentage of treatment visits with compliance = Yes: 96 (9) in Group 1 vs 98 (5) in Group 2, p = .74
- No. (%) reported 100% bandage compliance: 19 (79) in Group 1 vs 17 (81) in Group 2, p = .65
- No. (%) of AEs: 7 (58) in Group 1 vs 5 (42) in Group 2, p = .56
- Mean (95% CI) of ulcer size over 12 weeks, cm^2: 1.9 (0 to 3.9) in group 1 vs 3.4 (1.4 to 5.5), p = NS
  - The mean (95% CI) of Group differences in absolute ulcer size reduction, cm^2: 0.6 (1.9 to 3.2), p = NS
- Each 1 cm^2 increase in ulcer size at baseline, the likelihood of healing was reduced by 25%, p = .005
- Each week of ulcer duration, the likelihood of healing was reduced by 19%, p = .02
- Time to healing and proportion of healed (%):
  - 5 weeks: 24 in both Groups, 10 weeks: 55 in Group 1 vs 40 in Group 2
  - 12 weeks: 64 in Group 1 and 49 in Group 2, p = .18
- 27 wounds that healed, there were 6 recurrences and all occurred within 5 weeks of healing. A higher proportion of Group 1 recurred, p = NS
- RR for recurrence in Group 1 vs Group 2: 1.2 (95% CI = 0.3 to 5.3), p = NS
- Mean (SD) (Mean difference, 95% CI) SF 36 scores (Group 1 n = 13, Group 2 n= 16)
  - Physical component summary score: 4.5 (7.2) in Group 1 vs 3.6 (8.7) in Group 2, (0.9, -5.2 to 7.1), p = .76
  - Mental component summary score: 4.6 (7.2) in Group 1 vs 6.9 (16.2) in Group 2, (-2.2, -12.2 to 7.8), p = .65
- Mean (SD) (Mean difference, 95% CI) CWIS scores (Group 1 n = 12, Group 2 n= 12):
  - Well-being: 17.6 (26.0) in Group 1 vs 17.0 (25.0) in Group 2, (0.6, -24.0 to 22.2), p

For Peer Review

= .96
    - Physical symptoms and daily living: 18.9 (19.9) in Group 1 vs 16.7 (26.1) in Group 2, (2.3, -17.4 to 21.9), p = .81
    - Social life: 9.8 (23.1) in Group 1 vs 4.5 (19.1) in Group 2, (5.3, -13.3 to 23.8), p = .56
    - Quality of life: 3.7 (11.5) in Group 1 vs 14.5 (20.5) in Group 2, (-10.8, -25.1 to 3.5), p = .13
    - Healed participants had higher means (SD) change in scores: 9.6 (10.8), n = 20 vs unhealed participant -2.2 (13.8), n = 9, p = .02
- Median (range) total cost, $: 200 (25 to 300) in Group 1 vs 618 (158 to 618) in Group 2, p = .0001

Power calculation:
- Was a power calculation for the primary endpoint reported, and if so, was it appropriate? Y, N

Adjustment for multiplicity of statistical testing:
- Was an adjustment reported if there were more than 1 secondary endpoints statistically tested? N

Blinding:
- Were patients blinded from treatment? N
- Were assessing investigators blinded from patient treatment? N

Allocation concealment:
- Was allocation concealment carried out? Y

Lost to follow-up/discontinued/withdrawn:
- 3/45 (6.7%) total
    - 0/23 (0%) withdrawn from Group 1
    - 3/22 (13.6%) withdrawn from Group 2

140. White J, Ivins N, Wilkes A, Carolan-Rees G, Harding KG. Non-contact low-frequency ultrasound therapy compared with UK standard of care for venous leg ulcers: a single-centre, assessor-blinded, randomised controlled trial. *Int Wound J*. 2016;13(5):833-842.

Study Groups: Group 1: Noncontact low-frequency ultrasound therapy + SOC (n = 17); Group 2: SOC (n = 19)

Statistical results:
- ITT analysis done for primary endpoint(s): Y
- **Primary endpoint:** No statistically significant difference was observed in the percentage change in wound area between the 2 groups at week 13 while controlling for the baseline wound area, which had a significant influence as a covariate (p <.001)
    - Adjusted (planned)
        - Mean (SD) percentage change in area: Group 1: -46.6 (38.1); Group 2: -39.2 (38.0); Difference (95% CI): -7.4 (-33.4 to 18.6), p = .565
        - Actual change in wound area (cm^2): Group 1: -6.2 (5.5); Group 2: -5.3 (5.5); Difference (95% CI): -0.9 (-4.7 to 2.9), p = .618
    - Nonadjusted

For Peer Review

  - Mean (SD) percentage change in area: Group 1: -47.8 (40.3); Group 2: -38.2 (35.6); Difference (95% CI): -9.6 (-35.3 to 16.1), p = .453
  - Actual change in wound area (cm^2): Group 1: -5.6 (6.6); Group 2: -5.8 (6.2); Difference (95% CI): 0.1 (-4.2 to 4.5), p = .954
- 9 ulcers in Group 1 (52.9%) and 7 in Group 1 (36.8%) reached at least 50% closure after 8 weeks of treatment (p = NS)
- No significant difference was observed in the median number of infections per patient between Group 1 [2 (IQR: 0-3)] and Group 2 [2 (IQR: 1-3)], p =.346
  - 11 patients in Group 1 and 16 in Group 2 experienced at least 1 infection, p = .225
- Differences in mean (SD) score changes for Cardiff Wound Impact Schedule domain scores between end of study and baseline based on a scale of 0-100.
  - Well-being: Group 1: 8.3 (16.2); Group 2: 8.0 (16.2); Difference (95% CI): 0.4 (-10.6 to 11.4), p = .943
  - Physical symptoms and daily living: Group 1: 10.4 (10.7); Group 2: 5.8 (10.7); Difference (95% CI): 4.6 (-2.6 to 11.9), p = .204
  - Social life: Group 1: 3.0 (19.8); Group 2: -0.5 (19.8); Difference (95% CI): 3.5 (-10.0 to 17.0), p = .602
- Change in median (IQR) HRQoL, based on scale from 0 to 10: Group 1; -1 (-2 to 2) vs Group 2: 0: -1 to 1, p = .490
- Change in median (IQR) QoL satisfaction, based on scale from 0 to 10: Group 1; 0 (-2 to 1) vs Group 2: 0: -1 to 1, p = .452.
- Group 1 reported a mean (SD) reduction in pain score (adjusted for week 5 pain score which had a significant influence of p <.001), while Group 1 reduced by -5.27 (14.84)
  - Difference of -9.08 (95% CI: -19.23 to 1.06), p = .078

Power calculation:

- Was a power calculation for the primary endpoint reported, and if so, was it appropriate? Y, Y

Adjustment for multiplicity of statistical testing:

- Was an adjustment reported if there were more than 1 secondary endpoints statistically tested? N

Blinding:

- Were patients blinded from treatment? N
- Were assessing investigators blinded from patient treatment? Y

Allocation concealment:

- Was allocation concealment carried out? Y

Lost to follow-up/discontinued/withdrawn: None reported.

141. Wild T, Eberlein T, Andriessen A. Wound cleansing efficacy of two cellulose-based dressings. *Wounds.* 2010;6(3):14-21.

Study Groups: Group 1: Bio-cellulose dressing (n = 20); Group 2: hydrofiber dressing (Control) (n = 20)

Statistical results:

For Peer Review

- ITT analysis done for all endpoints: Y
- **No primary endpoints identified.**
- Mean PAR at 4 weeks larger in Group 1 (43.5%, t = 0.082) vs Group 2 (17.9%, t = 0.008), but "when comparing the reduction in ulcer size between the groups, no statistically significant difference was shown."
    - When discarding the outliers (1 ulcer in each group), the results remain not statistically significant (Group 1: t = 0.657 vs Group 2: t = 0.345).
- Mean percent reduction in yellow tissue (SD)
    - Group 1: Day 7: 9.9 (5.6), t = 0.000; Day 14: 32.9 (16.7), t = 0.000; Day 21: 42.9 (20.1), t = 0.000; Day 28: 58.8 (26.5), t = 0.000
    - Group 2: Day 7: 5 (2.7), t = 0.000; Day 14: 23.3 (9.8), t = 0.000; Day 21: 34.8 (10.7), t = 0.000; Day 28: 45.7 (11), t = 0.000
    - Reduction significantly larger in Group 1 at day 7 (t = 0.020) and at day 14 (t = 0.45) compared to Group 2

Power calculation:
- Was a power calculation for the primary endpoint reported, and if so, was it appropriate? N

Adjustment for multiplicity of statistical testing:
- Was an adjustment reported if there were more than 1 secondary endpoints statistically tested? N

Blinding:
- Were patients blinded from treatment? N
- Were assessing investigators blinded from patient treatment? N

Allocation concealment:
- Was allocation concealment carried out? Y

Lost to follow-up/discontinued/withdrawn: None reported.

142. Wille JJ, Burdge JJ, Pitttelkow MR. Rapid healing of chronic venous stasis leg ulcers treated by the application of a novel serum-free cultured autologous epidermis. *Wound Repair Regen.* 2011;19(4):464-474.

Study Groups: Group 1: living serum-free cultured epidermal autograft (n = 10); Group 2: 4-layer compression wrap (control) (n = 5)

Statistical results:
- ITT analysis done for primary endpoint(s): N
- **Primary endpoints:** wounds healed, fraction of wound closure, and fraction of wound volume closure
    - No. (%) of complete wound closure: 8/10 (80) in Group 1 vs 1/5 (20) in Group 1, p <.022
    - Rate of fraction of wound area healed, cm^2/day: 0.80 in Group 1 vs 0.11 in Group 2, ANCOVA/T3SOS, p <.001; Mann-Whitney, p <.019
    - Rate of fraction of wound volume healed, cm^3/day: 0.26 in Group 1 vs 0.05 in Group 2, ANCOVA/T3SOS, p <.001; Mann-Whitney, p <.003
- No significant correlation between the time of wound closure and initial size area in

For Peer Review

Group 1, r = .55, and insufficient data in Group 2 to make a reliable analysis (1 value).

- No significant correlation between the time of wound closure and initial wound volume in Group 1, r = .57, and insufficient data in Group 2 to make a reliable analysis (single response).
- The closure events occurred significantly faster in Group 1 vs Group 2, Kaplan-Meier, p <.022; Fisher's or Barnard's exact test, p <.038

Power calculation:

- Was a power calculation for the primary endpoint reported, and if so, was it appropriate? Y, N

Adjustment for multiplicity of statistical testing:

- Was an adjustment reported if there were more than 1 secondary endpoints statistically tested? N

Blinding:

- Were patients blinded from treatment? N
- Were assessing investigators blinded from patient treatment? N

Allocation concealment:

- Was allocation concealment carried out? N

Lost to follow-up/discontinued/withdrawn: 7/22 (31.8%) total

143. Zamboni P, Cisno C, Marchetti F, et al. Minimally invasive surgical management of primary venous ulcers vs. compression treatment: a randomized clinical trial. *Eur J Vasc Endovasc Surg.* 2003;25(4):313-318.

Study Groups: Group 1: Minimally invasive surgery (surgical hemodynamic correction) (n = 21 patients with 23 VLUs); Group 2: compression (n = 24 patients with 24 VLUs)

Statistical results:

- ITT analysis done for primary endpoint(s): Y
- **Primary endpoint not identified, but power calculation based on recurrence rate.**
- Healing rate was 100% in a median (range) period of 31 (17-53) days in Group 1 vs 63 (21-180) days in Group 2, p <.005
- Recurrences: 2 (9%) in Group 1 vs 9 (38%) in Group 2 (p < .05)
- Median air phlethysmographic parameters in Group 1 and their differences compared to baseline (preoperative)
    - Venous volume (ml): preoperative: 187; 6 months postoperative: 124, p <.001; 3 years: 152, p =NS; postoperative ulcer recurrence: 140, p = NS
    - Venous filling index (ml/s): preoperative: 5.4; 6 months postoperative: 2.9, p <.001; 3 years: 5, p =NS; postoperative ulcer recurrence: 7.5, p = NS
    - Ejection fraction (%): preoperative: 56; 6 months postoperative: 54, p = NS; 3 years: 51, p =NS; postoperative ulcer recurrence: 48, p = NS
    - Residual volume fraction (%):preoperative: 46; 6 months postoperative: 33, p <.001; 3 years: 31, p <.001; postoperative ulcer recurrence: 65, p <.001
    - For Group 2, only the venous volume improved significantly from 173 (122-268) to 142 (91-260) ml, p <.05 at 6 months, which was not sustained at 3 years. The other parameters were not significant at any time point (p = NS)

For Peer Review

- Median score differences (3 years – baseline) of SF 36 questionnaire
  - Physical functioning: Group 1: 15 p <.05 vs Group 2: 8, p <.05
  - Role-physicial limitation: Group 1: 22 p <.05 vs Group 2: 6, p = NS; ; significant difference between groups, p <.05
  - Pain: Group 1: 29, p <.05 vs Group 2: 21, p <.05
  - General health: Group 1: 15, p <.05 vs Group 2: 6, p <.05
  - Vitality: Group 1: 13, p <.05 vs Group 2: 2, p = NS; significant difference between groups, p <.05
  - Social functioning: Group 1: 14, p <.05 vs Group 2: 8, p <.05; significant difference between groups, p <.05
  - Role-emotional limitation: Group 1: 22, p <.05 vs Group 2: 3, p = NS; significant difference between groups, p <.05
  - Mental health: Group 1: 13, p <.05 vs Group 2: 3, p = NS; significant difference between groups, p <.05

Power calculation:
- Was a power calculation for the primary endpoint reported, and if so, was it appropriate? Y, N

Adjustment for multiplicity of statistical testing:
- Was an adjustment reported if there were more than 1 secondary endpoints statistically tested? N

Blinding:
- Were patients blinded from treatment? N
- Were assessing investigators blinded from patient treatment? N

Allocation concealment:
- Was allocation concealment carried out? N

Lost to follow-up/discontinued/withdrawn: None reported.

144. Zhou YT, Zhao XD, Jiang JW, Li XS, Wu ZH. Ozone gas bath combined with endovenous laser therapy for lower limb venous ulcers: a randomized clinical trial. *J Invest Surg.* 2016;29(5):254-259.

Study Groups: Group 1: Endovenous laser therapy (EVLT) + ozone gas bath (n = 50); Group 2: EVLT alone (n = 42)

Statistical results:
- ITT analysis done for primary endpoint(s): Y
- **Primary endpoints:** completion of local ozone gas bath treatment (no analysis), the feasibility of GSV cannulation (no analysis, unless patient satisfaction is the measurement), completion of GSV endovenous laser ablation, and complete epithelialization
  - Number (%) of complete occlusion of the treated vein detected by Doppler (n = 50 in Group 1, n = 42 in Group 2) Chi square was used:
    - 1 month: 50 (100) in Group 1 vs 42 (100) in Group 2, p = 1.00
    - 3 month: 48 (96.00) in Group 1 vs 40 (95.24) in Group 2, p = .38
    - 6 month: 43 (86.00) in Group 1 vs 37 (88.10) in Group 2, p = .23

For Peer Review

        - 12 month: 40 (80.00) in Group 1 vs 34 (81.00) in Group 2, p = .21
    - Healing rates at 12 months, No. (%) (n = 50 in Group 1, n = 42 in Group 2) Chi square was used:
        - 46 (92) healed, 4 (8) unhealed in Group 1 vs 32 (76.19) healed, 10 (23.8) unhealed in Group 2; comparing the ulcer healing in both Groups, p = .03
            - Group total: 78 healed, 14 unhealed (Ulcer healing 84.78%)
    - Healing rates at 24 months, No. (%) (n = 46 in Group 1, n = 32 in Group 2) Chi square was used:
    - 3 (6.52) recurred, 43 (93.48) non-recurred in Group 1 vs 8 (25.00) recurred, 24 (75.00) recurred in Group 2; comparing the ratio of ulcer recur in both Groups, p = .03
        - Group total: 11 recurred, 67 non-recurred (Ratio of recur 14.10%)
- Number (%) of patients satisfaction assessment (n = 50 in Group 1, n = 42 in Group 2) Chi square was used:
    - 45 (90.00) Satisfaction, 5 (10.00) Unsatisfaction in Group 1 vs 30 (71.43) Satisfaction, 12 (28.57) Unsatisfaction in Group 2; comparing the ratio of satisfaction in both Groups, p = .03
        - Group total: 75 Satisfaction, 17 Unsatisfaction (Ratio of Satisfaction 81.52%)

Power calculation:

- Was a power calculation for the primary endpoint reported, and if so, was it appropriate? N

Adjustment for multiplicity of statistical testing:

- Was an adjustment reported if there were more than 1 secondary endpoints statistically tested? N

Blinding:

- Were patients blinded from treatment? N
- Were assessing investigators blinded from patient treatment? N

Allocation concealment:

- Was allocation concealment carried out? N

Lost to follow-up/discontinued/withdrawn: None reported.

For Peer Review

WILEY

Color Charge Form: *Wound Repair and Regeneration*

Return this form to: wrr_edoffice@wiley.com

Dear Author:
If you have supplied one or more components for use in your article that you would like published in color, please complete this form and return it to the editorial office within 48 hours.

NOTE: If color is "informative" and information conveyed through the use of color will not translate into gray scale, the figure will require color publication in print with associated color page charges. Color figures intended for gray scale print publication should not have color descriptors in the figure legends.

Color charges are $800.00 per page; there is a discounted rate of $600.00 per page for WHS, ETRS and JSWH members (to receive discount, corresponding author must be an active member of the Wound Healing Society, excluding students and trainees). For more information about becoming a WHS member, please visit the following site http://www.woundheal.org/membership/index.html.

This represents an estimate of charges. We estimate that one figure will require one page of color. Maximum costs will not exceed the Estimated Charges and may be less.
(Please Complete & Please Print)

For Peer Review

Name: Marissa J. Carter
Manuscript No.: WRR-20-07-0205.R1
Manuscript Title: Assessing the Uncertainty of Treatment Outcomes in a Previous Systematic Review of Venous Leg Ulcer Randomized Controlled Trials: Additional Secondary Analysis
WHS Membership Number (if applicable):
Address: Strategic Solutions, Inc.<br>37 Voyager Lane Bozeman, MT 59718
Phone Number: 307 272-9906
Email Address: mcarter@strategic-solutions-inc.com

Component(s) in Article	Charge(s)	Quantity	Estimated Charge(s) (in USD)
Color Figure Charge **Standard**	$[**800**] per Page	0	$0
Color Figure Charge for **WHS, ETRS, and JSWH Members**	$[**600**] per Page	0	$0
			$ 0 = Total Estimated

Figure No.	Color in print and online	Color Online/ Black & White in Print	Black & white ONLY
1			
2			
3			
4			
5			
6			
7			

Place an '**X**' in **column 1** for **all figures** you want to appear as **color in print and online**. Figures paid for to appear as color in print will also appear online in color for free.

OR

Place an '**X**' in column 2 for all figures you want to appear in **color online and b/w in print**

[Note if you select this option, your figure captions should not contain color desriptors]

OR

Place an '**X**' in **column 3** for **all figures** you want to appear as **black and white only** at no charge.

[Note if you select this option, your figure captions should not contain color descriptors]

Remember: There should be **only one 'X' per figure**

Verify your agreement to pay by signing and dating below. You will be sent an invoice from Wiley:

Signature: Date: 1-19-21
